# Supplementary material for: Ligand control of regioselectivity in palladium-catalyzed heteroannulation reactions of 1,3-Dienes
Source: Nat Commun. 2024 Jun 26;15:5433. doi: 10.1038/s41467-024-49803-y (PMC11208576; doi:10.1038/s41467-024-49803-y)
Supplement: Supplementary file 1 — Supplementary Information [file 41467_2024_49803_MOESM1_ESM.pdf]

Ligand Control of Regioselectivity in Palladium-Catalyzed Heteroannulation Reactions of 1,3-Dienes

Dasha Rodina, Jakub Vaith, Shauna M. Paradine*

Department of Chemistry, University of Rochester, 414 Hutchison Hall, 120 Trustee Road, Rochester, NY 14627 (USA).

*Corresponding Author: sparadin@ur.rochester.edu

Supplementary Information

Contents

Supplementary Methods....S2

General Information....S2

Methods....S2

Materials and Reagents....S2

Instrumentation....S2

Abbreviations....S3

Preparation of *N*-Sulfonyl *o*-haloanilines....S3

Preparation of 1,3-Dienes....S4

Reaction Condition Optimization....S12

Solvent Screen....S13

Base Screen....S15

Reaction Tolerance....S16

Additional Experiments....S17

Ligand Structure-selectivity Relationship Studies....S19

Reaction Scope....S21

Bromoaniline Scope....S22

1,3-Diene Scope....S28

Tosyl Group Deprotection....S34

Gram Scale Reaction....S35

Reactivity with Mesyl-Protected Anilines....S35

Method Limitations....S36

Confirmation of Regioselectivity X-Ray Structure of 3ad....S37

Linear Regression Modeling....S39

Computational Studies....S39

Computational studies of the regioselectivity determining carbopalladation step with ligand L2 as a model ligand 3-selective ligand S40
NMR Spectra of New Compounds S81
Supplementary References S114

Supplementary Methods

General Information

Methods

All reactions were carried out under a nitrogen atmosphere in flame–dried glassware with magnetic stir bar unless otherwise specified. Stainless steel gas-tight syringes were used to transfer air- and moisture-sensitive liquids. Reactions were monitored by thin–layer chromatography (TLC) on pre–coated silica gel 60 F254 glass–supported plates from EMD, and visualized under UV light (254 nm) or with p–anisaldehyde followed by heating. Flash chromatography was performed using SiliaFlash P60 (230–400 mesh, SiliCycle). Reported product yields were determined based on material isolated after column purification.

Materials and Reagents

Reagents were used as obtained from commercial suppliers without further purification unless otherwise noted. $Pd_2(dba)_3$ was purchased from Strem. Reaction solvents – Tetrahydrofuran (THF), diethyl ether (Et_2O), dichloromethane (DCM) – were purchased from Fisher and dried by passing through columns of activated alumina (Pure Process Technology SPS). Pyridine (Fisher), acetonitrile (Fisher), N,Ndimethylacetamide (DMA), toluene (Fisher) and anisole (Alfa Aesar) were used without further purification. *N,N*-Dimethylformamide (Fisher) was stored over 3Å molecular sieves. Deuterated solvents $CDCl_3$, DMSO-d_6, acetone-d_6 (Cambridge Isotope Laboratories), and HPLC solvents (Fisher) were used without further purification. Synthesized dienes were stored at –30 °C; some decomposed over time and were used soon after isolation (see Preparation of 1,3-Dienes section for storage conditions of specific substrates).

Instrumentation

Proton nuclear magnetic resonance (^1H NMR) and proton-decoupled carbon nuclear magnetic resonance (^{13}C NMR) spectra were recorded on a Bruker DPX–400 instrument (operating at 400 MHz for ^1H, 100 MHz for ^{13}C) or a Bruker DPX–500 instrument (operating at 500 MHz for ^1H, 125 MHz for ^{13}C) at ambient temperature. Proton resonances are referenced to residual protium in the NMR solvent. Carbon resonances are referenced to the carbon resonances of the NMR solvent. Data are represented as follows: chemical shift, multiplicity (br = broad, s = singlet, d = doublet, t = triplet, q = quartet, m = multiplet, app = apparent), coupling constants (*J*) in Hertz (Hz), integration. Mass spectral (MS) data were obtained on a Thermo Fisher Q Exactive Plus spectrometer (University of Rochester Medical Center Mass Spectrometry Resource Laboratory). Microanalysis samples were weighed with a PerkinElmer Model AD6000 Autobalance and their compositions were determined with a PerkinElmer 2400 Series II Analyzer. High-performance liquid chromatography (HPLC) analysis was performed using a Shimadzu Prominence–I LC–2030 Plus system with commercially available Restek Pinnacle DB Cyano column (5 µm, 150x4.6 mm). All samples were eluted with 2% ^{i}PrOH/hexanes at an elution rate of 1 mL/min and detected

at 254 nm. Total run time was 25 min. X-ray crystallography data were collected by Dr. William W. Brennessel at the X-ray Crystallographic Facility of the University of Rochester, Rochester, NY 14627 (USA).

Abbreviations

aq. = aqueous, cm^{-1} = wavenumber, DBU = 1,8-Diazabicyclo[5.4.0]undec-7-ene, DCM = dichloromethane, DMA = *N,N*-dimethylacetamide, DMF = *N,N*-dimethylformamide, DMSO = dimethylsulfoxide, equiv. = equivalents, h = hours, min = minutes, m/z = mass to charge ratio, r.r. = regioisomeric ratio, rt = room temperature, sat = saturated, TBACl = tetrabutylammonium chloride, THF = tetrahydrofuran, TBS = *tert*-butyldimethylsilyl, Ts = toluenesulfonyl.

Preparation of *N*-Sulfonyl *o*-haloanilines

General procedure for synthesis of *N*-sulfonyl *o*-haloanilines: Prepared according to a literature procedure.[1] To a solution of corresponding *o*-haloaniline (1.0 equiv.) in pyridine (ca. 1.0M) was added corresponding sulfonyl chloride (1.0 equiv.) and the reaction mixture was stirred overnight at rt. Water (5-10 mL per mL of pyridine) was then added to the reaction mixture, resulting in precipitation of crude product. This mixture was filtered, then the solid residue was washed with water and recrystallized in refluxing EtOH/hexanes to afford the pure product.

N-Tosyl *o*-bromoanilines **1b-1i** were prepared according to the standard procedure (above). We have previously reported full characterization for all of these compounds.[1]

_N_-(2-bromophenyl)-4-methylbenzenesulfonamide (1a)

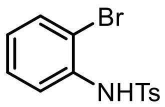

Prepared according to general procedure. 2-Bromoaniline (6.00 g, 34.9 mmol, 1.0 equiv.), pyridine (30 mL, 1.16M), and *p*-toluenesulfonyl chloride (6.66 g, 32.3 mmol, 1.0 equiv.) were used. The recrystallized product was obtained as a white crystalline solid (9.54 g, 29.3 mmol, 84%).

^{1}H NMR (400 MHz, $CDCl_3$) δ 7.69–7.65 (m, 3H), 7.42 (d, *J* = 8.0 Hz, 1H), 7.28 (dd, *J* = 8.0, 7.6 Hz, 1H), 7.22 (d, *J* = 8.4 Hz, 2H), 7.00–6.96 (m, 2H), 2.38 (s, 3H). Spectral data agree with that reported in the literature.[1]

_N_-(2-chlorophenyl)-4-methylbenzenesulfonamide (1a-Cl)

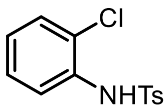

Prepared according to general procedure. 2-Chloroaniline (3.64 g, 28.5 mmol, 1.0 equiv.), pyridine (25 mL, 1.14M), and *p*-toluenesulfonyl chloride (5.43 g, 28.5 mmol, 1.0 equiv.) were used. The recrystallized product was obtained as a white crystalline solid (4.56 g, 16.2 mmol, 57%).

^{1}H NMR (500 MHz, $CDCl_3$) δ 7.68–7.62 (m, 3H), 7.25–7.19 (m, 4H), 7.03 (td, *J* = 7.8, 1.6 Hz, 1H), 6.97 (s, 1H), 2.37 (s, 3H). Spectral data agree with that reported in the literature.[2]

methyl 4-bromo-3-((4-methylphenyl)sulfonamido)benzoate (1j)

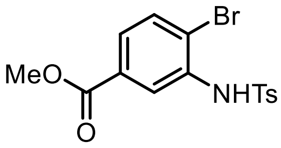

Prepared according to general procedure. Methyl 3-amino-4-bromobenzoate (2.00 g, 8.69 mmol, 1.0 equiv.), pyridine (7 mL, 1.24M), and *p*-toluenesulfonyl chloride (1.66 g, 8.69 mmol, 1.0 equiv.) were used. The recrystallized product was obtained as a white crystalline solid (1.40 g, 3.64 mmol, 42%).

^{1}H NMR (400 MHz, $CDCl_3$) δ 8.29 (d, *J* = 2.0 Hz, 1H), 7.68 (d, *J* = 8.0 Hz, 2H), 7.63 (dd, *J* = 8.4, 2.0 Hz, 1H), 7.50 (d, *J* = 8.4 Hz, 1H), 7.23 (d, *J* = 8.0 Hz, 2H), 7.01 (s, 1H), 3.93 (s, 3H), 2.38 (s, 3H). Spectral data agree with that reported in the literature.[3]

***N*-(2-bromophenyl)methanesulfonamide (1a-Ms)**

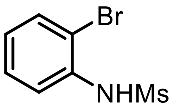

Prepared according to general procedure. 2-Bromoaniline (4.00 g, 23.3 mmol, 1.0 equiv.), pyridine (22 mL, 1.06M), and *p*-toluenesulfonyl chloride (2.67 g, 23.3 mmol, 1.0 equiv.) were used. The recrystallized product was obtained as a white crystalline solid (4.72 g, 18.9 mmol, 81%).

^{1}H NMR (400 MHz, $CDCl_3$) δ 7.66 (d, *J* = 8.1 Hz, 1H), 7.59 (d, *J* = 8.0 Hz, 1H), 7.35 (dd, *J* = 8.1, 7.7, 1 Hz, 1H), 7.08 (dd, *J* = 8.0, 7.7 Hz, 1H), 6.81 (s, 1H), 3.01 (s, 3H). Spectral data agree with that reported in the literature.[4]

***N*-(2-bromophenyl)-4-(trifluoromethyl)benzenesulfonamide (1k)**

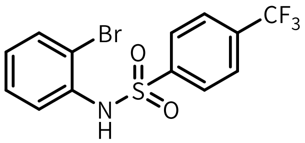

Prepared according to general procedure. 2-Bromoaniline (1.05 g, 6.13 mmol, 1.0 equiv.), pyridine (6.0 mL, 1.02 M), and 4-trifluorophenylsulfonyl chloride (1.50 g, 6.13 mmol, 1.0 equiv.) were used. The recrystallized product was obtained as a white crystalline solid (2.09 g, 5.49 mmol, 89%).

^{1}H NMR (400 MHz, $CDCl_3$) δ 7.87 (d, J = 8.3 Hz, 2H), 7.70 (d, J = 8.2 Hz, 3H), 7.44 (dd, J = 8.1, 1.2 Hz, 1H), 7.34 – 7.29 (m, 1H), 7.03 (tt, J = 9.1, 4.6 Hz, 1H), 6.99 (bs, 1H).

^{13}C NMR (100 MHz, $CDCl_3$) δ 142.44, 135.24 (q, $J_{C\text{-}F}$ = 33.1 Hz), 134.90, 134.05, 132.94, 128.94, 127.97, 127.31, 126.37 (q, $J_{C\text{-}F}$ = 3.7 Hz), 123.68, 116.56.

^{19}F NMR (376 MHz, $CDCl_3$) δ -63.5 (3F).

HRMS (ESI) m/z calculated for $C_{13}H_9BrF_3NO_2S$ $[M+H]^+$: 379.9561, found 379.9561

Preparation of 1,3-Dienes

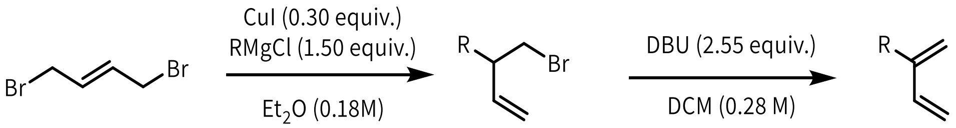

2-(bromomethyl)but-3-en-1-ylbenzene

Prepared according to modified literature procedure.[5] Copper iodide (404.6 mg, 2.12 mmol, 0.30 equiv.) and 1,4-dibromo-2-butene (1.50 g, 7.01 mmol, 1.00 equiv.) was taken up in diethyl ether

(40 mL, 0.18 M) in a round bottomed flask equipped with stir bar and cooled to -10°C. Benzyl magnesium chloride (5.25 mL, 10.5 mmol, 1.5 equiv.) was added dropwise over 5 min to the reaction mixture which then was left to stir at room temperature overnight. The reaction mixture was diluted with sat. aq. NH_4Cl (15 mL) extracted with Et_2O (3 x 10 mL), combined organic layers washed with brine (3 x 5 mL) three times and dried over $MgSO_4$. Upon concentration, colorless oil (1.16 g, 5.50 mmol) was used immediately without further purification.

2-benzyl-1,3-butadiene (2c)

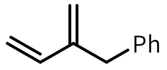

2-(bromomethyl)but-3-en-1-ylbenzene (1.16 g, 5.50 mmol, 1.00 equiv.) was taken up in DCM (20 mL, 0.28 M), DBU (2.08 mL, 14.0 mmol, 2.55 equiv.) was added to the reaction mixture which then was heated up to 40°C for 12h. The reaction mixture was diluted with water (10 mL), extracted with DCM (3 x 5 mL), dried over $MgSO_4$, filtered, and concentrated under reduced pressure. Crude material was purified via column chromatography on SiO2 using pentane. Pure product was isolated as colorless oil (239.0 mg, 1.66 mmol, 24% over two steps).

^{1}H NMR (400MHz, $CDCl_3$) δ 7.38 – 7.13 (m, 5H), 6.44 (ddd, J = 17.5, 10.8, 0.7 Hz, 1H), 5.25 (dd, J = 17.6, 0.9 Hz, 1H), 5.17 (td, J = 1.4, 0.7 Hz, 1H), 5.07 (dq, J = 10.8, 1.0 Hz, 1H), 4.92 (p, J = 1.5 Hz, 1H), 3.57 (s, 2H). Spectral data agree with that reported in the literature.[5]

buta-1,3-dien-2-ylbenzene (2d)

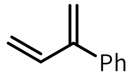

1-bromo-2-phenyl-3-butene (1.16 g, 5.50 mmol, 1.00 equiv.) was taken up in DCM (20 mL, 0.28 M), DBU (2.08 mL, 14.0 mmol, 2.55 equiv.) was added to the reaction mixture which then was heated up to 40°C for 12h. The reaction mixture was diluted with water (10 mL), extracted with DCM (3 x 5 mL), dried over $MgSO_4$, filtered, and concentrated under reduced pressure. Crude material was purified via column chromatography on SiO_2 using pentane. Pure product was isolated as colorless oil (255.0 mg, 1.96 mmol, 28% over two steps).

^{1}H NMR (400MHz, $CDCl_3$) δ 7.35 – 7.26 (m, 5H), 6.66 – 6.57 (m, 1H), 5.29(d, J = 1.7 Hz, 1H), 5.22 – 5.16 (m, 3H). Spectral data agree with that reported in the literature.[5]

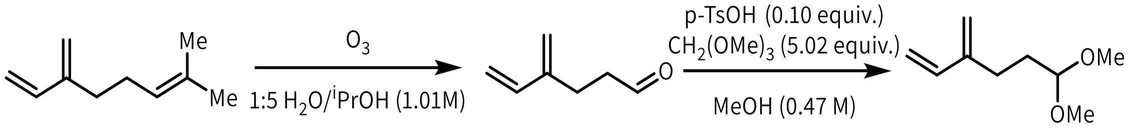

4-methylenehex-5-enal

Prepared according to modified literature procedure.[1] Myrcene **2a** (3.44 mL, 20.1 mmol, 1.00 equiv.) was taken up in 1:5 $H_2O/^iPrOH$ (20 mL, 1.01 M) in a pear-shaped flask equipped with stir bar. Ozone gas was bubbled into this solution for 50 min. Upon completion, argon was bubbled through reaction mixture for 10 min. The reaction mixture was diluted with water and extracted with Et_2O (3 x 5 mL), then the combined organic layers were washed with sat. aq. NH_4Cl and brine (3 x 5 mL) three times and dried over $MgSO_4$. Upon concentration, colorless oil (1.06 g, 9.43 mmol) was used immediately without further purification

6,6-dimethoxy-3-methylenehex-1-ene (2e)

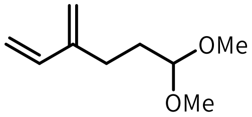

Prepared according to modified literature procedure.[6] 4-methylenehex-5-enal (1.06 g, 9.43 mmol, 1.00 equiv.), p-toluenesulfonic acid (175.0 mg, 0.908 mmol, 0.10 equiv.), was taken up in methanol (20 mL, 0.47 M) in a round bottomed flask equipped with stir bar. Trimethylorthoformate (4.84 g, 45.6 mmol, 5.02 equiv.) was added to the reaction mixture which then was heated at reflux for 1 h, cooled to 23 °C and diluted with water (20 mL) and extracted with Et_2O (3 x 10 mL). The combined organic layers were washed with brine, dried over Na_2SO_4, filtered, and concentrated under reduced pressure. Crude material was purified via column chromatography on SiO2 using 0% → 8% EtOAc/hexanes. Pure product was isolated as colorless oil (927.0 mg, 5.93 mmol, 63%).

^{1}H NMR (400MHz, $CDCl_3$) δ 6.38 (dd, J = 17.6, 10.9 Hz, 1H), 5.25 (d, J = 17.6 Hz, 1H), 5.07 (d, J = 10.9 Hz, 1H), 5.03 (s, 1H), 5.01 (s, 1H), 4.40 (t, J = 5.7 Hz, 1H), 3.33 (s, 6H), 2.27 (t, J = 7.9 Hz, 2H), 1.78–1.82 (m, 2H). Spectral data agree with that reported in the literature.[6]

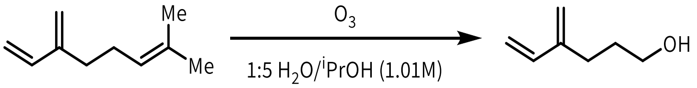

4-methylene-hex-5-en-1-ol

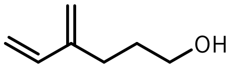

Prepared according to modified literature procedure.[1] Myrcene **2a** (3.44 mL, 20.1 mmol, 1.00 equiv.) was taken up in 1:5 $H_2O/^iPrOH$ (20 mL, 1.01 M) in a pear-shaped flask equipped with stir bar. Ozone gas was bubbled into this solution for 50 min. Upon completion, argon was bubbled through reaction mixture for 10 min. $NaBH_4$ (467.0 mg 12.3 mmol, 0.610 equiv.) was added, then the reaction mixture stirred for 5 minutes. The reaction mixture was diluted with water and extracted with Et_2O (3 x 5 mL), then the combined organic layers were washed with sat. aq. NH_4Cl and brine (3 x 5 mL) three times and dried over $MgSO_4$. Crude material was purified via column chromatography on SiO_2 using 5% → 25% EtOAc/hexanes. Pure product was isolated as a colorless oil (1.06 g, 9.43 mmol, 47%).

^{1}H NMR (500 MHz, $CDCl_3$) δ 6.38 (dd, J = 17.6, 10.8 Hz, 1H), 5.27 (dd, J = 17.6, 0.5 Hz, 1H), 5.08 (d, J = 10.8 Hz, 1H), 5.04 (d, J = 0.5 Hz, 1H), 5.01 (s, 1H), 3.69 (td, J = 6.4, 5.5 Hz, 2H), 2.31 (t, J = 7.3 Hz, 2H), 1.75–1.81 (m, 2H), 1.26 (t, J = 5.5 Hz, 1H). Spectral data agree with that reported in the literature.[1]

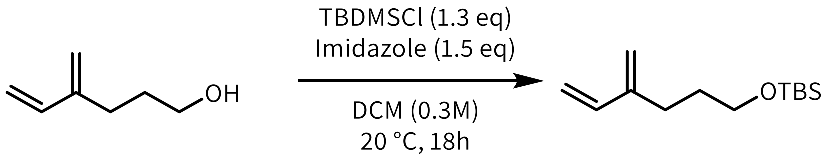

***tert*-butyldimethyl((4-methylenehex-5-en-1-yl)oxy)silane (2f)**

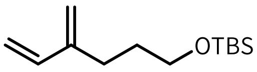

4-methylene-hex-5-en-1-ol (343.2 mg 3.06 mmol 1.00 equiv.), tert-butyldimethylsilyl chloride (609.8 mg 4.05 mmol, 1.30 equiv.), and imidazole (317 mg, 4.65 mmol, 1.50 equiv.) were dissolved in DCM (10 mL, 0.30 M) and left to stir at rt (20 °C) for 18h. Reaction mixture was quenched with water (5 mL), washed with sat. aq. $NaHCO_3$ (10 mL) and extracted with hexanes (3 x 5 mL), then the combined

organic layers were dried over Na_2SO_4. Crude material was purified via column chromatography on SiO_2 with hexanes to afford product as a colorless oil (0.401 g, 1.77 mmol, 58%).
^1H NMR (400MHz, $CDCl_3$) δ 6.40 (dd, J = 17.6, 10.8 Hz, 1H), 5.28 (d, J = 17.6 Hz, 1H), 5.08 (d, J = 10.8 Hz, 1H), 5.04-5.02 (m, 2H), 3.67 (t, J = 6.4 Hz, 2H), 2.30 (t, J = 6.4 Hz, 2H), 1.77-1.70 (m, 2H),0.93 (s, 9H), 0.08 (s, 6H). Spectral data agree with that reported in the literature.[7]

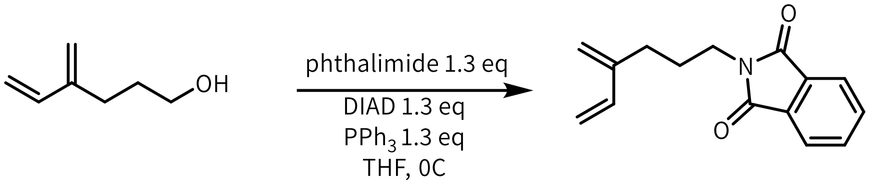

2-(4-methylenehex-5-en-1-yl)isoindoline-1,3-dione (2g)

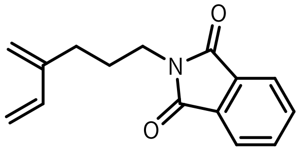

4-methylene-hex-5-en-1-ol (320.0 mg, 2.85 mmol, 1.00 equiv.), triphenylphosphine (971.8 mg, 3.70 mmol, 1.30 equiv.), phthalimide (544.4 mg, 3.70 mmol, 1.30 equiv.) and 20 mL of THF (0.146 M) were cooled to 0°C. To the cold reaction mixture added diisopropyl azodicarboxylate (728.5 µL, 3.70 mmol, 1.30 equiv.). Upon consumption of starting material as monitored by TLC, the reaction mixture was diluted with 15 mL of water and extracted with hexanes (3 x 5 mL). The organic layer was washed with brine (3 x 5 mL) and dried over $MgSO_4$. Crude material was purified via column chromatography on SiO_2 using hexanes → 5% EtOAc/hexanes. Pure product was isolated as a white solid (466.0 mg, 1.93 mmol, 68%).
^1H NMR (500 MHz, $CDCl_3$) δ 7.85 (dd, J = 5.4, 3.0 Hz, 2H), 7.77 – 7.67 (m, 2H), 6.37 (ddd, J = 17.7, 11.2, 1.2 Hz, 1H), 5.21 (dq, J = 17.6, 0.7 Hz, 1H), 5.11 – 5.01 (m, 3H), 3.78 – 3.68 (m, 2H), 2.34 – 2.23 (m, 2H), 1.98 – 1.84 (m, 2H). Spectral data agree with that reported in the literature.[8]

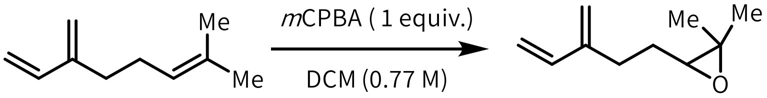

6,7-epoxymyrcene (2h)

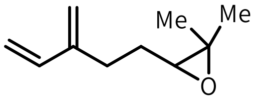

Prepared according to literature procedure.[1] Myrcene (10.6 g, 77.8 mmol 1.00 equiv.) was taken up in CH_2Cl_2 (100 mL, 0.77 M) in a round bottom flask. After cooling to 0°C, *m*CPBA (17.0 g, 77.8 mmol, 1.00 equiv.) was added, then the reaction mixture stirred for 10 min at 0°C. Upon completion, the reaction mixture was quenched with NaOH (2.00 M aq. sol.) and extracted with DCM (3 x 10 mL). The combined organic layers were washed with sat. aq. NaS_2O_3, water, and brine (3 x 7 mL), then dried over $MgSO_4$. Crude material was purified via column chromatography on SiO_2 using 5% → 10% EtOAc/hexanes. Pure product was isolated as colorless oil (6.28 g, 41.2 mmol, 53%).

^1H NMR (400 MHz, $CDCl_3$) δ 6.38 (dd, *J* = 17.5, 11.0 Hz, 1 H), 5.24 (d, *J* = 17.5 Hz, 1 H), 5.08 (d, *J* = 11.0 Hz, 1 H), 5.02 (s, 1 H), 5.05 (s, 1 H), 2.76 (t, *J* = 6.2 Hz, 1 H), 2.40–2.48 (m, 1 H),

2.28–2.36 (m, 1 H), 1.71–1.76 (m, 2 H), 1.31 (s, 3 H), 1.26 (s, 3 H). Spectral data agree with that reported in the literature.[1]

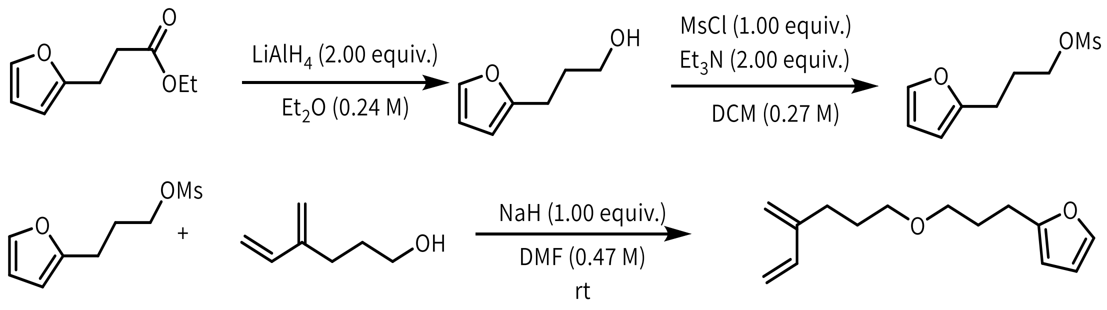

2-(3-((4-methylenehex-5-en-1-yl)oxy)propyl)furan (2i)

Ethyl 3-(furan-2-yl)propanoate (2.00 g, 11.9 mmol, 1.00 equiv.) was taken up in diethyl ether (50 mL, 0.23 M) in a round bottomed flask equipped with stir bar and cooled to 0°C.Lithium aluminum hydride (893.0 mg, 23.8 mmol, 2.00 equiv.) was added in portions over 15 min to the reaction mixture which then was left to stir at room temperature for 2 h. The reaction mixture was diluted with sat. aq. NH_4Cl (15 mL) extracted with Et_2O (3 x 10 mL), combined organic layers washed with brine (3 x 5 mL) three times and dried over $MgSO_4$. Upon concentration, colorless oil (1.26 g, 10.0 mmol) was used immediately without further purification.

3-Furan-2-yl-propan-1-ol (1.26 g, 10.0 mmol, 1.00 equiv.) was taken up in DCM (60 mL, 0.17 M) in a round bottomed flask equipped with stir bar and cooled to 0°C. Triethylamine (20.0 mmol, 2.80 mL, 2.00 equiv.) and methanesulfonyl chloride (775.5 µL, 10.0 mmol, 1.00 equiv.) was added to the reaction mixture which then was left to stir at 0°C for 2 h. The reaction mixture was quenched with sat. aq. $NaHCO_3$ (15 mL) extracted with DCM (3 x 10 mL), washed with water (3 x 10 mL) combined organic layers washed with brine (3 x 5 mL) and dried over $MgSO_4$. filtered, and concentrated under reduced pressure. Upon concentration, colorless oil (1.97 g, 9.63 mmol, 81% yield over two steps) was used immediately without further purification.

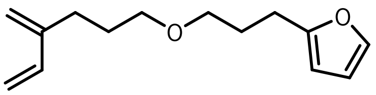

4-methylene-hex-5-en-1-ol (1.06 g, 9.43 mmol, 1.00 equiv.) was taken up in DMF (20 mL, 0.47 M) in a dry round bottomed flask equipped with stir bar at room temperature. Sodium hydride 60% dispersion in mineral oil (373.2 mg, 9.43 mmol, 1.00 equiv.) and 3-(furan-2-yl)propyl methanesulfonate (1.97 g, 9.63 mmol, 1.02 equiv.) was added to the reaction mixture which then was left to stir at room temperature overnight. The reaction mixture was quenched with sat. aq. NH_4Cl (5 mL) extracted with diethyl ether (3 x 10 mL), washed with water (3 x 10 mL) combined organic layers washed with brine (3 x 5 mL) and dried over $MgSO_4$. filtered, and concentrated under reduced pressure. Crude material was purified via column chromatography on SiO_2 using 10% EtOAc/hexanes. Pure product was isolated as clear oil (653.0 mg, 2.96 mmol, 25% yield over three steps).

^{1}H NMR (500 MHz, $CDCl_3$) δ 7.32 (d, J = 14.4 Hz, 1H), 6.38 (dd, J = 17.6, 10.8 Hz, 1H), 6.28 (s, 1H), 6.00 (s, 1H), 5.25 (d, J = 17.6 Hz, 1H), 5.06 (d, J = 10.9 Hz, 1H), 5.02 (d, J = 8.0 Hz, 2H), 3.44 (t, J = 6.1 Hz, 4H), 2.72 (t, J = 7.5 Hz, 2H), 2.29 (t, J = 7.6 Hz, 2H), 1.97 – 1.87 (m, 2H), 1.82 – 1.73 (m, 2H).

^{13}C NMR (101 MHz, $CDCl_3$) δ 155.96, 146.04, 140.97, 138.96, 115.98, 113.46, 110.21, 105.03, 70.54, 69.94, 28.33, 28.30, 27.92, 24.82.

HRMS (ESI) m/z calculated for $C_{14}H_{20}O_2$ $[M+H]^+$: 221.1536, found 221.1537.

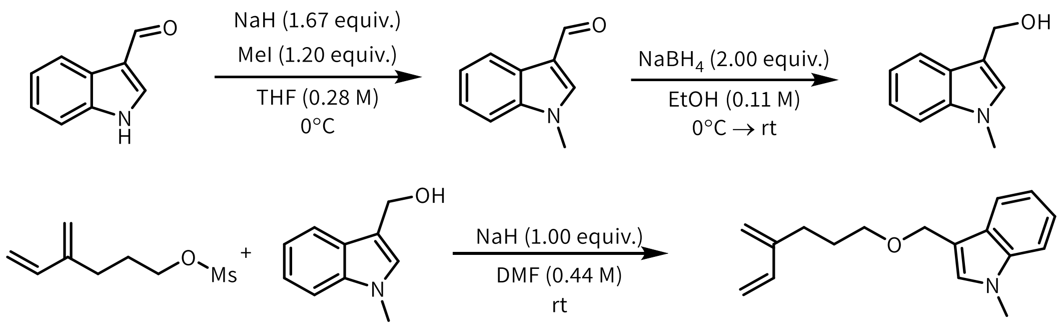

1-methyl-3-(((4-methylenehex-5-en-1-yl)oxy)methyl)-1*H*-indole (2j)

3-Indole-carboxaldehyde (4.02 g, 27.7 mmol, 1.00 equiv.) was taken up in THF (100 mL, 0.28 M) in a round bottomed flask equipped with stir bar and cooled to 0°C. Sodium hydride (1.85 g, 46.3 mmol, 1.67 equiv.) was added in portions over 15 min to the reaction mixture which then was left to stir at 0°C for 1 h. Methyl iodide (2.06 mL, 33.2 mmol, 1.20 equiv.) was added dropwise to the reaction mixture which then was warmed to room temperature and left to stir overnight. The reaction mixture was diluted with sat. aq. NH_4Cl (15 mL) extracted with Et_2O (3 x 10 mL), combined organic layers washed with brine (3 x 5 mL) three times and dried over $MgSO_4$. Upon concentration, colorless oil (4.29 g, 27.0 mmol) was used immediately without further purification.

N-methyl-3-formylindole (715.3 mg, 4.44 mmol, 1.00 equiv.) was taken up in ethanol (40 mL, 0.11 M) in a round bottomed flask equipped with stir bar and cooled to 0°C. Sodium borohydride (335.9 mg, 8.88 mmol, 2.00 equiv.) was added in portions over 15 min to the reaction mixture which then was warmed up to room temperature over 3 h. The reaction mixture was diluted with water (15 mL) extracted with Et_2O (3 x 10 mL), combined organic layers washed with brine (3 x 5 mL) three times and dried over $MgSO_4$. Upon concentration, colorless oil (710.3 mg, 4.40 mmol) was used immediately without further purification.

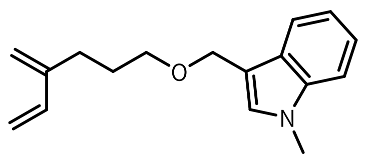

(1-methyl-1H-indol-3-yl)methanol (71 mg, 4.40 mmol, 1.00 equiv.) was taken up in DMF (10 mL, 0.44 M) in a round bottomed flask equipped with stir bar. Sodium hydride 60% dispersion in mineral oil (176.0 mg, 4.40 mmol, 1.00 equiv.) and 4-methylenehex-5-en-1-yl methanesulfonate (837 mg, 4.40 mmol, 1.00 equiv.) was added to the reaction mixture which then was left to stir at room temperature overnight. The reaction mixture was quenched with sat. aq. NH_4Cl (5 mL) extracted with diethyl ether (3 x 10 mL), washed with water (3 x 10 mL) combined organic layers washed with brine (3 x 5 mL) and dried over $MgSO_4$. filtered, and concentrated under reduced pressure. Crude material was purified via column chromatography on SiO_2 using 10% EtOAc/hexanes. Pure product was isolated as clear oil (753.2.0 mg, 2.95 mmol, 64% yield over three steps).

^1H NMR (400MHz, $CDCl_3$) δ 7.73 (d, J = 7.9 Hz, 1H), 7.32 (d, J = 8.1 Hz, 1H), 7.26 (t, J = 7.5 Hz, 1H), 7.16 (t, J = 7.4 Hz, 1H), 7.06 (s, 1H), 6.38 (dd, J = 17.6, 10.8 Hz, 1H), 5.25 (d, J = 17.6 Hz, 1H), 5.06 (d, J = 10.8 Hz, 1H), 5.01 (d, J = 10.5 Hz, 2H), 4.72 (s, 2H), 3.78 (s, 3H), 3.55 (t, J = 6.4 Hz, 2H), 2.34 – 2.28 (m, 2H), 1.87 – 1.77 (m, 2H).

^{13}C NMR (100 MHz, $CDCl_3$) δ 146.1, 139.0, 137.4, 128.4, 127.8, 121.9, 119.5, 119.4, 115.9, 113.4, 112.2, 109.4, 69.5, 64.8, 32.8, 28.4, 28.0.

HRMS (ESI) m/z calculated for $C_{17}H_{21}NO$ $[M+H]^+$: 256.1696, found 256.1694.

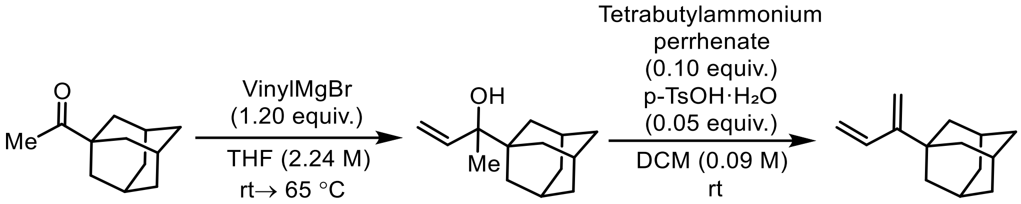

2-(1-adamantyl)-1,3-butadiene (2k)

1-Adamantyl methyl ketone (400 mg, 2.24 mmol, 1.00 equiv.) was taken up in 1 mL of THF and added to a vinylmagnesium bromide (2.70 mL, 2.69 mmol, 1.20 equiv.) in a round bottomed flask equipped with stir bar at room temperature. The reaction mixture was warmed to 65 °C and left to stir overnight. The reaction mixture was diluted with sat. aq. NH_4Cl (15 mL) extracted with Et_2O (3 x 10 mL), combined organic layers washed with water (3 x 5 mL) three times and dried over $MgSO_4$. Upon concentration, white solid (0.38 g, 1.84 mmol) was used without further purification.

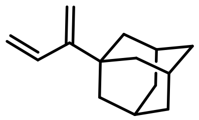

Tetrabutylammonium perrhenate (241.0 mg, 0.49 mmol, 0.10 equiv.) and *p*-toluenesulfonic acid monohydrate (47.0 mg, 0.25 mmol, 0.05 equiv.) were taken up in DCM (30 mL) in a round bottomed flask equipped with stir bar at room temperature. 2-(Adamantan-1-yl)but-3-en-2-ol (1.00 g, 4.84 mmol, 1.00 equiv.) was added in DCM (20 mL, 0.09 M). The reaction was left to stir at room temperature for seven hours. The reaction mixture was diluted with sat. aq. $NaHCO_3$ (40 mL) extracted with DCM (3 x 30 mL) and dried over $MgSO_4$. filtered, and concentrated under reduced pressure. Crude material was purified via column chromatography on SiO_2 using pentanes. Pure product was isolated as pale-yellow oil (611.8 mg, 3.24 mmol, 54% yield over two steps).

^1H NMR (400 MHz, $CDCl_3$) δ 6.45 (1H, dd, J = 15.6, 10.7 Hz), 5.38 (1H, dd, J = 17.0, 2.3 Hz), 5.08 (1H, dd, J = 1.7, 1.0 Hz), 5.00 (1H, dd, J = 10.7, 2.3 Hz), 4.72 (1H, d, J = 1.7 Hz), 2.01 (3H, br s), 1.78–1.61 (12H, m). Spectral data agree with that reported in literature.[5]

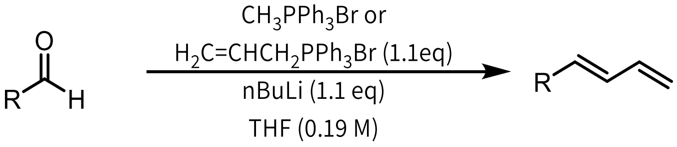

General Procedure for 1,3-diene Preparation from Aldehydes: Prepared according to a modified literature procedure.[1] Methyl triphenylphosphonium bromide or allyl triphenylphosphonium bromide (1.10 equiv.) was measured into an oven–dried round bottom flask equipped with stir bar and septum. The flask was evacuated and refilled with argon three times before the addition of dry THF (0.20 M). *n*–Butyllithium (1.10 equiv.) was added dropwise to the suspension of methyltriphenylphosphonium bromide at –78 °C, the solution was warmed up to 0 °C and stirred for 1 h, lastly aldehyde (1.00 equiv.) added dropwise at 0 °C and the reaction mixture stirred for 2–3 hours. Upon completion, the solution was diluted with diethyl ether and solids were filtered through celite. Solvent was removed under reduced pressure; then crude

residue was purified via column chromatography on SiO_2. Purified dienes were stored at –28 °C for no longer than one week.

(*E*)-1-Phenyl-1,3-butadiene (2l)

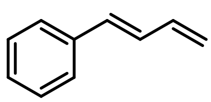

Prepared according to general procedure. Methyltriphenylphosphonium bromide (6.00 g, 16.7 mmol, 1.10 equiv.), THF (80 mL, 0.19 M), *n*–butyllithium (6.75 mL, 16.7 mmol, 1.00 equiv.), cinnamaldehyde (2.00 g, 15.1 mmol, 1.11 equiv.) were used. Crude material was purified via column chromatography on SiO_2 using 100% hexanes. The product was isolated as a colorless liquid (1.14 g, 8.80 mmol, 58%).

^{1}H NMR (400 MHz, $CDCl_3$) δ 7.42–7.40 (m, 2H), 7.34–7.31 (m, 2H), 7.25–7.22 (m, 1H), 6.81–6.76 (dd, *J* = 11.0, 11.0 Hz, 1H), 6.58 (d, *J* = 15.5 Hz, 1H), 6.53–6.49 (m, 1H), 5.34 (d, *J* = 17.2 Hz, 1H), 5.18 (d, *J* = 10.4 Hz, 1H). Spectral data agree with that reported in the literature.[1]

(*E*)-hexa-3,5-dien-1-ylbenzene (2m)

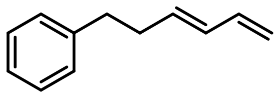

Prepared according to modified literature procedure.[1] Allyl triphenylphosphonium bromide (16.2 g, 42.3 mmol, 2.0 equiv.) was measured into an oven-dried round bottom flask equipped with a stir bar and septum. The flask was evacuated and refilled with nitrogen three times before the addition of dry THF (112 mL, 0.19 M). KO^{t}Bu (4.75 g, 42.3 mmol, 2.0 equiv.) was added the mixture was stirred at rt (20°C) for 30 min. 3-phenylpropanal (2.8 mL, 21.2 mmol, 1.0 equiv.) was added dropwise at rt and the reaction mixture was stirred for 12 hours. Upon completion, the solution was quenched with NH_4Cl (3 x 40 mL). The organic layer was extracted and washed with brine (3 X 40 mL), then dried over $MgSO_4$. Solvent was removed under reduced pressure. Crude material was purified via column chromatography on SiO_2 using 100% pentane. The product was isolated as a colorless oil (1.09 g, 6.89 mmol, 33%). Material was used immediately as it decomposes over time.

^{1}H NMR (400 MHz, $CDCl_3$) δ 7.35 – 7.13 (m, 5H), 6.68 – 6.23 (m, 1H), 6.15 – 5.97 (m, 1H), 5.82 – 5.43 (m, 1H), 5.24 – 4.94 (m, 2H), 2.76 – 2.65 (m, 2H), 2.57 – 2.35 (m, 2H). Spectral data agree with that reported in literature.[9]

Reaction Condition Optimization

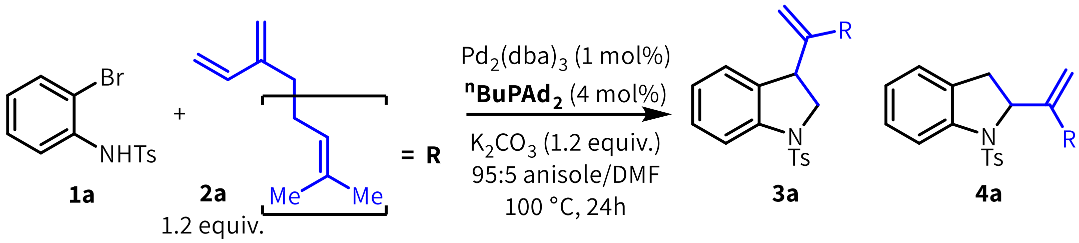

Table S1. Reaction optimization studies.

Entry	Base	Solvent	**3a** + **4a**	r.r.
		**Solvent Screen**		
1	K_2CO_3	DMF	14%	>95:5
2	K_2CO_3	50:50 toluene/DMF	20%	>95:5
3	K_2CO_3	95:5 toluene/DMF	35%	>95:5
4	K_2CO_3	anisole	14%[a]	>95:5
5	K_2CO_3	50:50 anisole/DMF	21%	>95:5
6	K_2CO_3	95:5 anisole/DMF	94%	>95:5
7	K_2CO_3	95:5 anisole/DMSO	61%	89:11
8	K_2CO_3	95:5 Butyl acetate/DMF	5%	>95:5
9	K_2CO_3	95:5 Methyl isobutyl ketone/DMF	26%	>95:5
		**Base Screen**		
10	Li_2CO_3	95:5 anisole/DMF	<3%	N/A
11	Na_2CO_3	95:5 anisole/DMF	<3%	N/A
12	Cs_2CO_3	95:5 anisole/DMF	96%	>95:5
13	KOAc	95:5 anisole/DMF	<3%	N/A
14	Et_3N	95:5 anisole/DMF	<3%	N/A
		**Reaction Tolerance** (K_2CO_3, 95:5 anisole/DMF)		
15		under air	23%	59:41
16		H_2O (1.0 equiv.) added	86%	>95:5
17		H_2O (5.0 equiv.) added	54%[a]	86:14
		**Additional Experiments** (K_2CO_3, 95:5 anisole/DMF)		
18		80 °C	20%	>95:5
19		1 equiv, TBACl	<5%	N/A
20		1 equiv, TBABr	<5%	N/A
21		1 equiv, TBAI	<5%	N/A
22		$AgNO_3$	23%	14:86

[a] Solubility issues

General procedure for reaction condition optimization studies: Bromoaniline **1a** (163 mg, 0.500 mmol, 1.0 equiv.), myrcene **2a** (103 µL, 0.600 mmol, 1.2 equiv.), di(1-adamantyl)-*n*-butylphosphine (7.2 mg, 0.020 mmol, 0.04 equiv.), tris(dibenzylideneacetone)dipalladium(0) (4.6 mg, 0.005 mmol, 0.010 equiv.), and 1,3,5-trimethoxybenze (internal standard, 40 mg, 0.238 mmol, 0.48 equiv.) were weighed out in the above-mentioned order into a 1-dram vial equipped with a stir bar and a cap with a silicone septum. The vial was then placed under nitrogen atmosphere and charged with 1 mL of freshly degassed solvent mixture (0.5M). An aliquot for HPLC analysis was taken. Then, base (0.600 mmol, 1.2 equiv.) was added to the reaction mixture. The reaction mixture was degassed with nitrogen and the reaction was stirred at 100 °C for 24 hours. After cooling to room temperature, the yield of **3a** + **4a** and the regioisomeric ratio were determined by HPLC analysis of the crude reaction mixture.

Solvent Screen

Entry 1: General procedure for condition optimization was followed. **1a** (163 mg, 0.500 mmol, 1.0 equiv.), **2a** (103 µL, 0.600 mmol, 1.2 equiv.), K_2CO_3 (83 mg, 0.600 mmol, 1.2 equiv.), di(1-adamantyl)-*n*-butylphosphine (7.2 mg, 0.020 mmol, 0.04 equiv.), $Pd_2(dba)_3$ (4.6 mg, 0.005 mmol, 0.010 equiv.), 1,3,5-trimethoxybenze (40 mg, 0.238 mmol, 0.48 equiv.) and DMF (1.0 mL, 0.5M) were used. The yield of **3a** + **4a** and the regioisomeric ratio were determined by HPLC analysis of the crude reaction mixture.

Run 1: 14%
Run 2: 13%
Average: 14% yield (>95:5 r.r.)

Entry 2: General procedure for condition optimization was followed. **1a** (163 mg, 0.500 mmol, 1.0 equiv.), **2a** (103 µL, 0.600 mmol, 1.2 equiv.), K_2CO_3 (83 mg, 0.600 mmol, 1.2 equiv.), di(1-adamantyl)-*n*-butylphosphine (7.2 mg, 0.020 mmol, 0.04 equiv.), $Pd_2(dba)_3$ (4.6 mg, 0.005 mmol, 0.010 equiv.), 1,3,5-trimethoxybenze (40 mg, 0.238 mmol, 0.48 equiv.) and 50:50 toluene/DMF (1.0 mL, 0.5M) were used. The yield of **3a** + **4a** and the regioisomeric ratio were determined by HPLC analysis of the crude reaction mixture.

Run 1: 19%
Run 2: 20%
Average: 20% yield (>95:5 r.r.)

Entry 3: General procedure for condition optimization was followed. **1a** (163 mg, 0.500 mmol, 1.0 equiv.), **2a** (103 µL, 0.600 mmol, 1.2 equiv.), K_2CO_3 (83 mg, 0.600 mmol, 1.2 equiv.), di(1-adamantyl)-*n*-butylphosphine (7.2 mg, 0.020 mmol, 0.04 equiv.), $Pd_2(dba)_3$ (4.6 mg, 0.005 mmol, 0.010 equiv.), 1,3,5-trimethoxybenze (40 mg, 0.238 mmol, 0.48 equiv.) and 95:5 toluene/DMF (1.0 mL, 0.5M) were used. The yield of **3a** + **4a** and the regioisomeric ratio were determined by HPLC analysis of the crude reaction mixture.

Run 1: 33%
Run 2: 37%
Average: 35% yield (>95:5 r.r.)

Entry 4: General procedure for condition optimization was followed. **1a** (163 mg, 0.500 mmol, 1.0 equiv.), **2a** (103 µL, 0.600 mmol, 1.2 equiv.), K_2CO_3 (83 mg, 0.600 mmol, 1.2 equiv.), di(1-adamantyl)-*n*-butylphosphine (7.2 mg, 0.020 mmol, 0.04 equiv.), $Pd_2(dba)_3$ (4.6 mg, 0.005 mmol, 0.010 equiv.), 1,3,5-trimethoxybenze (40 mg, 0.238 mmol, 0.48 equiv.) and anisole (1.0 mL, 0.5M) were used. The yield of **3a** + **4a** and the regioisomeric ratio were determined by HPLC analysis of the crude reaction mixture.

Run 1: 4%
Run 2: 10%
Run 3: 27%
Average: 14% yield (>95:5 r.r.) Solubility issues.

Entry 5: General procedure for condition optimization was followed. **1a** (163 mg, 0.500 mmol, 1.0 equiv.), **2a** (103 μL, 0.600 mmol, 1.2 equiv.), K_2CO_3 (83 mg, 0.600 mmol, 1.2 equiv.), di(1-adamantyl)-*n*-butylphosphine (7.2 mg, 0.020 mmol, 0.04 equiv.), $Pd_2(dba)_3$ (4.6 mg, 0.005 mmol, 0.010 equiv.), 1,3,5-trimethoxybenze (40 mg, 0.238 mmol, 0.48 equiv.) and 50:50 anisole/DMF (1.0 mL, 0.5M) were used. The yield of **3a** + **4a** and the regioisomeric ratio were determined by HPLC analysis of the crude reaction mixture.

Run 1: 23%
Run 2: 19%
Average: 21% yield (>95:5 r.r.)

Entry 6: General procedure for condition optimization was followed. **1a** (163 mg, 0.500 mmol, 1.0 equiv.), **2a** (103 μL, 0.600 mmol, 1.2 equiv.), K_2CO_3 (83 mg, 0.600 mmol, 1.2 equiv.), di(1-adamantyl)-*n*-butylphosphine (7.2 mg, 0.020 mmol, 0.04 equiv.), $Pd_2(dba)_3$ (4.6 mg, 0.005 mmol, 0.010 equiv.), 1,3,5-trimethoxybenze (40 mg, 0.238 mmol, 0.48 equiv.) and 95:5 anisole/DMF (1.0 mL, 0.5M) were used. The yield of **3a** + **4a** and the regioisomeric ratio were determined by HPLC analysis of the crude reaction mixture.

Run 1: 91%
Run 2: 96%
Run 3: 94%

Average: 94% yield (>95:5 r.r.)

Entry 7: General procedure for condition optimization was followed. **1a** (163 mg, 0.500 mmol, 1.0 equiv.), **2a** (103 μL, 0.600 mmol, 1.2 equiv.), K_2CO_3 (83 mg, 0.600 mmol, 1.2 equiv.), di(1-adamantyl)-*n*-butylphosphine (7.2 mg, 0.020 mmol, 0.04 equiv.), $Pd_2(dba)_3$ (4.6 mg, 0.005 mmol, 0.010 equiv.), 1,3,5-trimethoxybenze (40 mg, 0.238 mmol, 0.48 equiv.) and 95:5 anisole/DMSO (1.0 mL, 0.5M) were used. The yield of **3a** + **4a** and the regioisomeric ratio were determined by HPLC analysis of the crude reaction mixture.

Run 1: 60%
Run 2: 62%
Average: 61% yield (89:11 r.r.)

Entry 8: General procedure for condition optimization was followed. **1a** (163 mg, 0.500 mmol, 1.0 equiv.), **2a** (103 μL, 0.600 mmol, 1.2 equiv.), K_2CO_3 (83 mg, 0.600 mmol, 1.2 equiv.), di(1-adamantyl)-*n*-butylphosphine (7.2 mg, 0.020 mmol, 0.04 equiv.), $Pd_2(dba)_3$ (4.6 mg, 0.005 mmol, 0.010 equiv.), 1,3,5-trimethoxybenze (40 mg, 0.238 mmol, 0.48 equiv.) and 95:5 n-butyl acetate/DMF (1.0 mL, 0.5M) were used. The yield of **3a** + **4a** and the regioisomeric ratio were determined by HPLC analysis of the crude reaction mixture.

Run 1: 5%
Run 2: 5%
Average: 5% yield (>95:5 r.r.)

Entry 9: General procedure for condition optimization was followed. **1a** (163 mg, 0.500 mmol, 1.0 equiv.), **2a** (103 μL, 0.600 mmol, 1.2 equiv.), K_2CO_3 (83 mg, 0.600 mmol, 1.2 equiv.), di(1-

adamantyl)-*n*-butylphosphine (7.2 mg, 0.020 mmol, 0.04 equiv.), $Pd_2(dba)_3$ (4.6 mg, 0.005 mmol, 0.010 equiv.), 1,3,5-trimethoxybenze (40 mg, 0.238 mmol, 0.48 equiv.) and 95:5 methyl isobutyl ketone/DMF (1.0 mL, 0.5M) were used. The yield of **3a** + **4a** and the regioisomeric ratio were determined by HPLC analysis of the crude reaction mixture.

Run 1: 23%
Run 2: 28%
Average: 5% yield (>95:5 r.r.)

Base Screen

Entry 10: General procedure for condition optimization was followed. **1a** (163 mg, 0.500 mmol, 1.0 equiv.), **2a** (103 µL, 0.600 mmol, 1.2 equiv.), Li_2CO_3 (44 mg, 0.600 mmol, 1.2 equiv.), di(1-adamantyl)-*n*-butylphosphine (7.2 mg, 0.020 mmol, 0.04 equiv.), $Pd_2(dba)_3$ (4.6 mg, 0.005 mmol, 0.010 equiv.), 1,3,5-trimethoxybenze (40 mg, 0.238 mmol, 0.48 equiv.) and 95:5 anisole/DMF (1.0 mL, 0.5M) were used. The yield of **3a** + **4a** and the regioisomeric ratio were determined by HPLC analysis of the crude reaction mixture.

Run 1: <3%
Run 2: <3%

Average: <3% yield

Entry 11: General procedure for condition optimization was followed. **1a** (163 mg, 0.500 mmol, 1.0 equiv.), **2a** (103 µL, 0.600 mmol, 1.2 equiv.), Na_2CO_3 (64 mg, 0.600 mmol, 1.2 equiv.), di(1-adamantyl)-*n*-butylphosphine (7.2 mg, 0.020 mmol, 0.04 equiv.), $Pd_2(dba)_3$ (4.6 mg, 0.005 mmol, 0.010 equiv.), 1,3,5-trimethoxybenze (40 mg, 0.238 mmol, 0.48 equiv.) and 95:5 anisole/DMF (1.0 mL, 0.5M) were used. The yield of **3a** + **4a** and the regioisomeric ratio were determined by HPLC analysis of the crude reaction mixture.

Run 1: <3%
Run 2: <3%

Average: <3% yield

Entry 12: General procedure for condition optimization was followed. **1a** (163 mg, 0.500 mmol, 1.0 equiv.), **2a** (103 µL, 0.600 mmol, 1.2 equiv.), Cs_2CO_3 (195 mg, 0.600 mmol, 1.2 equiv.), di(1-adamantyl)-n-butylphosphine (7.2 mg, 0.020 mmol, 0.04 equiv.), $Pd_2(dba)_3$ (4.6 mg, 0.005 mmol, 0.010 equiv.), 1,3,5-trimethoxybenze (40 mg, 0.238 mmol, 0.48 equiv.) and 95:5 anisole/DMF (1.0 mL, 0.5M) were used. The yield of **3a** + **4a** and the regioisomeric ratio were determined by HPLC analysis of the crude reaction mixture.

Run 1: 98%
Run 2: 94%

Average: 96% yield (>95:5 r.r.)

Entry 13: General procedure for condition optimization was followed. **1a** (163 mg, 0.500 mmol, 1.0 equiv.), **2a** (103 µL, 0.600 mmol, 1.2 equiv.), KOAc (59 mg, 0.600 mmol, 1.2 equiv.), di(1-adamantyl)-*n*-butylphosphine (7.2 mg, 0.020 mmol, 0.04 equiv.), $Pd_2(dba)_3$ (4.6 mg, 0.005 mmol, 0.010 equiv.), 1,3,5-trimethoxybenze (40 mg, 0.238 mmol, 0.48 equiv.) and 95:5 anisole/DMF (1.0 mL, 0.5M) were used. The yield of **3a** + **4a** and the regioisomeric ratio were determined by HPLC analysis of the crude reaction mixture.

Run 1: <3%
Run 2: <3%

Average: <3% yield

Entry 14: General procedure for condition optimization was followed. **1a** (163 mg, 0.500 mmol, 1.0 equiv.), **2a** (103 µL, 0.600 mmol, 1.2 equiv.), Et_3N (84 uL, 0.600 mmol, 1.2 equiv.), di(1-adamantyl)-*n*-butylphosphine (7.2 mg, 0.020 mmol, 0.04 equiv.), $Pd_2(dba)_3$ (4.6 mg, 0.005 mmol, 0.010 equiv.), 1,3,5-trimethoxybenze (40 mg, 0.238 mmol, 0.48 equiv.) and 95:5 anisole/DMF (1.0 mL, 0.5M) were used. The yield of **3a** + **4a** and the regioisomeric ratio were determined by HPLC analysis of the crude reaction mixture.

Run 1: <3%
Run 2: <3%

Average: <3% yield

Reaction Tolerance

Entry 15: General procedure for condition optimization was followed. **1a** (163 mg, 0.500 mmol, 1.0 equiv.), **2a** (103 µL, 0.600 mmol, 1.2 equiv.), K_2CO_3 (83 mg, 0.600 mmol, 1.2 equiv.), di(1-adamantyl)-*n*-butylphosphine (7.2 mg, 0.020 mmol, 0.04 equiv.), $Pd_2(dba)_3$ (4.6 mg, 0.005 mmol, 0.010 equiv.), 1,3,5-trimethoxybenze (40 mg, 0.238 mmol, 0.48 equiv.) and non-degassed 95:5 anisole/DMF (1.0 mL, 0.5M) were used. The yield of **3a** + **4a** and the regioisomeric ratio were determined by HPLC analysis of the crude reaction mixture.

Run 1: 20%
Run 2: 26%
Average: 23% yield (59:41 r.r.)

Entry 16: General procedure for condition optimization was followed. **1a** (163 mg, 0.500 mmol, 1.0 equiv.), **2a** (103 µL, 0.600 mmol, 1.2 equiv.), K_2CO_3 (83 mg, 0.600 mmol, 1.2 equiv.), di(1-adamantyl)-*n*-butylphosphine (7.2 mg, 0.020 mmol, 0.04 equiv.), $Pd_2(dba)_3$ (4.6 mg, 0.005 mmol, 0.010 equiv.), 1,3,5-trimethoxybenze (40 mg, 0.238 mmol, 0.48 equiv.) and non-degassed 95:5 anisole/DMF (1.0 mL, 0.5M) were used. H_2O (9.0 µL, 0.500 mmol, 1.0 equiv.) was added to the reaction mixture before heating it up to 100 °C. The yield of **3a** + **4a** and the regioisomeric ratio were determined by HPLC analysis of the crude reaction mixture.

Run 1: 85%
Run 2: 87%
Average: 86% yield (>95:5 r.r.)

Entry 17: General procedure for condition optimization was followed. **1a** (163 mg, 0.500 mmol, 1.0 equiv.), **2a** (103 µL, 0.600 mmol, 1.2 equiv.), K_2CO_3 (83 mg, 0.600 mmol, 1.2 equiv.), di(1-adamantyl)-*n*-butylphosphine (7.2 mg, 0.020 mmol, 0.04 equiv.), $Pd_2(dba)_3$ (4.6 mg, 0.005 mmol, 0.010 equiv.), 1,3,5-trimethoxybenze (40 mg, 0.238 mmol, 0.48 equiv.) and non-degassed 95:5 anisole/DMF (1.0 mL, 0.5M) were used. H_2O (45 µL, 2.500 mmol, 5.0 equiv.) was added to the reaction mixture before heating it up to 100 °C. The yield of **3a** + **4a** and the regioisomeric ratio were determined by HPLC analysis of the crude reaction mixture.

Run 1: 49%
Run 2: 58%
Average: 54% yield (86:14 r.r.) solubility issues

Additional Experiments

Entry 18: General procedure for condition optimization was followed. **1a** (163 mg, 0.500 mmol, 1.0 equiv.), **2a** (103 μL, 0.600 mmol, 1.2 equiv.), K_2CO_3 (83 mg, 0.600 mmol, 1.2 equiv.), di(1-adamantyl)-*n*-butylphosphine (7.2 mg, 0.020 mmol, 0.04 equiv.), $Pd_2(dba)_3$ (4.6 mg, 0.005 mmol, 0.010 equiv.), 1,3,5-trimethoxybenze (40 mg, 0.238 mmol, 0.48 equiv.) and 95:5 anisole/DMF (1.0 mL, 0.5M) were used. The reaction was stirred at 80 °C for 24 hours. The yield of **3a** + **4a** and the regioisomeric ratio were determined by HPLC analysis of the crude reaction mixture.

Run 1: 21%
Run 2: 19%
Average: 20% yield (>95:5 r.r.)

Entry 19: General procedure for condition optimization was followed. **1a** (163 mg, 0.500 mmol, 1.0 equiv.), **2a** (103 μL, 0.600 mmol, 1.2 equiv.), K_2CO_3 (83 mg, 0.600 mmol, 1.2 equiv.), di(1-adamantyl)-*n*-butylphosphine (7.2 mg, 0.020 mmol, 0.04 equiv.), $Pd_2(dba)_3$ (4.6 mg, 0.005 mmol, 0.010 equiv.), 1,3,5-trimethoxybenze (40 mg, 0.238 mmol, 0.48 equiv.), TBACl (139 mg, 0.500 mmol, 1.0 equiv.) and 95:5 anisole/DMF (1.0 mL, 0.5M) were used. The reaction was stirred at 100 °C for 24 hours. The yield of **3a** + **4a** and the regioisomeric ratio were determined by HPLC analysis of the crude reaction mixture.

Run 1: <5%
Run 2: <5%
Average: <5% yield

Entry 20: General procedure for condition optimization was followed. **1a** (163 mg, 0.500 mmol, 1.0 equiv.), **2a** (103 μL, 0.600 mmol, 1.2 equiv.), K_2CO_3 (83 mg, 0.600 mmol, 1.2 equiv.), di(1-adamantyl)-*n*-butylphosphine (7.2 mg, 0.020 mmol, 0.04 equiv.), $Pd_2(dba)_3$ (4.6 mg, 0.005 mmol, 0.010 equiv.), 1,3,5-trimethoxybenze (40 mg, 0.238 mmol, 0.48 equiv.), TBABr (161 mg, 0.500 mmol, 1.0 equiv.) and 95:5 anisole/DMF (1.0 mL, 0.5M) were used. The reaction was stirred at 100 °C for 24 hours. The yield of **3a** + **4a** and the regioisomeric ratio were determined by HPLC analysis of the crude reaction mixture.

Run 1: <5%
Run 2: <5%
Average: <5% yield

Entry 21: General procedure for condition optimization was followed. **1a** (163 mg, 0.500 mmol, 1.0 equiv.), **2a** (103 μL, 0.600 mmol, 1.2 equiv.), K_2CO_3 (83 mg, 0.600 mmol, 1.2 equiv.), di(1-adamantyl)-*n*-butylphosphine (7.2 mg, 0.020 mmol, 0.04 equiv.), $Pd_2(dba)_3$ (4.6 mg, 0.005 mmol, 0.010 equiv.), 1,3,5-trimethoxybenze (40 mg, 0.238 mmol, 0.48 equiv.), TBAI (185 mg, 0.500 mmol, 1.0 equiv.) and 95:5 anisole/DMF (1.0 mL, 0.5M) were used. The reaction was stirred at 100 °C for 24 hours. The yield of **3a** + **4a** and the regioisomeric ratio were determined by HPLC analysis of the crude reaction mixture.

Run 1: <5%
Run 2: <5%
Average: <5% yield

Entry 22: General procedure for condition optimization was followed. **1a** (163 mg, 0.500 mmol, 1.0 equiv.), **2a** (103 μL, 0.600 mmol, 1.2 equiv.), K_2CO_3 (83 mg, 0.600 mmol, 1.2 equiv.), di(1-adamantyl)-*n*-butylphosphine (7.2 mg, 0.020 mmol, 0.04 equiv.), $Pd_2(dba)_3$ (4.6 mg, 0.005 mmol, 0.010 equiv.), 1,3,5-trimethoxybenze (40 mg, 0.238 mmol, 0.48 equiv.), $AgNO_3$ (85 mg, 0.500

mmol, 1.0 equiv.) and 95:5 anisole/DMF (1.0 mL, 0.5M) were used. The reaction was stirred at 100 °C for 24 hours. The yield of **3a** + **4a** and the regioisomeric ratio were determined by HPLC analysis of the crude reaction mixture.

Run 1: 22%
Run 2: 24%
Average: 23% yield (86:14 r.r.)

Ligand Structure-selectivity Relationship Studies

General Procedure Bromoaniline **1a** (163 mg, 0.500 mmol, 1.0 equiv.), myrcene **2a** (103 µL, 0.600 mmol, 1.2 equiv.), ligand (0.020 mmol, 0.04 equiv.), potassium carbonate (83 mg, 0.600 mmol, 1.2 equiv.), and tris(dibenzylideneacetone)dipalladium(0) (4.6 mg, 0.005 mmol, 0.010 equiv.) were weighed out in the above-mentioned order into a 1-dram vial equipped with a stir bar and a cap with a silicone septum. The vial was then placed under nitrogen atmosphere and charged with 1 mL of freshly degassed anisole/dimethylformamide (95:5) solvent mixture. The reaction was stirred at 100 °C for 24 hours. After cooling to room temperature, the yield of 3a + 4a and the regioisomeric ratio were determined by HPLC analysis of the crude reaction mixture.

Table S2. Phosphine screening results.

Label	Individual run yields (%)						Triplicates average yield (%)	Selectivity for **3a**
	Run 1		Run 2		Run 3			
	**3a**	**4a**	**3a**	**4a**	**3a**	**4a**		
$PAd_2{}^nBu$	88.9	2.4	93.6	2.9	90.9	2.8	93.8	97.1
P^tBu_2Me	10.7	1.1	9.6	0.9	9.3	0.9	10.8	91.1
PCy_3	2.3	0.2	2.5	0.5	1.6	0.4	2.5	85.3
RuPhos	12.0	2.8	8.0	2.2	8.8	2.0	11.9	80.4
Xphos	4.1	2.3	3.2	1.7	4.3	2.2	5.9	65.2
VPhos	6.2	8.0	13.5	7.6	13.5	7.6	18.8	58.9
$PCy_2(4NMe_2Ph)$	4.3	2.2	1.7	3.3	1.6	2.9	5.3	47.5
PCy_2oTol	0.7	1.2	0.9	1.4	0.7	0.4	1.8	43.4
$PPhCy_2$	1.3	2.0	0.9	1.1	1.2	1.8	2.8	41.0
Sphos	7.0	10.7	7.6	12.0	7.7	11.8	18.9	39.3
CyTyrranoPhos	2.2	3.8	1.1	1.9	1.3	2.4	4.2	36.2
$PtBuiPr_2$	6.5	17.0	6.3	16.3	3.7	19.5	23.1	23.8
PPh_3	0.4	0.7	0.1	0.8	0.1	0.9	1.0	19.9
$P(p\text{-}OMePh)_3$	0.7	2.0	0.2	1.4	0.2	1.5	2.0	18.3
CPhos	2.7	14.4	1.7	8.3	2.0	11.6	13.6	15.7
$PPh_2(2\text{-}OMe\text{-}Ph)$	0.7	4.3	0.9	4.7	1.4	7.4	6.5	15.5
MePhos	2.3	15.4	2.2	12.2	2.1	11.4	15.2	14.5
PPh_2Cy	2.1	12.6	1.7	10.0	1.6	9.7	12.6	14.3
mono-methoxySPhos	2.8	17.4	3.2	19.4	3.9	22.8	23.2	14.2
CataCXium P^tBu	1.5	11.5	1.3	10.2	1.7	6.2	10.8	13.9
$P(OBn)_2NiPr_2$	1.7	10.0	1.6	9.2	1.8	12.6	12.3	13.8
Cy-vBRIDP	1.3	9.7	2.0	12.2	1.4	8.2	11.6	13.5
P^tBuPh_2	1.7	12.0	1.9	12.5	2.5	15.0	15.2	13.4
$PoTol_3$	1.3	9.1	1.2	7.4	1.3	8.3	9.5	13.3
$P(OiPr)_3$	2.9	22.5	2.4	16.9	3.5	22.3	23.5	12.5
$PnOct_3$	1.3	8.0	1.1	8.3	1.1	8.5	9.4	12.4
PPh_2iPr	1.4	10.6	2.3	15.3	1.9	13.8	15.1	12.4
CyJohnPhos	2.1	14.1	2.3	17.4	2.1	15.0	17.7	12.3

$P(OtBu)_2NiPr_2$	2.0	15.4	2.1	14.4	2.6	17.9	18.1	12.3
^{t}BuXPhos	0.4	3.7	0.7	4.5	0.4	3.3	4.3	11.5
CataCXium PCy	2.3	17.4	1.9	16.3	1.7	13.6	17.7	11.1
$PPhMe_2$	2.9	23.7	2.4	18.8	2.2	18.8	22.9	10.9
PPh_2Me	1.0	8.9	0.9	8.3	0.8	4.9	8.3	10.9
$P(OPh)_3$	2.5	20.3	1.8	15.3	1.9	16.6	19.5	10.6
AdBippyphos	5.7	40.4	4.9	40.3	4.1	43.1	46.2	10.6
$P(5\text{-}F\text{-}Ph)_2Ph$	1.5	12.3	1.0	9.6	1.8	14.3	13.5	10.6
$P(pCF_3Ph)_3$	1.8	17.6	1.9	17.1	1.5	14.4	18.1	9.6
$PtBu_2$neopentyl	0.2	2.6	0.3	4.4	0.4	4.9	4.3	7.0
JohnPhos	1.4	20.2	1.3	19.1	0.9	17.0	20.0	6.0
PAd_3	1.0	14.4	1.1	16.9	1.2	19.7	18.1	6.1
$PtBu_3$	1.4	28.4	1.3	27.0	1.3	26.5	28.6	4.7

Reaction Scope

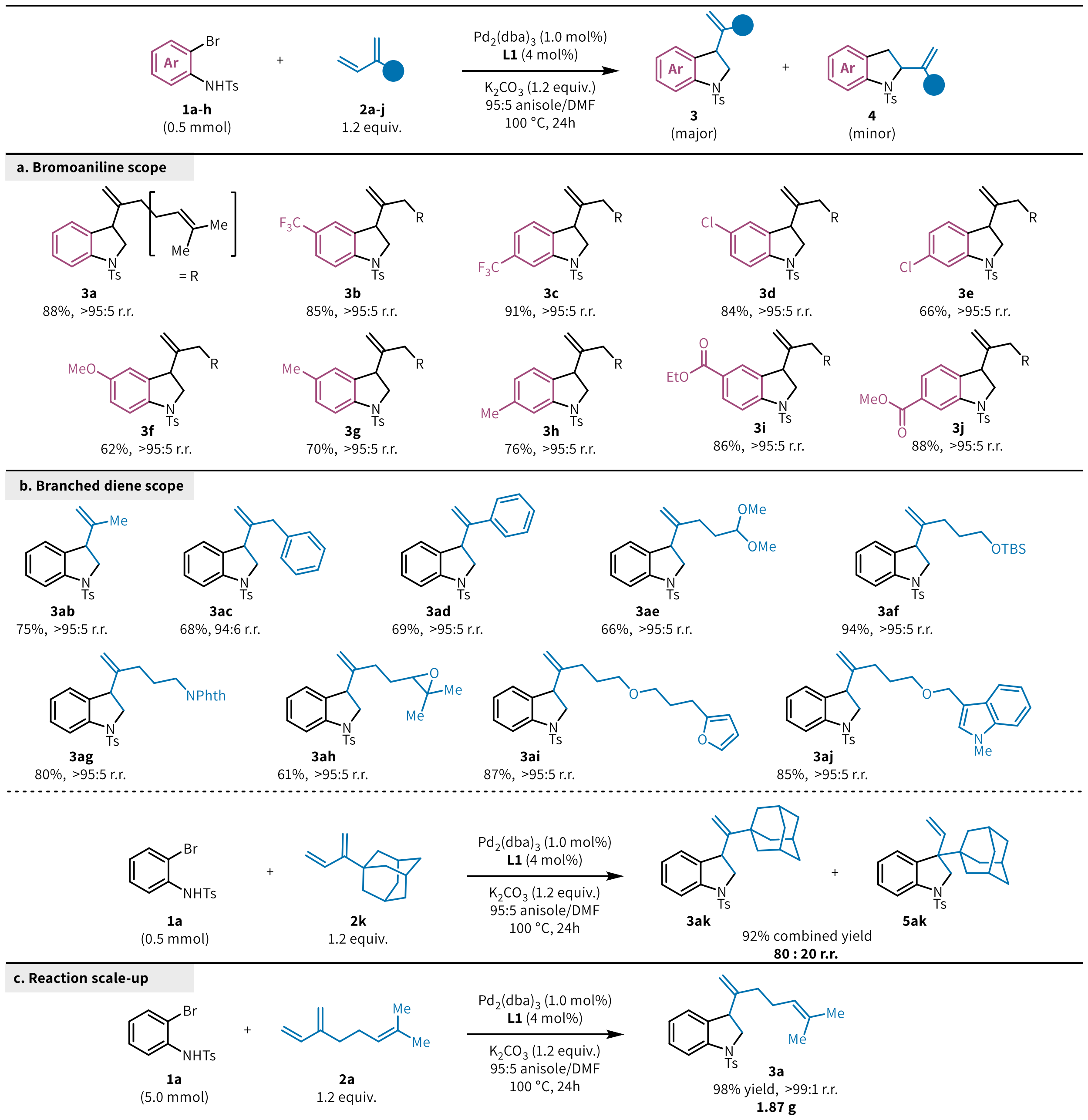

Figure S1. Substrate scope of ligand-controlled heteroannulation reaction.

General heteroannulation procedure: Bromoaniline **1a** (163 mg, 0.500 mmol, 1.0 equiv.), myrcene **2a** (103 μL, 0.600 mmol, 1.2 equiv.), di(1-adamantyl)-*n*-butylphosphine (7.2 mg, 0.020 mmol, 0.04 equiv.), potassium carbonate (83 mg, 0.600 mmol, 1.2 equiv.), and tris(dibenzylideneacetone)dipalladium(0) (4.6 mg, 0.005 mmol, 0.010 equiv.) were weighed out in the above-mentioned order into a 1-dram vial equipped with a stir bar and a cap with a silicone septum. The vial was then placed under nitrogen atmosphere and charged with 1 mL of freshly degassed anisole/dimethylformamide (95:5) solvent mixture. The reaction was stirred at 100 °C

for 24 hours. After cooling to room temperature, regioisomeric ratios were determined by HPLC analysis of the crude reaction mixture and the reaction mixture was filtered with ethyl acetate through cotton plug. The solvents were removed under reduced pressure and the crude mixture was purified by flash column chromatography to obtain products **3**.

Bromoaniline Scope

3-(6-methylhepta-1,5-dien-2-yl)-*N*-tosylindoline (3a)

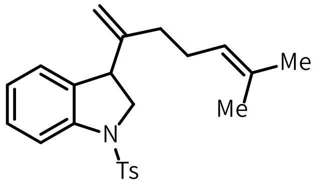

Prepared according to the general procedure. Bromoaniline **1a** (163 mg, 0.500 mmol, 1.0 equiv.), myrcene **2a** (103 µL, 0.600 mmol, 1.2 equiv.), K_2CO_3 (83 mg, 0.600 mmol, 1.2 equiv.), di(1-adamantyl)-*n*-butylphosphine (7.2 mg, 0.020 mmol, 0.04 equiv.), $Pd_2(dba)_3$ (4.6 mg, 0.005 mmol, 0.01 equiv.), and 95:5 anisole/DMF (1 mL, 0.5M) were used. The crude material was purified by flash column chromatography (silica, hexanes/ethyl acetate 19:1) to obtain products **3a** and **4a** (>95:5 r.r.) as pale yellow oil.

Run 1: (167 mg, 0.435 mmol, 87%)
Run 2: (169 mg, 0.440 mmol, 88%)
Run 3: (171 mg, 0.450 mmol, 90%)
Average yield: 88%

^{1}H NMR (500 MHz, $CDCl_3$) δ 7.70–7.67 (m, 3H), 7.24–7.19 (m, 3H), 6.99-6.96 (m, 2H), 4.95–4.91 (m, 1H), 4.77 (d, *J* = 1.5 Hz, 1H), 4.65 (s, 1H), 4.07 (dd, *J* = 10.7, 10.0 Hz, 1H), 3.83 (dd, *J* = 10.0, 6.6 Hz, 1H), 3.68 (dd, *J* = 10.7, 6.6 Hz, 1H), 2.36 (s, 3H), 2.04–1.93 (m, 2H), 1.81–1.69 (m, 2H), 1.66 (s, 3H), 1.53 (s, 3H).

^{13}C NMR (125 MHz, $CDCl_3$) δ 148.6, 144.2, 142.4, 134.0, 133.5, 132.0, 129.7, 128.3, 127.5, 125.6, 123.8, 123.7, 114.8, 112.4, 55.2, 47.6, 32.7, 26.4, 25.8, 21.6, 17.8.

HRMS (ESI) *m/z* calculated for $C_{23}H_{28}NO_2S$ $[M+H]^+$: 382.1841, found 382.1833.

3-(6-methylhepta-1,5-dien-2-yl)-*N*-tosyl-5-(trifluoromethyl)indoline (3b)

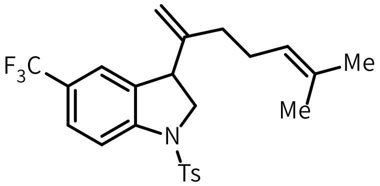

Prepared according to the general procedure. Bromoaniline **1b** (197 mg, 0.500 mmol, 1.0 equiv.), **2a** (103 µL, 0.600 mmol, 1.2 equiv.), K_2CO_3 (83 mg, 0.600 mmol, 1.2 equiv.), di(1-adamantyl)-*n*-butylphosphine (7.2 mg, 0.020 mmol, 0.04 equiv.), $Pd_2(dba)_3$ (4.6 mg, 0.005 mmol, 0.01 equiv.), and 95:5 anisole/DMF (1 mL, 0.5M) were used. The crude material was purified by flash column chromatography (silica, hexanes/ethyl acetate 19:1) to obtain products **3b** and **4b** (>95:5 r.r.) as pale yellow oil.

Run 1: (198 mg, 0.440 mmol, 88%)
Run 2: (186 mg, 0.415 mmol, 83%)
Run 3: (187 mg, 0.415 mmol, 83%)
Average yield: 85%

^{1}H NMR (500 MHz, $CDCl_3$) δ 7.73 (d, *J* = 8.6 Hz, 1H), 7.70 (d, *J* = 8.2 Hz, 2H), 7.48 (d, *J* = 8.6 Hz, 1H), 7.26 (d, *J* = 8.2 Hz, 2H), 7.20 (s, 1H), 4.92 (t, *J* = 6.9 Hz, 1H), 4.83 (s, 1H), 4.69 (s, 1H),

4.13 (dd, J = 10.5, 10.3 Hz, 1H), 3.90 (dd, J = 10.3, 6.5 Hz, 1H), 3.74 (dd, J = 10.5, 6.5 Hz, 1H), 2.39 (s, 3H), 2.03–1.96 (m, 2H), 1.80–1.68 (m, 2H), 1.66 (s, 3H), 1.52 (s, 3H).

^{13}C NMR (125 MHz, $CDCl_3$) δ 147.8, 145.4, 144.8, 134.1, 133.9, 132.4, 130.0, 127.4, 126.1 (q, J_{C-F} = 3.8 Hz), 125.8 (q, J_{C-F} = 32.5 Hz), 124.3 (q, J_{C-F} = 270.0 Hz), 123.4, 122.8 (q, J_{C-F} = 3.8 Hz), 114.1, 113.3, 55.5, 47.3, 32.7, 26.4, 25.8, 21.7, 17.8.

^{19}F NMR (376 MHz, $CDCl_3$) δ -61.5 (3F).

HRMS (ESI) *m/z* calculated for $C_{24}H_{27}F_3NO_2S$ $[M+H]^+$: 450.1715, found 450.1709.

3-(6-methylhepta-1,5-dien-2-yl)-*N*-tosyl-6-(trifluoromethyl)indoline (3c)

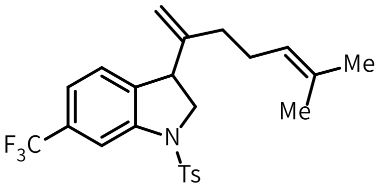

Prepared according to the general procedure. Bromoaniline **1c** (197 mg, 0.500 mmol, 1.0 equiv.), **2a** (103 μL, 0.600 mmol, 1.2 equiv.), K_2CO_3 (83 mg, 0.600 mmol, 1.2 equiv.), di(1-adamantyl)-*n*-butylphosphine (7.2 mg, 0.020 mmol, 0.04 equiv.), $Pd_2(dba)_3$ (4.6 mg, 0.005 mmol, 0.01 equiv.), and 95:5 anisole/DMF (1 mL, 0.5M) were used. The crude material was purified by flash column chromatography (silica, hexanes/ethyl acetate 19:1) to obtain products **3c** and **4c** (>95:5 r.r.) as pale yellow oil.

Run 1: (208 mg, 0.460 mmol, 92%)
Run 2: (211 mg, 0.470 mmol, 94%)
Run 3: (199 mg, 0.445 mmol, 88%)
Average yield: 91%

^{1}H NMR (500 MHz, $CDCl_3$) δ 7.90 (s, 1H), 7.68 (d, J = 8.5 Hz, 2H), 7.25 (d, J = 8.5 Hz, 2H), 7.23 (d, J = 7.8 Hz, 1H), 7.06 (d, J = 7.8 Hz, 1H), 4.92 (t, J = 7.0 Hz, 1H), 4.82 (s, 1H), 4.67 (s, 1H), 4.13 (dd, J = 10.6, 10.2 Hz, 1H), 3.89 (dd, J = 10.2, 6.2 Hz, 1H), 3.73 (dd, J = 10.6, 6.6 Hz, 1H), 2.38 (s, 3H), 2.06–1.93 (m, 2H), 1.81–1.68 (m, 2H), 1.66 (s, 3H), 1.53 (s, 3H).

^{13}C NMR (125 MHz, $CDCl_3$) δ 147.9, 144.7, 143.0, 137.4, 133.7, 132.3, 131.0 (q, J_{C-F} = 32.5 Hz), 130.0, 127.5, 125.9, 124.1 (q, J_{C-F} = 271.3 Hz), 123.5, 120.7 (q, J_{C-F} = 3.8 Hz), 113.2, 111.5 (q, J_{C-F} = 3.8 Hz), 55.4, 47.4, 32.8, 26.4, 25.8, 21.7, 17.8.

^{19}F NMR (376 MHz, $CDCl_3$) δ -61.6 (3F).

HRMS (ESI) *m/z* calculated for $C_{24}H_{27}F_3NO_2S$ $[M+H]^+$: 450.1715, found 450.1709.

5-chloro-3-(6-methylhepta-1,5-dien-2-yl)-*N*-tosylindoline (3d)

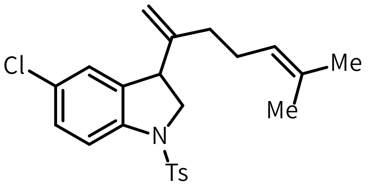

Prepared according to the general procedure. Bromoaniline **1d** (180 mg, 0.500 mmol, 1.0 equiv.), **2a** (103 μL, 0.600 mmol, 1.2 equiv.), K_2CO_3 (83 mg, 0.600 mmol, 1.2 equiv.), di(1-adamantyl)-*n*-butylphosphine (7.2 mg, 0.020 mmol, 0.04 equiv.), $Pd_2(dba)_3$ (4.6 mg, 0.005 mmol, 0.01 equiv.), and 95:5 anisole/DMF (1 mL, 0.5M) were used. The crude material was purified by flash column chromatography (silica, hexanes/ethyl acetate 19:1) to obtain products **3d** and **4d** (>95:5 r.r.) as pale yellow oil.

Run 1: (181 mg, 0.440 mmol, 87%)
Run 2: (168 mg, 0.405 mmol, 81%)
Run 3: (173 mg, 0.415 mmol, 83%)

Average yield: 84%

^{1}H NMR (500 MHz, $CDCl_3$) δ 7.66 (d, *J* = 8.3 Hz, 2H), 7.60 (d, *J* = 8.6 Hz, 1H), 7.24 (d, *J* = 8.3 Hz, 2H), 7.17 (dd, *J* = 8.6, 2.3 Hz, 1H), 6.92 (d, *J* = 2.3 Hz, 1H), 4.92 (t, *J* = 6.9 Hz, 1H), 4.80 (s, 1H), 4.65 (s, 1H), 4.07 (dd, *J* = 10.8, 9.6 Hz, 1H), 3.80 (dd, *J* = 9.6, 7.3 Hz, 1H), 3.68 (dd, *J* = 10.8, 7.3 Hz, 1H), 2.37 (s, 3H), 2.04–1.93 (m, 2H), 1.77–1.69 (m, 2H), 1.66 (s, 3H), 1.53 (s, 3H).

^{13}C NMR (125 MHz, $CDCl_3$) δ 147.8, 144.5, 141.1, 135.6, 133.6, 132.3, 129.9, 129.0, 128.4, 127.5, 125.7, 123.5, 115.9, 113.1, 55.4, 47.5, 32.6, 26.3, 25.8, 21.7, 17.8.

HRMS (ESI) *m/z* calculated for $C_{23}H_{27}ClNO_2S$ $[M+H]^+$: 416.1451, found 416.1442.

6-chloro-3-(6-methylhepta-1,5-dien-2-yl)-*N*-tosylindoline (3e)

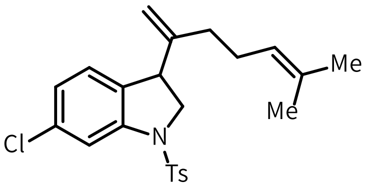

Prepared according to the general procedure. Bromoaniline **1e** (180 mg, 0.500 mmol, 1.0 equiv.), **2a** (103 μL, 0.600 mmol, 1.2 equiv.), K_2CO_3 (83 mg, 0.600 mmol, 1.2 equiv.), di(1-adamantyl)-*n*-butylphosphine (7.2 mg, 0.020 mmol, 0.04 equiv.), $Pd_2(dba)_3$ (4.6 mg, 0.005 mmol, 0.01 equiv.), and 95:5 anisole/DMF (1 mL, 0.5M) were used. The crude material was purified by flash column chromatography (silica, hexanes/ethyl acetate 19:1) to obtain products **3e** and **4e** (>95:5 r.r.) as pale yellow oil.

Run 1: (137 mg, 0.330 mmol, 66%)
Run 2: (136 mg, 0.325 mmol, 65%)
Run 3: (142 mg, 0.340 mmol, 68%)
Average yield: 66%

^{1}H NMR (500 MHz, $CDCl_3$) δ 7.72–7.68 (m, 3H), 7.26 (d, *J* = 8.3 Hz, 2H), 6.94 (dd, *J* = 8.1, 1.9 Hz, 1H), 6.87 (d, *J* = 8.0 Hz, 1H), 4.94–4.89 (m, 1H), 4.77 (s, 1H), 4.65 (s, 1H), 4.07 (dd, *J* = 10.6, 10.3 Hz, 1H), 3.81 (dd, *J* = 10.3, 6.2 Hz, 1H), 3.68 (dd, *J* = 10.6, 6.2 Hz, 1H), 2.39 (s, 3H), 2.03–1.92 (m, 2H), 1.77–1.67 (m, 2H), 1.66 (s, 3H), 1.53 (s, 3H).

^{13}C NMR (125 MHz, $CDCl_3$) δ 148.2, 144.6, 143.6, 134.1, 133.8, 132.2, 132.0, 129.9, 127.5, 126.4, 123.8, 123.6, 115.0, 112.7, 55.6, 47.1, 32.6, 26.3, 25.8, 21.7, 17.8.

HRMS (ESI) *m/z* calculated for $C_{23}H_{27}ClNO_2S$ $[M+H]^+$: 416.1451, found 416.1442.

5-methoxy-3-(6-methylhepta-1,5-dien-2-yl)-*N*-tosylindoline (3f)

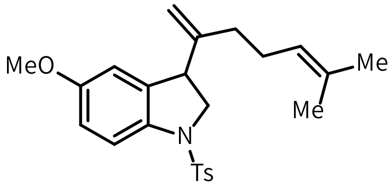

Prepared according to the general procedure. Bromoaniline **1f** (178 mg, 0.500 mmol, 1.0 equiv.), myrcene **2a** (103 μL, 0.600 mmol, 1.2 equiv.), K_2CO_3 (83 mg, 0.600 mmol, 1.2 equiv.), di(1-adamantyl)-*n*-butylphosphine (7.2 mg, 0.020 mmol, 0.04 equiv.), $Pd_2(dba)_3$ (4.6 mg, 0.005 mmol, 0.01 equiv.), and 95:5 anisole/DMF (1 mL, 0.5M) were used. The crude material was purified by flash column chromatography (silica, hexanes/ethyl acetate 19:1) to obtain products **3f** and **4f** (>95:5 r.r.) as pale yellow oil.

Run 1: (128 mg, 0.310 mmol, 62%)
Run 2: (125 mg, 0.305 mmol, 61%)
Run 3: (132 mg, 0.320 mmol, 64%)
Average yield: 62%

^{1}H NMR (500 MHz, $CDCl_3$) δ 7.60 (d, J = 8.2 Hz, 2H), 7.57 (d, J = 8.8 Hz, 1H), 7.18 (d, J = 8.2 Hz, 2H), 6.74 (dd, J = 8.8, 1.8 Hz, 1H), 6.58 (d, J = 1.8 Hz, 1H), 4.93 (t, J = 7.0 Hz, 1H), 4.74 (s, 1H), 4.62 (s, 1H), 4.03 (dd, J = 10.8, 10.6 Hz, 1H), 3.73-3.68 (m, 4H), 3.63 (dd, J = 10.8, 7.2 Hz, 1H), 2.34 (s, 3H), 2.00–1.90 (m, 2H), 1.73–1.65 (m, 2H), 1.64 (s, 3H), 1.51 (s, 3H).

^{13}C NMR (125 MHz, $CDCl_3$) δ 156.8, 148.1, 144.1, 135.9, 135.4, 133.7, 132.0, 129.7, 127.6, 123.7, 116.3, 113.5, 112.7, 111.1, 55.7, 55.5, 48.0, 32.6, 26.3, 25.8, 21.7, 17.8.

HRMS (ESI) *m/z* calculated for $C_{24}H_{30}NO_3S$ $[M+H]^+$: 412.1946, found 412.1937.

5-methyl-3-(6-methylhepta-1,5-dien-2-yl)-*N*-tosylindoline (3g)

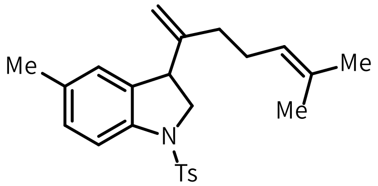

Prepared according to the general procedure. Bromoaniline **1g** (170 mg, 0.500 mmol, 1.0 equiv.), **2a** (103 μL, 0.600 mmol, 1.2 equiv.), K_2CO_3 (83 mg, 0.600 mmol, 1.2 equiv.), di(1-adamantyl)-*n*-butylphosphine (7.2 mg, 0.020 mmol, 0.04 equiv.), $Pd_2(dba)_3$ (4.6 mg, 0.005 mmol, 0.01 equiv.), and 95:5 anisole/DMF (1 mL, 0.5M) were used. The crude material was purified by flash column chromatography (silica, hexanes/ethyl acetate 19:1) to obtain products **3g** and **4g** (>95:5 r.r.) as pale yellow oil.

Run 1: (139 mg, 0.350 mmol, 70%)
Run 2: (138 mg, 0.350 mmol, 70%)
Run 3: (141 mg, 0.355 mmol, 71%)
Average yield: 70%

^{1}H NMR (500 MHz, $CDCl_3$) δ 7.66 (d, J = 8.5 Hz, 2H), 7.55 (d, J = 8.3 Hz, 1H), 7.21 (d, J = 8.5 Hz, 2H), 7.01 (d, J = 8.3 Hz, 1H), 6.76 (s, 1H), 4.94 (t, J = 6.2 Hz, 1H), 4.77 (s, 1H), 4.65 (s, 1H), 4.04 (dd, J = 10.7, 10.0 Hz, 1H), 3.77 (dd, J = 10.0, 6.4 Hz, 1H), 3.65 (dd, J = 10.7, 6.4 Hz, 1H), 2.36 (s, 3H), 2.26 (s, 3H), 2.02–1.94 (m, 1H), 1.78–1.68 (m, 2H), 1.66 (s, 3H), 1.53 (s, 3H).

^{13}C NMR (125 MHz, $CDCl_3$) δ 148.6, 144.1, 140.1, 134.0, 133.7, 133.5, 132.0, 129.7, 128.9, 127.5, 126.1, 123.8, 114.8, 112.4, 55.4, 47.7, 32.6, 26.4, 25.8, 21.7, 21.0, 17.8.

HRMS (ESI) *m/z* calculated for $C_{24}H_{30}NO_2S$ $[M+H]^+$: 396.1997, found 396.1990.

6-methyl-3-(6-methylhepta-1,5-dien-2-yl)-*N*-tosylindoline (3h)

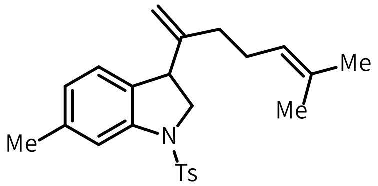

Prepared according to the general procedure. Bromoaniline **1h** (170 mg, 0.500 mmol, 1.0 equiv.), **2a** (103 μL, 0.600 mmol, 1.2 equiv.), K_2CO_3 (83 mg, 0.600 mmol, 1.2 equiv.), di(1-adamantyl)-*n*-butylphosphine (7.2 mg, 0.020 mmol, 0.04 equiv.), $Pd_2(dba)_3$ (4.6 mg, 0.005 mmol, 0.01 equiv.), and 95:5 anisole/DMF (1 mL, 0.5M) were used. The crude material was purified by flash column chromatography (silica, hexanes/ethyl acetate 19:1) to obtain products **3h** and **4h** (>95:5 r.r.) as pale yellow oil.

Run 1: (151 mg, 0.380 mmol, 76%)
Run 2: (155 mg, 0.390 mmol, 78%)
Run 3: (148 mg, 0.378 mmol, 75%)
Average yield: 76%

^{1}H NMR (500 MHz, $CDCl_3$) δ 7.66 (d, J = 8.3 Hz, 2H), 7.55 (d, J = 8.3 Hz, 1H), 7.21 (d, J = 8.3 Hz, 2H), 7.01 (d, J = 8.3 Hz, 1H), 6.76 (s, 1H), 4.98–4.89 (m, 1H), 4.76 (s, 1H), 4.65 (s, 1H), 4.04

(dd, J = 10.7, 10.0 Hz, 1H), 3.77 (dd, J = 10.0, 6.4 Hz, 1H), 3.65 (dd, J = 10.7, 6.4 Hz, 1H), 2.36 (s, 3H), 2.25 (s, 3H), 2.02–1.94 (m, 2H), 1.78–1.68 (m, 2H), 1.66 (s, 3H), 1.53 (s, 3H).

^{13}C NMR (125 MHz, $CDCl_3$) δ 148.6, 144.0, 140.1, 134.0, 133.7, 133.5, 132.0, 129.7, 128.9, 127.5, 126.1, 123.8, 114.8, 112.4, 55.4, 47.7, 32.6, 26.4, 25.8, 21.7, 21.0, 17.8.

HRMS (ESI) *m/z* calculated for $C_{24}H_{30}NO_2S$ $[M+H]^+$: 396.1997, found 396.1990.

ethyl 3-(6-methylhepta-1,5-dien-2-yl)-*N*-tosylindoline-5-carboxylate (3i)

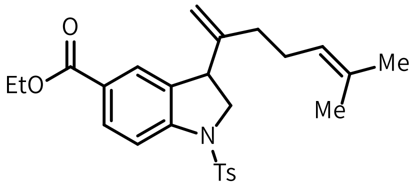

Prepared according to the general procedure. Bromoaniline **1i** (199 mg, 0.500 mmol, 1.0 equiv.), **2a** (103 μL, 0.600 mmol, 1.2 equiv.), K_2CO_3 (83 mg, 0.600 mmol, 1.2 equiv.), di(1-adamantyl)-*n*-butylphosphine (7.2 mg, 0.020 mmol, 0.04 equiv.), $Pd_2(dba)_3$ (4.6 mg, 0.005 mmol, 0.01 equiv.), and 95:5 anisole/DMF (1 mL, 0.5M) were used. The crude material was purified by flash column chromatography (silica, hexanes/ethyl acetate 19:1) to obtain products **3i** and **4i** (>95:5 r.r.) as pale yellow oil.

Run 1: (198 mg, 0.435 mmol, 87%)
Run 2: (191 mg, 0.420 mmol, 84%)
Run 3: (195 mg, 0.430 mmol, 86%)
Average yield: 86%

^{1}H NMR (500 MHz, $CDCl_3$) δ 7.94 (dd, J = 8.5, 1.5 Hz, 1H), 7.72–7.67 (m, 3H), 7.64 (d, J = 1.5 Hz, 1H), 7.24 (d, J = 8.2 Hz, 2H), 4.95–4.88 (m, 1H), 4.81 (s, 1H), 4.68 (s, 1H), 4.36–4.26 (m, 2H), 4.11 (dd, J = 10.6, 10.2 Hz, 1H), 3.89 (dd, J = 10.2, 6.5 Hz, 1H), 3.74 (dd, J = 10.6, 6.5 Hz, 1H), 2.37 (s, 3H), 2.04–1.95 (m, 2H), 1.82–1.69 (m, 2H), 1.66 (s, 3H), 1.53 (s, 3H), 1.36 (t, J = 7.2 Hz, 3H).

^{13}C NMR (125 MHz, $CDCl_3$) δ 166.3, 148.1, 146.4, 144.7, 133.9, 133.7, 132.2, 130.7, 129.9, 127.4, 127.2, 126.0, 123.6, 113.7, 113.0, 61.0, 55.6, 47.1, 32.7, 26.3, 25.8, 21.7, 17.8, 14.5.

HRMS (ESI) *m/z* calculated for $C_{26}H_{32}NO_4S$ $[M+H]^+$: 454.2052, found 454.2045.

methyl 3-(6-methylhepta-1,5-dien-2-yl)-*N*-tosylindoline-6-carboxylate (3j)

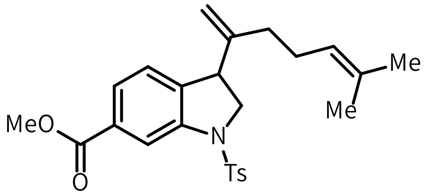

Prepared according to the general procedure. Bromoaniline **1j** (192 mg, 0.500 mmol, 1.0 equiv.), **2a** (103 μL, 0.600 mmol, 1.2 equiv.), K_2CO_3 (83 mg, 0.600 mmol, 1.2 equiv.), di(1-adamantyl)-*n*-butylphosphine (7.2 mg, 0.020 mmol, 0.04 equiv.), $Pd_2(dba)_3$ (4.6 mg, 0.005 mmol, 0.01 equiv.), and 95:5 anisole/DMF (1 mL, 0.5M) were used. The crude material was purified by flash column chromatography (silica, hexanes/ethyl acetate 19:1) to obtain products **3j** and **4j** (>95:5 r.r.) as pale yellow oil.

Run 1: (201 mg, 0.455 mmol, 91%)
Run 2: (189 mg, 0.430 mmol, 86%)
Run 3: (191 mg, 0.435 mmol, 87%)
Average yield: 88%

^{1}H NMR (500 MHz, $CDCl_3$) δ 8.29 (s, 1H), 7.69 (m, 3H), 7.23 (d, J = 8.3 Hz, 2H), 7.03 (d, J = 7.8 Hz, 1H), 4.95–4.87 (m, 1H), 4.80 (s, 1H), 4.65 (s, 1H), 4.11 (dd, J = 10.6, 9.9 Hz, 1H), 3.94 (s,

3H), 3.87 (dd, *J* = 9.9, 6.8 Hz, 1H), 3.72 (dd, *J* = 10.6, 6.8 Hz, 1H), 2.37 (s, 3H), 2.04–1.91 (m, 2H), 1.83–1.67 (m, 2H), 1.65 (s, 3H), 1.52 (s, 3H).

^{13}C NMR (125 MHz, $CDCl_3$) δ 166.9, 147.9, 144.5, 142.8, 138.8, 133.8, 132.2, 130.8, 129.9, 127.5, 125.6, 125.4, 123.5, 115.5, 113.0, 55.4, 52.4, 47.6, 32.8, 26.3, 25.8, 21.7, 17.8.

HRMS (ESI) *m/z* calculated for $C_{25}H_{30}NO_4S$ $[M+H]^+$: 440.1896, found 440.1890.

3-(6-methylhepta-1,5-dien-2-yl)-1-(methylsulfonyl)indoline (3a-Ms)

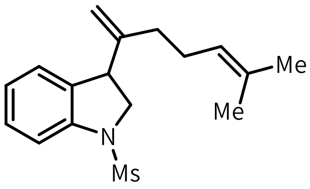

Prepared according to the general procedure. Bromoaniline **1a-Ms** (125.1 mg, 0.500 mmol, 1.0 equiv.), **2a** (103 μL, 0.600 mmol, 1.2 equiv.), K_2CO_3 (83 mg, 0.600 mmol, 1.2 equiv.), di(1-adamantyl)-*n*-butylphosphine (7.2 mg, 0.020 mmol, 0.04 equiv.), $Pd_2(dba)_3$ (4.6 mg, 0.005 mmol, 0.01 equiv.), and 95:5 anisole/DMF (1 mL, 0.5M) were used. The crude material was purified by flash column chromatography (silica, hexanes/ethyl acetate 19:1) to obtain products **3a-Ms** and **4a-Ms** (>95:5 r.r.) as pale yellow oil.

Run 1: (49 mg, 0.160 mmol, 32%)
Run 2: (49 mg, 0.161 mmol, 32%)
Average yield: 32%

^{z1}H NMR (400 MHz, $CDCl_3$) δ 7.43 (d, *J* = 8.0 Hz, 1H), 7.23 (t, *J* = 7.8 Hz, 1H), 7.12 (d, *J* = 7.3 Hz, 1H), 7.05 (t, *J* = 7.3 Hz, 1H), 5.07 (t, *J* = 6.6 Hz, 1H), 4.96 (s, 1H), 4.85 (s, 1H), 4.15 (dd, *J* = 18.1, 8.1 Hz, 1H), 4.07 – 4.02 (m, 1H), 3.77 (dd, *J* = 10.3, 6.2 Hz, 1H), 2.86 (s, 3H), 2.19 – 2.10 (m, 2H), 2.08 – 1.96 (m, 2H), 1.68 (s, 3H), 1.58 (s, 3H).

^{13}C NMR (126 MHz, CDCl3) δ 148.84, 142.35, 133.26, 132.39, 128.68, 126.02, 123.92, 123.64, 113.91, 112.67, 55.95, 47.60, 34.89, 33.46, 26.47, 25.81, 17.88.

HRMS (ESI) *m/z* calculated for $C_{17}H_{23}NO_2S$ $[M+H]^+$: 306.1523, found 306.1519.

3-(6-methylhepta-1,5-dien-2-yl)-1-(methylsulfonyl)indoline (3k)

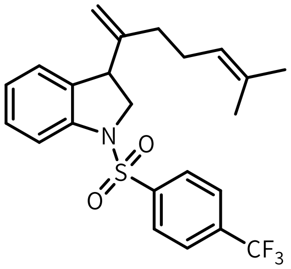

Prepared according to the general procedure. N-(2-bromophenyl)-4-(trifluoromethyl)benzenesulfonamide (190.1 mg, 0.500 mmol, 1.00 equiv.), myrcene (103.3 μg, 0.60 mmol, 1.20 equiv.), K_2CO_3 (82.9 mg, 0.600 mmol, 1.20 equiv.), di(1-adamantyl)-*n*-butylphosphine (7.2 mg, 0.020 mmol, 4 mol %), $Pd_2(dba)_3$ (4.6 mg, 0.005 mmol, 1 mol%), anisole/DMF (1 mL, 95:5) were used. Crude material was purified via column chromatography on SiO_2 using hexanes → 10% EtOAc/hexanes to afford product as yellow oil (>95:5 r.r.).

<u>Run 1:</u> (132.7 mg, 0.305 mmol, 61%).
<u>Run 2:</u> (130.0 mg, 0.299 mmol, 60%).
Average: 61%

^{1}H NMR (400 MHz, $CDCl_3$) δ 7.92 (d, *J* = 8.2 Hz, 2H), 7.69 (d, *J* = 8.4 Hz, 3H), 7.28 – 7.23 (m, 1H), 7.07 – 6.98 (m, 2H), 4.98 – 4.92 (m, 1H), 4.72 (t, *J* = 3.0 Hz, 1H), 4.52 (s, 1H), 4.11 (t, *J* =

10.3 Hz, 1H), 3.84 (dd, *J* = 9.5, 6.4 Hz, 1H), 3.72 (dd, *J* = 10.7, 6.2 Hz, 1H), 2.00 (dt, *J* = 13.8, 6.8 Hz, 2H), 1.76 (dq, *J* = 16.4, 8.1 Hz, 2H), 1.66 (s, 3H), 1.53 (s, 3H).

^{13}C NMR (101 MHz, $CDCl_3$) δ 148.4, 141.7, 140.5, 135.1, 134.8, 133.6, 132.3, 128.6, 127.9, 126.3, 126.2 (q, $J_{C\text{-}F}$ = 3.5 Hz), 126.0, 124.4, 123.5, 114.9, 112.6, 55.3, 47.4, 32.9, 26.3, 25.7, 17.8.

^{19}F NMR (376 MHz, $CDCl_3$) δ -63.4 (3F).

HRMS (ESI) m/z calculated for $C_{23}H_{24}F_3NO_2S$ $[M+H]^+$: 436.1553, found 436.1549

1,3-Diene Scope

3-(prop-1-en-2-yl)-1-tosylindoline (3ab)

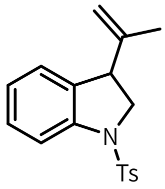

Prepared according to the general procedure. Bromoaniline **1a** (163 mg, 0.500 mmol, 1.00 equiv.), distilled isoprene (68.1 mg, 1.00 mmol, 2.00 equiv.), K_2CO_3 (82.9 mg, 0.600 mmol, 1.20 equiv.), di(1-adamantyl)-*n*-butylphosphine (7.2 mg, 0.020 mmol, 4 mol %), $Pd_2(dba)_3$ (4.6 mg, 0.005 mmol, 1 mol%), anisole/DMF (1 mL, 95:5) were used. Crude material was purified via column chromatography on SiO_2 using hexanes → 10% EtOAc/hexanes to afford product as colorless liquid (>95:5 r.r.).

<u>Run 1:</u> (116.0 mg, 0.370 mmol, 74%)
<u>Run 2:</u> (119.0 mg, 0.380 mmol, 76%)
<u>Run 3:</u> (117.5 mg, 0.375 mmol, 75%)
Average: 75% yield

^{1}H NMR (500 MHz, $CDCl_3$) δ = 7.67 (dd, J = 17.8, 9.3 Hz, 3H), 7.22 (d, J = 7.4 Hz, 3H), 6.98 (d, J = 4.2 Hz, 2H), 4.74 (d, J = 24.7 Hz, 2H), 4.04 (t, J = 10.3 Hz, 1H), 3.89 – 3.82 (m, 1H), 3.70 (dd, J = 10.5, 6.9 Hz, 1H), 2.36 (s, 3H), 1.40 (s, 3H).

^{13}C NMR (126 MHz, $CDCl_3$) δ = 144.2, 144.1, 142.3, 133.2, 129.7, 128.4, 127.5, 127.3, 125.4, 123.8, 114.8, 113.8, 54.3, 48.2, 21.6, 18.5.

HRMS (ESI) m/z calculated for $C_{18}H_{19}NO_2S$ $[M+H]^+$: 314.1209, found 314.1207.

3-(3-phenylprop-1-en-2-yl)-1-tosylindoline (3ac)

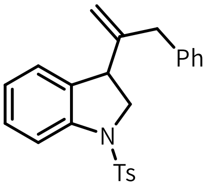

Prepared according to the general procedure. Bromoaniline **1a** (163 mg, 0.500 mmol, 1.00 equiv.), diene **2c** (86.5 mg, 0.60 mmol, 1.20 equiv.), K_2CO_3 (82.9 mg, 0.600 mmol, 1.20 equiv.), di(1-adamantyl)-*n*-butylphosphine (7.2 mg, 0.020 mmol, 4 mol %), $Pd_2(dba)_3$ (4.6 mg, 0.005 mmol, 1 mol%), anisole/DMF (1 mL, 95:5) were used. Crude material was purified via column chromatography on SiO_2 using hexanes → 10% EtOAc/hexanes to afford product as white solid (94:6 r.r.).

<u>Run 1:</u> (130.5 mg, 0.335 mmol, 67%)
<u>Run 2:</u> (132.4 mg, 0.340 mmol, 68%)
<u>Run 3:</u> (132.5 mg, 0.340 mmol, 68%)
Average: 68% yield

^1H NMR (500 MHz, $CDCl_3$) δ 7.68 (d, J = 8.1 Hz, 1H), 7.63 (d, J = 8.0 Hz, 2H), 7.26 (m, 2H), 7.22 – 7.16 (m, 4H), 7.02 (d, J = 7.3 Hz, 2H), 7.00 – 6.94 (m, 2H), 4.65 (d, J = 16.9, 2H), 3.99 (t, J = 10.2 Hz, 1H), 3.79 (dd, J = 9.4, 6.9 Hz, 1H), 3.67 (dd, J = 10.6, 6.6 Hz, 1H), 3.06 (q, J = 15.7 Hz, 2H), 2.31 (s, 3H).

^{13}C NMR (126 MHz, $CDCl_3$) δ 148.6, 144.3, 142.4, 138.7, 133.9, 133.5, 129.8, 129.3, 128.5, 128.4, 127.5, 126.5, 125.6, 123.9, 115.0, 114.8z, 55.5, 46.6, 40.4, 21.6.

HRMS (ESI) m/z calculated for $C_{24}H_{23}NO_2S$ $[M+H]^+$: 390.1523, found 390.1519.

3-(1-phenylvinyl)-1-tosylindoline (3ad)

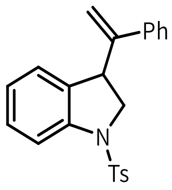

Prepared according to the general procedure. Bromoaniline **1a** (163 mg, 0.500 mmol, 1.00 equiv.), diene **3d** (78.1 mg, 0.60 mmol, 1.20 equiv.), K_2CO_3 (82.9 mg, 0.600 mmol, 1.20 equiv.), di(1-adamantyl)-*n*-butylphosphine (7.2 mg, 0.020 mmol, 4 mol %), $Pd_2(dba)_3$ (4.6 mg, 0.005 mmol, 1 mol%), anisole/DMF (1 mL, 95:5) were used. Crude material was purified via column chromatography on SiO_2 using hexanes → 10% EtOAc/hexanes to afford product as white solid (>95:5 r.r.).

Run 1: (127.7 mg, 0.340 mmol, 68%)
Run 2: (135.1 mg, 0.360 mmol, 72%)
Run 3: (127.6 mg, 0.340 mmol, 68%)
Average: 69% yield

^1H NMR (500 MHz, $CDCl_3$) δ 7.69 (d, J = 7.8 Hz, 1H), 7.60 (d, J = 8.0 Hz, 2H), 7.25 – 7.20 (m, J = 7.5 Hz, 3H), 7.17 (d, J = 7.9 Hz, 3H), 7.13 (d, J = 6.7 Hz, 2H), 7.05 (d, J = 7.4 Hz, 1H), 7.00 (t, J = 7.4 Hz, 1H), 5.21 (s, 1H), 4.63 (s, 1H), 4.33 – 4.27 (m, 1H), 4.14 (t, J = 10.3 Hz, 1H), 3.72 – 3.64 (m, 1H), 2.36 (s, 3H).

^{13}C NMR (126 MHz, $CDCl_3$) δ 148.6, 144.1, 142.5, 140.1, 134.1, 133.5, 129.7, 128.6, 128.6, 127.9, 127.5, 126.6, 126.0, 123.9, 115.5, 115.2, 56.1, 45.5, 21.7.

HRMS (ESI) m/z calculated for $C_{23}H_{21}NO_2S$ $[M+H]^+$: 376.1366, found 376.1362.

3-(5,5-dimethoxypent-1-en-2-yl)-1-tosylindoline (3ae)

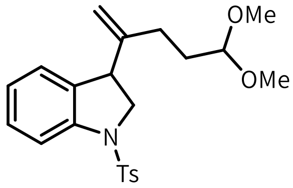

Prepared according to the general procedure. Bromoaniline **1a** (163 mg, 0.500 mmol, 1.00 equiv.), diene **2e** (93.7 mg, 0.60 mmol, 1.20 equiv.), K_2CO_3 (82.9 mg, 0.600 mmol, 1.20 equiv.), di(1-adamantyl)-*n*-butylphosphine (7.2 mg, 0.020 mmol, 4 mol %), $Pd_2(dba)_3$ (4.6 mg, 0.005 mmol, 1 mol%), anisole/DMF (1 mL, 95:5) were used. Crude material was purified via column chromatography on SiO_2 using hexanes → 10% EtOAc/hexanes to afford product as colorless oil (>95:5 r.r.).

Run 1: (130.5 mg, 0.325 mmol, 65%)
Run 2: (134.0 mg, 0.334 mmol, 67%)
Run 3: (129.6 mg, 0.323 mmol, 65%)
Average: 66% yield

^1H NMR (500 MHz, $CDCl_3$) δ 7.67 (m, 3H), 7.22 (d, J = 8.1 Hz, 3H), 6.99 – 6.96 (m, 2H), 4.77 (s, 1H), 4.63 (s, 1H), 4.24 (t, J = 5.6 Hz, 1H), 4.06 (t, J = 10.2 Hz, 1H), 3.82 (dd, J = 9.6, 6.8 Hz, 1H), 3.70 (dd, J = 10.6, 6.7 Hz, 1H), 3.27 (s, 3H), 3.25 (s, 3H), 2.36 (s, 3H), 1.91 – 1.78 (m, 2H), 1.71 – 1.57 (m, 2H).

^{13}C NMR (126 MHz, $CDCl_3$) δ 148.1, 144.2, 142.4, 134.1, 133.4, 129.7, 128.4, 127.5, 125.6, 123.8, 114.9, 112.4, 104.0, 55.3, 53.0, 52.7, 47.5, 30.8, 27.9, 21.6.

HRMS (ESI) m/z calculated for $C_{22}H_{27}NO_4S$ $[M+Na]^+$: 424.1559, found 424.1550.

3-(5-((*tert*-butyldimethylsilyl)oxy)pent-1-en-2-yl)-1-tosylindoline (3af)

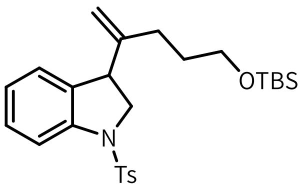

Prepared according to the general procedure. Bromoaniline **1a** (163 mg, 0.500 mmol, 1.00 equiv.), diene **2f** (135.9 mg, 0.600 mmol, 1.20 equiv.), K_2CO_3 (82.9 mg, 0.600 mmol, 1.20 equiv.), di(1-adamantyl)-*n*-butylphosphine (7.2 mg, 0.020 mmol, 4 mol %), $Pd_2(dba)_3$ (4.6 mg, 0.005 mmol, 1 mol%), anisole/DMF (1 mL, 95:5) were used. Crude material was purified via column chromatography on SiO_2 using hexanes → 10% EtOAc/hexanes to afford product as colorless oil (>95:5 r.r.).

<u>Run 1:</u> (198.8 mg, 0.463 mmol, 92%)
<u>Run 2:</u> (202.9 mg, 0.472 mmol, 94%)
<u>Run 3:</u> (206.5 mg, 0.480 mmol, 96%)
Average: 94% yield

^{1}H NMR (400 MHz, $CDCl_3$) δ 7.67 (m, 3H), 7.25 – 7.19 (m, 3H), 6.97 (d, *J* = 4.3 Hz, 2H), 4.76 (s, 1H), 4.62 (s, 1H), 4.06 (t, *J* = 10.2 Hz, 1H), 3.81 (m, 1H), 3.69 (dd, *J* = 9.9, 6.0 Hz, 1H), 3.49 (dd, *J* = 9.1, 3.6 Hz, 2H), 2.37 (s, 3H), 1.80 (dt, *J* = 14.2, 7.3 Hz, 2H), 1.61 – 1.50 (m, 2H), 0.86 (s, 9H), 0.01 (s, 6H).

^{13}C NMR (101 MHz, $CDCl_3$) δ 148.56, 144.17, 142.36, 134.05, 133.55, 129.74, 128.37, 127.50, 125.57, 123.79, 114.88, 112.22, 77.16, 62.66, 55.34, 47.55, 30.99, 29.12, 26.06, 21.67, 18.42, -5.16.

HRMS (ESI) m/z calculated for $C_{26}H_{37}NO_3SSi$ $[M+H]^+$: 472.2336, found 472.2331.

2-(4-(1-tosylindolin-3-yl)pent-4-en-1-yl)isoindoline-1,3-dione (3ag)

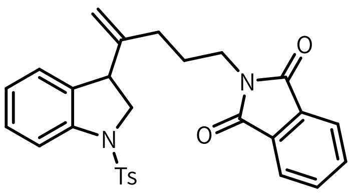

Prepared according to the general procedure. Bromoaniline **1a** (163 mg, 0.500 mmol, 1.00 equiv.), diene **2g** (144.8 mg, 0.600 mmol, 1.20 equiv.), K_2CO_3 (82.9 mg, 0.600 mmol, 1.20 equiv.), di(1-adamantyl)-*n*-butylphosphine (7.2 mg, 0.020 mmol, 4 mol %), $Pd_2(dba)_3$ (4.6 mg, 0.005 mmol, 1 mol%), anisole/DMF (1 mL, 95:5) were used. Crude material was purified via column chromatography on SiO_2 using hexanes → 25% EtOAc/hexanes to afford product as colorless oil (>95:5 r.r.).

<u>Run 1:</u> (194.9 mg, 0.401 mmol, 80%)
<u>Run 2:</u> (196.6 mg, 0.404 mmol, 81%)
<u>Run 3:</u> (194.3 mg, 0.399 mmol, 80%)
Average: 80% yield

^{1}H NMR (400 MHz, $CDCl_3$) δ 7.84 (dd, *J* = 5.4, 3.1 Hz, 2H), 7.72 (dd, *J* = 5.4, 3.0 Hz, 2H), 7.67 (m, 3H), 7.24 (d, *J* = 8.1 Hz, 2H), 7.20 (dd, *J* = 8.5, 4.4 Hz, 1H), 6.97 (d, *J* = 4.3 Hz, 2H), 4.79 (s, 1H), 4.64 (s, 1H), 4.07 (t, *J* = 10.3 Hz, 1H), 3.82 (dd, *J* = 9.9, 6.4 Hz, 1H), 3.66 (dd, *J* = 10.7, 6.4 Hz, 1H), 3.57 – 3.51 (m, 2H), 2.37 (s, 3H), 1.81 – 1.65 (m, 4H).

^{13}C NMR (101 MHz, $CDCl_3$) δ 168.4, 147.4, 144.3, 142.4, 134.1, 133.9, 133.3, 132.2, 129.8, 128.5, 127.5, 125.6, 123.9, 123.4, 114.9, 112.7, 55.3, 47.4, 37.6, 29.9, 26.4, 21.7.

HRMS (ESI) m/z calculated for $C_{28}H_{26}N_2O_4S$ $[M+H]^+$: 487.1686, found 487.1686.

3-(4-(3,3-dimethyloxiran-2-yl)but-1-en-2-yl)-1-tosylindoline (3ah)

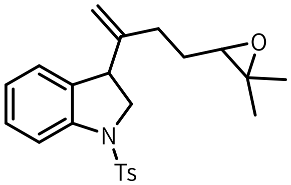

Prepared according to the general procedure. Bromoaniline **1a** (163 mg, 0.500 mmol, 1.00 equiv.), diene **2h** (91.3 mg, 0.60 mmol, 1.20 equiv.), K_2CO_3 (82.9 mg, 0.600 mmol, 1.20 equiv.), di(1-adamantyl)-*n*-butylphosphine (7.2 mg, 0.020 mmol, 4 mol %), $Pd_2(dba)_3$ (4.6 mg, 0.005 mmol, 1 mol%), anisole/DMF (1 mL, 95:5) were used. Crude material was purified via column chromatography on SiO_2 using hexanes → 10% EtOAc/hexanes to afford product as colorless oil (>95:5 r.r.).

<u>Run 1:</u> (119.2 mg, 0.300 mmol, 60%)
<u>Run 2:</u> (120.3 mg, 0.303 mmol, 61%)
<u>Run 3:</u> (123.1 mg, 0.310 mmol, 62%)
Average: 61% yield

^{1}H NMR (500 MHz, $CDCl_3$) δ 7.68 (dd, *J* = 7.9, 5.7 Hz, 3H), 7.25 – 7.20 (m, 3H), 7.00 – 6.96 (m, 2H), 4.79 (s, 1H), 4.70 (s, 1H), 4.06 (td, *J* = 10.3, 3.8 Hz, 1H), 3.86 (dd, *J* = 9.0, 7.2 Hz, 1H), 3.74 – 3.67 (m, 1H), 2.61 – 2.51 (m, 1H), 2.36 (s, 3H), 2.00 – 1.76 (m, 2H), 1.61 – 1.49 (m, 2H), 1.27 (s, 3H), 1.19 (d, *J* = 16.7 Hz, 3H).

^{13}C NMR (126 MHz, $CDCl_3$) δ 147.9, 147.9, 144.3, 144.2, 142.4, 142.4, 134.1, 133.9, 133.2, 129.8, 129.7, 129.4, 128.5, 127.5, 127.4, 127.3, 125.6, 125.5, 125.4, 123.8, 123.8, 121.8, 114.9, 112.8, 112.7, 63.7, 63.7, 58.4, 58.3, 55.2, 55.1, 47.7, 47.6, 29.5, 29.4, 27.5, 27.2, 24.9, 21.6, 18.8, 18.7.
HRMS (ESI) m/z calculated for $C_{23}H_{27}NO_3S$ $[M+H]^+$: 398.1785, found 398.1778.

3-(5-(3-(furan-2-yl)propoxy)pent-1-en-2-yl)-1-tosylindoline (3ai)

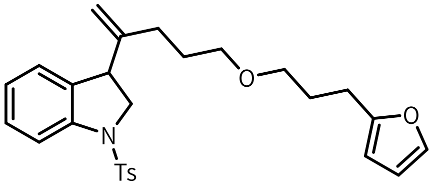

Prepared according to the general procedure. Bromoaniline **1a** (163 mg, 0.500 mmol, 1.00 equiv.), diene **2i** (132.2 mg, 0.600 mmol, 1.20 equiv.), K_2CO_3 (82.9 mg, 0.600 mmol, 1.20 equiv.), di(1-adamantyl)-*n*-butylphosphine (7.2 mg, 0.020 mmol, 4 mol %), $Pd_2(dba)_3$ (4.6 mg, 0.005 mmol, 1 mol%), anisole/DMF (1 mL, 95:5) were used. Crude material was purified via column chromatography on SiO_2 using hexanes → 15% EtOAc/hexanes to afford product as colorless oil (>95:5 r.r.).

<u>Run 1:</u> (200.5 mg, 0.431 mmol, 86%)
<u>Run 2:</u> (202.1 mg, 0.434 mmol, 87%)
<u>Run 3:</u> (205.8 mg, 0.442 mmol, 88%)
Average: 87% yield

^{1}H NMR (400 MHz, $CDCl_3$) δ 7.68 (d, *J* = 8.1 Hz, 3H), 7.29 (s, 1H), 7.21 (d, *J* = 8.0 Hz, 3H), 6.97 (d, *J* = 4.2 Hz, 2H), 6.28 (s, 1H), 5.98 (d, *J* = 2.2 Hz, 1H), 4.78 (s, 1H), 4.64 (s, 1H), 4.07 (t, *J* = 10.2 Hz, 1H), 3.88 – 3.80 (m, 1H), 3.70 (dd, *J* = 10.5, 6.6 Hz, 1H), 3.38 (t, *J* = 6.3 Hz, 2H), 3.28 (t, *J* = 6.4 Hz, 2H), 2.67 (t, *J* = 7.4 Hz, 2H), 2.36 (s, 3H), 1.85 (ddd, *J* = 18.9, 14.2, 6.7 Hz, 4H), 1.68 – 1.53 (m, 2H).

^{13}C NMR (101 MHz, $CDCl_3$) δ 155.8, 148.3, 144.1, 142.3, 140.9, 134.0, 133.5, 129.7, 128.3, 127.5, 125.5, 123.8, 114.8, 112.3, 110.2, 105.0, 70.2, 69.9, 55.3, 47.5, 29.3, 28.2, 27.8, 24.7, 21.6.

HRMS (ESI) m/z calculated for $C_{27}H_{31}NO_4S$ $[M+H]^+$: 466.2047, found 466.2045.

1-methyl-2-(((4-(1-tosylindolin-3-yl)pent-4-en-1-yl)oxy)methyl)-1*H*-indole (3aj)

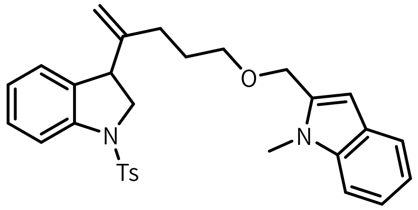

Prepared according to the general procedure. Bromoaniline **1a** (163 mg, 0.500 mmol, 1.00 equiv.), diene **2j** (153.2 mg, 0.600 mmol, 1.20 equiv.), K_2CO_3 (82.9 mg, 0.600 mmol, 1.20 equiv.), di(1-adamantyl)-*n*-butylphosphine (7.2 mg, 0.020 mmol, 4 mol %), $Pd_2(dba)_3$ (4.6 mg, 0.005 mmol, 1 mol%), anisole/DMF (1 mL, 95:5) were used. Crude material was purified via column chromatography on SiO_2 using hexanes → 20% EtOAc/hexanes to afford product as colorless oil (>95:5 r.r.).

Run 1: (211.5 mg, 0.422 mmol, 84%)
Run 2: (217.0 mg, 0.433 mmol, 86%)
Run 3: (214.9 mg, 0.429 mmol, 86%)
Average: 85% yield

^1H NMR (400 MHz, $CDCl_3$) δ 7.69 (t, *J* = 8.5 Hz, 4H), 7.32 (d, *J* = 8.1 Hz, 1H), 7.26 (m, 2H), 7.17 (m, 3H), 7.05 (s, 1H), 6.98 (m, 2H), 4.78 (s, 1H), 4.67 (s, 2H), 4.62 (s, 1H), 4.07 (t, *J* = 10.1 Hz, 1H), 3.83 – 3.78 (m, 1H), 3.77 (s, 3H), 3.70 (dd, *J* = 10.4, 6.7 Hz, 1H), 3.41 (t, *J* = 6.4 Hz, 2H), 2.34 (s, 3H), 1.85 (m, 2H), 1.67 (m, 2H).

^{13}C NMR (101 MHz, $CDCl_3$) δ 148.4, 144.1, 142.3, 137.3, 133.9, 133.5, 129.7, 128.4, 128.3, 127.7, 127.4, 125.6, 123.7, 121.9, 119.4, 119.3, 114.8, 112.2, 111.9, 109.4, 69.0, 64.7, 55.3, 47.4, 32.7, 29.5, 27.9, 21.5.

HRMS (ESI) m/z calculated for $C_{30}H_{32}N_2O_3S$ $[M+H]^+$: 501.2207, found 501.2203.

3-(1-((3*r*,5*r*,7*r*)-adamantan-1-yl)vinyl)-1-tosylindoline (3ak)

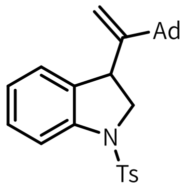

Prepared according to the general procedure. Bromoaniline **1a** (163 mg, 0.500 mmol, 1.00 equiv.), diene **2k** (113.0 mg, 0.600 mmol, 1.20 equiv.), K_2CO_3 (82.9 mg, 0.600 mmol, 1.20 equiv.), di(1-adamantyl)-*n*-butylphosphine (7.2 mg, 0.020 mmol, 4 mol %), $Pd_2(dba)_3$ (4.6 mg, 0.005 mmol, 1 mol%), anisole/DMF (1 mL, 95:5) were used. Crude material was purified via column chromatography on SiO_2 using hexanes → 10% EtOAc/hexanes to afford product as colorless oil.

Run 1: (197.6 mg, 0.446 mmol, 91%).
Run 2: (200.0 mg, 0.461 mmol, 92%).
Run 3: (202.1 mg, 0.466 mmol, 93%).
Average: 92% yield (80:20 r.r. **3ak**:**5ak**).

^1H NMR (400 MHz, $CDCl_3$) δ 7.65 (t, *J* = 8.2 Hz, 3H), 7.22 (d, *J* = 8.3 Hz, 2H), 7.18 (d, *J* = 7.8 Hz, 1H), 6.96 (t, *J* = 7.4 Hz, 1H), 6.84 (d, *J* = 7.4 Hz, 1H), 4.80 (s, 1H), 4.25 – 4.12 (m, 2H), 3.79 (t, *J* = 8.8 Hz, 1H), 3.45 (dd, *J* = 10.2, 8.6 Hz, 1H), 2.37 (s, 3H), 2.04 (bs, 3H), 1.75 (d, *J* = 12.0 Hz, 3H), 1.70 – 1.54 (m, 9H).

^{13}C NMR (101 MHz, $CDCl_3$) δ 161.6, 160.5, 144.1, 142.6, 139.8, 137.1, 129.7, 127.8, 127.6, 127.1, 125.5, 124.2, 124.1, 122.5, 115.0, 110.9, 60.7, 40.9, 40.7, 37.9, 36.9, 36.6, 28.6, 21.7.

HRMS (ESI) m/z calculated for $C_{27}H_{31}NO_2S$ $[M+H]^+$: 434.2149, found 434.2148.

3-((3*r*,5*r*,7*r*)-adamantan-1-yl)-1-tosyl-3-vinylindoline (5ak)

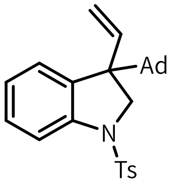

^1H NMR (400 MHz, $CDCl_3$) δ 7.71 (d, *J* = 8.1 Hz, 2H), 7.66 (d, *J* = 8.0 Hz, 1H), 7.22 (d, *J* = 7.8 Hz, 3H), 7.17 (d, *J* = 7.5 Hz, 1H), 6.97 (t, *J* = 7.4 Hz, 1H), 6.13 (dd, *J* = 17.4, 10.8 Hz, 1H), 5.00 (d, *J* = 10.8 Hz, 1H), 4.58 (d, *J* = 17.4 Hz, 1H), 4.14 (d, *J* = 10.6 Hz, 1H), 3.60 (d, *J* = 10.6 Hz, 1H), 2.35 (s, 3H), 1.89 (bs, 3H), 1.60 (d, *J* = 10.2 Hz, 6H), 1.56 – 1.47 (m, 3H), 1.36 (d, *J* = 11.9 Hz, 3H).

^{13}C NMR (126 MHz, $CDCl_3$) δ 144.1, 142.6, 139.8, 134.1, 133.6, 129.7, 128.3, 127.6, 127.1, 122.5, 115.6, 113.8, 56.7, 56.1, 40.5, 38.8, 36.8, 36.6, 28.6, 21.6.

HRMS (ESI) m/z calculated for $C_{27}H_{31}NO_2S$ $[M+H]^+$: 434.2149, found 434.2148.

(*E*)- 2-styryl-*N*-tosylindoline (4al)

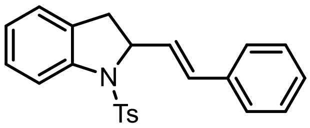

Prepared according to the general procedure. *N*-tosyl-2-bromoaniline (163 mg, 0.500 mmol, 1.00 equiv.), (*E*)-1-Phenyl-1,3-butadiene (84.6mg, 0.650 mmol, 1.30 equiv.), K_2CO_3 (82.9 mg, 0.600 mmol, 1.20 equiv.), di(1-adamantyl)-*n*-butylphosphine (7.2 mg, 0.020 mmol, 4 mol %), $Pd_2(dba)_3$ (4.6 mg, 0.005 mmol, 1 mol%), anisole/DMF (1 mL, 95:5) were used. Crude material was purified via column chromatography on SiO_2 using 5% → 10% EtOAc/hexanes. Product was isolated as white foam (>95:5 *E/Z*).

^1H NMR (400 MHz, $CDCl_3$) δ 7.68 (d, J = 8.0 Hz, 1H), 7.62 (d, J = 8.2 Hz, 2H), 7.33 – 7.28 (m, 4H), 7.23 (d, J = 7.0 Hz, 2H), 7.15 (d, J = 8.1 Hz, 2H), 7.09 – 7.01 (m, 2H), 6.69 (d, J = 15.8 Hz, 1H), 6.16 (dd, J = 15.8, 7.2 Hz, 1H), 4.97 (td, J = 6.9 Hz, 2.6 Hz, 1H), 3.08 (dd, J = 16.0, 9.6 Hz, 1H), 2.73 (dd, J = 16.0, 2.6 Hz, 1H), 2.37 (d, J = 19.7 Hz, 3H). Spectral data agree with that reported in literature. [1]

2-phenyl-1-tosyl-3-vinylindoline (6al)

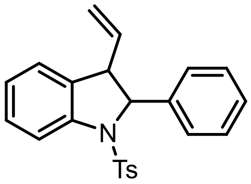

^1H NMR (400 MHz, $CDCl_3$) δ 7.79 (d, *J* = 8.1 Hz, 1H), 7.58 (d, *J* = 8.2 Hz, 2H), 7.36 – 7.27 (m, 6H), 7.18 (d, *J* = 8.1 Hz, 2H), 7.06 (t, *J* = 7.2 Hz, 1H), 6.99 (d, *J* = 7.2 Hz, 1H), 5.27 – 5.11 (m, 1H), 4.91 (d, *J* = 3.6 Hz, 1H), 4.82 (s, 1H), 4.78 (d, *J* = 6.6 Hz, 1H), 3.63 (dd, *J* = 8.2, 3.3 Hz, 1H), 2.37 (s, 3H).

^{13}C NMR (126 MHz, $CDCl_3$) δ 144.1, 142.5, 141.9, 138.4, 134.9, 132.6, 129.6, 129.1, 128.8, 128.7, 128.6, 127.8, 127.6, 126.0, 125.8, 124.6, 116.1, 115.8, 71.3, 55.9, 21.7.

HRMS (ESI) m/z calculated for $C_{23}H_{21}NO_2S$ $[M+H]^+$: 376.1366, found 376.1357.

<u>Run 1:</u> (35.7 mg, 0.095 mmol, 19%)
<u>Run 2:</u> (31.8 mg, 0.085 mmol, 17%)
<u>Run 3:</u> (37.9 mg, 0.101 mmol, 20%)
Average yield: 19% (66:29:5 r.r. **4al**:**6al:3al**).

Tosyl Group Deprotection

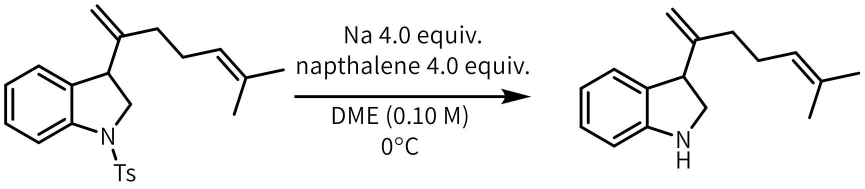

3-(6-methylhepta-1,5-dien-2-yl)indoline (3l)

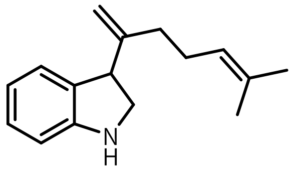

Sodium metal (71.7 mg, 3.12 mmol, 4.00 equiv.) and naphthalene (400 mg, 3.12 mmol, 4.00 equiv.) was taken up in DME (8.0 mL, 0.10 M) in a round bottomed flask equipped with stir bar and cooled to 0°C. After half an hour the solution was cannulated into a separate dry round bottomed flask containing 3-(6-methylhepta-1,5-dien-2-yl)-1-tosylindoline (300 mg, 0.78 mmol, 1.00 equiv.), cooled to 0°C and left to stir for three hours. The reaction mixture was diluted with 10% HCl (15 mL) extracted with Et_2O (3 x 10 mL), combined organic layers washed with brine (3 x 5 mL) three times and dried over $MgSO_4$, filtered, and concentrated under reduced pressure. Crude material was purified via column chromatography on SiO_2 using 5% EtOAc/hexanes. Pure product was isolated as pale yellow oil (753.2.0 mg, 2.95 mmol, 64% yield over three steps).

Run 1: (117.6 mg, 0.517 mmol, 66%)
Run 2: (123.7 mg, 0.544 mmol, 70%)
Average: 68% yield

^{1}H NMR (400 MHz, $CDCl_3$) δ 7.04 (m, 2H), 6.73 (t, *J* = 7.4 Hz, 1H), 6.66 (d, *J* = 7.7 Hz, 1H), 5.15 – 5.06 (m, 1H), 4.91 (d, *J* = 5.0 Hz, 2H), 4.00 (t, *J* = 8.6 Hz, 1H), 3.73 (m, 2H), 3.40 (t, *J* = 8.4 Hz, 1H), 2.14 (m, 2H), 2.12 – 2.00 (m, 2H), 1.68 (s, 3H), 1.60 (s, 3H).

^{13}C NMR (101 MHz, $CDCl_3$) δ 151.8, 149.9, 131.8, 131.2, 127.8, 125.1, 124.2, 118.9, 110.9, 109.8, 53.2, 49.9, 33.6, 26.6, 25.8, 17.8.

HRMS (ESI) m/z calculated for $C_{23}H_{28}NO_3S$ $[M+H]^+$: 228.1747, found 228.1743.

Gram Scale Reaction

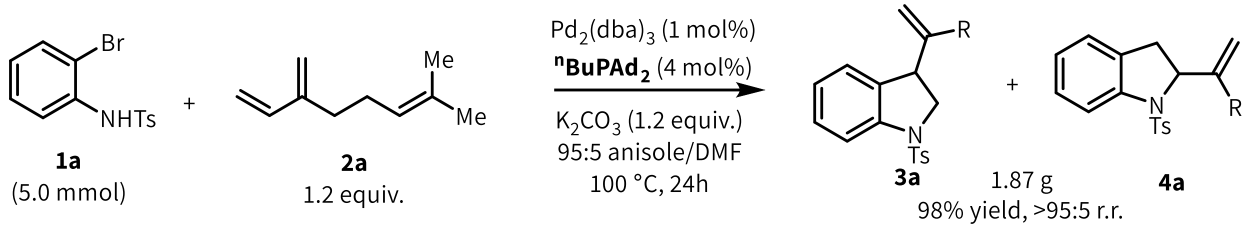

N-Tosylbromoaniline **1a** (1.63 g, 5.00 mmol, 1.0 equiv.), myrcene **2a** (1.03 mL, 6.00 mmol, 1.2 equiv.), di(1-adamantyl)-*n*-butylphosphine (71 mg, 0.20 mmol, 0.04 equiv.), potassium carbonate (830 mg, 6.00 mmol, 1.2 equiv.), tris(dibenzylideneacetone)dipalladium(0) (46 mg, 0.05 mmol, 0.01 equiv.) and 1,3,5-trimethoxybenze (internal standard, 40 mg, 0.238 mmol, 0.05 equiv.) were weighed out in the above-mentioned order into a 4-dram vial equipped with a stir bar and a cap with a silicone septum. The vial was then placed under nitrogen atmosphere and charged with 10 mL of a freshly degassed anisole/dimethylformamide (95:5) solvent mixture. The reaction was stirred at 100 °C for 24 h. After cooling to room temperature, the reaction mixture was filtered with ethyl acetate through celite. The solvents were removed under reduced pressure and the crude mixture was purified by flash column chromatography (SiO_2, hexanes/ethyl acetate 18:2) to obtain products **3a** and **4a** (>99:1 r.r.).

Run 1: (1.89 g, 4.95 mmol, 99%)
Run 2: (1.87 g, 4.90 mmol, 98%)
Run 3: (1.88 g, 4.93 mmol, 98%)

Average: 98% yield

Reactivity with Mesyl-Protected Anilines

3-(6-methylhepta-1,5-dien-2-yl)-1-(methylsulfonyl)indoline (3a-Ms)

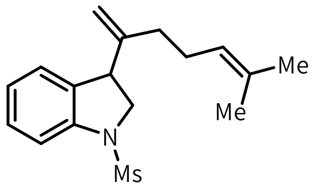

Prepared according to the general procedure. Bromoaniline **1a-Ms** (125.1 mg, 0.500 mmol, 1.0 equiv.), **2a** (103 µL, 0.600 mmol, 1.2 equiv.), K_2CO_3 (83 mg, 0.600 mmol, 1.2 equiv.), di(1-adamantyl)-*n*-butylphosphine **L1** (14.4 mg, 0.040 mmol, 0.08 equiv.), $Pd_2(dba)_3$ (9.2 mg, 0.01 mmol, 0.02 equiv.), and 95:5 anisole/DMF (1 mL, 0.5M) were used. The reaction was warmed up to 120 °C. The crude material was purified by flash column chromatography (silica, hexanes/ethyl acetate 19:1) to obtain products **3a-Ms** and **4a-Ms** (>95:5 r.r.) as pale yellow oil.

Run 1: (103.8 mg, 0.160 mmol, 68%)
Run 2: (110.6 mg, 0.161 mmol, 72%)
Average yield: 70%

1-(methylsulfonyl)-3-(prop-1-en-2-yl)indoline (3ab-Ms)

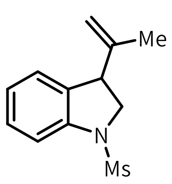

Prepared according to the general procedure; the Pd source and ligand were pre-stirred in the reaction solvent for 20 min prior to addition of the other reaction components. Bromoaniline **1a-Ms** (125.1 mg, 0.500 mmol, 1.0 equiv.), distilled isoprene (68.1 mg, 1.00 mmol, 2.00 equiv.), K_2CO_3 (82.9 mg, 0.600 mmol, 1.20 equiv.), di-tert-butyl(methyl)phosphonium tetrafluoroborate (7.2 mg, 0.040 mmol, 0.08 equiv.), $Pd_2(dba)_3$ (9.2 mg, 0.01 mmol, 2 mol%), anisole/DMF (1 mL, 95:5) were used. The reaction was warmed up to 120 °C. Crude material was purified via column chromatography on SiO_2 using hexanes → 10% EtOAc/hexanes to afford product as colorless liquid (>95:5 r.r.).

Run 1: (37.0 mg, 0.156 mmol, 31%)
Run 2: (42.5 mg, 0.179 mmol, 36%)
Average: 34% yield

^{1}H NMR (400 MHz, $CDCl_3$) δ 7.42 (d, J = 8.1 Hz, 1H), 7.23 (d, t = 7.6 Hz, 1H), 7.13 (d, J = 7.4 Hz, 1H), 7.05 (td, J = 7.4, 0.6 Hz, 1H), 4.90 (d, J = 21.0 Hz, 2H), 4.16 – 4.03 (m, 2H), 3.80 (dd, J = 9.5, 5.5 Hz, 1H), 2.87 (s, 3H), 1.68 (s, 3H).

^{13}C NMR (101 MHz, $CDCl_3$) δ 143.15, 141.21, 131.95, 127.74, 124.78, 122.94, 113.15, 112.82, 54.01, 47.21, 33.81, 18.20.

HRMS (ESI) m/z calculated for $C_{23}H_{28}NO_3S$ $[M+H]^+$: 238.0896, found 238.0894.

Method Limitations

Table S3. Unsuccessful substrates.

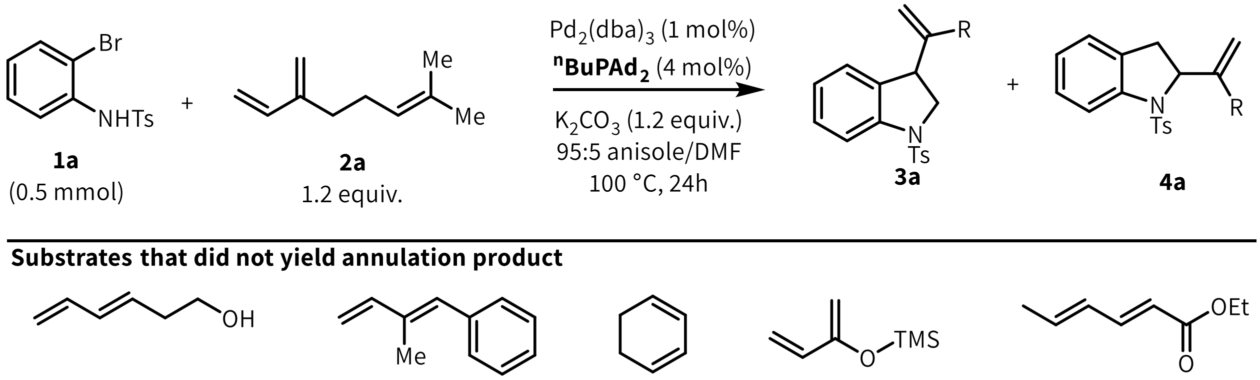

Confirmation of Regioselectivity X-Ray Structure of 3ad

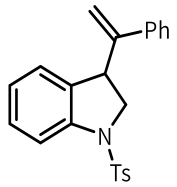

Indoline **3ad** (100.0 mg, 0.266 mmol, 1.00 equiv) was taken up in hexanes (2.0 mL, 0.13 M) and heated to 65 °C, then ethanol (0.5 mL) was added dropwise until the solid dissolved. The mixture was left to cool to room temperature overnight to afford pale yellow square crystals (100 mg, 0.266 mmol, quant. yield).

Figure S2. Crystal Structure of 3ad.

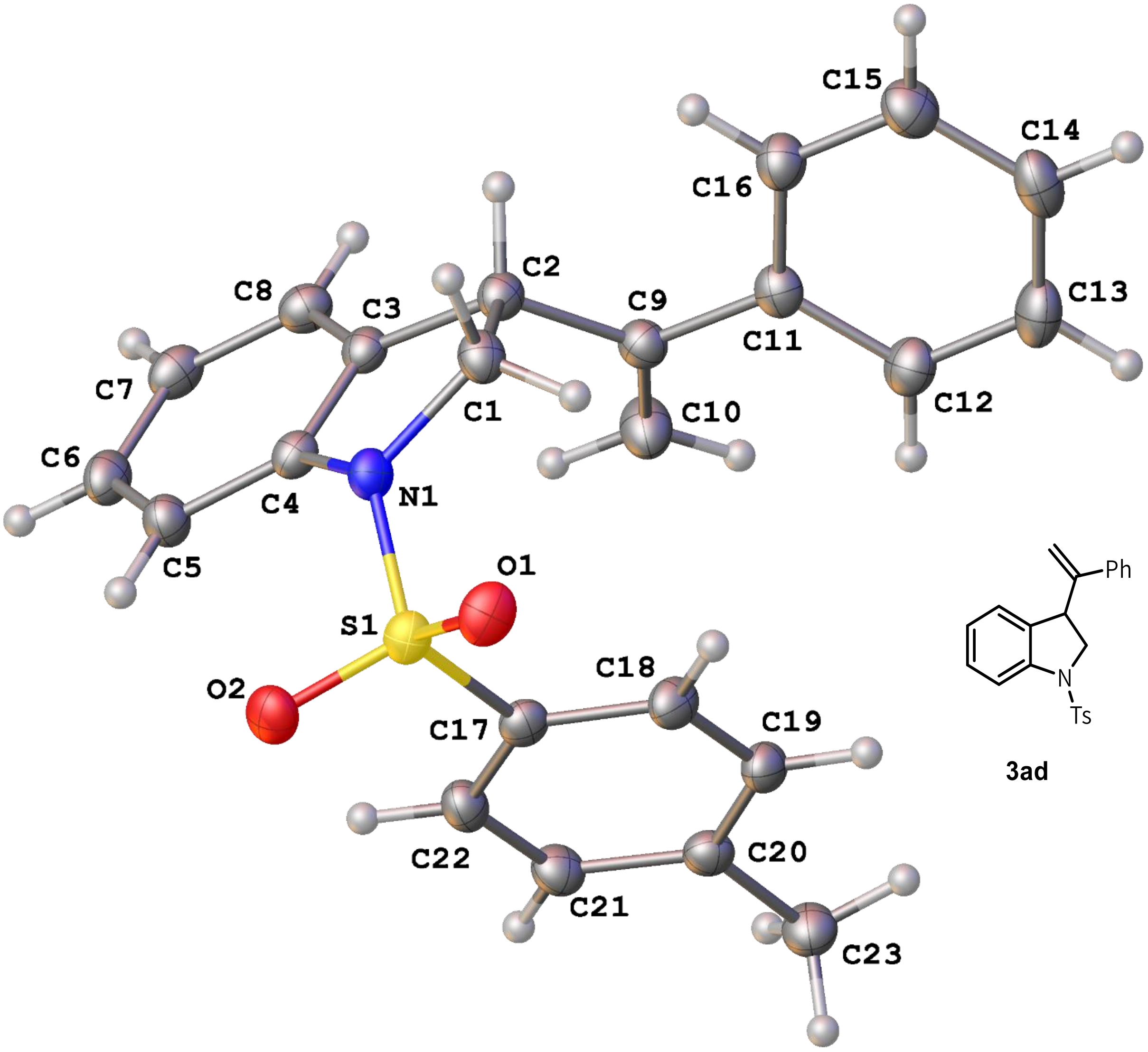

3ad

Table S4. Crystal data and structure refinement for **3ad**.

Identification code	pardr01	
Empirical formula	C23 H21 N O2 S	
Formula weight	375.47	
Temperature	100.00(10) K	
Wavelength	1.54184 Å	
Crystal system	monoclinic	
Space group	$P2_1/c$	
Unit cell dimensions	*a* = 14.27050(10) Å	a = 90°
	b = 9.12020(10) Å	b = 116.0070(10)°
	c = 15.9397(2) Å	g = 90°
Volume	1864.48(4) Å^3	
Z	4	
Density (calculated)	1.338 Mg/m^3	
Absorption coefficient	1.680 mm^{-1}	
F(000)	792	
Crystal color, morphology	pale yellow, block	
Crystal size	0.278 x 0.244 x 0.191 mm^3	
Theta range for data collection	3.446 to 80.361°	
Index ranges	-18 £ *h* £ 18, -10 £ *k* £ 11, -20 £ *l* £ 20	
Reflections collected	32912	
Independent reflections	4041 [*R*(int) = 0.0360]	
Observed reflections	3856	
Completeness to theta = 74.504°	100.0%	
Absorption correction	Multi-scan	
Max. and min. transmission	1.00000 and 0.87014	
Refinement method	Full-matrix least-squares on F^2	
Data / restraints / parameters	4041 / 0 / 253	
Goodness-of-fit on F^2	1.054	
Final *R* indices [*I*>2sigma(*I*)]	*R*1 = 0.0370, *wR*2 = 0.0943	
R indices (all data)	*R*1 = 0.0381, *wR*2 = 0.0951	
Largest diff. peak and hole	0.295 and -0.540 e.Å^{-3}	

Linear Regression Modeling

A multivariate linear regression analysis on the experimental data obtained in the ligand structure studies was performed, using ligand parameters from the Kraken database.[10] Due to the limited spread in the product yield data, with only two ligands with yields above 30%, a comparable analysis regarding the ligand effects on yield was not feasible. Experimental measured regioselectivity was translated into $\Delta\Delta G^{\ddagger}$ using the Gibbs free energy equation ($\Delta\Delta G^{\ddagger} = -RT\ln(r.r.)$), to model the energy differential between the transition states of the two competing carbopalladation pathways (i.e., 1,2- vs. 2,1-carbopalladation). The collected experimental data was divided into three categories based on the observed 3-selectivity (<30%, 30-75%, >75% r.r.). Each of these categories was then randomly partitioned into a training set and a validation set, following a 75:25 ratio. To identify initial model candidates, an exhaustive search linear regression algorithm, coded and implemented using Python, was used. In this search both the parameters from the Kraken database and their reciprocal values were tested. The exhaustive search was limited to models using three parameters and a cross-term (i.e., a product or quotient of any of these three parameters). This code is included as a separate .txt file as part of the Supporting Information for this publication. The viability of the model candidates was then assessed based on their R^2 values, along with Q^2 values derived from a Leave-One-Out cross-validation analysis. Subsequently, the final model was obtained by iterative addition of a fourth parameter to the selected model candidates.

Computational Studies

Density functional theory (DFT) calculations were performed with Gaussian 16 package using University of Rochester's BlueHive computer cluster.[11] All calculations, including geometry optimizations, were performed in acetone solvent using the SMD solvent continuum.[12] Geometries were optimized using the B3LYP density functional, combined with Grimme's D3 empirical dispersion correction.[13] The LanL2DZ basis set was used on Pd, and a split-valence 6-31G(d,p) basis set was used for all other atoms.[14] When the substrates showed conformational freedom, conformational analysis was performed and only the most stable conformer of each stationary point was considered and reported, unless otherwise noted. Harmonic vibrational frequencies calculations were performed at the same level, to confirm the stationary points as true minima (0 imaginary frequencies) or transition states (1 imaginary frequencies) and to provide thermodynamic corrections to the SCF energies at 298 K. Single point electronic energies were computed at the ωB97X-D/6-311++G(2d,p)/SDD(Pd) level.[15] In all cases, Gibbs free energies for calculations using ωB97X-D/6-311++G(2d,p)/SDD(Pd) were approximated by summing the internal energy calculated with it and the thermal correction from the frequency calculation on the same structure with B3LYP-D3/6-31G(d,p)/LanL2DZ(Pd). Grid data for visualization of weak interactions between the ligand and diene using independent gradient model (IGM) analysis were generated using Multiwfn 3.8 and visualized using ChimeraX.[16]

Computational studies of the regioselectivity determining carbopalladation step with ligand L2 as a model ligand 3-selective ligand

Both “neutral” and “cationic” pathways were investigated. Computational studies were limited to the selectivity-determining carbopalladation step, starting from the oxidative addition complexes **I-Br** (“neutral” pathway) and **I** (“cationic pathway). It should be clarified that the “neutral” pathway is effectively an “anionic” pathway, and the “cationic” pathway is, in fact, “zwitterionic” due to the ready deprotonation of the sulfonamide under the reaction conditions. For each pathway, we accounted for the potential cis and trans orientation of the aryl group and the phosphine ligand. Initially, the **trans-I-Br** complex is formed in the oxidative addition step, where the halide and the aryl group adopt a cis arrangement to each other. Such complexes have been reported to easily isomerise into the form where the halide and aryl groups are in trans arrangement (**cis-I-Br**). Consistent with these findings, our calculations suggest that the **cis-I-Br** complex is energetically favored by about 10 kcal/mol. Given this strong energetic preference, further investigations into the “neutral” pathway focused solely on the “cis” orientation, henceforth referred to as the “cis-neutral” pathway. Conversely, for the “cationic” complex **I**, formed via halide dissociation from **I-Br**, both orientations were nearly isoenergetic, with only a slight preference for the cis isomer (1.1 kcal/mol). Therefore, both orientations were investigated in the context of the “cationic” pathway, starting from **cis-I** and **trans-I**, which we refer to as the “cis-cationic” and “trans-cationic” pathways respectively.

A direct comparison between the “neutral” and “cationic” pathways was not feasible due to the complexity involved in accurately calculating the free energy of the halide anion dissociation between the two pathways.[16] However, this did not ultimately pose a major issue as the experimental regioselectivity could only be reproduced with one out of the possible pathways. In the case of the “cis-neutral” pathway, the 3-selective 2,1-carbopalladation transition state **cis-TS1(3s)-Br** has a reasonable activation energy barrier of 15.8 kcal/mol. However, inconsistent with our experimental data, it is only weakly favored compared to the 2-selective 1,2-carbopalladation transition state **cis-TS1(2s)-Br** (0.6 kcal/mol, 69:31 r.r. at 100 °C).

The “cis-cationic” pathway also failed to align with the experimental data. In fact, the 2-selective transition state, **cis-TS1-2s**, is slightly preferred over **cis-TS1-3s** (0.8 kcal/mol, 25:75 r.r. at 100 °C). In contrast, the “trans-cationic” pathway ended up predicting the 3-selective transition state, **trans-TS1-3s**, to be significantly favored over **trans-TS1-2s** (4.2 kcal/mol, >99:1 r.r. at 100 °C). This is consistent with the observed 3-selectivity in our model reaction (92:8 r.r.). To evaluate and visualise the weak intermolecular interactions between the phosphine ligand and diene fragments in the transition states **trans-TS1-3s** and **trans-TS1-2s**, I carried out an independent gradient model (IGM) analysis of the molecular wavefunctions from single-point energy calculations.[15]

Figure S3. "Neutral" pathway with ligand **L2**.

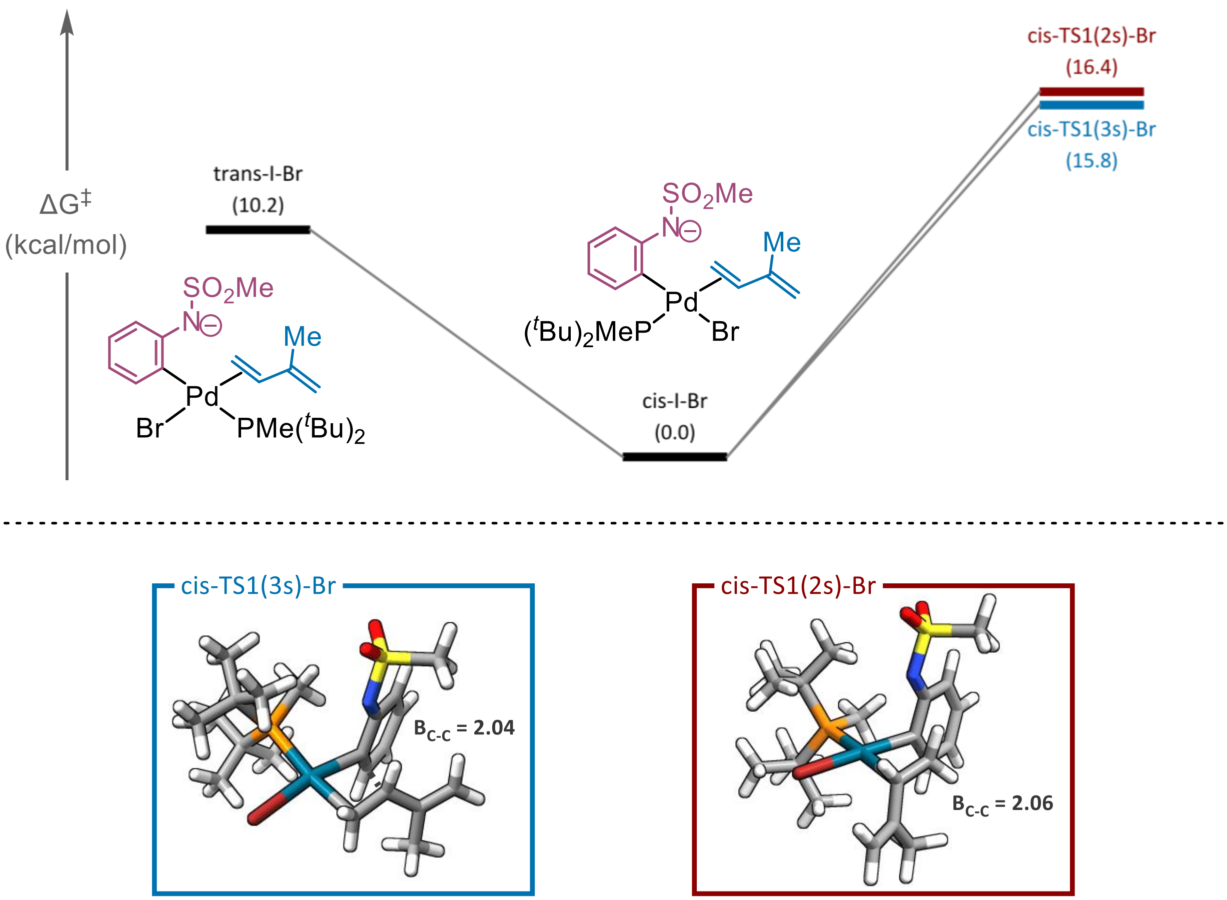

Figure S4. "Cationic" pathway with ligand **L2.**

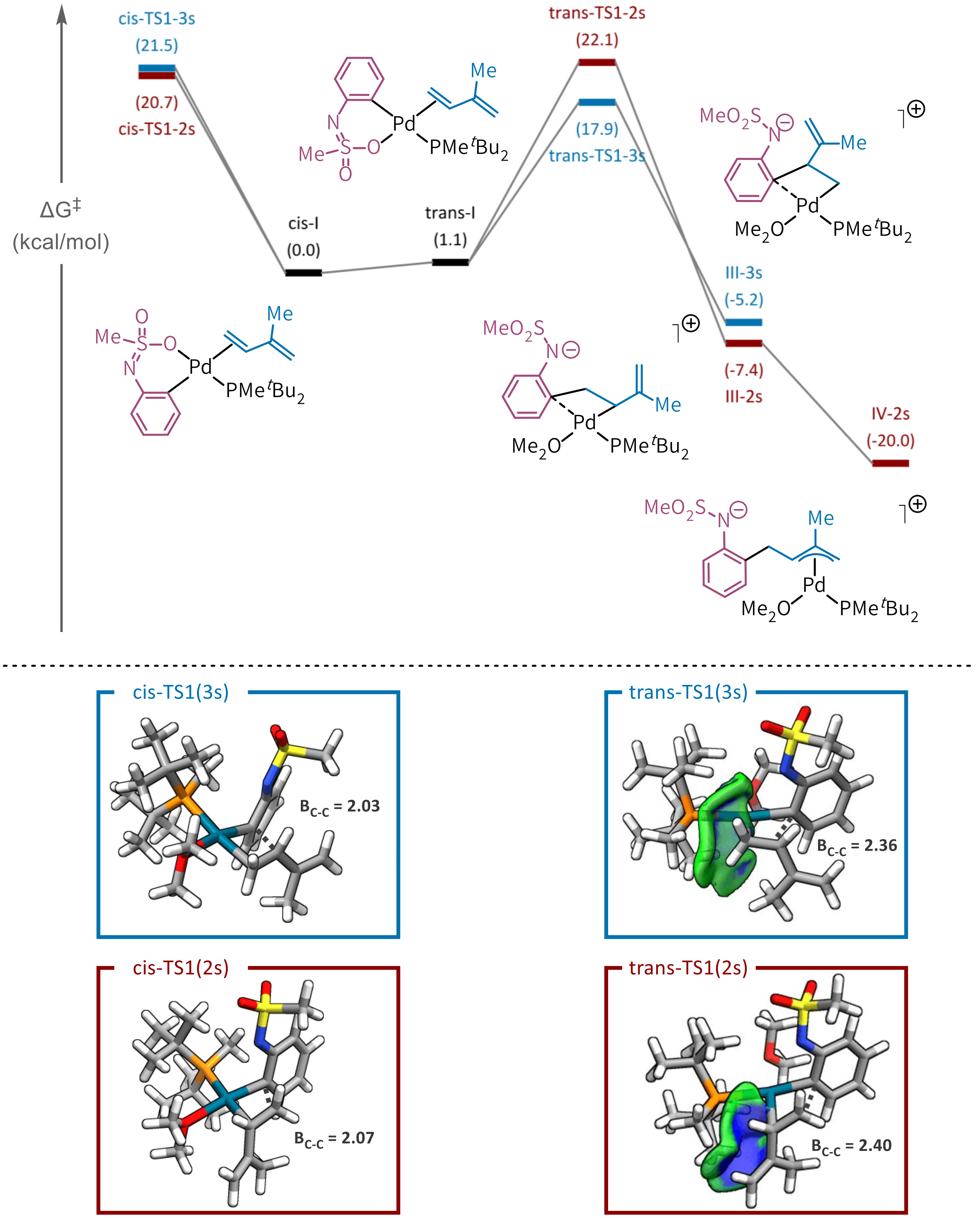

Continuing the exploration of the "trans-cationic" pathway, we modelled the carbopalladation transition states with two 2-selective ligands, **L13** and **L14**, which were included in the linear regression model. Both ligands led to 11:89 r.r. in the model reaction, equivalent to $\Delta\Delta G^{\ddagger}$ = 1.55 kcal/mol at 100 °C. While our calculations correctly predicted that the 2,1-carbopalladation is not significantly favored with **L13** and **L14,** they inaccurately suggested that the two transition states would be nearly isoenergetic, which is inconsistent with the experimentally observed regioselectivity. Specifically, with **L13**, the calculated $\Delta\Delta G^{\ddagger}$ was 0.0 kcal/mol, and with **L14**, the $\Delta\Delta G^{\ddagger}$ was slightly in favor of the 3-selective pathway, at 0.2 kcal/mol. Although the calculations in this instance were not accurate, they showed a certain level of precision, overestimating the 3-selective pathway by approximately 1.6 kcal/mol in both cases. If we apply this assumption to the calculations with **L2**, we could introduce a linear correction of 1.6 kcal/mol to the calculated $\Delta\Delta G^{\ddagger}$, giving us a corrected value of $\Delta\Delta G^{\ddagger}$ = 2.6 kcal/mol (equivalent to a 97:3 r.r. at 100 °C). Compared to the original 4.2 kcal/mol (>99:1 r.r. at 100 °C), this adjusted value offers a more accurate reflection of the experimentally observed regioselectivity with L4 (92:8 r.r., 1.8 kcal/mol at 100 °C).

Figure S5. “trans-Cationic” carbopalladation transition states with two 2-selective ligands.

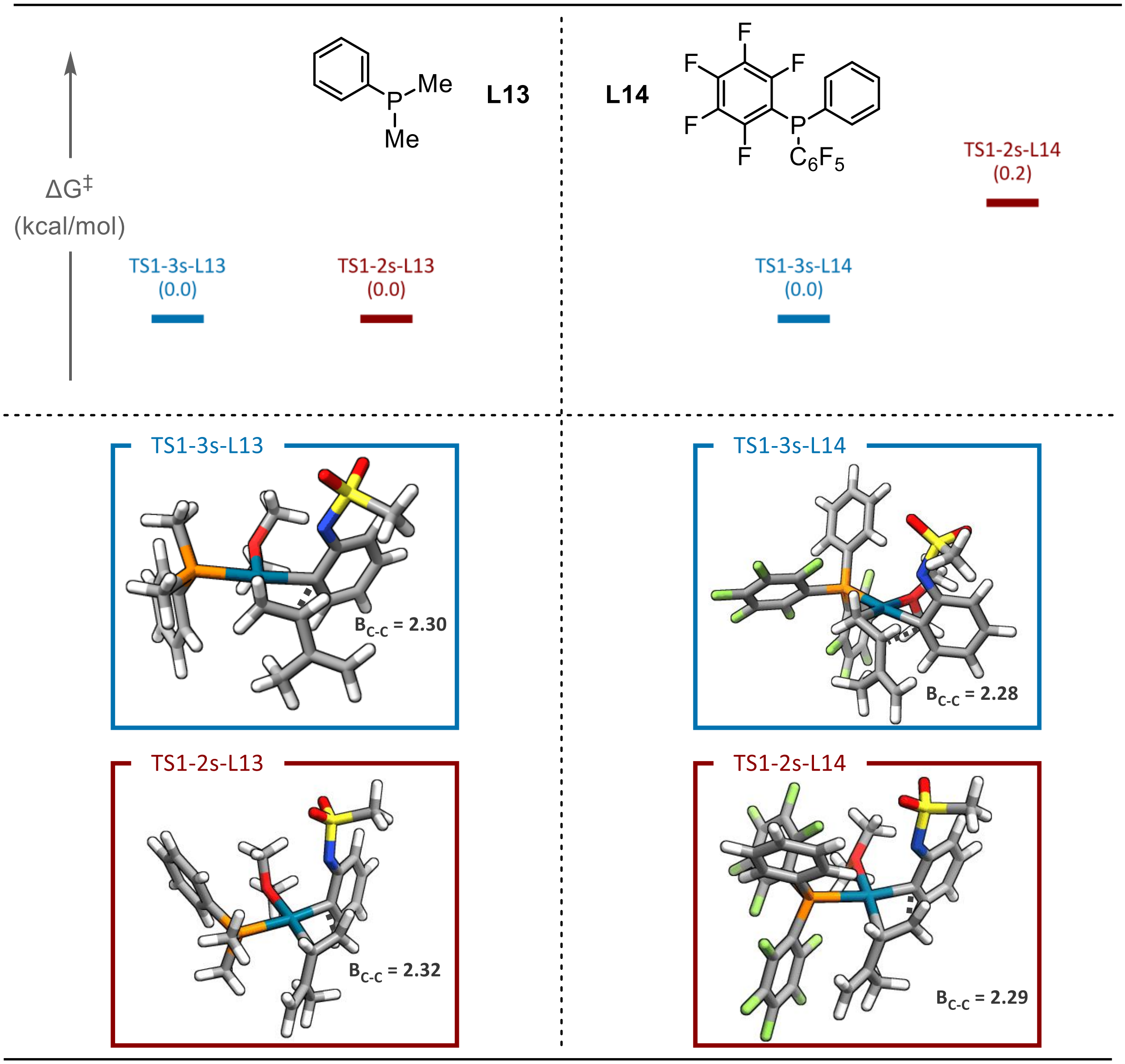

In the final part of the computational analysis, we investigated the carbopalladation step with ligand **L12**. This ligand is an example of the more sterically demanding ligands that demonstrated increased 2-selectivity in the model reaction compared to all other ligands and for which we hypothesised an alternative underligated, three-coordinate palladium complex was the active species during the regioselectivity-determining step. In the transition state optimisation calculations of a four-coordinate palladium complex with **L12** for both the “neutral” and “cationic” pathways - where the fourth ligand is either bromide or solvent - no stable stationary point could be localised. These calculations generally resulted in the expulsion of a “ligand” (diene, halide, or solvent), leading to the formation of an underligated, three-coordinate palladium complex. Starting from the underligated “zwitterionic” complex **V**, the 1,2-carbopalladation pathway is strongly

favoured (7.5 kcal/mol) compared to the 2,1-carbopalladation pathway. This preference primarily stems from the stabilisation of **TS2-2s** through weak coordination of the other olefinic portion of the diene to the underligated palladium centre.

Figure S6. "Cationic" carbopalladation pathway with ligand **L12.**

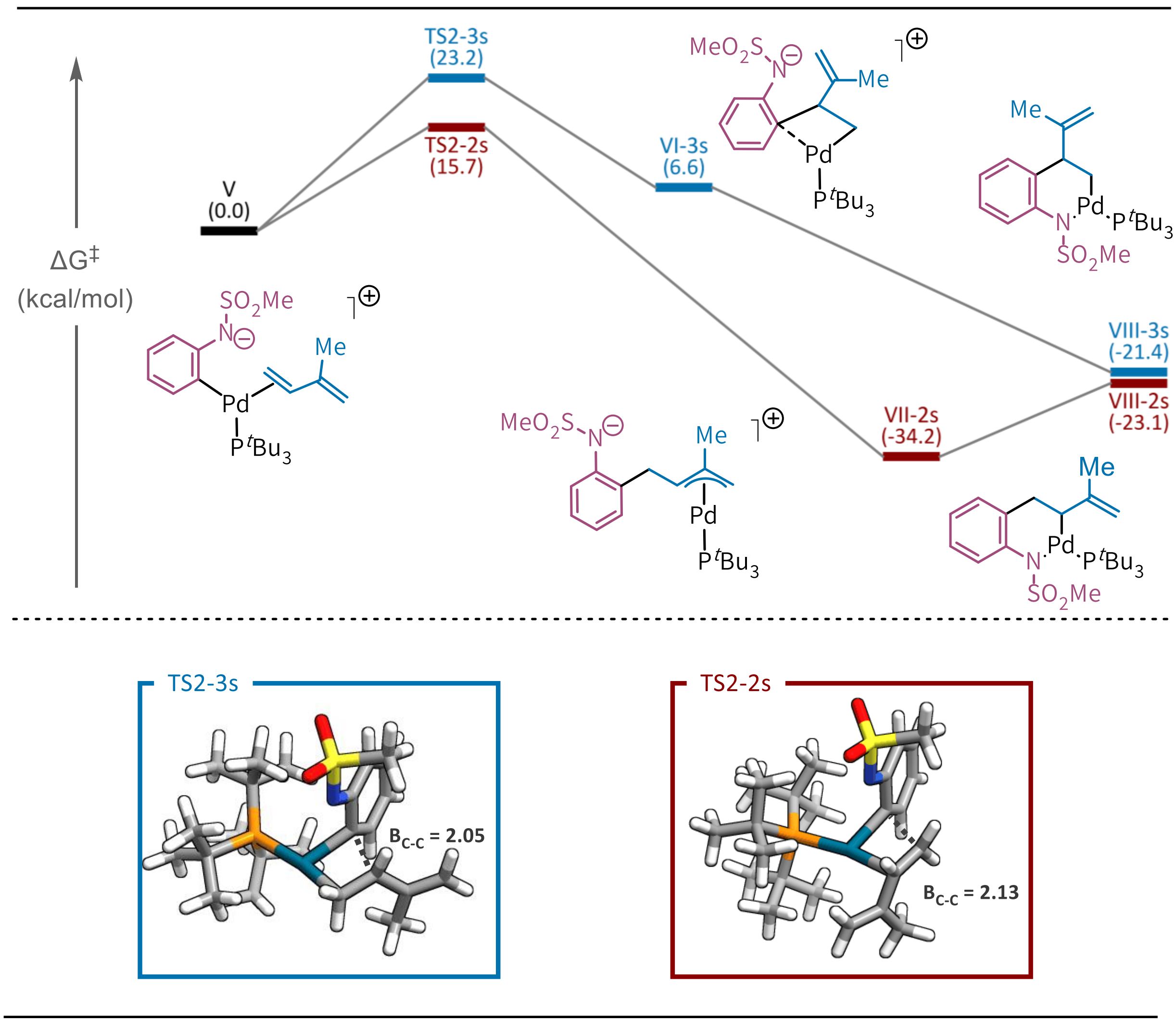

trans-I-Br trans open Br complex

		Hartree
Zero-point correction	=	0.535360
Thermal correction to Energy	=	0.570882
Thermal correction to Enthalpy	=	0.571826
Thermal correction to Gibbs Free Energy	=	0.468900
E(RwB97XD)	=	-4468.92953502
		cm^{-1}

Lowest energy vibration	=	16.61
Second lowest energy vibration	=	30.31

-1 1							
C	-3.93626	-1.26214	1.12431	H	4.858927	0.353014	0.815324
H	-2.96538	-1.61414	1.480512	H	3.452703	-3.51538	2.081266
H	-4.24775	-0.44464	1.776584	H	5.319565	-1.89471	1.703579
C	-3.8832	-0.84659	-0.35828	C	0.232658	-1.68046	-2.0344
C	-3.53036	-2.10516	-1.18225	H	-0.82373	-1.83916	-2.23304
H	-2.56024	-2.51781	-0.88563	C	0.745612	-0.41466	-2.12712
H	-3.5134	-1.91449	-2.25943	H	0.146258	0.415548	-2.48069
C	-5.26204	-0.32287	-0.7946	N	2.345428	1.295225	0.056126
H	-5.58974	0.520834	-0.18184	S	3.416718	2.321973	-0.54533
H	-5.28173	-0.01492	-1.84441	O	2.668278	3.484277	-1.08824
C	-1.53305	2.893385	0.020446	O	4.558665	2.664743	0.356514
H	-1.01856	2.885661	-0.94694	C	4.18401	1.512109	-1.97889
C	-3.48221	2.114866	1.388797	H	4.622977	0.561285	-1.66821
H	-4.49906	1.712384	1.393991	H	4.963845	2.169595	-2.37053
H	-2.86435	1.531138	2.074997	H	3.420251	1.341843	-2.74021
C	-3.84232	2.911087	-0.96189	H	1.805475	-0.22578	-2.02051
H	-4.79609	2.396277	-1.10104	C	1.028227	-2.90843	-1.85885
H	-3.40595	3.09546	-1.94736	C	0.388054	-4.07586	-1.66744
C	-2.88385	2.145022	-0.02895	H	-0.69685	-4.12562	-1.61134
P	-2.45691	0.386025	-0.65013	H	0.930746	-5.01082	-1.5581
C	-2.50442	0.634146	-2.48682	C	2.529429	-2.81852	-1.96381
H	-2.11821	-0.2464	-3.00286	H	2.935966	-2.11919	-1.22872
H	-1.86554	1.483498	-2.74192	H	2.821107	-2.45202	-2.95624
H	-0.86126	2.454177	0.763641	H	2.994605	-3.79496	-1.80519
Pd	-0.1807	-0.38906	0.152477	H	-1.70842	3.942275	0.292911
C	1.685599	-0.89238	0.784841	H	-4.05852	3.890426	-0.51594
C	2.725009	0.060161	0.586436	H	-3.53695	3.142793	1.771068
C	1.948112	-2.13968	1.338784	H	-6.00512	-1.12229	-0.6755
C	4.040335	-0.3494	0.932437	H	-4.66648	-2.07404	1.239139
C	3.257923	-2.52902	1.666628	H	-3.51229	0.838214	-2.85537
H	1.128475	-2.83655	1.499809	H	-4.28809	-2.87706	-0.99876
C	4.295903	-1.62203	1.453804	Br	-0.72776	-0.14913	2.61549

cis-I-Br

			Hartree
Zero-point correction	=		0.535669
Thermal correction to Energy	=		0.571359
Thermal correction to Enthalpy	=		0.572303
Thermal correction to Gibbs Free Energy	=		0.467233
E(RwB97XD)	=		-4468.94449557
			cm^{-1}
Lowest energy vibration	=		9.54
Second lowest energy vibration	=		19.98

-1 1							
C	-3.56285	-1.39377	1.102699	C	3.149768	1.001939	3.017378
H	-3.4635	-0.36941	0.736523	H	4.384021	0.254191	1.43127
H	-3.90202	-2.00993	0.269498	H	1.678351	1.695889	4.448249
C	-2.25188	-1.90826	1.725645	H	3.991267	1.095438	3.701077
C	-1.97267	-1.0612	2.985776	C	0.273212	2.665111	-1.18097
H	-1.90856	0.002849	2.741728	H	1.276186	2.250909	-1.22936
H	-1.05575	-1.35351	3.504463	C	-0.18678	3.24766	-0.03758
C	-2.41526	-3.38444	2.132823	H	0.454943	3.231804	0.838506
H	-2.66117	-4.02264	1.280806	N	2.428386	-0.03649	-0.53566
H	-1.52219	-3.78576	2.620451	S	3.822368	-0.47529	-1.19196
C	0.092992	-2.20662	-2.0425	O	3.528755	-1.00377	-2.5478
H	1.097465	-1.96757	-1.68249	O	4.686226	-1.34493	-0.33781
C	-2.22371	-3.01083	-1.525	C	4.774535	1.047691	-1.45319
H	-2.87079	-3.57949	-0.85201	H	4.919193	1.553954	-0.49601
H	-2.68964	-2.04968	-1.74987	H	5.743264	0.787879	-1.88728
C	-0.2397	-4.22417	-0.5927	H	4.216204	1.691536	-2.13589
H	-0.77131	-4.69697	0.237125	H	-0.29404	2.697555	-2.10526
H	0.824837	-4.18849	-0.34775	C	-1.43735	4.006286	0.121716
C	-0.80486	-2.83461	-0.95605	C	-1.75862	4.463239	1.346999
P	-0.76828	-1.64518	0.552376	H	-1.128	4.264239	2.210341
C	0.661059	-2.29036	1.523014	H	-2.65755	5.049513	1.518049
H	0.829542	-1.66411	2.397209	C	-2.28766	4.277557	-1.0908
H	1.549557	-2.23442	0.891708	H	-3.16465	4.878222	-0.83289
H	-0.35398	-1.28757	-2.43127	H	-1.71247	4.815313	-1.8551
Br	-2.52415	0.65052	-1.81664	H	-2.6201	3.33458	-1.53773
Pd	-0.54082	0.610771	-0.07606	H	0.500165	-3.32255	1.837522
C	1.008772	0.720123	1.234767	H	-2.80451	-1.19332	3.688346
C	2.321175	0.396996	0.781601	H	-3.24199	-3.46232	2.850245
C	0.799892	1.179666	2.53367	H	-4.34575	-1.41667	1.87151

C	3.378946	0.542454	1.718056	H	0.188152	-2.91925	-2.87274
C	1.86282	1.33361	3.439916	H	-0.34912	-4.87651	-1.46779
H	-0.20645	1.430087	2.859699	H	-2.15018	-3.57854	-2.4617

cis-TS1(3s)-Br

		Hartree
Zero-point correction	=	0.534325
Thermal correction to Energy	=	0.569210
Thermal correction to Enthalpy	=	0.570155
Thermal correction to Gibbs Free Energy	=	0.468759
E(RwB97XD)	=	-4468.91957528
		cm^{-1}
Lowest energy vibration	=	-387.87
Second lowest energy vibration	=	21.10

-1 1							
C	3.883258	-0.59917	1.388501	C	-2.80196	0.257382	2.884877
H	3.378162	-1.4513	0.92744	H	-3.4559	1.808892	1.569373
H	4.535242	-0.16033	0.631427	H	-1.93708	-1.36289	4.044692
C	2.878969	0.418193	1.962157	H	-3.38924	0.646126	3.714142
C	2.097522	-0.28169	3.097329	C	-0.72848	-2.51962	-1.31608
H	1.51856	-1.13236	2.724205	H	-0.52827	-2.21643	-2.34186
H	1.412919	0.394549	3.616962	C	-1.93745	-2.03747	-0.72387
C	3.64077	1.620362	2.546196	H	-2.42502	-1.23924	-1.27833
H	4.272446	2.107724	1.799331	N	-2.04286	1.028641	-0.70443
H	2.970576	2.372136	2.97391	S	-2.85658	2.369498	-1.08212
C	1.30866	2.081699	-1.83868	O	-2.44649	2.756395	-2.45233
H	0.275498	2.288999	-1.54331	O	-2.78022	3.445227	-0.052
C	3.678206	1.679497	-1.1377	C	-4.60253	1.89521	-1.19144
H	4.446875	1.810082	-0.37078	H	-4.93431	1.500422	-0.22886
H	3.674777	0.636935	-1.46595	H	-5.18768	2.780059	-1.45376
C	2.368504	3.564619	-0.11667	H	-4.70307	1.132356	-1.96611
H	2.987127	3.653566	0.779883	H	-0.36359	-3.511	-1.05765
H	1.381005	3.978353	0.105907	C	-2.88028	-2.91263	0.023981
C	2.288235	2.118491	-0.64443	C	-4.16103	-2.5301	0.150258
P	1.579669	0.891188	0.643733	H	-4.51308	-1.58329	-0.25227
C	0.424874	1.968034	1.601786	H	-4.89227	-3.14984	0.662766
H	-0.14636	1.354181	2.297829	C	-2.37757	-4.22772	0.56124
H	-0.28223	2.43321	0.912323	H	-3.1299	-4.71196	1.189734

H	1.328689	1.103148	-2.32832	H	-2.12582	-4.91153	-0.25887
Br	2.462909	-1.83634	-1.83305	H	-1.46502	-4.09908	1.15248
Pd	0.474325	-1.02231	-0.37831	H	0.951535	2.746684	2.158339
C	-1.32113	-0.73724	0.727489	H	2.810461	-0.66158	3.839925
C	-2.10545	0.452955	0.540523	H	4.298007	1.272406	3.354144
C	-1.27888	-1.36533	1.988816	H	4.5218	-0.96687	2.202718
C	-2.8638	0.905976	1.657237	H	1.608324	2.840619	-2.57395
C	-1.99185	-0.87402	3.076078	H	2.818416	4.198559	-0.89171
H	-0.68398	-2.26663	2.104908	H	3.963949	2.305518	-1.99369

cis-TS1(2s)-Br

		Hartree
Zero-point correction	=	0.534928
Thermal correction to Energy	=	0.569668
Thermal correction to Enthalpy	=	0.570612
Thermal correction to Gibbs Free Energy	=	0.470257
E(RwB97XD)	=	-4468.92057687
		cm^{-1}
Lowest energy vibration	=	-347.72
Second lowest energy vibration	=	23.67

-1 1							
C	1.089755	-2.3864	-2.27921	C	-2.90625	-0.21582	3.134904
H	0.752473	-1.3807	-2.53939	H	-4.11572	-0.82517	1.473796
H	2.181037	-2.37681	-2.29889	H	-1.49957	0.491792	4.626527
C	0.525563	-2.83641	-0.91993	H	-3.59898	-0.63519	3.86142
C	-1.01543	-2.82073	-1.01077	C	0.214514	2.945271	-0.37244
H	-1.40903	-1.81166	-1.15692	H	0.1495	2.915417	-1.45788
H	-1.49049	-3.24354	-0.1206	C	-0.97148	2.673816	0.352129
C	0.991649	-4.27108	-0.61367	H	-1.87245	2.499501	-0.22751
H	2.080003	-4.36672	-0.62277	N	-2.57397	0.24662	-0.58233
H	0.620722	-4.63352	0.349915	S	-3.96762	-0.24481	-1.23063
C	3.159915	-0.5462	1.82101	O	-3.8439	-0.09202	-2.69945
H	2.439784	-0.32027	2.615832	O	-4.44478	-1.57562	-0.75518
C	3.784727	-2.01307	-0.10977	C	-5.22717	0.948665	-0.705
H	3.650737	-2.9698	-0.62144	H	-5.27557	0.97262	0.385655
H	3.696315	-1.20459	-0.83854	H	-6.19083	0.636019	-1.11433
C	2.927235	-3.03365	2.015813	H	-4.95401	1.932967	-1.09041

H	2.614114	-3.97681	1.562723	H	-1.11641	3.161358	1.308114
H	2.359068	-2.89208	2.939119	C	1.328089	3.739082	0.17797
C	2.792867	-1.83571	1.053562	C	2.155406	4.405008	-0.64842
P	0.994872	-1.57691	0.435103	H	2.043126	4.349549	-1.72689
C	0.017144	-2.17218	1.881823	H	2.975802	5.007801	-0.26682
H	-1.0343	-1.92961	1.733542	C	1.52098	3.783057	1.676152
H	0.355736	-1.65057	2.778098	H	1.726662	2.779131	2.066721
H	3.207958	0.311642	1.144025	H	0.630017	4.149989	2.199358
Br	2.457331	0.962815	-1.83641	H	2.358906	4.431775	1.947002
Pd	0.559914	0.753503	-0.0832	H	4.144193	-0.67099	2.291203
C	-1.12291	0.814933	1.233866	H	3.982734	-3.13855	2.297852
C	-2.34899	0.223061	0.780135	H	4.806272	-2.00055	0.29337
C	-0.85472	0.916637	2.608105	H	0.744531	-3.08307	-3.05455
C	-3.21201	-0.30997	1.778338	H	0.597546	-4.94342	-1.38693
C	-1.73081	0.409136	3.567917	H	-1.32578	-3.4324	-1.86739
H	0.056033	1.416182	2.928157	H	0.124751	-3.24872	2.032039

I Migratory insertion chelate complex

		Hartree
Zero-point correction	=	0.535085
Thermal correction to Energy	=	0.568142
Thermal correction to Enthalpy	=	0.569086
Thermal correction to Gibbs Free Energy	=	0.473531
E(RwB97XD)	=	-1894.58037353
		cm^{-1}
Lowest energy vibration	=	25.76
Second lowest energy vibration	=	31.31

0 1							
C	2.434619	0.840755	-2.44596	H	-4.85449	1.640016	0.172863
H	1.350735	0.725442	-2.53533	H	-4.75676	-1.66772	-2.58987
H	2.628009	1.798149	-1.95958	H	-6.04506	0.098681	-1.37845
C	3.075175	-0.34553	-1.69745	N	-2.33622	1.633022	0.811073
C	2.742414	-1.63777	-2.47703	S	-1.09159	2.441425	0.291218
H	1.663641	-1.82987	-2.50292	O	-0.39786	3.121537	1.404781
H	3.241438	-2.51723	-2.05973	O	-0.14558	1.615255	-0.61412
C	4.600813	-0.16323	-1.64215	C	-1.62997	3.716778	-0.87056
H	4.882018	0.785555	-1.1784	H	-2.19685	3.234466	-1.66989
H	5.097716	-0.97388	-1.10049	H	-0.75014	4.220079	-1.27641
C	1.891176	0.644452	2.462313	H	-2.26146	4.422199	-0.32703

H	1.794188	-0.37047	2.863574	H	2.244959	1.286873	3.278733
C	2.8964	2.143673	0.717901	H	4.607042	1.157273	2.53282
H	3.672964	2.276006	-0.04045	H	3.10817	2.849986	1.530731
H	1.926997	2.405136	0.29287	H	4.99852	-0.16064	-2.66512
C	4.305234	0.378061	1.821664	H	2.855835	0.888729	-3.458
H	5.05644	0.34724	1.029116	H	4.039068	-2.17446	0.542196
H	4.330791	-0.57552	2.356336	H	3.084962	-1.52581	-3.51285
C	2.897987	0.715884	1.292245	C	-0.54023	-2.29745	1.095215
P	2.263072	-0.54048	0.010132	H	0.362451	-2.19821	1.694855
C	2.952369	-2.13245	0.647339	C	-0.41521	-2.72176	-0.22051
H	2.518363	-2.97909	0.113407	H	0.53402	-3.08785	-0.59525
H	2.702283	-2.23297	1.706463	H	-1.29442	-3.01632	-0.78111
H	0.904098	1.004314	2.162622	C	-1.79625	-2.19851	1.870412
Pd	-0.21927	-0.51296	-0.29882	C	-2.82812	-3.02188	1.628598
C	-2.22044	-0.32957	-0.72326	H	-2.79302	-3.77369	0.845747
C	-2.93237	0.722141	-0.09645	H	-3.7358	-2.97211	2.224116
C	-2.88804	-1.15513	-1.63379	C	-1.8045	-1.16799	2.970865
C	-4.31455	0.845238	-0.33632	H	-1.75627	-0.15848	2.545728
C	-4.25996	-1.00832	-1.88244	H	-0.93091	-1.28817	3.623909
H	-2.35024	-1.93882	-2.16112	H	-2.70765	-1.24256	3.582809
C	-4.97736	-0.01762	-1.20987				

I-OMe₂ Migratory insertion chelate complex with OMe_2 instead of isoprene

		Hartree
Zero-point correction	=	0.501394
Thermal correction to Energy	=	0.533109
Thermal correction to Enthalpy	=	0.534053
Thermal correction to Gibbs Free Energy	=	0.440633
E(RwB97XD)	=	-1854.293222

Lowest energy vibration	=	20.18
Second lowest energy vibration	=	37.15

0 1							
C	-2.30912	0.836724	2.436754	C	5.178123	-0.75268	0.217585
H	-1.22333	0.7212	2.492303	H	5.126645	1.235953	-0.62804
H	-2.51843	1.832831	2.043226	H	4.903457	-2.70996	1.095872
C	-2.95952	-0.28083	1.596278	H	6.263867	-0.80935	0.211795
C	-2.6511	-1.62956	2.284825	N	2.575673	1.68629	-0.8034

H	-1.57421	-1.83376	2.308706	S	1.4418	2.414238	0.000148
H	-3.15362	-2.47165	1.800323	O	0.71951	3.383908	-0.84819
C	-4.48145	-0.07408	1.538856	O	0.495002	1.454532	0.779217
H	-4.74356	0.911231	1.144864	C	2.156599	3.330806	1.38288
H	-4.98029	-0.83448	0.929766	H	2.737062	2.633752	1.991206
C	-1.68814	0.996023	-2.46077	H	1.350137	3.772663	1.971389
H	-1.62081	0.012904	-2.94002	H	2.803059	4.107678	0.970058
C	-2.62301	2.383938	-0.5991	H	-2.0137	1.713377	-3.22484
H	-3.3962	2.497048	0.16572	H	-4.37513	1.62823	-2.50179
H	-1.64168	2.556184	-0.15198	H	-2.78789	3.167548	-1.34976
C	-4.11386	0.78456	-1.8504	H	-4.89418	-0.14301	2.553709
H	-4.87211	0.727273	-1.06616	H	-2.71163	0.792831	3.456709
H	-4.17764	-0.1244	-2.45567	H	-3.92583	-1.89745	-0.85986
C	-2.69731	1.01103	-1.28999	H	-3.00369	-1.58924	3.322602
P	-2.12568	-0.36831	-0.10945	O	0.225811	-2.51485	-0.79299
C	-2.83319	-1.88947	-0.88078	C	0.997491	-2.75308	-1.98407
H	-2.45941	-2.77281	-0.36301	H	2.045466	-2.94674	-1.73309
H	-2.49399	-1.94233	-1.91808	H	0.573467	-3.60964	-2.52057
H	-0.6886	1.292438	-2.12968	H	0.922696	-1.85682	-2.60071
Pd	0.339579	-0.50921	0.069227	C	0.11642	-3.67952	0.042186
C	2.369517	-0.59143	0.197498	H	-0.41905	-4.46405	-0.50456
C	3.13893	0.506721	-0.26008	H	1.105016	-4.05023	0.33305
C	3.019844	-1.72442	0.694777	H	-0.44908	-3.394	0.929204
C	4.543511	0.397585	-0.25433				
C	4.419437	-1.81648	0.709415				
H	2.437669	-2.56061	1.072563				

II Migratory insertion open complex with OMe_2 instead of isoprene

		Hartree
Zero-point correction	=	0.617088
Thermal correction to Energy	=	0.655970
Thermal correction to Enthalpy	=	0.656914
Thermal correction to Gibbs Free Energy	=	0.547401
E(RwB97XD)	=	-2049.610157
		cm^{-1}
Lowest energy vibration	=	17.12
Second lowest energy vibration	=	30.31

0 1							
C	3.60637	0.84271	1.734315	H	1.395682	2.063708	-1.84663
H	2.713514	1.46851	1.796536	C	-0.35062	0.868993	-2.10956
H	3.449989	-0.03828	2.359475	H	-1.42848	0.854932	-2.22283
C	3.926509	0.494152	0.26909	N	-2.18685	-1.09948	-0.2546
C	4.042709	1.821029	-0.51299	S	-3.14407	-2.3421	-0.59641
H	3.106708	2.389253	-0.4927	O	-2.29363	-3.41281	-1.17151
H	4.336522	1.669738	-1.55556	O	-4.0515	-2.76073	0.511947
C	5.268637	-0.25164	0.190687	C	-4.23587	-1.79447	-1.9367
H	5.281586	-1.1391	0.82804	H	-4.80715	-0.92402	-1.60624
H	5.517754	-0.55363	-0.83118	H	-4.91568	-2.61164	-2.18993
C	1.130416	-2.87647	-0.49102	H	-3.62113	-1.53285	-2.80042
H	0.990483	-2.7799	-1.57281	H	0.183402	0.045949	-2.57187
C	2.624868	-2.48291	1.494312	C	-0.31737	3.35799	-1.56877
H	3.634686	-2.248	1.842032	C	0.432496	4.36455	-1.08465
H	1.92055	-1.85129	2.039202	H	1.473519	4.214104	-0.80848
C	3.608021	-3.11121	-0.73384	H	0.024821	5.3626	-0.9505
H	4.608636	-2.76432	-0.47068	C	-1.7506	3.547777	-1.99464
H	3.510208	-3.08474	-1.82285	H	-2.41435	2.842743	-1.4884
C	2.493576	-2.31341	-0.03034	H	-1.85137	3.372609	-3.07303
P	2.460559	-0.46167	-0.48342	H	-2.09409	4.562004	-1.77489
C	2.857155	-0.49425	-2.28992	H	1.084282	-3.94611	-0.25116
H	2.654869	0.478828	-2.74227	H	3.532745	-4.16226	-0.4268
H	2.220931	-1.23436	-2.78152	H	2.420577	-3.52755	1.760048
H	0.279113	-2.38887	-0.0111	H	6.068189	0.414305	0.539884
Pd	0.171455	0.524364	0.010474	H	4.442592	1.411225	2.160238
C	-1.70697	1.098244	0.495872	H	3.902388	-0.75476	-2.47437
C	-2.66617	0.062446	0.340279	H	4.813242	2.443121	-0.04128
C	-2.01568	2.314128	1.08324	O	0.314858	-0.02517	2.241894
C	-3.97168	0.331656	0.827631	C	-0.54716	-1.08173	2.702975
C	-3.31865	2.564475	1.55073	H	-0.18634	-1.44487	3.672758
H	-1.25421	3.087226	1.16679	H	-1.577	-0.72263	2.799301
C	-4.28142	1.563629	1.413547	H	-0.51295	-1.883	1.968055
H	-4.73027	-0.44179	0.776419	C	0.318704	1.070478	3.171514
H	-3.56574	3.520017	2.005963	H	-0.69903	1.435171	3.34483
H	-5.29403	1.733755	1.773529	H	0.762342	0.741573	4.119114
C	0.313194	2.03953	-1.76403	H	0.919529	1.872304	2.744361

trans-TS1-3s

		Hartree
Zero-point correction	=	0.616603
Thermal correction to Energy	=	0.654770
Thermal correction to Enthalpy	=	0.655714
Thermal correction to Gibbs Free Energy	=	0.548119
E(RwB97XD)	=	-2049.603842
		cm^{-1}
Lowest energy vibration	=	-118.30
Second lowest energy vibration	=	19.00

0 1							
C	1.705978	2.536059	1.080195	H	-0.08634	0.250871	-2.52889
H	0.67149	2.190042	1.013475	C	-1.21869	-1.47207	-1.95358
H	2.170913	2.069778	1.951227	H	-2.13858	-0.90122	-2.0211
C	2.457606	2.248721	-0.23344	N	-2.16476	1.135175	-0.30024
C	1.622417	2.844495	-1.38892	S	-3.07244	2.464358	-0.35276
H	0.643761	2.361987	-1.47749	O	-2.33328	3.465929	-1.1581
H	2.136778	2.781443	-2.35205	O	-3.568	2.932152	0.973783
C	3.838756	2.923	-0.21088	C	-4.55191	2.042928	-1.31113
H	4.427534	2.624803	0.660236	H	-5.07975	1.220011	-0.82428
H	4.420098	2.709062	-1.11316	H	-5.19719	2.923458	-1.3579
C	3.681886	-1.99477	0.293589	H	-4.24196	1.747722	-2.31557
H	3.702642	-2.31771	-0.75289	H	0.840906	-1.33654	-2.56252
C	3.885	-0.08728	1.924417	C	-1.34197	-2.93364	-2.0557
H	4.205634	0.94624	2.081555	C	-2.5561	-3.46089	-2.29054
H	2.882806	-0.19967	2.342066	H	-3.4409	-2.83416	-2.36869
C	5.340505	-0.17454	-0.12655	H	-2.69567	-4.53093	-2.41722
H	5.589465	0.887299	-0.09218	C	-0.10331	-3.78072	-1.91715
H	5.455896	-0.52174	-1.15714	H	-0.35034	-4.84511	-1.93726
C	3.938236	-0.4779	0.436097	H	0.612304	-3.5823	-2.72383
P	2.523374	0.36678	-0.52019	H	0.411433	-3.55752	-0.97472
C	3.097257	0.22624	-2.26935	H	3.702507	4.010655	-0.15847
H	2.305408	0.538665	-2.95117	H	1.444163	3.906474	-1.18053
H	3.342274	-0.81665	-2.48587	H	1.683051	3.619633	1.250615
H	2.717215	-2.2883	0.715105	H	4.567262	-0.73294	2.491415
Pd	0.311859	-0.57338	-0.09595	H	6.083402	-0.7068	0.481007
C	-1.66752	-1.10601	0.337332	H	4.466972	-2.54638	0.825354
C	-2.54113	0.01572	0.422403	H	3.981153	0.841813	-2.4525
C	-1.94848	-2.31721	0.957656	O	0.523105	-0.45419	2.261709
C	-3.68689	-0.14967	1.248297	C	-0.42977	0.35518	2.966769
C	-3.08435	-2.45371	1.766358	H	-1.37658	-0.18037	3.094494
H	-1.27798	-3.16215	0.81951	H	-0.02387	0.626874	3.949545

C	-3.94228	-1.35673	1.900276	H	-0.60585	1.254824	2.380112
H	-4.35931	0.687677	1.396759	C	0.731888	-1.69988	2.939257
H	-3.2914	-3.39204	2.273593	H	1.12724	-1.51522	3.946353
H	-4.83117	-1.43895	2.522053	H	-0.20424	-2.26456	3.013188
C	-0.01182	-0.77857	-2.18622	H	1.458596	-2.27422	2.365565

trans-TS1-2s

		Hartree
Zero-point correction	=	0.617482
Thermal correction to Energy	=	0.655386
Thermal correction to Enthalpy	=	0.656330
Thermal correction to Gibbs Free Energy	=	0.549926
E(RwB97XD)	=	-2049.59970347
		cm^{-1}
Lowest energy vibration	=	-61.24
Second lowest energy vibration	=	14.33

0 1							
C	3.041315	0.057847	2.324531	C	-0.63099	1.918354	-1.82066
H	2.284326	0.844973	2.27526	H	-1.31935	1.223451	-2.28979
H	2.57323	-0.83995	2.732325	N	-2.55211	-0.43391	-0.7422
C	3.679209	-0.16879	0.941892	S	-3.64725	-1.59933	-0.90015
C	4.209058	1.191485	0.437981	O	-3.03068	-2.67879	-1.70946
H	3.400588	1.907839	0.266552	O	-4.28598	-2.02506	0.378991
H	4.785776	1.09742	-0.48624	C	-4.98688	-0.92648	-1.92039
C	4.859283	-1.14764	1.058499	H	-5.41683	-0.05116	-1.42867
H	4.565949	-2.09604	1.514193	H	-5.75135	-1.69745	-2.04529
H	5.320791	-1.3561	0.088503	H	-4.57344	-0.64437	-2.89088
C	0.681525	-2.82096	-0.98368	H	-1.03315	2.879416	-1.53093
H	0.803144	-2.52483	-2.03164	C	1.720544	2.847047	-1.77206
C	1.754042	-3.02479	1.290873	C	2.795879	2.940213	-2.5735
H	2.694872	-3.06067	1.847187	H	2.97282	2.232981	-3.37896
H	1.075154	-2.35391	1.815836	H	3.516112	3.745779	-2.46028
C	3.084061	-3.47626	-0.788	C	1.464605	3.841962	-0.66803
H	4.054801	-3.33262	-0.30993	H	1.437441	3.337784	0.305231
H	3.199888	-3.30496	-1.8616	H	0.499184	4.345325	-0.79198
C	1.976669	-2.59863	-0.17175	H	2.244505	4.607455	-0.64071
P	2.332443	-0.72456	-0.28579	H	0.424808	-3.88756	-0.97083
C	3.182777	-0.60668	-1.91694	H	2.804588	-4.52926	-0.65608
H	3.351504	0.43799	-2.17138	H	1.323216	-4.03355	1.310211
H	2.547726	-1.05405	-2.68576	H	5.63025	-0.70006	1.698653

H	-0.16921	-2.26655	-0.58317	H	3.815826	0.383665	3.029996
Pd	0.214486	0.585058	-0.07452	H	4.144399	-1.12521	-1.90667
C	-1.60986	1.47705	0.326098	H	4.874366	1.614829	1.200386
C	-2.73938	0.6417	0.111026	O	-0.30659	-0.51136	2.034327
C	-1.66654	2.64025	1.082231	C	-1.33834	-1.51109	2.075097
C	-3.91675	1.007333	0.817056	H	-1.2203	-2.12029	2.981064
C	-2.85265	2.989984	1.747504	H	-2.33066	-1.05325	2.062272
H	-0.78763	3.273127	1.173615	H	-1.23051	-2.13696	1.193629
C	-3.9639	2.155556	1.612224	C	-0.39267	0.348908	3.176064
H	-4.78922	0.366384	0.762195	H	-1.38545	0.806648	3.247684
H	-2.89649	3.888785	2.356496	H	-0.18597	-0.22523	4.08871
H	-4.89032	2.398924	2.127819	H	0.353365	1.134625	3.065069
C	0.743045	1.760009	-2.00109				
H	1.070756	0.952183	-2.65039				

TS1-3s-Isoprene

		Hartree
Zero-point correction	=	0.650356
Thermal correction to Energy	=	0.689945
Thermal correction to Enthalpy	=	0.690890
Thermal correction to Gibbs Free Energy	=	0.580967
E(RwB97XD)	=	-2089.86930291
		cm^{-1}
Lowest energy vibration	=	-365.31
Second lowest energy vibration	=	20.23

0 1							
C	-3.73775	-0.58377	1.441849	H	2.254798	1.877133	-0.94523
H	-3.39114	0.437752	1.264812	N	2.222286	-0.57051	-0.78929
H	-4.2369	-0.94494	0.539099	S	3.384808	-1.56969	-1.31814
C	-2.57319	-1.48831	1.880861	O	3.038533	-1.92926	-2.71162
C	-1.92099	-0.83745	3.120746	O	3.668377	-2.70593	-0.39807
H	-1.46374	0.1269	2.883011	C	4.901611	-0.58563	-1.40385
H	-1.15849	-1.47495	3.575995	H	5.142458	-0.19891	-0.41147
C	-3.11731	-2.87029	2.279541	H	5.709404	-1.2292	-1.76059
H	-3.69953	-3.33129	1.478119	H	4.738612	0.238489	-2.10102
H	-2.32195	-3.56155	2.57342	H	-0.41165	3.367325	-0.65517
C	-0.61281	-2.60592	-1.96549	C	2.089837	3.411939	0.575094
H	0.413356	-2.75458	-1.61898	C	3.408979	3.372995	0.828712
C	-3.06875	-2.59581	-1.38931	H	4.051757	2.621187	0.378268

H	-3.83567	-2.87205	-0.66071	H	3.881254	4.090861	1.493891
H	-3.24451	-1.55956	-1.68728	C	1.182199	4.450977	1.181396
C	-1.51909	-4.2867	-0.36959	H	1.710178	5.047918	1.929568
H	-2.1973	-4.53521	0.44761	H	0.797919	5.129797	0.410091
H	-0.49948	-4.52822	-0.05751	H	0.310133	3.992785	1.659922
C	-1.64865	-2.82342	-0.83982	H	-0.1526	-3.25606	1.898329
P	-1.23536	-1.53442	0.511461	H	-2.69717	-0.66233	3.876038
C	0.171734	-2.34837	1.384076	H	-3.78468	-2.75332	3.143227
H	0.595384	-1.6602	2.114202	H	-4.48907	-0.54676	2.240699
H	0.953015	-2.59759	0.664009	H	-0.80134	-3.32917	-2.76909
H	-0.66487	-1.60408	-2.396	H	-1.76448	-4.94626	-1.21188
Pd	-0.62281	0.711349	-0.39038	H	-3.2176	-3.2235	-2.27684
C	1.091804	0.772092	0.818244	C	-2.08626	0.885653	-2.46416
C	2.172989	-0.1251	0.500924	H	-2.01912	-0.16977	-2.69487
C	0.898037	1.228664	2.140298	C	-2.93567	1.339008	-1.50448
C	3.073129	-0.43526	1.563198	H	-3.50035	0.612676	-0.9297
C	1.762988	0.867057	3.160453	H	-1.56655	1.56016	-3.13662
H	0.071937	1.901363	2.347554	C	-3.28456	2.751282	-1.26162
C	2.866174	0.049899	2.84594	C	-4.06482	3.063413	-0.21095
H	3.902375	-1.10754	1.380341	H	-4.42565	2.303115	0.476467
H	1.604879	1.219969	4.175201	H	-4.36992	4.088033	-0.01631
H	3.571446	-0.22115	3.628302	C	-2.82451	3.792387	-2.25196
C	0.242376	2.569945	-1.00061	H	-3.13648	4.793806	-1.94394
H	0.240554	2.403755	-2.07704	H	-1.73802	3.794926	-2.37398
C	1.515933	2.423595	-0.36507	H	-3.25016	3.587283	-3.24229

TS1-2s-Isoprene

		Hartree
Zero-point correction	=	0.651358
Thermal correction to Energy	=	0.690850
Thermal correction to Enthalpy	=	0.691794
Thermal correction to Gibbs Free Energy	=	0.582725
E(RwB97XD)	=	-2089.86173232
		cm^{-1}
Lowest energy vibration	=	-303.97
Second lowest energy vibration	=	19.45

0 1							
C	-0.82216	2.824557	-2.34479	H	1.321827	-2.86005	-0.52987
H	-1.08671	1.813966	-2.66339	N	2.556431	-0.91975	-0.54278
H	-1.71685	3.314761	-1.95849	S	4.022533	-0.48491	-1.0517

C	0.34387	2.814103	-1.34025	O	3.951574	-0.33628	-2.52373
C	1.543574	2.114751	-2.0157	O	4.599095	0.667667	-0.30031
H	1.353136	1.056228	-2.20961	C	5.133856	-1.8868	-0.75694
H	2.458465	2.176936	-1.42315	H	5.155091	-2.12794	0.307522
C	0.739772	4.263369	-0.99885	H	6.134283	-1.60895	-1.0976
H	-0.08294	4.829672	-0.55654	H	4.765032	-2.74143	-1.32771
H	1.595145	4.311182	-0.31963	H	0.387928	-3.52866	0.914121
C	-1.99732	1.589707	2.211062	C	-2.04782	-3.52902	-0.33332
H	-1.26021	0.998349	2.764097	C	-2.83197	-4.1142	-1.25741
C	-2.3945	3.486459	0.601179	H	-2.5815	-4.08875	-2.31515
H	-1.98043	4.380738	0.129822	H	-3.74107	-4.64005	-0.97928
H	-2.88946	2.900144	-0.1703	C	-2.39792	-3.54315	1.134418
C	-0.62456	3.679174	2.332562	H	-2.54649	-2.51897	1.49843
H	-0.06105	4.451709	1.802203	H	-1.59828	-3.98124	1.742115
H	0.048709	3.175473	3.030428	H	-3.31304	-4.11197	1.319077
C	-1.32312	2.695014	1.371224	H	-2.6747	2.049407	2.942106
P	-0.07804	1.781776	0.215494	H	-1.3945	4.183721	2.929588
C	1.485704	1.925713	1.178846	H	-3.16564	3.816518	1.308222
H	2.307097	1.475528	0.625965	H	-0.51004	3.375188	-3.24082
H	1.375468	1.398724	2.127462	H	1.03123	4.776343	-1.92427
H	-2.58507	0.907811	1.590279	H	1.740049	2.605898	-2.97697
Pd	-0.66006	-0.59902	-0.25051	H	1.726081	2.970658	1.377744
C	0.847522	-1.18814	1.098198	C	-2.45014	-0.56432	-2.05213
C	2.229903	-0.99037	0.792355	H	-1.64617	-0.1906	-2.68102
C	0.402435	-1.32218	2.421459	C	-3.17453	0.275798	-1.26837
C	3.112726	-0.9052	1.904876	H	-2.87562	1.316035	-1.22156
C	1.291652	-1.21259	3.490219	H	-2.75463	-1.59252	-2.20737
H	-0.64784	-1.5237	2.611752	C	-4.42134	-0.0557	-0.561
C	2.650262	-1.00232	3.212818	C	-5.05823	0.901964	0.139434
H	4.163072	-0.70291	1.733537	H	-4.66622	1.90838	0.231948
H	0.937191	-1.3017	4.513133	H	-6.00252	0.698318	0.637282
H	3.362067	-0.91675	4.030648	C	-4.99926	-1.44154	-0.68526
C	-0.80043	-2.86228	-0.75202	H	-5.96714	-1.50755	-0.18155
H	-0.67751	-2.70061	-1.82264	H	-5.13767	-1.7182	-1.73712
C	0.379608	-2.94735	0.00055	H	-4.33155	-2.18821	-0.2526

III-3s Migratory insertion 3-selective σ complex

		Hartree
Zero-point correction	=	0.619235
Thermal correction to Energy	=	0.657299

Thermal correction to Enthalpy	=	0.658243
Thermal correction to Gibbs Free Energy	=	0.552092
E(RwB97XD)	=	-2049.64716894
		cm^{-1}
Lowest energy vibration	=	30.52
Second lowest energy vibration	=	32.71

0 1							
C	1.680809	2.665023	0.619651	H	-0.48704	0.103239	-2.34936
H	0.618596	2.412004	0.655738	C	-1.68326	-1.60946	-1.64854
H	2.158699	2.326015	1.540762	H	-2.4692	-1.14592	-2.25615
C	2.347944	2.09667	-0.64648	N	-2.55951	0.92717	-0.81487
C	1.512716	2.550078	-1.86371	S	-2.55822	2.504968	-0.49933
H	0.488489	2.167129	-1.83008	O	-2.12809	3.207434	-1.73176
H	1.970254	2.265347	-2.81547	O	-1.83489	2.876816	0.753366
C	3.777774	2.649449	-0.77956	C	-4.28227	2.997161	-0.24048
H	4.375347	2.460795	0.115679	H	-4.69405	2.457609	0.614904
H	4.306801	2.240401	-1.64574	H	-4.31149	4.07373	-0.05474
C	3.303433	-2.07875	0.603961	H	-4.84729	2.754973	-1.14297
H	3.248924	-2.57546	-0.37045	H	0.382496	-1.46461	-2.43873
C	3.647234	0.064332	1.871462	C	-1.62799	-3.09318	-1.99059
H	4.028469	1.088466	1.84872	C	-2.68283	-3.6899	-2.55837
H	2.644345	0.076993	2.302637	H	-3.58627	-3.13636	-2.80546
C	5.061195	-0.46793	-0.14665	H	-2.68056	-4.75194	-2.79403
H	5.378395	0.564691	-0.29807	C	-0.36197	-3.86366	-1.68367
H	5.132113	-0.99517	-1.10205	H	-0.5375	-4.94282	-1.71949
C	3.654992	-0.58	0.474595	H	0.426771	-3.63182	-2.40988
P	2.292502	0.192893	-0.61137	H	0.043622	-3.60851	-0.69872
C	2.79307	-0.29309	-2.31463	H	3.721228	3.736828	-0.9135
H	2.009906	-0.02844	-3.02528	H	1.446275	3.644627	-1.84671
H	2.943408	-1.37453	-2.35223	H	1.75439	3.759629	0.592584
H	2.350011	-2.23118	1.11532	H	4.296697	-0.51582	2.53826
Pd	0.188304	-0.55672	0.034573	H	5.779308	-0.93575	0.538242
C	-2.06768	-1.33464	-0.16434	H	4.085491	-2.57887	1.188277
C	-2.53488	-0.01332	0.190367	H	3.719034	0.207465	-2.60665
C	-2.13228	-2.37259	0.793562	O	0.241436	-0.36754	2.365684
C	-2.98238	0.175389	1.530079	C	-0.06358	0.838008	3.07305
C	-2.59605	-2.15617	2.084163	H	-0.80596	0.638971	3.855282
H	-1.83038	-3.37254	0.503891	H	0.841418	1.255607	3.534394
C	-3.01204	-0.8667	2.442868	H	-0.48639	1.542433	2.357582

H	-3.29684	1.161502	1.846742	C	0.541085	-1.46024	3.233784
H	-2.63026	-2.97162	2.800179	H	1.47208	-1.28048	3.787959
H	-3.36867	-0.67417	3.452157	H	-0.27814	-1.61874	3.944842
C	-0.35837	-0.87962	-1.89718	H	0.650419	-2.3527	2.616811

III-2s Migratory insertion 2-selective σ complex

		Hartree
Zero-point correction	=	0.619962
Thermal correction to Energy	=	0.657905
Thermal correction to Enthalpy	=	0.658849
Thermal correction to Gibbs Free Energy	=	0.552826
E(RwB97XD)	=	-2049.6522207
		cm^{-1}
Lowest energy vibration	=	15.78
Second lowest energy vibration	=	32.97

0 1							
C	-3.43144	-0.07112	1.933077	H	-0.34266	-0.86071	-2.4926
H	-2.73683	-0.91311	1.992322	C	1.091012	-2.24772	-1.57223
H	-3.0192	0.755531	2.514427	H	1.747502	-1.84724	-2.34748
C	-3.71241	0.308203	0.467898	N	2.704909	0.037619	-1.15883
C	-4.24429	-0.94663	-0.25652	S	3.495063	1.443116	-1.09943
H	-3.50106	-1.746	-0.2887	O	3.185652	2.175162	-2.34906
H	-4.56469	-0.73266	-1.27975	O	3.298371	2.201806	0.170425
C	-4.78833	1.406209	0.405465	C	5.262166	1.053938	-1.17628
H	-4.52091	2.284765	0.996648	H	5.537566	0.424665	-0.3275
H	-4.99548	1.724952	-0.62047	H	5.822411	1.991625	-1.14442
C	-0.11845	2.749193	-0.51204	H	5.459224	0.528323	-2.11275
H	-0.02678	2.529031	-1.5803	H	1.051683	-3.33528	-1.68416
C	-1.62466	2.859116	1.504271	C	-1.42997	-2.59997	-1.85722
H	-2.65189	2.965234	1.862827	C	-2.22972	-2.57031	-2.93892
H	-1.14644	2.057436	2.066637	H	-2.08802	-1.84605	-3.73662
C	-2.43946	3.639775	-0.74563	H	-3.03642	-3.28791	-3.0687
H	-3.49865	3.568962	-0.49384	C	-1.61128	-3.66411	-0.79784
H	-2.33649	3.568861	-1.83183	H	-1.7043	-3.21927	0.19933
C	-1.57115	2.598667	-0.01104	H	-0.74728	-4.3378	-0.76268
P	-2.08151	0.798533	-0.38936	H	-2.50017	-4.27084	-0.993
C	-2.52355	0.878126	-2.16981	H	0.205006	3.786413	-0.36058

H	-2.70301	-0.12655	-2.54795	H	-2.09753	4.639842	-0.45105
H	-1.69091	1.316829	-2.72452	H	-1.09852	3.794884	1.728533
H	0.582849	2.103952	0.018269	H	-5.72226	1.004791	0.818539
Pd	-0.27187	-0.6241	0.098475	H	-4.37099	-0.37726	2.409389
C	1.695197	-1.9112	-0.20192	H	-3.41903	1.483956	-2.32614
C	2.599055	-0.79091	-0.06826	H	-5.1172	-1.32174	0.291352
C	1.527451	-2.8144	0.873106	O	0.271569	0.142114	2.321194
C	3.301475	-0.68475	1.164713	C	1.189625	1.166077	2.718995
C	2.217289	-2.66825	2.070898	H	0.671686	1.952839	3.283832
H	0.861393	-3.66041	0.730153	H	1.981945	0.741684	3.345702
C	3.112364	-1.59757	2.193548	H	1.641959	1.583446	1.820054
H	3.969114	0.15186	1.32429	C	-0.24292	-0.57864	3.43968
H	2.074062	-3.37308	2.884064	H	0.576147	-0.96962	4.055939
H	3.669504	-1.46495	3.118361	H	-0.8851	0.062804	4.058604
C	-0.30727	-1.62882	-1.71876	H	-0.82616	-1.41678	3.057419

IV-2s Migratory insertion 2 selectivity π complex

		Hartree
Zero-point correction	=	0.619504
Thermal correction to Energy	=	0.657658
Thermal correction to Enthalpy	=	0.658602
Thermal correction to Gibbs Free Energy	=	0.550421
E(RwB97XD)	=	-2049.6693905
		cm^{-1}
Lowest energy vibration	=	12.25
Second lowest energy vibration	=	29.00

0 1							
C	4.014331	0.106878	1.756803	H	-1.12495	-1.00114	-0.91864
H	3.704175	-0.9122	1.512836	C	-1.55459	-1.3012	1.19792
H	4.577923	0.503118	0.910586	H	-1.35154	-0.29093	1.550251
C	2.798222	0.973775	2.131552	N	-2.81601	0.694697	-0.21579
C	2.038846	0.256347	3.270325	S	-3.35289	2.066278	-0.84556
H	1.589349	-0.68431	2.930571	O	-2.15971	2.913549	-1.11508
H	1.251043	0.879812	3.701881	O	-4.30619	1.924615	-1.98398
C	3.269824	2.350678	2.627367	C	-4.27607	2.909923	0.465818
H	3.911069	2.850701	1.897199	H	-5.10648	2.276627	0.785956
H	2.433586	3.014231	2.867314	H	-4.65731	3.854455	0.070656

C	1.217435	2.048178	-1.88857	H	-3.5996	3.093863	1.302891
H	0.156732	2.183401	-1.65115	H	-1.27817	-1.99805	1.992733
C	3.598908	2.090415	-1.06017	C	-0.10449	-2.81099	-0.33742
H	4.282653	2.429819	-0.2769	C	0.582225	-2.87048	-1.55121
H	3.813071	1.03989	-1.26733	H	0.269451	-2.26891	-2.40263
C	1.931538	3.786824	-0.24379	H	1.253527	-3.69853	-1.76195
H	2.492746	4.047862	0.655601	C	0.047325	-3.89283	0.702059
H	0.878329	4.027913	-0.07767	H	-0.90932	-4.41334	0.828031
C	2.128087	2.318633	-0.66865	H	0.330241	-3.48634	1.676612
P	1.591199	1.06082	0.662036	H	0.798902	-4.62494	0.396233
C	0.10333	1.871953	1.380118	H	1.473259	2.755873	-2.68768
H	-0.3422	1.230607	2.139926	H	2.288965	4.43396	-1.05476
H	-0.63528	2.043733	0.593245	H	3.823887	2.668591	-1.96508
H	1.353226	1.035281	-2.28115	H	3.857052	2.217525	3.54489
Pd	1.325835	-1.07742	-0.39209	H	4.693888	0.052019	2.616323
C	-3.03625	-1.40569	0.886217	H	0.370274	2.822654	1.848533
C	-3.65715	-0.34314	0.154609	H	2.745861	0.016713	4.073651
C	-3.78296	-2.50983	1.296333	O	3.450491	-1.33555	-1.3227
C	-5.04404	-0.46651	-0.11526	C	3.471266	-1.23746	-2.75478
C	-5.15287	-2.61083	1.025363	H	4.508002	-1.20122	-3.11203
H	-3.2819	-3.30094	1.851336	H	2.957133	-2.09228	-3.21147
C	-5.77029	-1.5775	0.317439	H	2.961406	-0.31446	-3.03008
H	-5.54041	0.303742	-0.69465	C	4.140666	-2.50986	-0.87271
H	-5.71995	-3.47503	1.359665	H	3.692579	-3.41423	-1.30312
H	-6.83252	-1.6345	0.088619	H	5.198447	-2.45466	-1.15899
C	-0.75022	-1.55447	-0.05843	H	4.059574	-2.54867	0.213418

cis-I cis chelate complex

		Hartree
Zero-point correction	=	0.535462
Thermal correction to Energy	=	0.568681
Thermal correction to Enthalpy	=	0.569625
Thermal correction to Gibbs Free Energy	=	0.473569
E(RwB97XD)	=	-1894.58249182
		cm^{-1}
Lowest energy vibration	=	25.00
Second lowest energy vibration	=	39.79

0 1							
C	3.315276	1.321496	-1.24287	H	-3.4222	-3.52548	0.343958
H	2.375851	1.710857	-1.63775	H	-0.92896	-3.80348	-3.16229
H	3.572725	1.872833	-0.33719	H	-2.6991	-4.77145	-1.68611
C	3.232229	-0.19863	-1.02244	N	-2.51734	-1.23961	1.226692
C	2.825596	-0.8379	-2.36746	S	-3.07239	0.220843	0.979546
H	1.855383	-0.46594	-2.71027	O	-3.14633	0.986374	2.244024
H	2.790939	-1.92964	-2.32672	O	-2.34944	0.951154	-0.16582
C	4.614112	-0.73385	-0.60544	C	-4.75261	0.11402	0.324605
H	5.000608	-0.22643	0.281417	H	-4.7295	-0.50066	-0.57794
H	4.610365	-1.81147	-0.41941	H	-5.10977	1.119565	0.092466
C	1.110898	-0.37955	2.902884	H	-5.38218	-0.35105	1.085793
H	0.615022	-1.34851	2.786332	H	1.384067	-0.26885	3.959418
C	3.006581	1.124581	2.177089	H	3.693736	-1.00953	3.596444
H	4.013096	1.165231	1.75276	H	3.087047	1.379296	3.240638
H	2.404777	1.89709	1.695505	H	5.32015	-0.54612	-1.42378
C	3.399648	-1.31717	2.585531	H	4.095405	1.525823	-1.98627
H	4.308762	-1.38518	1.986253	H	2.829271	-2.8863	0.409872
H	2.961168	-2.31503	2.665418	H	3.569538	-0.56005	-3.12354
C	2.392127	-0.28008	2.044954	C	0.298855	2.972646	-0.12948
P	1.867501	-0.63759	0.239246	H	1.38025	3.00987	-0.23905
C	1.835351	-2.47887	0.21674	C	-0.20697	2.632049	1.081804
H	1.474916	-2.84412	-0.7422	H	0.449599	2.479938	1.930197
H	1.145125	-2.82431	0.989441	H	-1.27321	2.601206	1.275344
H	0.383423	0.396826	2.658297	C	-0.48021	3.39203	-1.31679
Pd	-0.21887	0.364029	-0.14538	C	-1.66334	4.013408	-1.19808
C	-0.97551	-1.41427	-0.72351	H	-2.0965	4.240531	-0.22871
C	-2.03825	-1.9087	0.074736	H	-2.22796	4.32134	-2.07438
C	-0.61271	-2.0859	-1.89263	C	0.149733	3.108067	-2.65957
C	-2.62657	-3.13642	-0.28678	H	0.261449	2.027459	-2.81456
C	-1.23308	-3.29053	-2.25352	H	1.154928	3.543072	-2.72289
H	0.179319	-1.69654	-2.52214	H	-0.45184	3.51089	-3.47917
C	-2.22357	-3.82804	-1.43007				

cis-II cis open OMe_2 complex

		Hartree
Zero-point correction	=	0.617331
Thermal correction to Energy	=	0.656339
Thermal correction to Enthalpy	=	0.657283
Thermal correction to Gibbs Free Energy	=	0.547503

E(RwB97XD)	=	-2049.62989931
		cm^{-1}
Lowest energy vibration	=	9.15
Second lowest energy vibration	=	28.65

0 1							
C	-2.01009	2.209457	-1.75977	H	-0.13007	-3.27356	-1.48999
H	-1.09708	1.718295	-2.10558	C	1.343461	-1.7728	-1.71085
H	-2.86232	1.57341	-2.00877	H	0.942213	-1.40002	-2.64756
C	-1.9152	2.52758	-0.25725	N	2.052747	1.117566	-0.34815
C	-0.60758	3.31226	-0.01157	S	3.364651	1.888089	-0.86426
H	0.282604	2.706163	-0.21057	O	3.04594	2.44749	-2.20064
H	-0.5476	3.701679	1.00891	O	3.964979	2.846716	0.109855
C	-3.10791	3.39727	0.17651	C	4.632987	0.620478	-1.13524
H	-4.0693	2.944108	-0.07559	H	4.810237	0.078962	-0.2034
H	-3.09385	3.614774	1.248622	H	5.552485	1.114891	-1.45769
C	-3.1626	-1.32552	1.6073	H	4.283369	-0.06628	-1.90858
H	-2.57241	-1.2909	2.528914	H	2.287164	-1.34852	-1.38889
C	-4.28444	-0.02422	-0.2355	C	1.367131	-3.57731	0.053222
H	-4.66622	0.947142	-0.56035	C	0.643184	-4.54711	0.640327
H	-3.69427	-0.44676	-1.04899	H	-0.36533	-4.78274	0.308071
C	-4.29956	0.837212	2.130504	H	1.038473	-5.13046	1.467261
H	-4.53511	1.862049	1.840448	C	2.771914	-3.24286	0.483291
H	-3.79978	0.858444	3.102688	H	2.855801	-2.19149	0.771709
C	-3.47244	0.088277	1.066933	H	3.476007	-3.40895	-0.34161
P	-1.7819	0.903387	0.729065	H	3.08119	-3.85855	1.331693
C	-1.25034	1.462344	2.398839	H	-4.10638	-1.83527	1.836379
H	-0.21407	1.7962	2.363244	H	-5.25004	0.305703	2.263715
H	-1.31638	0.629316	3.100014	H	-5.14862	-0.67742	-0.06407
H	-2.61575	-1.93125	0.879109	H	-2.11598	3.147696	-2.31783
Pd	-0.38568	-0.65424	-0.3816	H	-3.04718	4.356253	-0.35291
C	0.900518	-0.38426	1.113846	H	-0.59019	4.173058	-0.69146
C	2.020367	0.458732	0.874456	H	-1.889	2.279129	2.743722
C	0.754535	-1.10781	2.291277	O	-1.79127	-1.24962	-2.20562
C	2.986793	0.519151	1.914899	C	-2.54703	-2.46607	-2.20284
C	1.734892	-1.04377	3.294102	H	-3.53623	-2.30312	-2.6491
H	-0.12171	-1.73377	2.437199	H	-2.02667	-3.24976	-2.76862
C	2.843729	-0.22235	3.090851	H	-2.66473	-2.78253	-1.16644
H	3.843607	1.17339	1.797798	C	-1.55809	-0.7766	-3.5367
H	1.621908	-1.61815	4.209588	H	-1.06575	-1.54892	-4.14192

H	3.611636	-0.14529	3.857604	H	-2.50613	-0.49712	-4.01359
C	0.77942	-2.84242	-1.07938	H	-0.91172	0.09751	-3.47257

cis-TS1-3s

		Hartree
Zero-point correction	=	0.616019
Thermal correction to Energy	=	0.654160
Thermal correction to Enthalpy	=	0.655104
Thermal correction to Gibbs Free Energy	=	0.548157
E(RwB97XD)	=	-2049.59753248
		cm^{-1}
Lowest energy vibration	=	-401.10
Second lowest energy vibration	=	21.27

0 1							
C	3.73704	-1.15359	1.216251	H	-0.68266	-2.09573	-2.42387
H	3.027873	-1.94766	0.967168	C	-2.06095	-1.89205	-0.79077
H	4.281384	-0.88657	0.30818	H	-2.48988	-1.03531	-1.30544
C	3.010544	0.043665	1.858212	N	-1.8371	1.182736	-0.6113
C	2.202833	-0.48687	3.063962	S	-2.58014	2.594856	-0.895
H	1.397008	-1.15945	2.753531	O	-2.13296	3.051076	-2.23041
H	1.763886	0.320506	3.656733	O	-2.44978	3.582117	0.21269
C	4.037056	1.070254	2.363853	C	-4.34486	2.21827	-1.04351
H	4.720572	1.393192	1.57474	H	-4.706	1.786077	-0.10807
H	3.558054	1.956291	2.791965	H	-4.87788	3.148411	-1.25533
C	1.462514	2.203602	-1.70544	H	-4.4783	1.510509	-1.864
H	0.5536	2.64423	-1.28766	H	-0.57236	-3.4368	-1.17198
C	3.758075	1.266904	-1.30047	C	-3.0644	-2.7293	-0.08549
H	4.605814	1.186714	-0.6144	C	-4.3007	-2.23773	0.098178
H	3.522432	0.266188	-1.67119	H	-4.56923	-1.23817	-0.2351
C	2.959161	3.288523	-0.02695	H	-5.07716	-2.81887	0.58851
H	3.70836	3.188611	0.76024	C	-2.67513	-4.11624	0.356164
H	2.103729	3.835625	0.37956	H	-3.47654	-4.58583	0.932692
C	2.541639	1.935077	-0.63341	H	-2.45765	-4.75332	-0.51003
P	1.739135	0.742799	0.620663	H	-1.77022	-4.10431	0.972809
C	0.723401	1.873044	1.664497	H	1.348119	2.539789	2.26444
H	0.093341	1.279755	2.327588	H	2.875533	-1.05101	3.721835
H	0.067039	2.464926	1.023317	H	4.643505	0.610803	3.155257

H	1.166787	1.287806	-2.22404	H	4.460667	-1.56423	1.931783
Pd	0.455896	-1.03077	-0.47688	H	1.862907	2.901959	-2.45147
C	-1.24718	-0.73556	0.670868	H	3.395609	3.910248	-0.81929
C	-1.94613	0.52243	0.578041	H	4.082751	1.87684	-2.15301
C	-1.24257	-1.46477	1.883737	O	2.158936	-1.76576	-1.86587
C	-2.67935	0.930226	1.731367	C	2.509677	-3.14533	-1.69148
C	-1.93454	-1.02022	2.997985	H	1.749518	-3.8007	-2.1349
H	-0.70373	-2.40632	1.924459	H	3.480724	-3.34559	-2.16141
C	-2.66887	0.177322	2.895947	H	2.575711	-3.33864	-0.62124
H	-3.21024	1.87418	1.718133	C	2.032736	-1.43432	-3.2558
H	-1.92372	-1.58763	3.923808	H	2.985549	-1.61313	-3.76943
H	-3.23603	0.532009	3.753652	H	1.246843	-2.03334	-3.73278
C	-0.86802	-2.4129	-1.39571	H	1.77647	-0.37749	-3.32169

cis-TS1-2s

		Hartree
Zero-point correction	=	0.616896
Thermal correction to Energy	=	0.654956
Thermal correction to Enthalpy	=	0.655900
Thermal correction to Gibbs Free Energy	=	0.548833
E(RwB97XD)	=	-2049.60024813
		cm^{-1}
Lowest energy vibration	=	-365.79
Second lowest energy vibration	=	13.51

0 1							
C	-1.41436	2.271863	-2.37049	H	-0.24249	-2.80811	-1.61605
H	-1.09607	1.267349	-2.65347	C	0.780573	-2.80386	0.255325
H	-2.48676	2.246176	-2.16903	H	1.720365	-2.65408	-0.2659
C	-0.58723	2.794914	-1.18191	N	2.461984	-0.3302	-0.53759
C	0.9055	2.705649	-1.56826	S	3.879314	0.183455	-1.1253
H	1.246725	1.672765	-1.68223	O	3.732141	0.252609	-2.59727
H	1.552943	3.195058	-0.83537	O	4.41845	1.404697	-0.46285
C	-0.94139	4.267081	-0.90805	C	5.073118	-1.13693	-0.79116
H	-2.0107	4.413226	-0.73849	H	5.124716	-1.31609	0.285003
H	-0.39522	4.673637	-0.05161	H	6.050284	-0.82156	-1.16483
C	-2.66945	0.863405	2.235823	H	4.74355	-2.0404	-1.3079
H	-1.82431	0.736079	2.920204	H	0.837984	-3.35061	1.188809

C	-3.63828	2.122202	0.286565	C	-1.57605	-3.67696	-0.12144
H	-3.62277	3.044731	-0.29937	C	-2.29793	-4.34734	-1.03931
H	-3.65541	1.281282	-0.40763	H	-2.02672	-4.3427	-2.09168
C	-2.38799	3.340885	2.09363	H	-3.17229	-4.92873	-0.75929
H	-2.17615	4.222898	1.486722	C	-1.96291	-3.68022	1.338431
H	-1.63998	3.283014	2.889144	H	-2.23144	-2.66845	1.665756
C	-2.45163	2.0439	1.263151	H	-1.13822	-4.01116	1.979768
P	-0.82989	1.655912	0.329042	H	-2.81718	-4.33816	1.519611
C	0.452587	2.254118	1.50611	H	-3.56408	1.054679	2.841638
H	1.440627	1.998954	1.128174	H	-3.36247	3.497167	2.573635
H	0.318497	1.753386	2.466884	H	-4.57648	2.109574	0.855226
H	-2.81569	-0.08206	1.704893	H	-1.25232	2.930006	-3.23339
Pd	-0.65974	-0.73125	-0.1527	H	-0.66435	4.865373	-1.78553
C	0.934086	-0.95655	1.180657	H	1.050874	3.218879	-2.52701
C	2.217347	-0.43164	0.810056	H	0.3911	3.33512	1.649037
C	0.581676	-1.11341	2.533316	O	-2.49296	-0.76061	-1.58236
C	3.093313	-0.08456	1.878514	C	-3.78186	-1.19726	-1.12958
C	1.454461	-0.74791	3.554433	H	-4.56803	-0.69435	-1.70709
H	-0.3833	-1.54783	2.778172	H	-3.88582	-2.28168	-1.23435
C	2.713045	-0.23245	3.207684	H	-3.87182	-0.92669	-0.07821
H	4.058077	0.352003	1.649254	C	-2.25459	-1.14267	-2.94267
H	1.169172	-0.86953	4.59546	H	-2.34768	-2.22769	-3.06497
H	3.411069	0.058431	3.989477	H	-2.97114	-0.63969	-3.60435
C	-0.38267	-2.91613	-0.53926	H	-1.24102	-0.83582	-3.20236

TS1-3s-L13

		Hartree
Zero-point correction	=	0.498221
Thermal correction to Energy	=	0.532391
Thermal correction to Enthalpy	=	0.533335
Thermal correction to Gibbs Free Energy	=	0.429829
E(RwB97XD)	=	-2005.44323359
		cm^{-1}
Lowest energy vibration	=	-178.17
Second lowest energy vibration	=	13.23

0 1							
C	-2.15556	-2.99066	-0.94248	H	-1.22172	2.957935	-0.62399
P	-2.2293	-1.17694	-1.19759	H	-3.59614	-1.58407	-3.18924
C	-2.70158	-0.99589	-2.9636	O	-0.96257	-1.01262	1.679152
H	-1.87138	-1.33918	-3.58811	C	0.069944	-1.79539	2.293495

H	-2.89586	0.056769	-3.18498	H	0.850765	-1.15306	2.716388
Pd	-0.34278	-0.01469	-0.3626	H	-0.36039	-2.42364	3.083626
C	1.269723	0.937987	0.606073	H	0.502901	-2.42654	1.515781
C	2.46688	0.17087	0.713989	C	-1.63097	-0.15864	2.615467
C	0.938127	1.932828	1.524029	H	-2.11262	-0.76365	3.393968
C	3.26275	0.425208	1.86763	H	-0.92513	0.541325	3.077869
C	1.742061	2.164021	2.645044	H	-2.39097	0.395103	2.06288
H	0.043208	2.530647	1.369313	H	-3.09579	-3.47829	-1.21687
C	2.902024	1.396071	2.800664	H	-1.3488	-3.38268	-1.56873
H	4.1574	-0.16435	2.032244	H	-1.91695	-3.20075	0.102906
H	1.470313	2.922886	3.37338	C	-3.69437	-0.58837	-0.26747
H	3.544122	1.556887	3.663961	C	-4.0353	0.772379	-0.37295
C	0.249843	0.789744	-2.21445	C	-4.41233	-1.40475	0.617792
H	0.710886	-0.05735	-2.72036	C	-5.08142	1.300608	0.382642
C	1.123971	1.700095	-1.56028	H	-3.47377	1.422142	-1.04
H	2.176987	1.438981	-1.54631	C	-5.45551	-0.87018	1.379989
N	2.717062	-0.73265	-0.30295	H	-4.16202	-2.45488	0.726787
S	4.118217	-1.52757	-0.40208	C	-5.79209	0.479775	1.26524
O	4.040386	-2.4011	-1.59661	H	-5.33693	2.352495	0.289656
O	4.551007	-2.19223	0.860726	H	-6.00258	-1.51196	2.065012
C	5.38585	-0.28484	-0.76428	H	-6.60109	0.892754	1.861088
H	5.402626	0.461887	0.03254	H	1.607771	5.068929	-1.12281
H	6.354275	-0.78717	-0.82443	C	-0.65661	3.537187	-1.36426
H	5.146113	0.189219	-1.71814	H	-0.76129	4.599506	-1.12972
H	-0.64612	1.178384	-2.69236	H	-1.12957	3.355937	-2.33677
C	0.792692	3.122868	-1.36863	H	2.835759	3.683039	-1.21565
C	1.797178	4.004059	-1.22664				

TS1-2s-L13

		Hartree
Zero-point correction	=	0.499225
Thermal correction to Energy	=	0.533034
Thermal correction to Enthalpy	=	0.533978
Thermal correction to Gibbs Free Energy	=	0.433132
E(RwB97XD)	=	-2005.44611169
		cm^{-1}
Lowest energy vibration	=	-108.79
Second lowest energy vibration	=	14.83

0 1							
C	3.032251	0.375919	-2.26349	H	2.406477	3.617833	-2.73977

P	2.526783	0.247153	-0.50304	H	3.037912	4.781594	-1.44479
C	3.794181	1.250685	0.374626	C	1.241467	4.158346	0.510889
H	3.617479	1.214092	1.45213	H	1.388917	3.361656	1.250608
H	3.698088	2.286457	0.038502	H	0.248881	4.58428	0.695869
Pd	0.255183	0.870732	0.054285	H	1.984297	4.94012	0.689709
C	-1.66688	1.174667	0.79033	H	4.802036	0.886282	0.154542
C	-2.60842	0.215714	0.329495	O	0.272501	-0.94804	1.437854
C	-1.8525	1.919742	1.94749	C	-0.23502	-2.18054	0.902125
C	-3.71019	-0.02563	1.193455	H	0.184664	-3.02089	1.468515
C	-2.96862	1.682899	2.764377	H	-1.32796	-2.20478	0.936653
H	-1.12187	2.675066	2.228401	H	0.093634	-2.24087	-0.13411
C	-3.87918	0.696991	2.377712	C	-0.04772	-0.78656	2.8228
H	-4.42132	-0.80451	0.943415	H	-1.13163	-0.74126	2.97527
H	-3.11236	2.250759	3.679423	H	0.372181	-1.62448	3.393517
H	-4.74376	0.483424	3.002402	H	0.407387	0.146763	3.157091
C	0.389459	2.581665	-1.34051	H	4.033224	-0.03862	-2.41387
H	0.624953	2.101854	-2.28904	H	2.315847	-0.14714	-2.90149
C	-0.95563	2.560037	-0.93041	H	3.036284	1.432515	-2.53875
H	-1.68791	2.071762	-1.56437	C	2.913448	-1.48217	-0.03419
N	-2.33072	-0.37439	-0.89272	C	3.16783	-1.80144	1.310473
S	-3.17862	-1.62151	-1.44991	C	2.867831	-2.51682	-0.98144
O	-2.47972	-2.12415	-2.65787	C	3.383855	-3.12449	1.69464
O	-3.52162	-2.64421	-0.41871	H	3.187305	-1.02144	2.065019
C	-4.76362	-0.96349	-2.03368	C	3.080239	-3.8422	-0.59268
H	-5.2827	-0.47478	-1.20632	C	3.33821	-4.14976	0.744453
H	-5.36576	-1.79212	-2.41462	H	3.582908	-3.3557	2.737411
H	-4.56667	-0.24276	-2.82994	H	3.042514	-4.6322	-1.33754
H	-1.3275	3.332937	-0.27027	H	3.501588	-5.18055	1.045943
C	1.361011	3.605032	-0.88727	H	2.661791	-2.30018	-2.02489
C	2.322084	4.016383	-1.73232				

TS1-3s-L14

		Hartree
Zero-point correction	=	0.523598
Thermal correction to Energy	=	0.573068
Thermal correction to Enthalpy	=	0.574012
Thermal correction to Gibbs Free Energy	=	0.436865
E(RwB97XD)	=	-3381.23441697
		cm^{-1}
Lowest energy vibration	=	-179.74

Second lowest energy vibration = 8.89

0 1							
P	-1.15679	-0.28322	0.584297	H	-0.33158	-0.0673	-4.12777
Pd	0.93748	0.2566	-0.40734	H	0.123694	1.329218	-3.16016
C	2.814115	0.897568	-1.07687	O	0.942741	2.038089	1.162372
C	3.868178	0.325147	-0.30374	C	2.016598	2.001685	2.113683
C	2.933142	2.12683	-1.71934	H	2.967995	2.262846	1.63673
C	5.037602	1.126514	-0.16951	H	1.808624	2.700482	2.933579
C	4.095406	2.889211	-1.56933	H	2.073434	0.986084	2.504558
H	2.117206	2.494548	-2.33602	C	0.759882	3.35097	0.616119
C	5.136915	2.371101	-0.78889	H	0.472104	4.048054	1.412843
H	5.854436	0.771953	0.448423	H	1.677455	3.703416	0.132107
H	4.185315	3.858998	-2.05013	H	-0.03926	3.290096	-0.12308
H	6.050546	2.947272	-0.66048	C	-2.07082	1.309281	0.488556
C	1.007948	-1.28432	-1.83911	C	-2.38713	1.790138	-0.78746
H	1.283521	-2.15567	-1.2531	C	-2.2389	2.200992	1.549485
C	2.046707	-0.64889	-2.56862	C	-2.87909	3.066467	-1.01142
H	3.049048	-1.04485	-2.44172	C	-2.72397	3.492777	1.35181
N	3.622253	-0.92226	0.231021	C	-3.04931	3.925818	0.072465
S	4.727106	-1.71978	1.096213	C	-2.25806	-1.55293	-0.17904
O	4.082946	-2.96551	1.574142	C	-1.69395	-2.82164	-0.35899
O	5.41147	-0.89675	2.133199	C	-3.58227	-1.40025	-0.59662
C	6.017165	-2.22829	-0.06855	C	-2.37364	-3.87095	-0.96282
H	6.450127	-1.34378	-0.54102	C	-4.2872	-2.43415	-1.20799
H	6.788201	-2.77078	0.483827	C	-3.68155	-3.67159	-1.39518
H	5.56695	-2.87532	-0.82398	C	-1.11289	-0.83872	2.321227
H	0.008347	-1.30605	-2.2686	C	0.135349	-1.16959	2.865582
C	1.798084	0.129437	-3.79325	C	-2.28263	-1.00915	3.078456
C	2.799904	0.271913	-4.67767	C	0.214929	-1.64779	4.176189
H	3.786128	-0.145	-4.48951	H	1.036155	-1.07141	2.266322
H	2.658296	0.802881	-5.61499	C	-2.19486	-1.48311	4.385061
C	0.42743	0.715968	-4.01644	C	-0.9464	-1.79906	4.935311
H	0.408592	1.336344	-4.91597	H	1.183154	-1.90367	4.596199
F	-1.89825	1.873773	2.80381	H	-3.09796	-1.60877	4.975152
F	-2.86052	4.324059	2.392566	H	-0.88363	-2.16973	5.954454
F	-3.51439	5.162166	-0.121	F	-4.24661	-0.24797	-0.41317
F	-3.17675	3.478352	-2.24963	F	-5.55317	-2.24541	-1.60063
F	-2.19404	0.992714	-1.85682	F	-4.355	-4.66719	-1.97532
H	-3.25051	-0.7649	2.649483	F	-1.78781	-5.06349	-1.11895
F	-0.44518	-3.06067	0.082839				

TS1-2s-L14

		Hartree
Zero-point correction	=	0.523901
Thermal correction to Energy	=	0.573295
Thermal correction to Enthalpy	=	0.574239
Thermal correction to Gibbs Free Energy	=	0.439414
E(RwB97XD)	=	-3381.23695129
		cm^{-1}
Lowest energy vibration	=	-136.47
Second lowest energy vibration	=	19.07

0 1							
P	0.994013	0.301861	0.600784	C	-0.74213	1.099574	-2.88147
Pd	-0.8219	-0.94741	-0.43398	H	-1.26757	0.476419	-3.61531
C	-2.5283	-1.68825	-1.33994	H	-0.75581	2.145549	-3.21174
C	-3.7227	-1.27854	-0.69109	H	0.289491	0.764051	-2.78468
C	-2.47839	-2.09225	-2.66712	C	1.092445	2.013848	-0.08455
C	-4.87833	-1.23221	-1.5139	C	2.121559	2.581521	-0.83929
C	-3.6416	-2.04565	-3.45053	C	-0.05875	2.794916	0.079605
H	-1.53962	-2.43251	-3.09757	C	2.003133	3.843848	-1.41788
C	-4.82932	-1.60769	-2.8583	C	-0.20647	4.048764	-0.49366
H	-5.80807	-0.85423	-1.10474	C	0.834892	4.577655	-1.25156
H	-3.61318	-2.34504	-4.49438	C	0.798241	0.631509	2.389336
H	-5.74027	-1.55565	-3.45024	C	1.59565	1.571732	3.062218
C	-0.16342	-2.85695	0.569311	C	-0.25713	0.001167	3.062227
H	-0.11015	-2.55176	1.611922	C	1.353109	1.852783	4.404295
C	-1.43396	-3.14728	0.050933	C	-0.49662	0.289299	4.40834
H	-2.29313	-3.06165	0.707153	C	0.309101	1.209425	5.079727
N	-3.60426	-0.95355	0.650825	C	2.650066	-0.43983	0.301062
S	-4.79844	-0.24721	1.466268	C	2.92949	-0.82813	-1.01402
O	-4.26542	0.116295	2.801171	C	3.581939	-0.82551	1.266469
O	-5.48456	0.840383	0.712058	C	4.060088	-1.5434	-1.37153
C	-6.05719	-1.51527	1.772095	C	4.733544	-1.53869	0.934937
H	-6.41937	-1.91347	0.822175	C	4.975495	-1.89839	-0.38436
H	-6.88214	-1.05754	2.323597	F	6.06862	-2.59476	-0.7022
H	-5.60478	-2.31341	2.364189	F	4.264482	-1.90763	-2.64303
H	-1.52543	-3.82044	-0.79175	F	2.061951	-0.51149	-1.99674
C	1.084303	-3.41878	0.012666	F	5.59931	-1.89448	1.891992
C	2.104383	-3.66925	0.851192	F	3.403994	-0.56253	2.568313
H	2.044254	-3.42978	1.909688	H	2.402527	2.077144	2.540294
H	3.024807	-4.12775	0.502558	H	1.973836	2.577253	4.923369
C	1.161246	-3.72847	-1.46167	H	0.119808	1.436457	6.124998

H	0.986392	-2.82389	-2.05466	H	-1.3218	-0.19586	4.920952
H	0.403713	-4.46136	-1.7627	H	-0.90997	-0.68593	2.535034
H	2.143265	-4.12829	-1.72724	F	3.282959	1.939824	-1.04782
O	-1.35455	0.988782	-1.5923	F	3.015357	4.352886	-2.13265
C	-2.67293	1.560066	-1.56899	F	0.713468	5.783774	-1.81082
H	-2.59575	2.642534	-1.72894	F	-1.34474	4.737446	-0.33912
H	-3.3022	1.114605	-2.34627	F	-1.08575	2.318821	0.802828
H	-3.10623	1.357437	-0.59216				

V P^{t}Bu$_3$ trans complex

		Hartree
Zero-point correction	=	0.620319
Thermal correction to Energy	=	0.657817
Thermal correction to Enthalpy	=	0.658761
Thermal correction to Gibbs Free Energy	=	0.553449
E(RwB97XD)	=	-2012.50477785
		cm^{-1}
Lowest energy vibration	=	20.99
Second lowest energy vibration	=	26.86

0 1							
C	1.547525	-0.45626	-2.75813	H	-3.70254	0.342627	3.977936
H	0.613432	0.114022	-2.67844	C	0.21568	3.331697	-0.01145
H	2.386263	0.217173	-2.58714	H	-0.01145	3.439996	1.04719
C	1.534776	-1.69642	-1.83195	C	-0.80221	2.968733	-0.86126
C	0.246415	-2.46887	-2.18764	H	-1.81159	2.840242	-0.48561
H	-0.65697	-1.89561	-1.95753	H	-0.68317	2.984958	-1.94166
H	0.185117	-3.44209	-1.69907	N	-2.41843	-0.02359	-0.49357
C	2.758717	-2.57504	-2.15164	S	-3.77912	-0.61691	-1.11386
H	3.699574	-2.03038	-2.05298	O	-4.40263	-1.71	-0.31471
H	2.811634	-3.46853	-1.52821	O	-3.50976	-0.92361	-2.53927
C	3.016538	0.425572	1.822634	C	-4.97758	0.742102	-1.1109
H	2.560774	-0.07943	2.6726	H	-4.5831	1.553718	-1.72569
C	3.844143	0.405399	-0.50456	H	-5.92028	0.376821	-1.52563
H	4.124398	-0.1502	-1.40014	H	-5.13048	1.08812	-0.08622
H	3.204579	1.244702	-0.7914	C	1.573932	3.733828	-0.41759
C	4.17707	-1.62222	0.920878	C	2.450386	4.110302	0.531559
H	4.337447	-2.30101	0.081328	H	2.175829	4.123875	1.583296

H	3.855069	-2.20833	1.782874	H	3.461642	4.417759	0.280852
C	3.197849	-0.48167	0.582342	C	1.930978	3.710238	-1.88183
P	1.446833	-1.07881	0.001235	H	1.784781	2.712084	-2.31205
C	0.853525	-2.52579	1.136791	H	1.29236	4.395707	-2.45185
H	2.41574	1.307317	1.58254	H	2.973368	3.998556	-2.03796
C	1.562579	-3.86015	0.822468	H	4.764064	0.832458	-0.08733
H	1.288618	-4.25473	-0.15733	H	4.007321	0.773484	2.140205
H	2.649601	-3.79357	0.876851	H	5.147599	-1.17871	1.176246
H	1.241072	-4.59794	1.567927	H	0.25302	-2.65169	-3.26924
Pd	0.232984	0.963121	-0.26957	H	1.614487	-0.80112	-3.79746
C	-1.00519	0.720896	1.264374	H	2.683022	-2.90556	-3.19525
C	-0.69173	1.062539	2.569126	C	1.097189	-2.17329	2.61999
C	-2.28296	0.253933	0.856824	H	0.627579	-2.95424	3.230105
C	-1.67311	0.935936	3.569704	H	2.154396	-2.1502	2.886977
H	0.303988	1.413672	2.82286	H	0.636352	-1.22228	2.896165
C	-3.24307	0.122905	1.894086	C	-0.67039	-2.72944	0.988145
C	-2.93592	0.46089	3.21551	H	-1.22486	-1.87129	1.363006
H	-1.43914	1.195905	4.59818	H	-0.99485	-2.92859	-0.03023
H	-4.22152	-0.28112	1.659909	H	-0.94986	-3.59857	1.596512

TS2-3s P^{t}Bu$_3$ trans 3-selective TS

		Hartree
Zero-point correction	=	0.619459
Thermal correction to Energy	=	0.656127
Thermal correction to Enthalpy	=	0.657071
Thermal correction to Gibbs Free Energy	=	0.553703
E(RwB97XD)	=	-2012.46727423
		cm^{-1}
Lowest energy vibration	=	-398.21
Second lowest energy vibration	=	20.99

0 1							
Pd	0.171262	0.793178	-0.95731	C	2.358405	-2.30249	-1.94395
C	-1.33955	0.897633	0.486253	H	1.275793	-2.16852	-2.03622
C	-2.08366	-0.32649	0.636308	H	2.553009	-3.13844	-1.27089
C	-1.09603	1.745611	1.584957	C	4.566045	-1.30352	-1.31045
C	-2.51161	-0.63785	1.963292	H	5.152673	-0.40093	-1.12984
C	-1.54238	1.415265	2.85482	H	4.75003	-1.99974	-0.49015

H	-0.56774	2.678614	1.429137	C	2.401633	1.968769	1.746803
C	-2.24634	0.20873	3.028126	H	1.882403	1.438315	2.543044
H	-3.02472	-1.57494	2.142128	C	3.846878	1.998415	-0.23885
H	-1.34682	2.072254	3.696782	H	4.602862	1.522648	-0.86479
H	-2.58968	-0.0743	4.020433	H	3.05185	2.39229	-0.88097
C	-1.35806	1.688795	-2.06323	C	4.464184	0.524178	1.691544
H	-1.60636	0.88119	-2.75271	H	5.114035	-0.13958	1.117912
C	-2.29136	1.936994	-1.00653	H	4.127848	-0.01004	2.58233
H	-3.14483	1.266249	-0.96567	C	3.288717	1.066537	0.857865
N	-2.30741	-1.05399	-0.49728	P	2.160974	-0.2809	0.048219
S	-3.24563	-2.37919	-0.50807	C	1.846135	-1.70263	1.31839
O	-3.22119	-2.90219	-1.89216	H	1.653001	2.489547	1.144654
O	-2.93242	-3.35514	0.5709	C	3.054943	-2.61803	1.588863
C	-4.93353	-1.80285	-0.20284	H	3.354126	-3.17778	0.700318
H	-4.97605	-1.29014	0.760318	H	3.925388	-2.07755	1.963283
H	-5.59414	-2.67307	-0.19282	H	2.770371	-3.35367	2.35262
H	-5.21761	-1.12105	-1.00666	H	4.325958	2.85573	0.249967
H	-0.82659	2.536533	-2.49845	H	3.039296	2.728142	2.217201
C	-2.54455	3.303132	-0.49369	H	5.07495	1.369804	2.033457
C	-3.72064	3.56528	0.098033	H	2.742328	-2.5871	-2.9314
H	-4.47684	2.794132	0.223418	H	3.34856	-0.44604	-3.57471
H	-3.96066	4.557428	0.471032	H	4.955023	-1.76477	-2.22766
C	-1.48079	4.354072	-0.68552	C	1.383825	-1.07323	2.649923
H	-1.70611	5.254008	-0.10768	H	1.061702	-1.88038	3.318997
H	-1.39727	4.640734	-1.74108	H	2.180386	-0.53142	3.1625
H	-0.49519	3.981204	-0.38352	H	0.531491	-0.40354	2.513088
C	2.894091	-0.00977	-2.6762	C	0.671198	-2.57465	0.816474
H	1.833645	0.161741	-2.91269	H	-0.18941	-1.98382	0.506775
H	3.366554	0.956365	-2.509	H	0.948006	-3.22738	-0.0102
C	3.067557	-1.00519	-1.50198	H	0.34625	-3.22227	1.640077

TS2-2s P^{t}Bu$_3$ trans 2-selective TS

		Hartree
Zero-point correction	=	0.619932
Thermal correction to Energy	=	0.656648
Thermal correction to Enthalpy	=	0.657592
Thermal correction to Gibbs Free Energy	=	0.553953
E(RwB97XD)	=	-2012.47994363
		cm^{-1}
Lowest energy vibration	=	-322.24

Second lowest energy vibration = 14.85

0 1							
Pd	0.213562	1.092577	-0.10766	C	0.461887	-2.4648	-1.89517
C	-1.26423	0.636115	1.316642	H	-0.45398	-1.9854	-1.53528
C	-1.01838	2.854592	-0.48868	H	0.628513	-3.38424	-1.33212
C	-1.72021	2.629593	0.711975	C	2.929524	-2.26002	-2.31462
C	-0.97209	0.715515	2.684885	H	3.790497	-1.59972	-2.43489
C	-2.31569	-0.19313	0.810813	H	3.210952	-3.06957	-1.63978
H	-1.50944	2.564408	-1.41629	C	3.432888	1.14065	1.290015
C	0.258016	3.560657	-0.6064	H	3.229384	0.674505	2.25316
H	-1.41527	3.135402	1.621168	C	3.849132	0.970051	-1.1362
H	-2.76963	2.371403	0.645082	H	4.065858	0.358646	-2.01305
C	-1.69867	-0.0355	3.608398	H	3.044427	1.668097	-1.38139
H	-0.17974	1.377777	3.022495	C	4.706905	-0.82858	0.36764
C	-3.04468	-0.93538	1.783268	H	4.817061	-1.56755	-0.42847
N	-2.49333	-0.18035	-0.55218	H	4.626137	-1.35676	1.318921
C	0.963678	3.401454	-1.76054	C	3.528221	0.132306	0.11941
C	0.795133	4.362018	0.555737	P	1.797839	-0.7254	-0.07899
C	-2.73593	-0.85413	3.138162	C	1.653407	-2.13165	1.240074
H	-1.46599	0.018286	4.66777	H	2.655792	1.88956	1.104428
H	-3.82651	-1.60987	1.454178	C	2.487278	-3.38913	0.92411
S	-3.75466	-0.91008	-1.25728	H	2.116872	-3.91669	0.042726
H	0.545963	2.866935	-2.60932	H	3.547369	-3.17899	0.778716
H	1.942091	3.853805	-1.8886	H	2.402843	-4.08072	1.772181
H	1.799334	4.736176	0.34191	H	4.746365	1.566041	-0.92842
H	0.140647	5.217114	0.761203	H	4.392544	1.665945	1.376043
H	0.8406	3.762976	1.470855	H	5.633944	-0.24211	0.402399
H	-3.31326	-1.44807	3.842979	H	0.293975	-2.75252	-2.94038
O	-3.98113	-2.31494	-0.81645	H	1.253678	-0.83486	-3.85928
O	-3.61099	-0.70153	-2.71627	H	2.725007	-2.70683	-3.2961
C	-5.2223	0.019597	-0.7463	C	2.074802	-1.58107	2.619795
H	-5.12086	1.048722	-1.09623	H	1.822946	-2.33248	3.378142
H	-6.10079	-0.44879	-1.1965	H	3.146183	-1.39282	2.69783
H	-5.30519	-0.00198	0.342464	H	1.532779	-0.66636	2.876211
C	1.350684	-0.38159	-2.86468	C	0.1731	-2.54757	1.390234
H	0.396543	0.103364	-2.63164	H	-0.43051	-1.7252	1.763522
H	2.120074	0.38601	-2.92987	H	-0.2812	-2.91707	0.473546
C	1.670017	-1.5052	-1.84836	H	0.122335	-3.35744	2.129035

VI-3s Migratory insertion 3 selectivity sigma complex

		Hartree
Zero-point correction	=	0.620971
Thermal correction to Energy	=	0.657800
Thermal correction to Enthalpy	=	0.658744
Thermal correction to Gibbs Free Energy	=	0.554481
E(RwB97XD)	=	-2012.49590945
		cm^{-1}
Lowest energy vibration	=	17.28
Second lowest energy vibration	=	26.26

0 1							
Pd	0.029901	0.701304	-0.58037	C	2.251265	-1.94304	-2.38218
C	-1.85661	1.163133	0.525611	H	1.161062	-1.85885	-2.32354
C	-2.36611	-0.21353	0.601659	H	2.558016	-2.86968	-1.89585
C	-1.17551	1.718336	1.658403	C	4.479381	-0.89932	-1.89321
C	-2.04111	-0.95136	1.788378	H	5.034485	0.003565	-1.6312
C	-0.95172	0.982888	2.804503	H	4.828314	-1.71283	-1.25448
H	-0.87718	2.759412	1.624303	C	2.633416	1.56267	2.032062
C	-1.38357	-0.36059	2.847516	H	2.394801	0.847931	2.817858
H	-2.3317	-1.99103	1.858073	C	3.694859	2.117141	-0.11906
H	-0.44031	1.425977	3.652389	H	4.358321	1.843493	-0.94069
H	-1.18756	-0.95411	3.73693	H	2.770663	2.533697	-0.5347
C	-1.43558	1.60475	-1.6817	C	4.752092	0.383378	1.35421
H	-1.79554	0.77604	-2.29551	H	5.345812	-0.06903	0.557736
C	-2.40822	2.062501	-0.61837	H	4.61666	-0.35915	2.143209
H	-3.42892	1.731289	-0.83022	C	3.411758	0.946044	0.845723
N	-3.15035	-0.61723	-0.42088	P	2.259719	-0.3297	-0.03553
S	-3.70529	-2.14999	-0.54541	C	2.230599	-1.97617	0.973893
O	-4.33705	-2.26127	-1.87611	H	1.699612	2.023645	1.698768
O	-2.68629	-3.1766	-0.20203	C	3.483895	-2.85852	0.821179
C	-5.03388	-2.29296	0.669666	H	3.606682	-3.23993	-0.19404
H	-4.6395	-2.1139	1.671629	H	4.400121	-2.33608	1.104045
H	-5.44248	-3.30407	0.601437	H	3.383788	-3.72911	1.482455
H	-5.8029	-1.55693	0.428204	H	4.191243	2.917482	0.44337
H	-0.87871	2.367025	-2.23096	H	3.253839	2.347441	2.483325
C	-2.41889	3.549962	-0.30359	H	5.343322	1.204181	1.780748
C	-3.52744	4.111111	0.193702	H	2.517856	-2.03172	-3.4426
H	-4.43479	3.533467	0.356663	H	2.868931	0.235401	-3.76137

H	-3.56499	5.165846	0.456051	H	4.746283	-1.15445	-2.92715
C	-1.17215	4.362779	-0.56607	C	2.040659	-1.65313	2.470799
H	-1.22978	5.341979	-0.08246	H	1.84199	-2.59132	3.0035
H	-1.03461	4.530331	-1.64187	H	2.927758	-1.20795	2.924083
H	-0.26661	3.853863	-0.21559	H	1.186894	-0.99354	2.641547
C	2.547877	0.473092	-2.73918	C	0.979135	-2.77763	0.545583
H	1.455941	0.600258	-2.76837	H	0.064388	-2.19395	0.670693
H	2.994151	1.429033	-2.47152	H	1.018919	-3.12888	-0.48437
C	2.955914	-0.69509	-1.80783	H	0.897894	-3.66469	1.186904

VII-2s Migratory insertion 2 selectivity pi complex

		Hartree
Zero-point correction	=	0.623761
Thermal correction to Energy	=	0.659870
Thermal correction to Enthalpy	=	0.660814
Thermal correction to Gibbs Free Energy	=	0.560019
E(RwB97XD)	=	-2012.56928718
		cm^{-1}
Lowest energy vibration	=	23.74
Second lowest energy vibration	=	26.44

0 1							
Pd	-0.1098	0.632525	-0.71316	C	2.507276	-2.44575	-1.27548
C	-3.37612	0.919659	0.240973	H	1.426563	-2.45262	-1.4344
C	-1.82469	1.606518	-1.72892	H	2.747095	-3.09977	-0.43501
C	-3.22858	1.442316	-1.18255	C	4.574622	-1.13138	-0.79274
C	-4.27981	1.531018	1.117304	H	5.08086	-0.16543	-0.77818
C	-2.67199	-0.22986	0.673884	H	4.777896	-1.63741	0.152389
H	-1.62926	1.133961	-2.69097	C	2.073258	2.46313	1.51921
C	-0.94477	2.632086	-1.31986	H	1.64899	2.0769	2.445276
H	-3.75007	2.405546	-1.24908	C	3.651049	2.208916	-0.38084
H	-3.7644	0.759095	-1.84956	H	4.483667	1.666022	-0.82914
C	-4.51808	1.017281	2.395543	H	2.918756	2.413341	-1.15964
H	-4.8121	2.420285	0.786988	C	4.203356	1.173922	1.808457
C	-2.92869	-0.74894	1.95479	H	4.927077	0.468121	1.397232
N	-1.66981	-0.79593	-0.15791	H	3.859215	0.794921	2.771536
C	0.398992	2.500482	-1.76006	C	3.045209	1.473522	0.83466
C	-1.33682	3.642895	-0.27072	P	2.027257	-0.08208	0.250325

C	-3.84747	-0.1356	2.808223	C	1.814642	-1.24254	1.793336
H	-5.22094	1.513876	3.058774	H	1.247493	2.740379	0.862374
H	-2.39821	-1.64384	2.260092	C	3.094177	-1.98137	2.235347
S	-1.94289	-2.24264	-0.82925	H	3.416167	-2.71701	1.495352
H	0.610868	2.078899	-2.74144	H	3.933053	-1.31924	2.44693
H	1.131504	3.208027	-1.38939	H	2.871303	-2.53201	3.158565
H	-0.46184	4.158735	0.130943	H	4.043713	3.173782	-0.03707
H	-1.99643	4.393723	-0.72347	H	2.62553	3.377506	1.770525
H	-1.88653	3.18272	0.554164	H	4.737724	2.112017	2.006652
H	-4.02716	-0.55125	3.796372	H	2.981209	-2.88257	-2.1642
O	-2.32626	-3.27345	0.168247	H	3.415089	-0.84545	-3.21587
O	-0.79602	-2.5533	-1.71495	H	5.040543	-1.72653	-1.58879
C	-3.38722	-2.05709	-1.90354	C	1.272911	-0.40819	2.975036
H	-3.14138	-1.35689	-2.70304	H	0.967831	-1.09782	3.771733
H	-3.63016	-3.0383	-2.31801	H	2.014068	0.267414	3.403754
H	-4.22434	-1.68585	-1.30874	H	0.389085	0.172456	2.690948
C	2.861807	-0.29799	-2.4422	C	0.726237	-2.2967	1.50192
H	1.805452	-0.29024	-2.72891	H	-0.23526	-1.8256	1.314751
H	3.223676	0.729687	-2.44931	H	0.948175	-2.94094	0.654454
C	3.065975	-1.01916	-1.08932	H	0.628614	-2.93889	2.387001

VIII-3s Migratory insertion 3 selectivity chelate complex

		Hartree
Zero-point correction	=	0.624304
Thermal correction to Energy	=	0.660331
Thermal correction to Enthalpy	=	0.661275
Thermal correction to Gibbs Free Energy	=	0.559644
E(RwB97XD)	=	-2012.54899477
		cm^{-1}
Lowest energy vibration	=	17.78
Second lowest energy vibration	=	27.58

0 1							
Pd	-0.06152	-0.44281	-0.18211	C	-2.82405	-0.92503	2.477337
C	3.160417	0.91956	-0.2536	H	-1.83123	-1.37513	2.377772
C	3.039642	-0.4209	-0.69649	H	-3.57653	-1.65924	2.186737
C	4.302712	1.640641	-0.62684	C	-4.37997	0.927813	1.817275
C	4.037412	-0.97565	-1.51725	H	-4.49201	1.933638	1.408378

C	5.297382	1.079085	-1.43067	H	-5.11145	0.277905	1.33429
H	4.433536	2.654299	-0.26291	C	-2.03662	1.613653	-2.51955
C	5.160787	-0.23486	-1.88081	H	-2.21112	0.746512	-3.15442
H	3.918255	-2.00118	-1.84728	C	-2.59621	2.947535	-0.5091
H	6.170896	1.666733	-1.6995	H	-3.21911	3.101612	0.372709
H	5.92292	-0.68371	-2.51241	H	-1.54838	3.049705	-0.22282
C	0.672776	1.446031	0.008737	C	-4.38138	1.599904	-1.61489
H	-0.03178	2.001414	0.622405	H	-5.04116	1.589641	-0.74549
C	2.074895	1.511292	0.642212	H	-4.64249	0.757109	-2.25637
H	2.056929	0.923297	1.567106	C	-2.88818	1.611767	-1.2286
N	1.900805	-1.18707	-0.33589	P	-2.35309	0.110379	-0.1285
S	2.1191	-2.48593	0.633998	C	-3.17599	-1.50693	-0.82125
O	0.783412	-3.12021	0.776077	H	-0.96788	1.665819	-2.30246
O	3.23659	-3.34781	0.190194	C	-4.68002	-1.64995	-0.52149
C	2.582505	-1.84833	2.258189	H	-4.88004	-1.75978	0.546263
H	3.486438	-1.24532	2.147783	H	-5.27164	-0.81464	-0.89774
H	2.774951	-2.6964	2.91932	H	-5.04546	-2.56039	-1.01371
H	1.759125	-1.24203	2.638769	H	-2.82227	3.761656	-1.20828
H	0.673803	1.849417	-1.0075	H	-2.30242	2.503485	-3.10363
C	2.345556	2.961783	1.048334	H	-4.59816	2.514456	-2.1811
C	2.785346	3.249871	2.278876	H	-2.98119	-0.70488	3.540181
H	2.964819	2.471899	3.017718	H	-2.31283	1.529209	3.415615
H	2.984119	4.273878	2.587819	H	-4.63883	0.980758	2.882311
C	2.06686	4.04383	0.030292	C	-2.95625	-1.56848	-2.34839
H	2.479699	5.005286	0.349303	H	-3.26472	-2.56097	-2.69843
H	0.988002	4.173843	-0.11834	H	-3.55098	-0.83731	-2.89699
H	2.487752	3.793081	-0.94947	H	-1.90199	-1.43894	-2.61369
C	-1.96936	1.38289	2.384118	C	-2.4392	-2.73946	-0.24065
H	-0.95542	0.975457	2.426717	H	-1.36785	-2.74043	-0.46824
H	-1.92729	2.365623	1.915764	H	-2.54962	-2.84906	0.836003
C	-2.93855	0.397749	1.690022	H	-2.8656	-3.63742	-0.70535

VIII-2s Migratory insertion 2 selectivity chelate complex

		Hartree
Zero-point correction	=	0.623910
Thermal correction to Energy	=	0.660078
Thermal correction to Enthalpy	=	0.661022
Thermal correction to Gibbs Free Energy	=	0.558705
E(RwB97XD)	=	-2012.550404
		cm^{-1}

Lowest energy vibration	=	12.71
Second lowest energy vibration	=	22.82

0 1							
Pd	0.111327	-0.14021	-0.21725	H	-3.47369	2.067847	1.991938
C	3.241272	1.075071	0.686744	H	-1.89903	2.616175	1.386122
C	3.286213	0.01378	-0.24495	C	-4.68806	1.58291	-0.40847
C	4.339854	1.936588	0.786922	H	-5.20293	1.014638	0.367304
C	4.437189	-0.16989	-1.02953	H	-4.92216	1.136934	-1.37645
C	5.484343	1.747769	0.008438	C	-3.17015	1.697873	-0.16265
H	4.295803	2.764889	1.490201	P	-2.2487	0.004186	-0.03071
C	5.531825	0.685271	-0.89752	C	-2.87388	-1.14929	-1.46274
H	4.455262	-0.99067	-1.7375	H	-1.46188	2.577682	-1.23235
H	6.327523	2.426024	0.10593	C	-4.26989	-1.75677	-1.22701
H	6.414431	0.527821	-1.512	H	-4.28346	-2.45278	-0.38652
C	0.696715	1.584321	0.780407	H	-5.03963	-1.00214	-1.06016
H	-0.07521	1.743258	1.533173	H	-4.55504	-2.32476	-2.12182
C	2.009497	1.280226	1.531881	H	-3.36393	3.509101	0.988018
H	1.84564	0.4104	2.178602	H	-2.93746	3.540896	-1.26036
N	2.133291	-0.79453	-0.41513	H	-5.11099	2.595621	-0.40996
S	2.25505	-2.374	-0.01095	H	-2.3077	-2.71443	2.648965
O	0.921589	-2.97309	-0.27662	H	-2.01011	-0.65445	3.724666
O	3.427521	-3.03157	-0.62982	H	-4.26354	-1.30892	3.026678
C	2.519889	-2.44016	1.773764	C	-2.88864	-0.36413	-2.79104
H	3.421965	-1.8733	2.013719	H	-3.04632	-1.07972	-3.60715
H	2.64598	-3.48624	2.061875	H	-3.69591	0.367053	-2.84587
H	1.650131	-2.00707	2.269369	H	-1.93613	0.141947	-2.97722
C	-1.77985	-0.17177	2.76668	C	-1.857	-2.29691	-1.66863
H	-0.71569	-0.3227	2.564317	H	-0.88425	-1.9264	-2.00937
H	-1.95624	0.895941	2.891082	H	-1.68372	-2.90713	-0.78675
C	-2.65642	-0.8266	1.672558	H	-2.24221	-2.95397	-2.45898
C	-2.23646	-2.31288	1.630575	H	2.185307	2.135514	2.199586
H	-1.20274	-2.44192	1.295177	C	0.77799	2.784159	-0.12875
H	-2.88725	-2.92137	1.000751	C	0.351436	3.973669	0.325978
C	-4.13493	-0.74072	2.096696	H	0.434188	4.875689	-0.27643
H	-4.44939	0.284483	2.300851	H	-0.08282	4.088027	1.315987
H	-4.81269	-1.16778	1.35596	C	1.362839	2.652223	-1.51588
C	-2.54592	2.517473	-1.3152	H	0.744145	1.984121	-2.12765
H	-2.79961	2.12892	-2.2997	H	1.417124	3.624133	-2.01586
C	-2.9544	2.502822	1.137035	H	2.366056	2.218285	-1.49306

NMR Spectra of New Compounds

N-(2-bromophenyl)-4-(trifluoromethyl)benzenesulfonamide (1k)

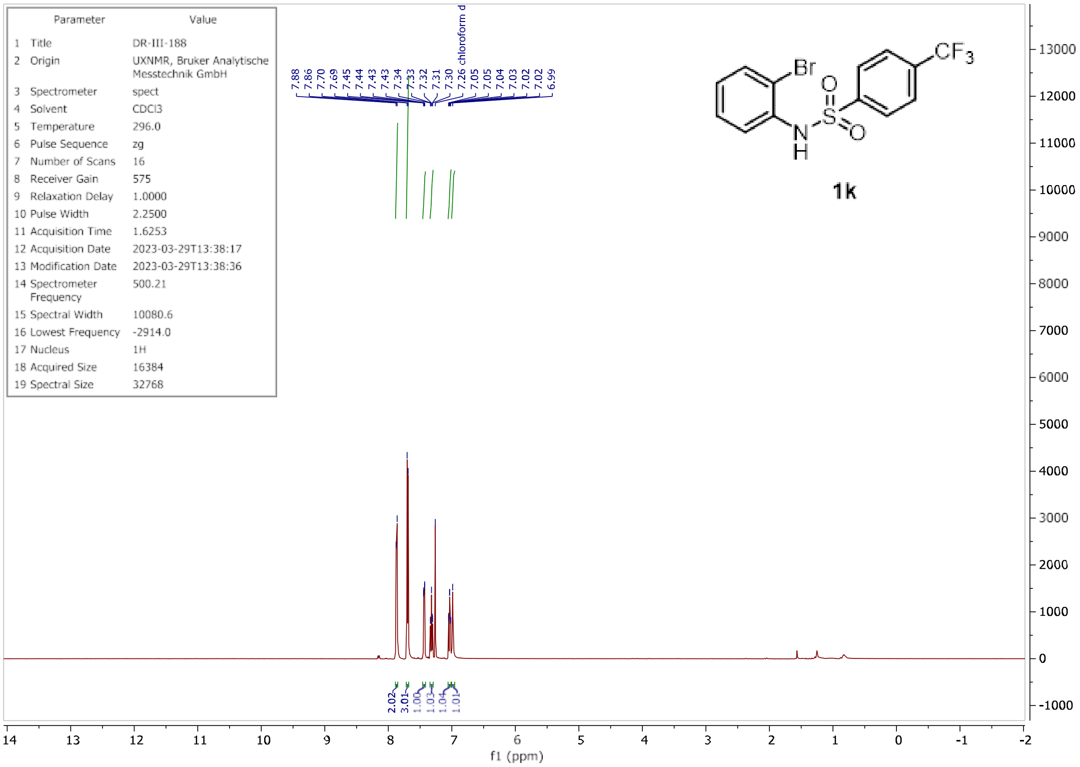

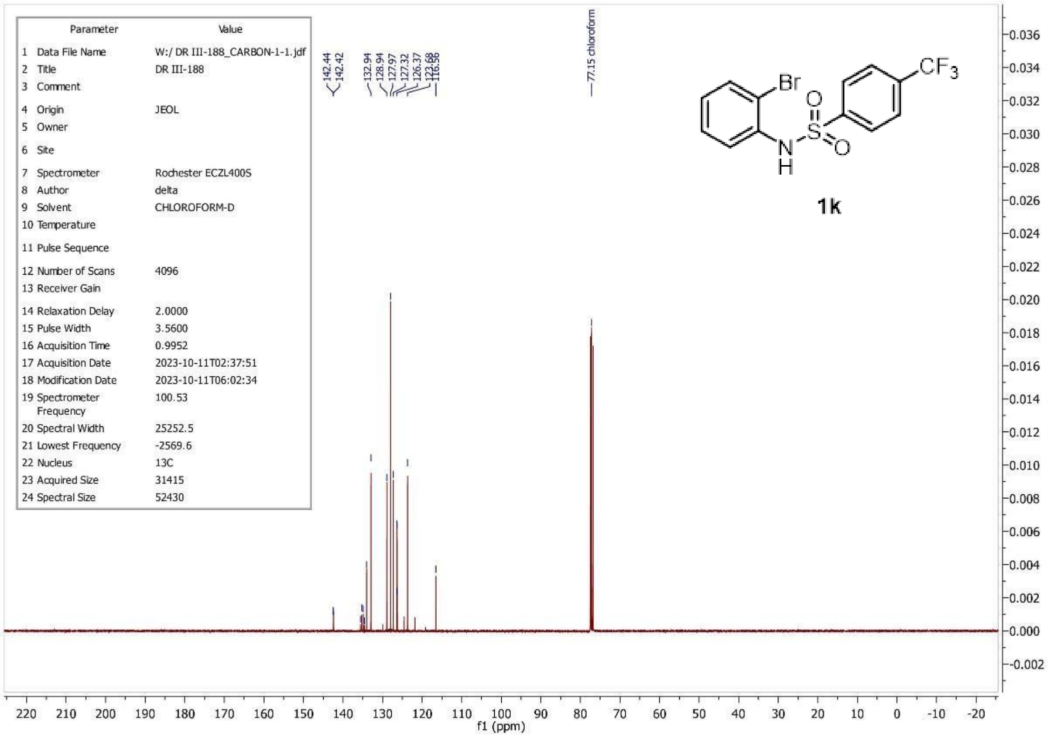

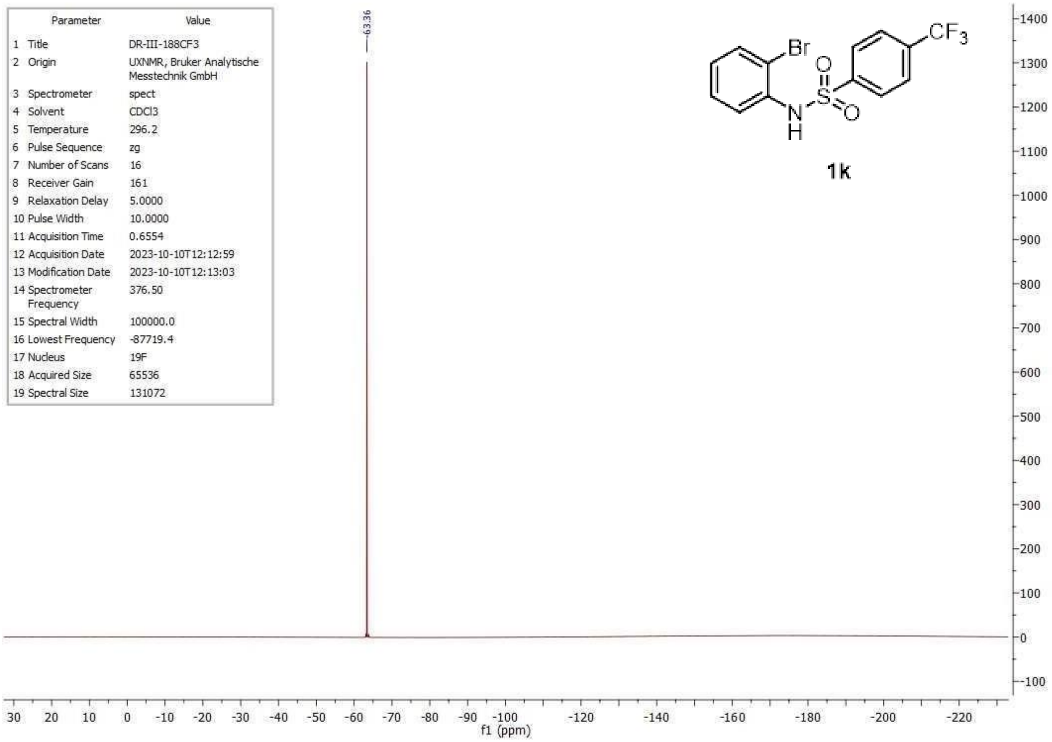
Parameter Value
1 Title DR-III-188CF3
2 Origin UXNMR, Bruker Analytische Messtechnik GmbH
3 Spectrometer spect
4 Solvent CDCl3
5 Temperature 296.2
6 Pulse Sequence zg
7 Number of Scans 16
8 Receiver Gain 161
9 Relaxation Delay 5.0000
10 Pulse Width 10.0000
11 Acquisition Time 0.6554
12 Acquisition Date 2023-10-10T12:12:59
13 Modification Date 2023-10-10T12:13:03
14 Spectrometer Frequency 376.50
15 Spectral Width 100000.0
16 Lowest Frequency -87719.4
17 Nucleus 19F
18 Acquired Size 65536
19 Spectral Size 131072
-63.36
Br
CF3
N
H
S
O
O
1k
f1 (ppm)

2-(3-((4-methylenehex-5-en-1-yl)oxy)propyl)furan (2i)

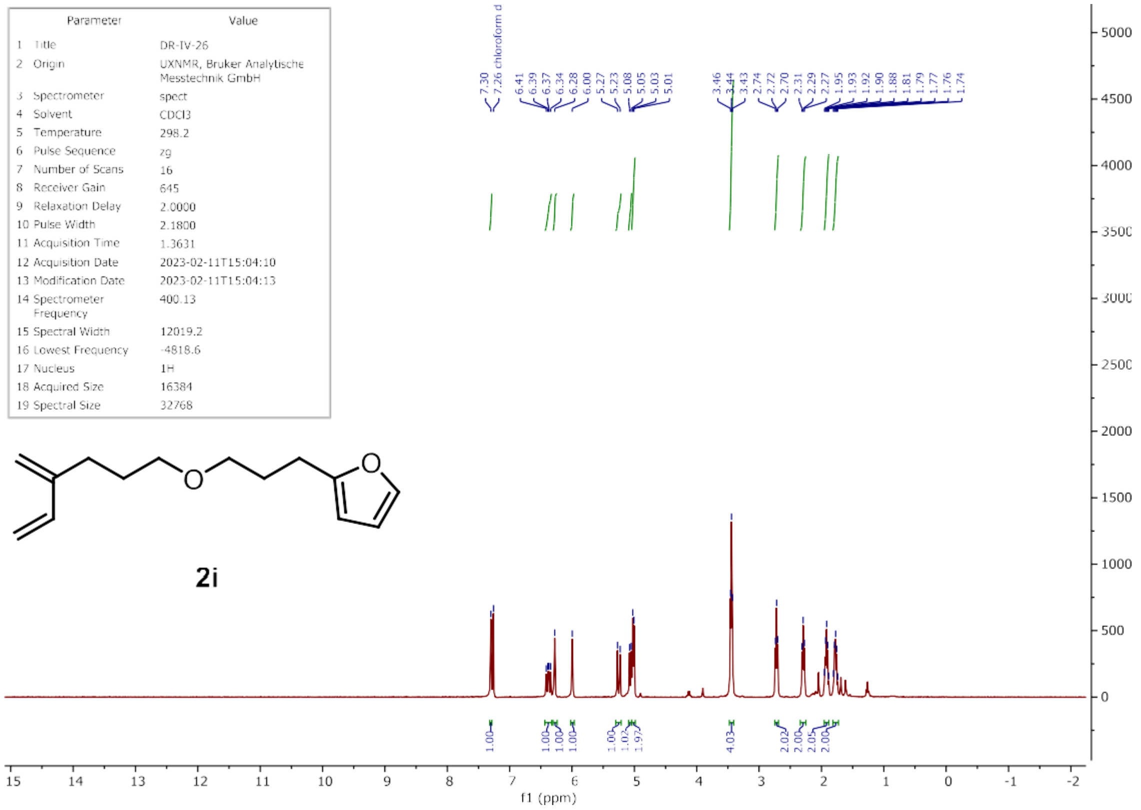

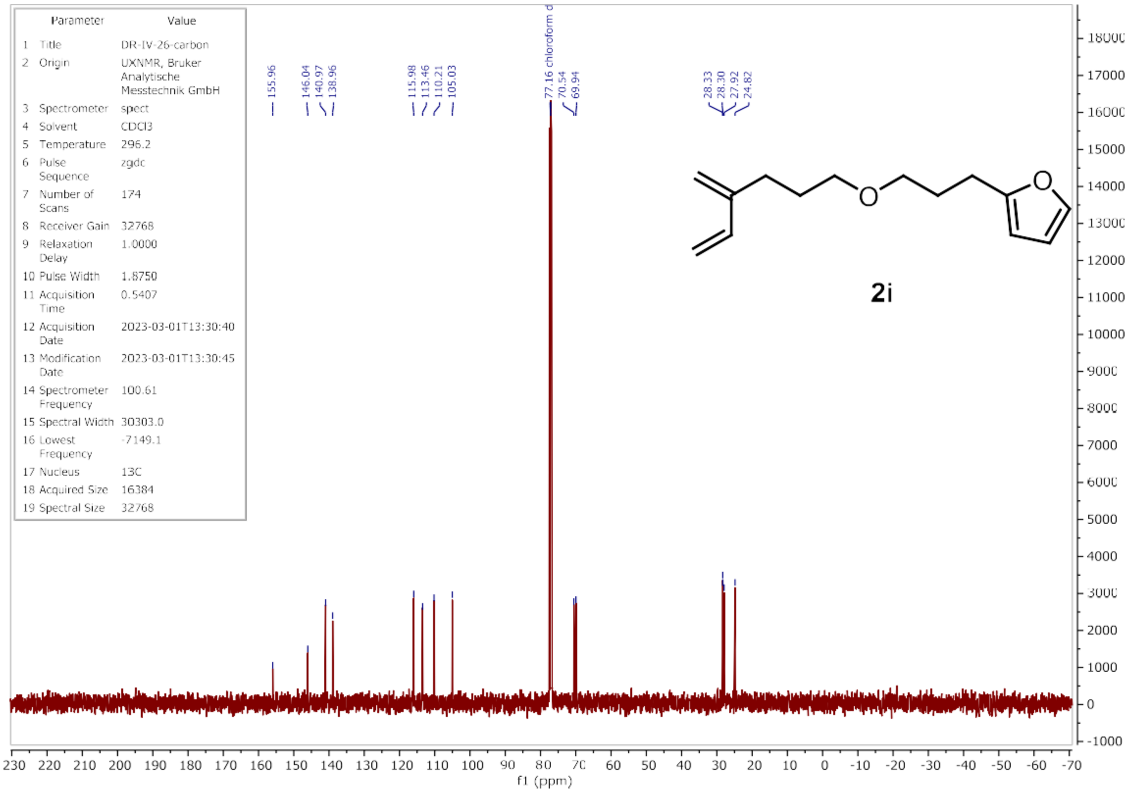

1-methyl-3-(((4-methylenehex-5-en-1-yl)oxy)methyl)-1*H*-indole (2j)

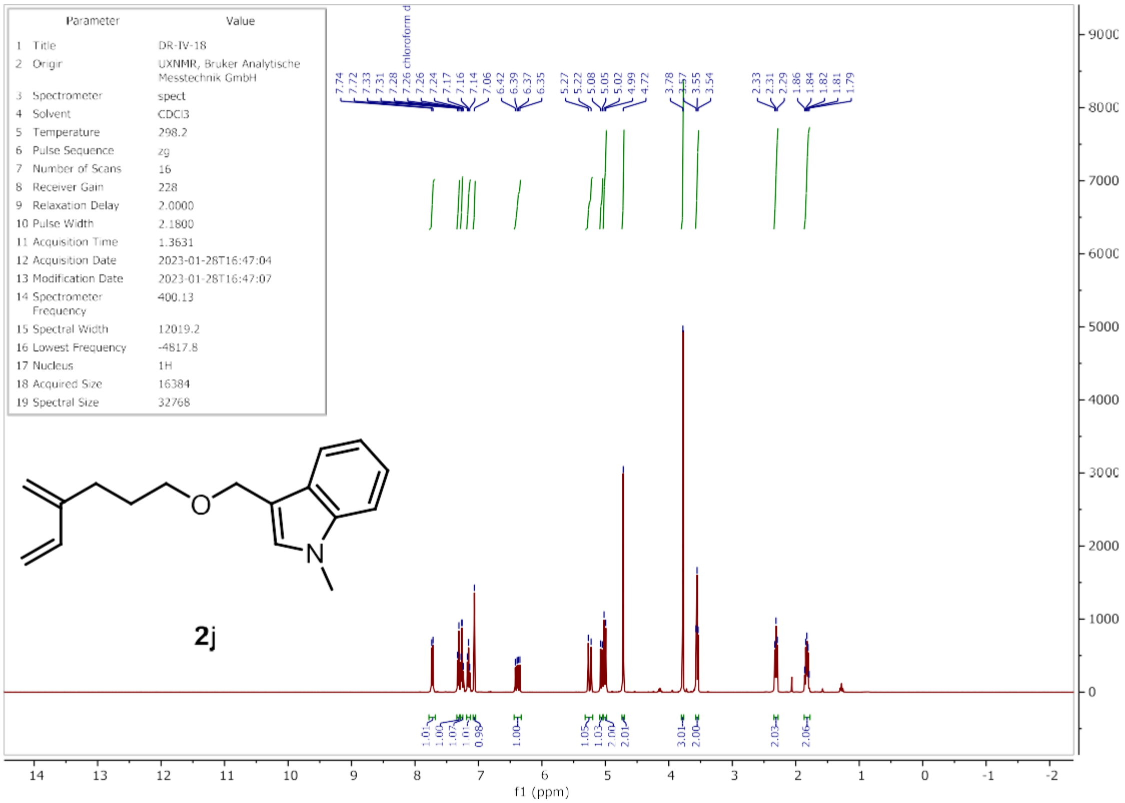

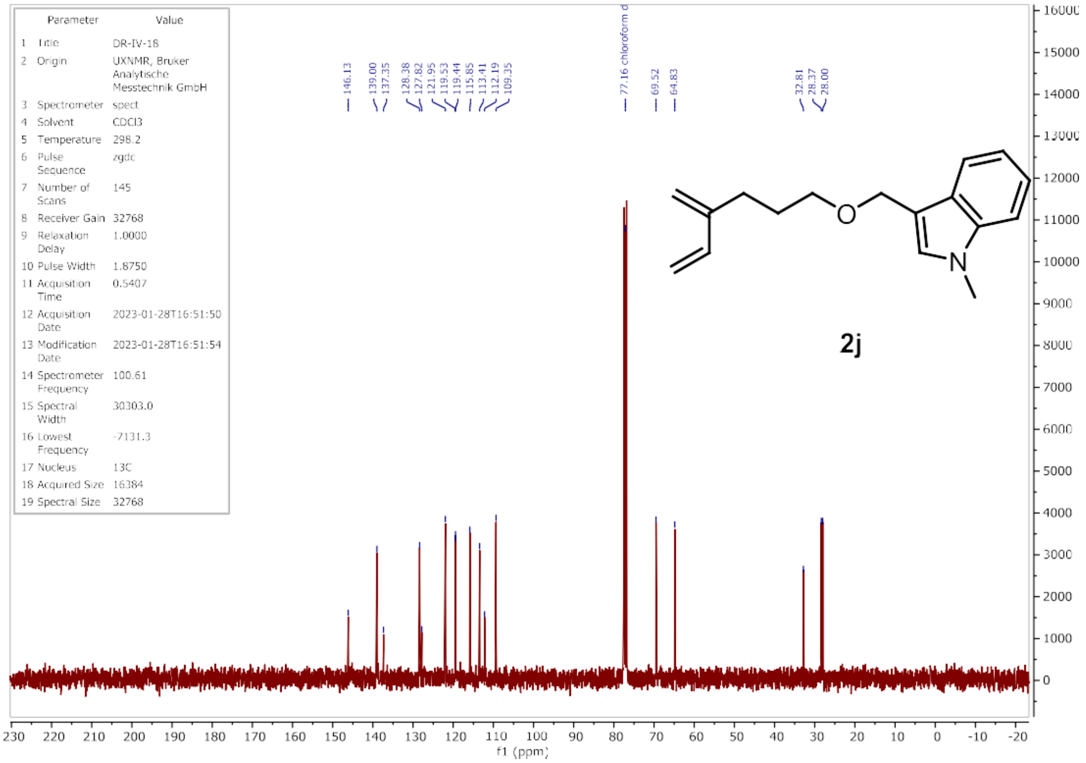

3-(6-methylhepta-1,5-dien-2-yl)-*N*-tosylindoline (3a)

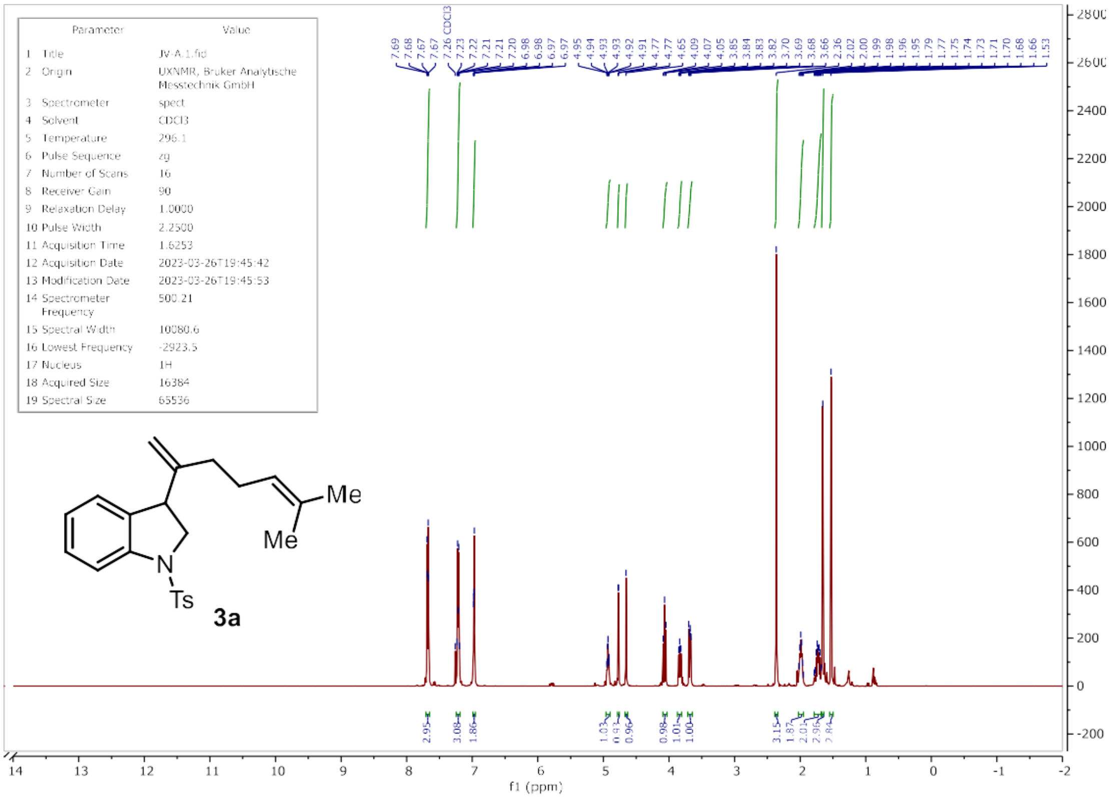

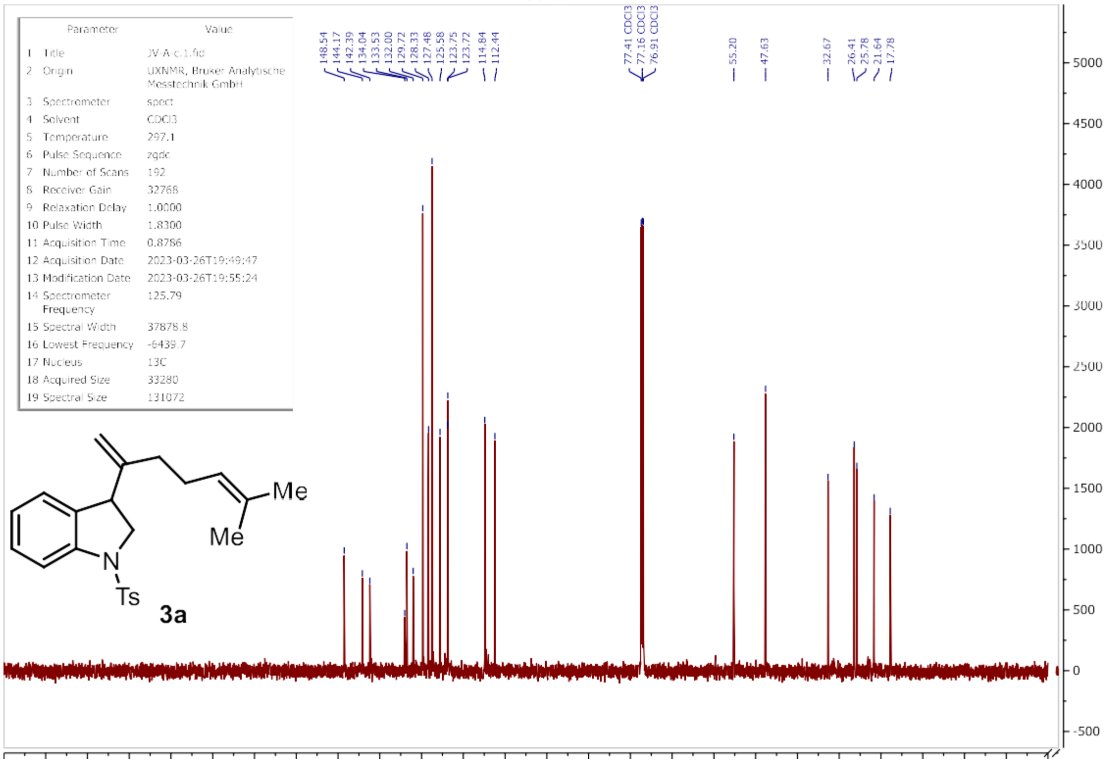

3-(6-methylhepta-1,5-dien-2-yl)-*N*-tosyl-5-(trifluoromethyl)indoline (3b)

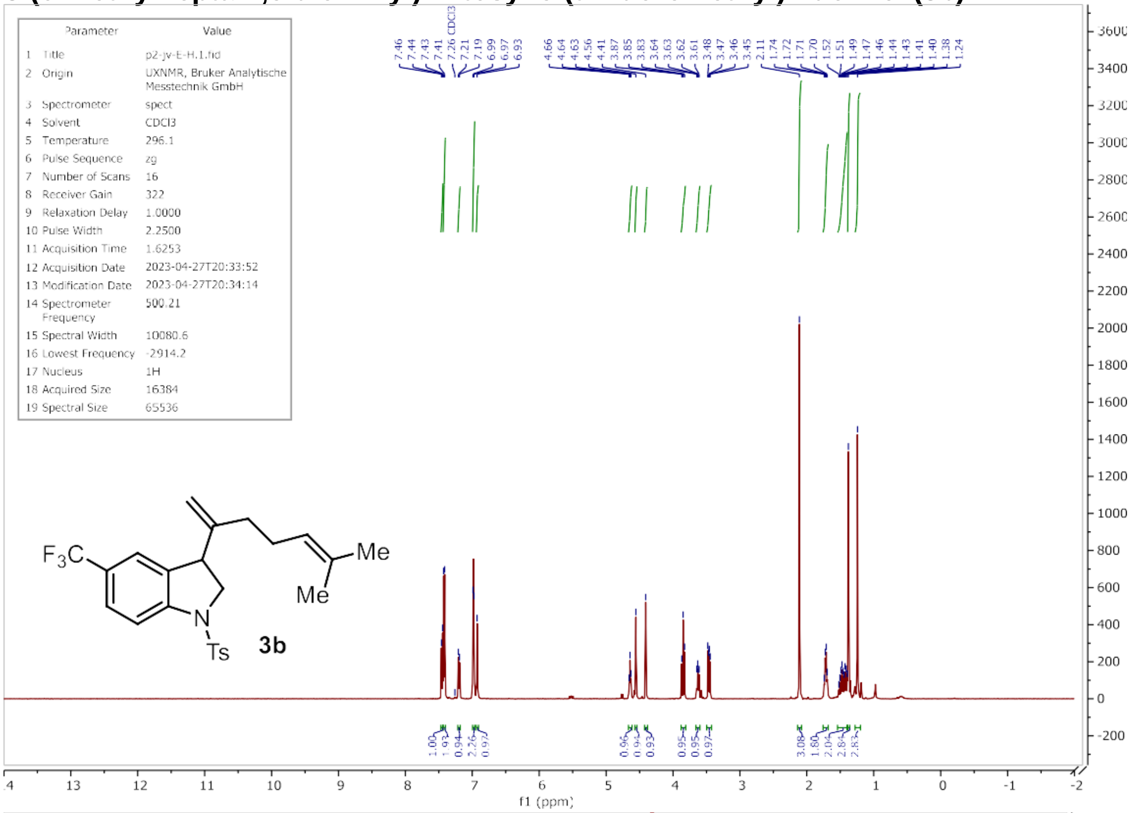

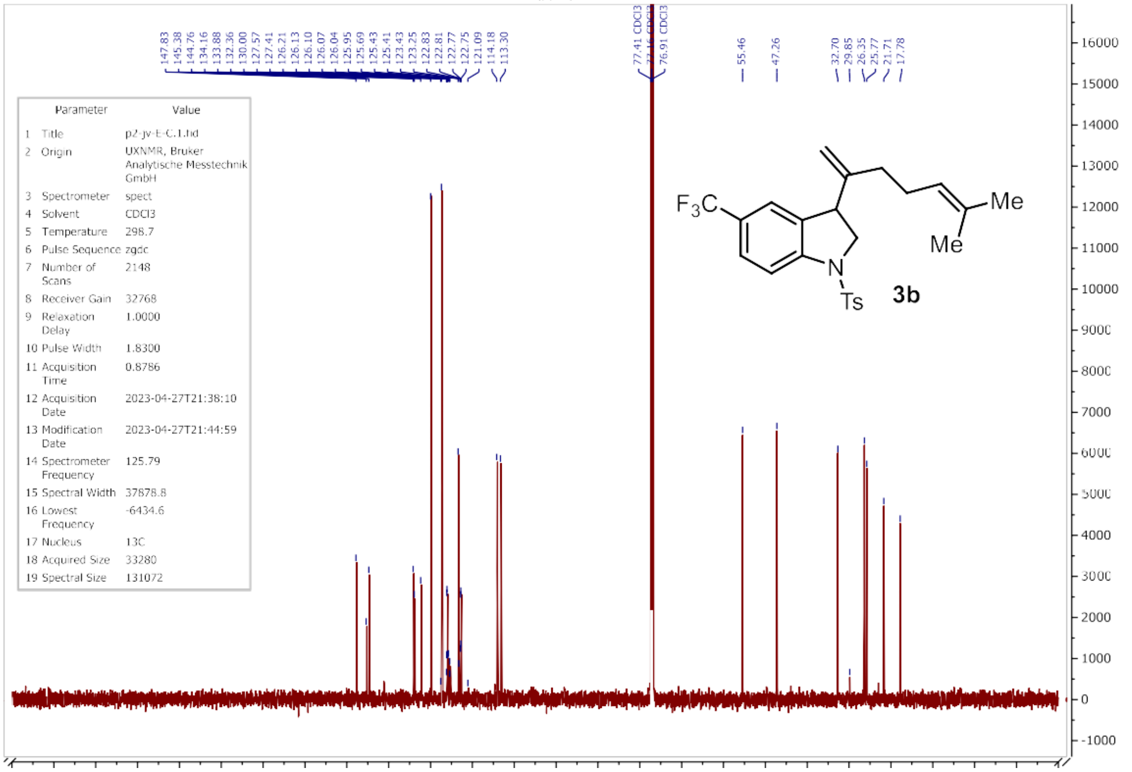

3-(6-methylhepta-1,5-dien-2-yl)-*N*-tosyl-5-(trifluoromethyl)indoline (3b)

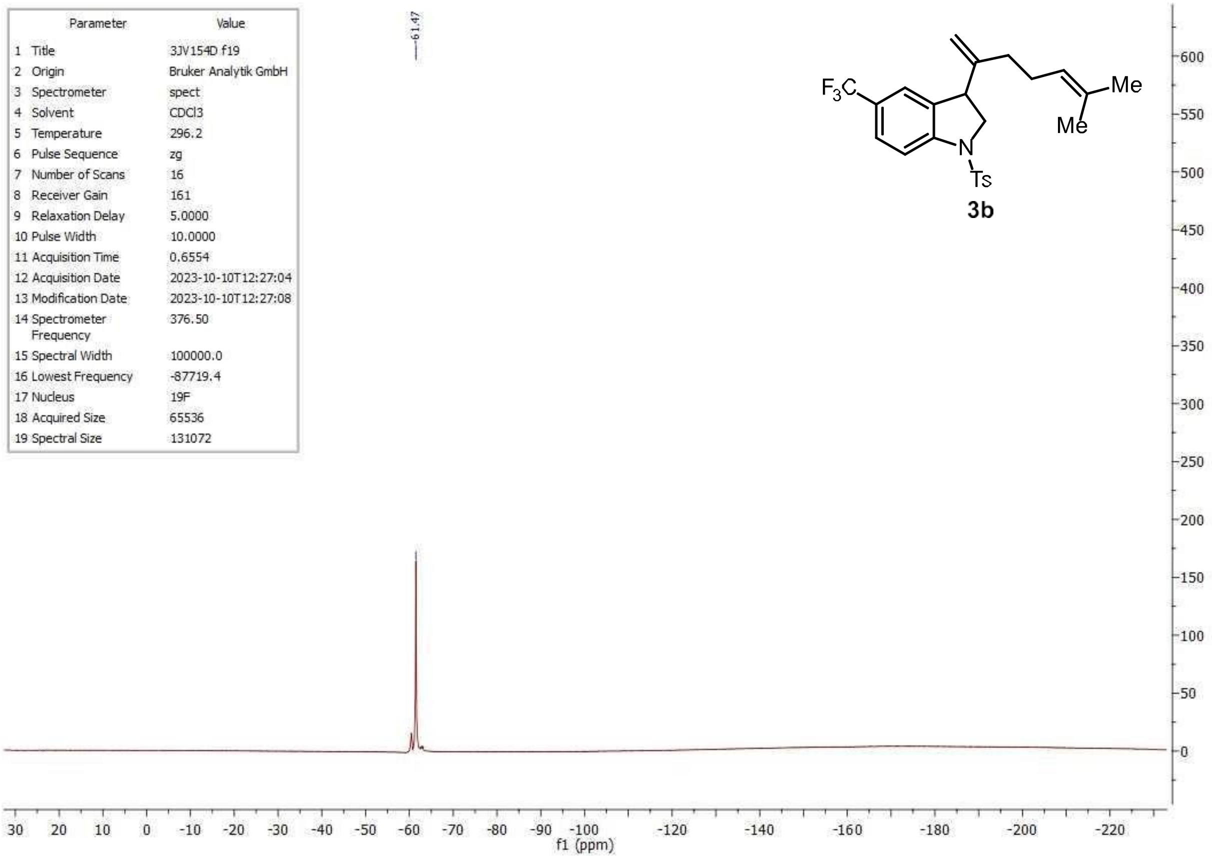

3-(6-methylhepta-1,5-dien-2-yl)-*N*-tosyl-6-(trifluoromethyl)indoline (3c)

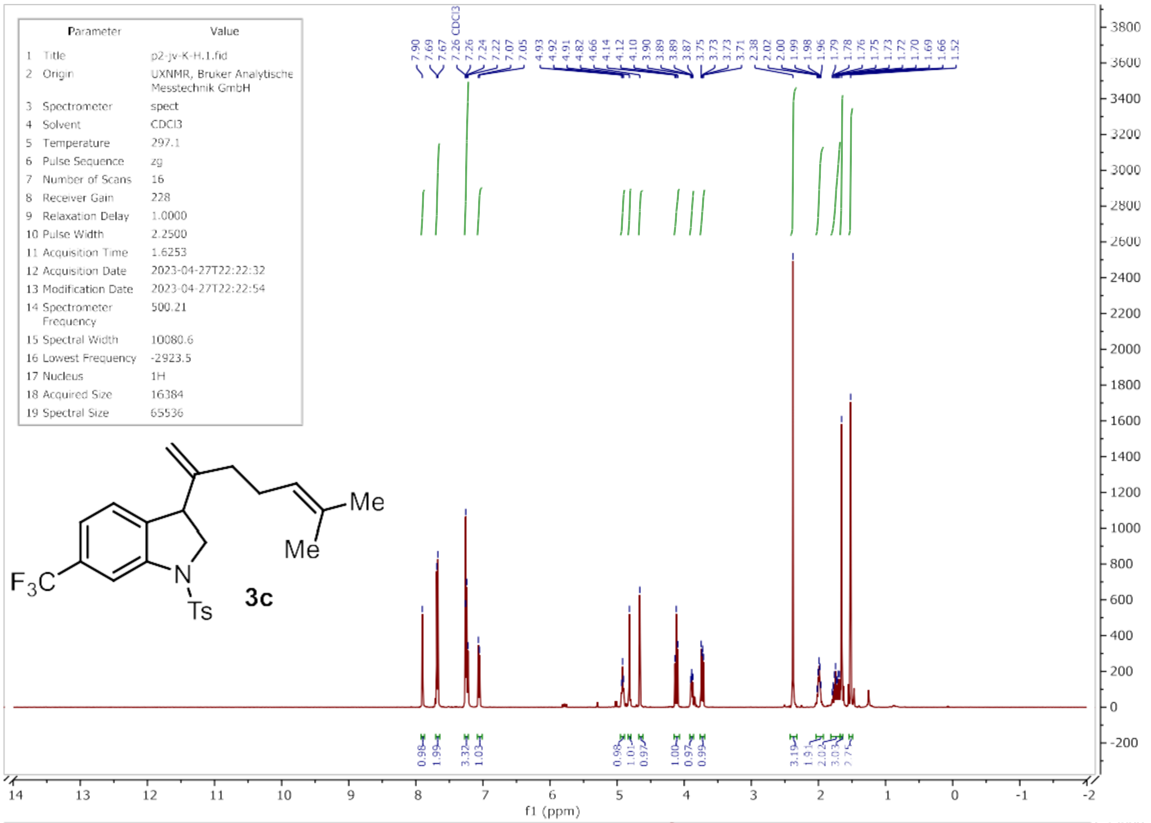

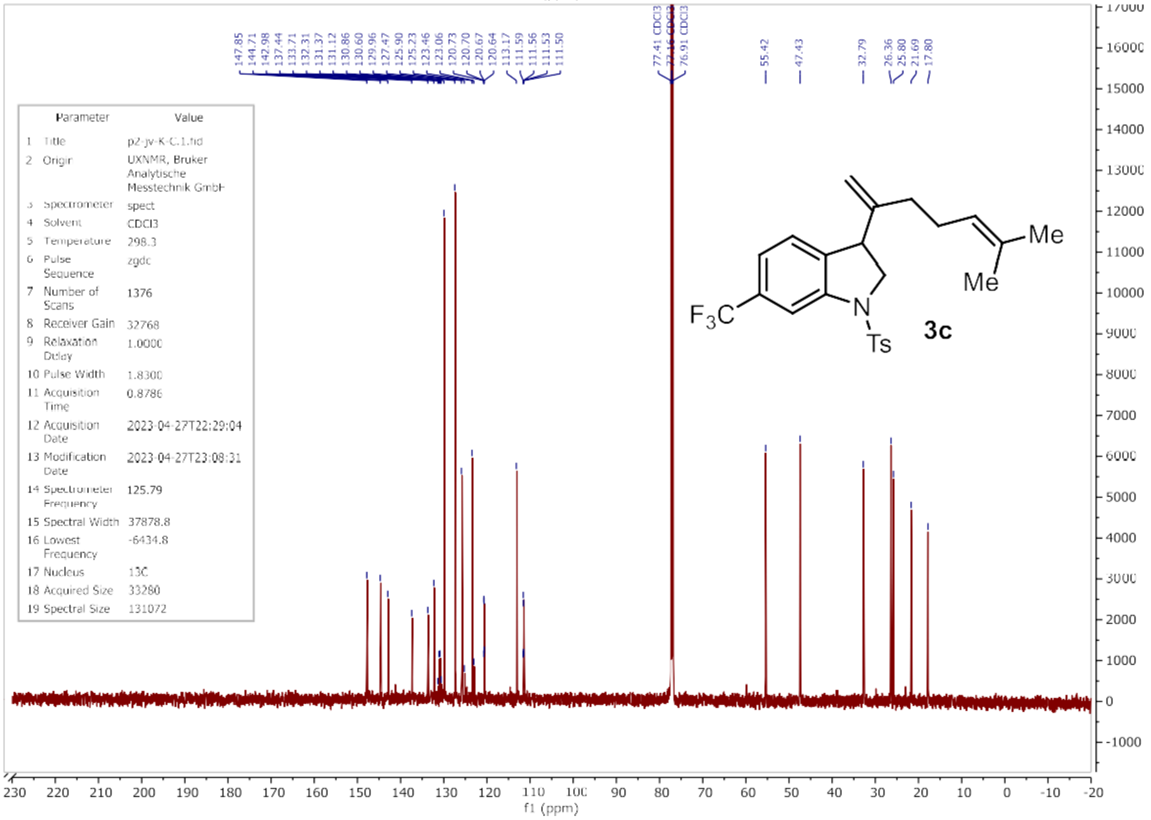

3-(6-methylhepta-1,5-dien-2-yl)-*N*-tosyl-6-(trifluoromethyl)indoline (3c)

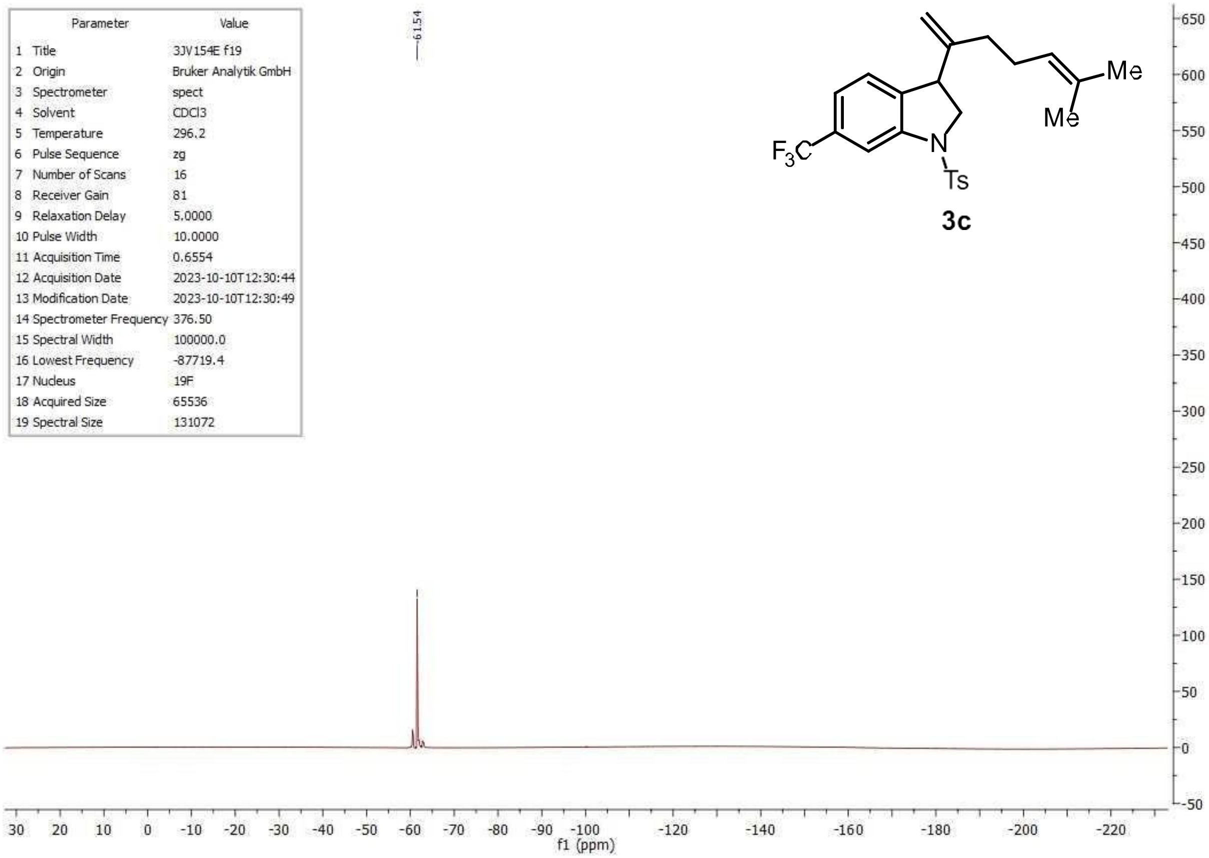

5-chloro-3-(6-methylhepta-1,5-dien-2-yl)-*N*-tosylindoline (3d)

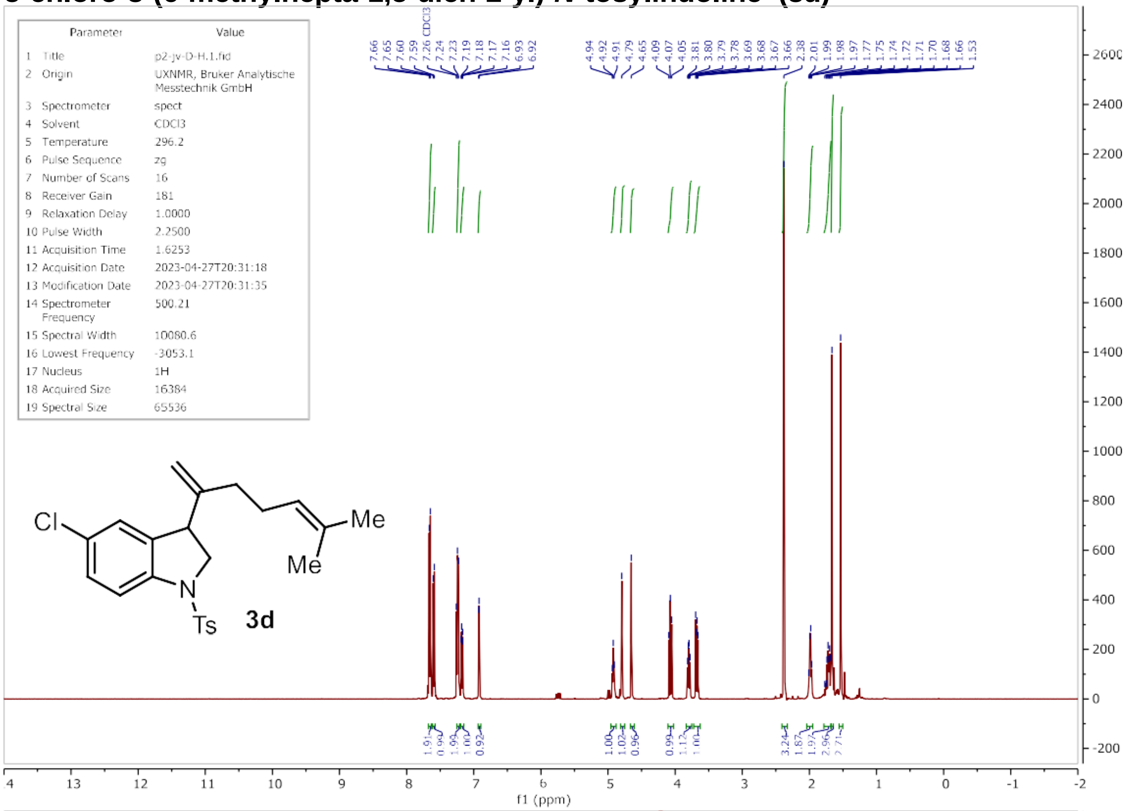

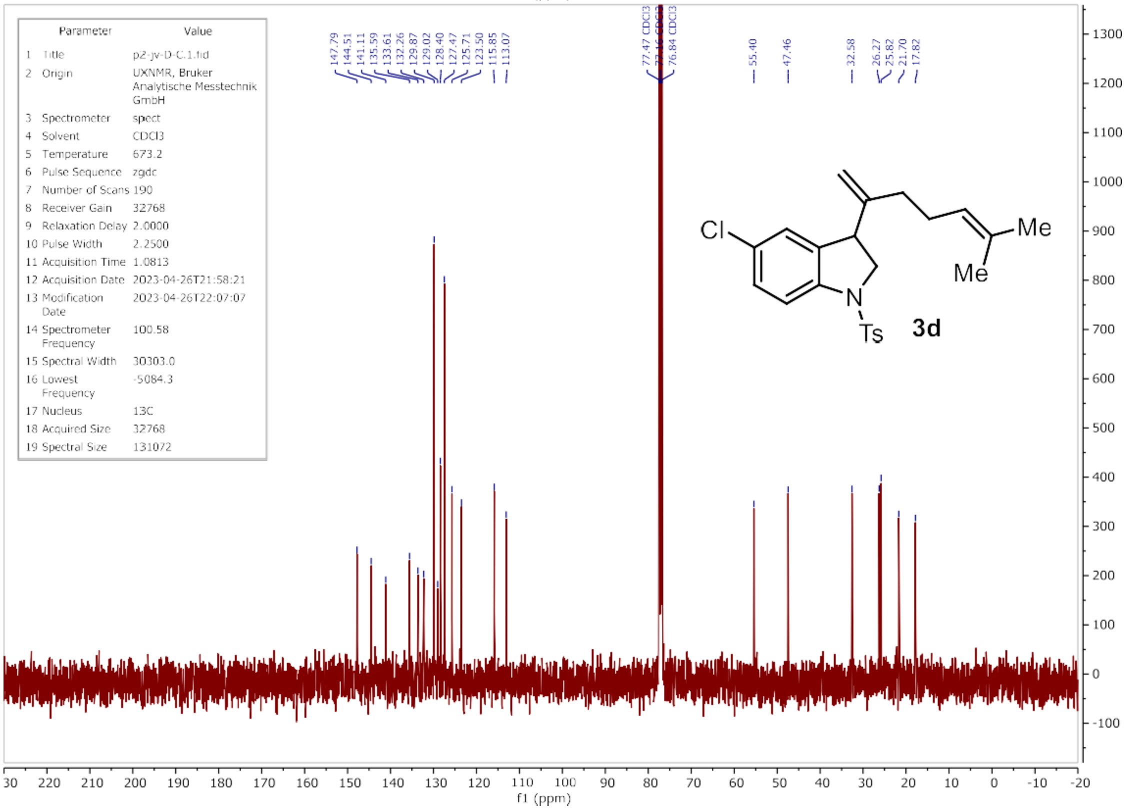

6-chloro-3-(6-methylhepta-1,5-dien-2-yl)-*N*-tosylindoline (3e)

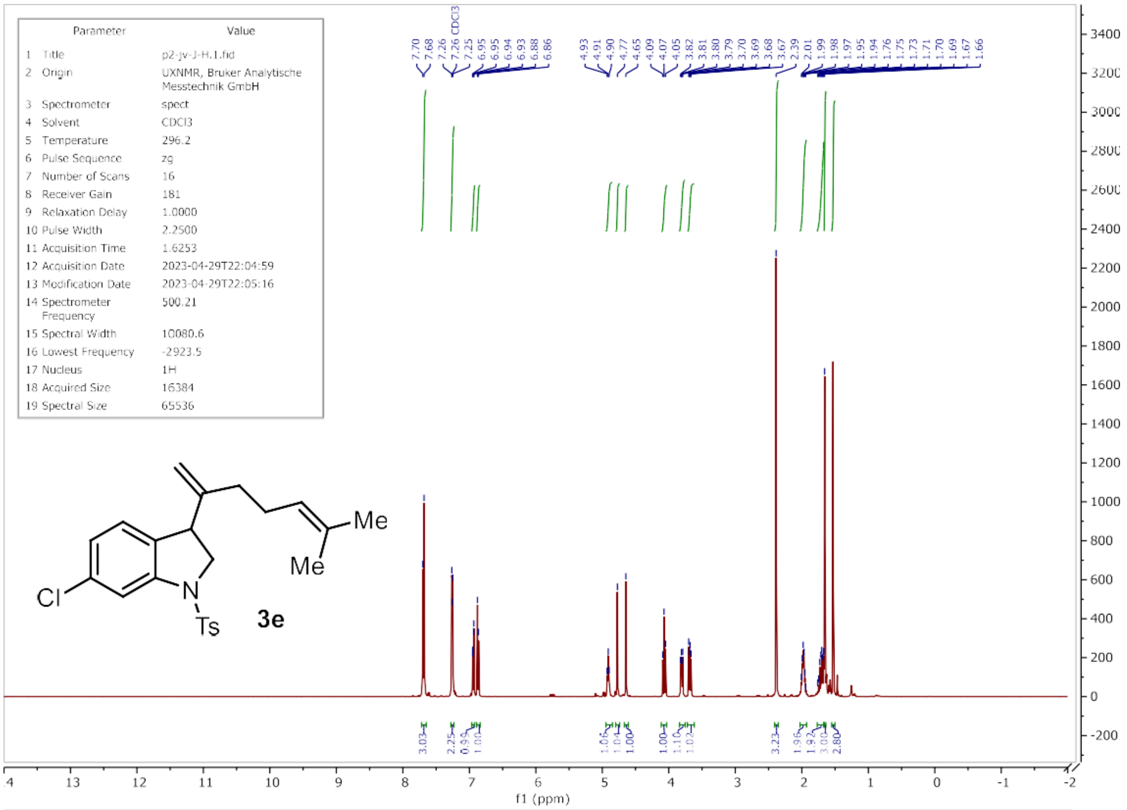

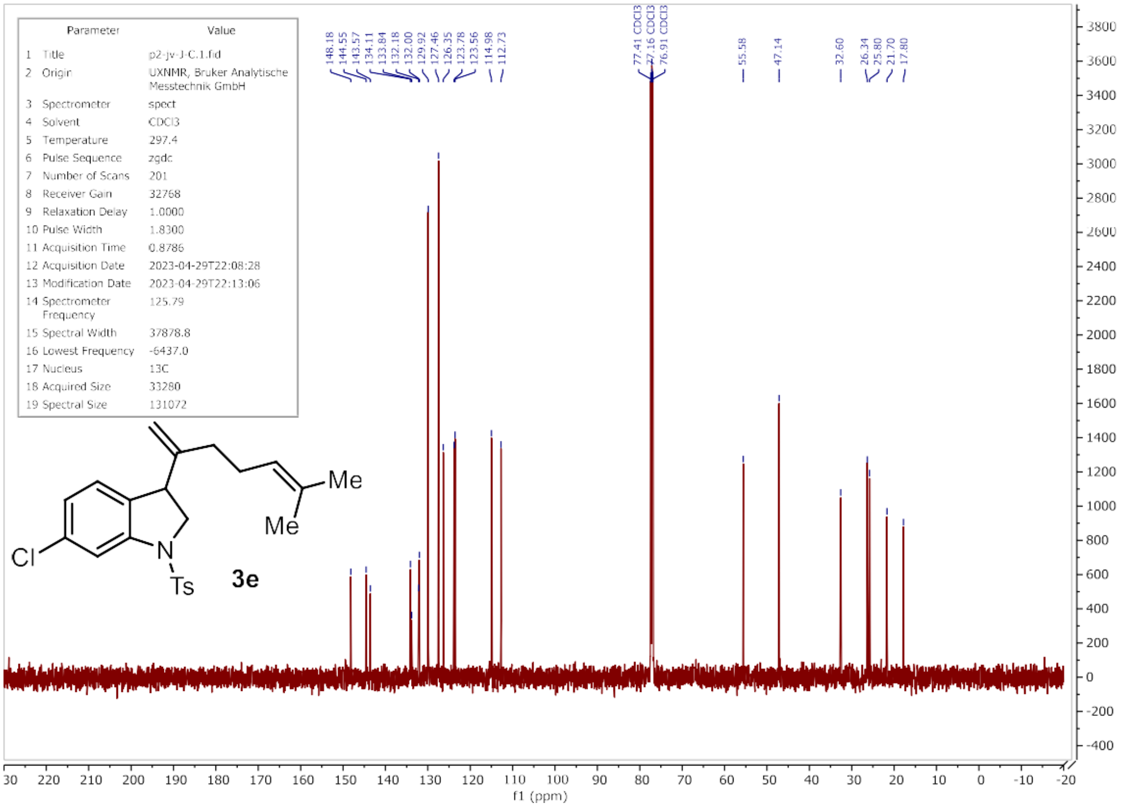

5-methoxy-3-(6-methylhepta-1,5-dien-2-yl)-*N*-tosylindoline (3f)

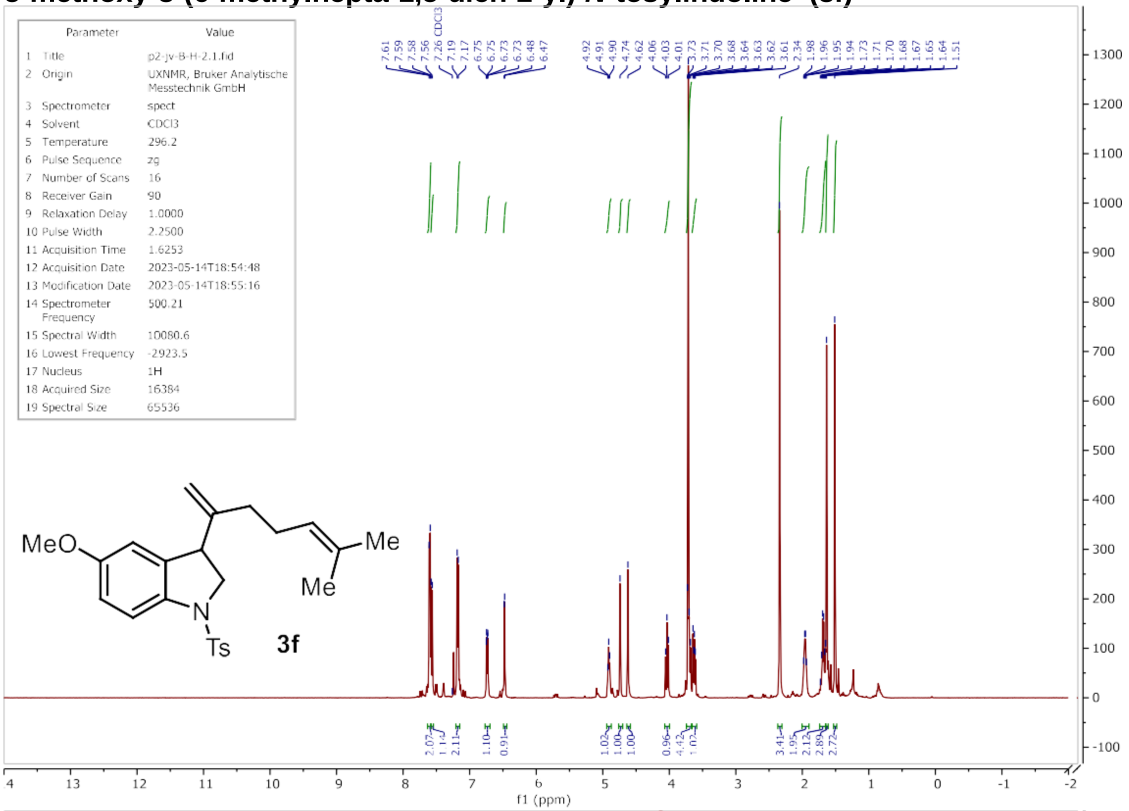

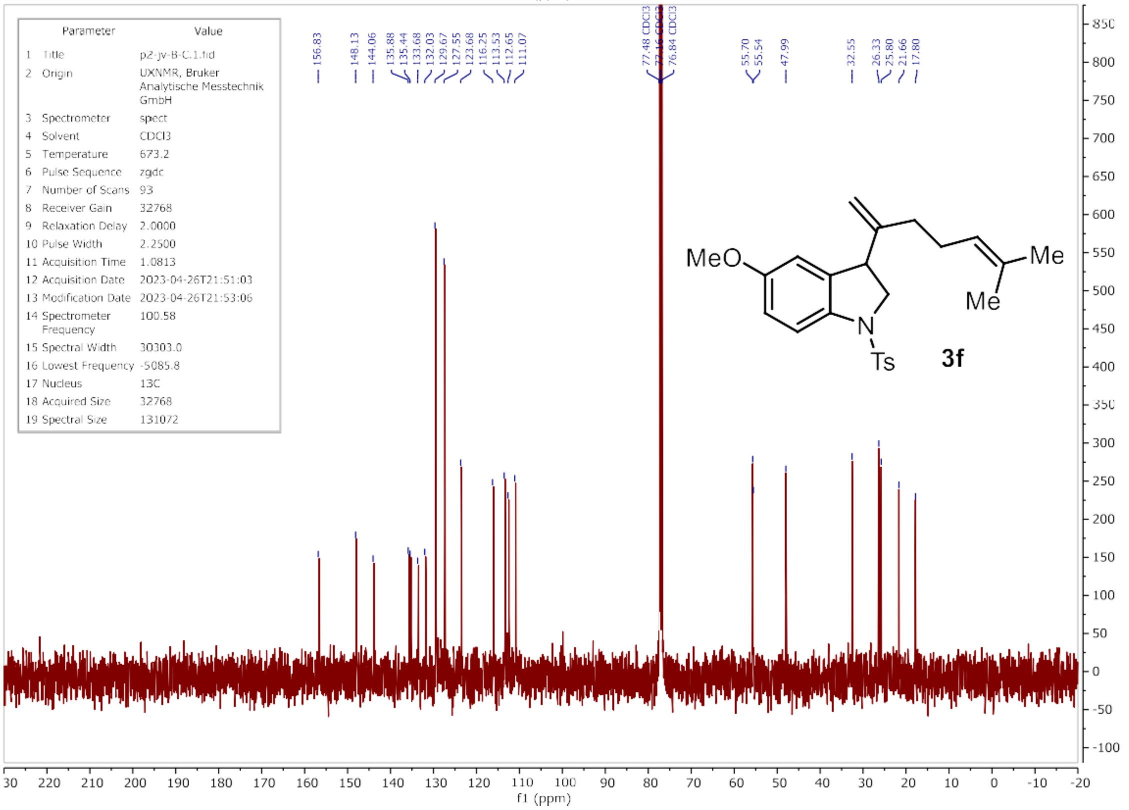

5-methyl-3-(6-methylhepta-1,5-dien-2-yl)-*N*-tosylindoline (3g)

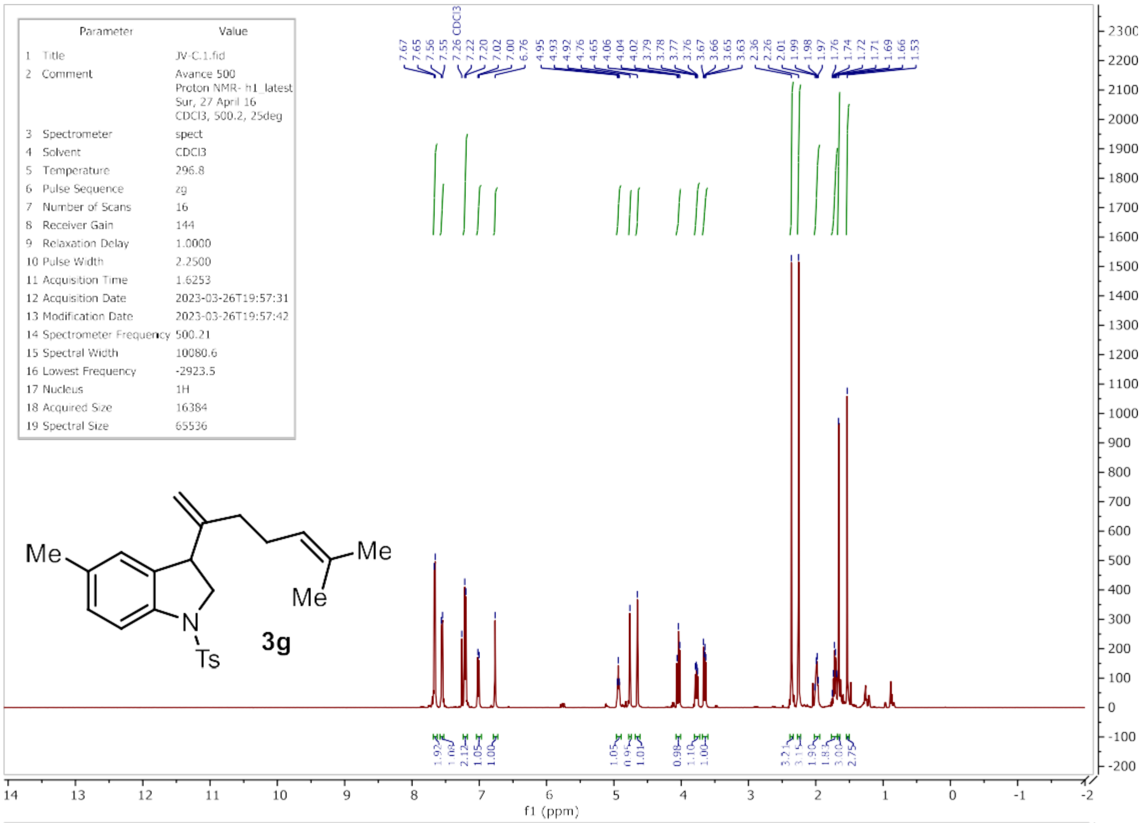

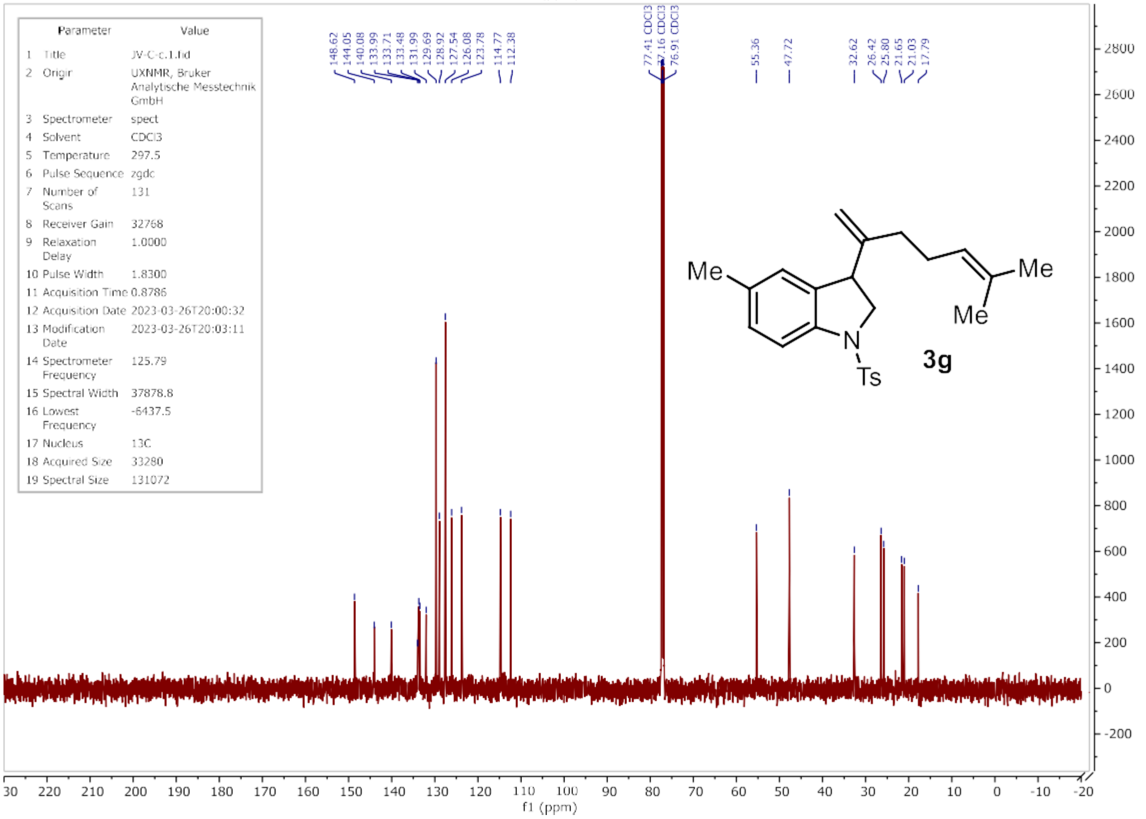

6-methyl-3-(6-methylhepta-1,5-dien-2-yl)-*N*-tosylindoline (3h)

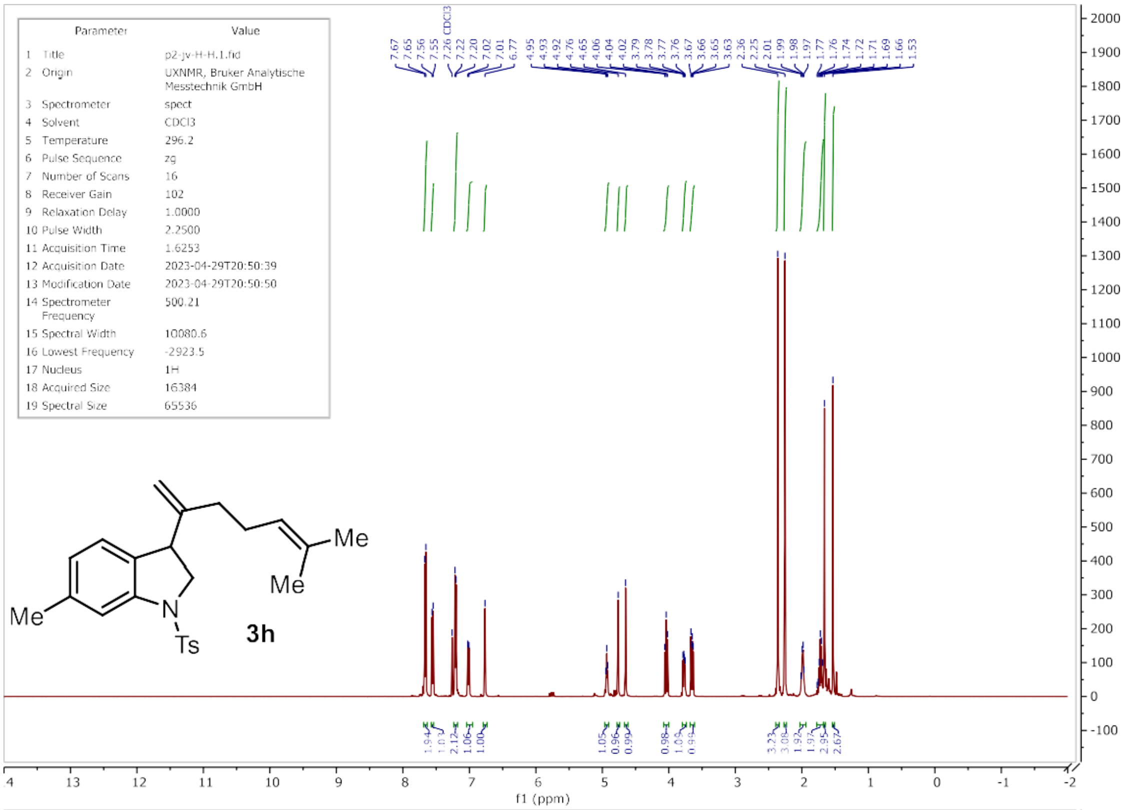

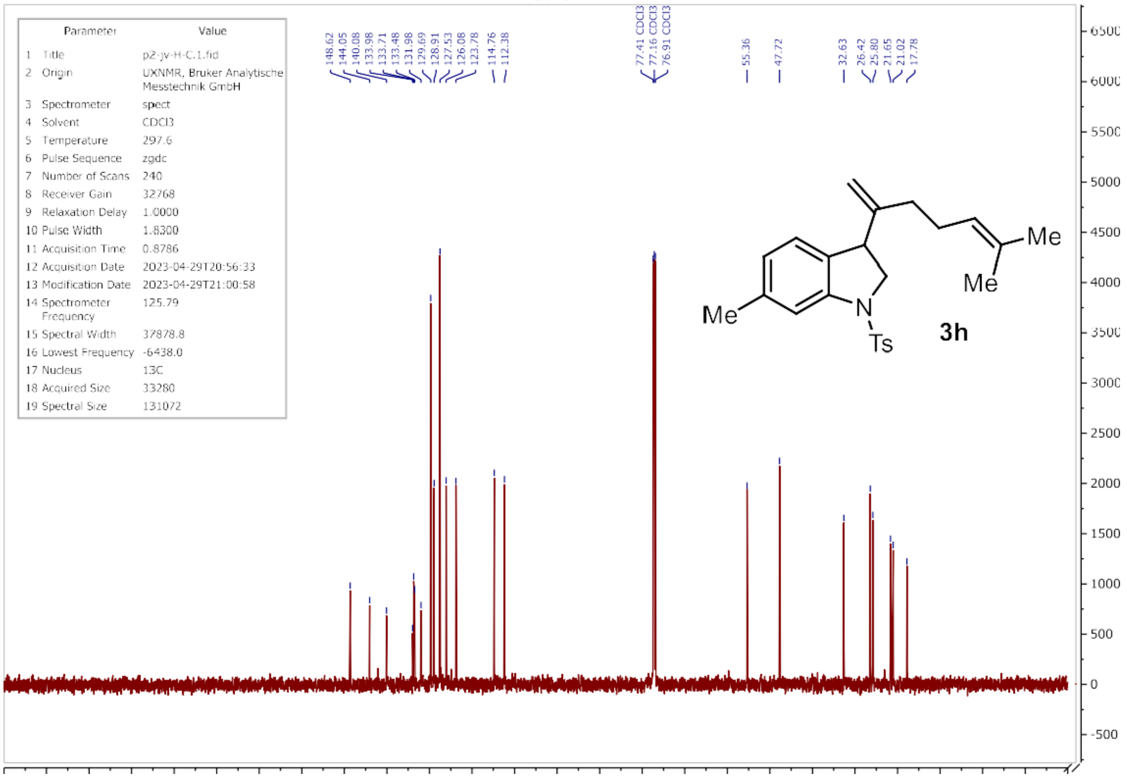

ethyl 3-(6-methylhepta-1,5-dien-2-yl)-*N*-tosylindoline-5-carboxylate (3i)

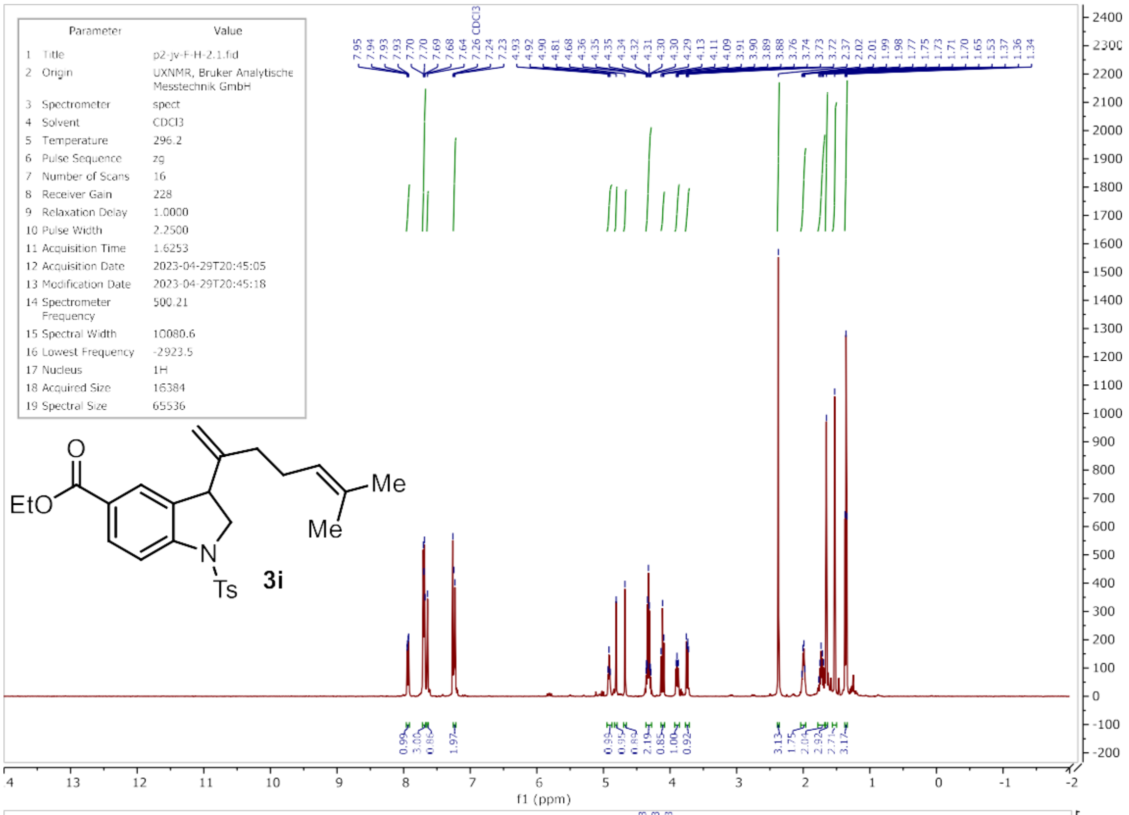

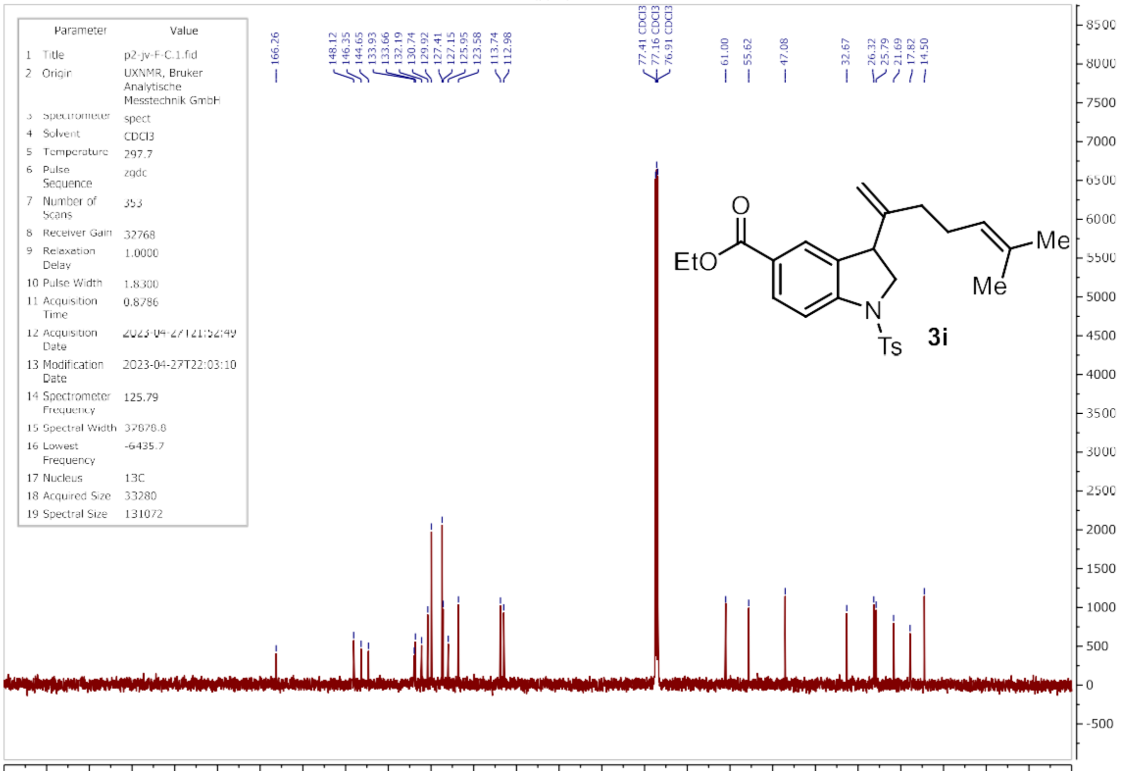

methyl 3-(6-methylhepta-1,5-dien-2-yl)-*N*-tosylindoline-6-carboxylate (3j)

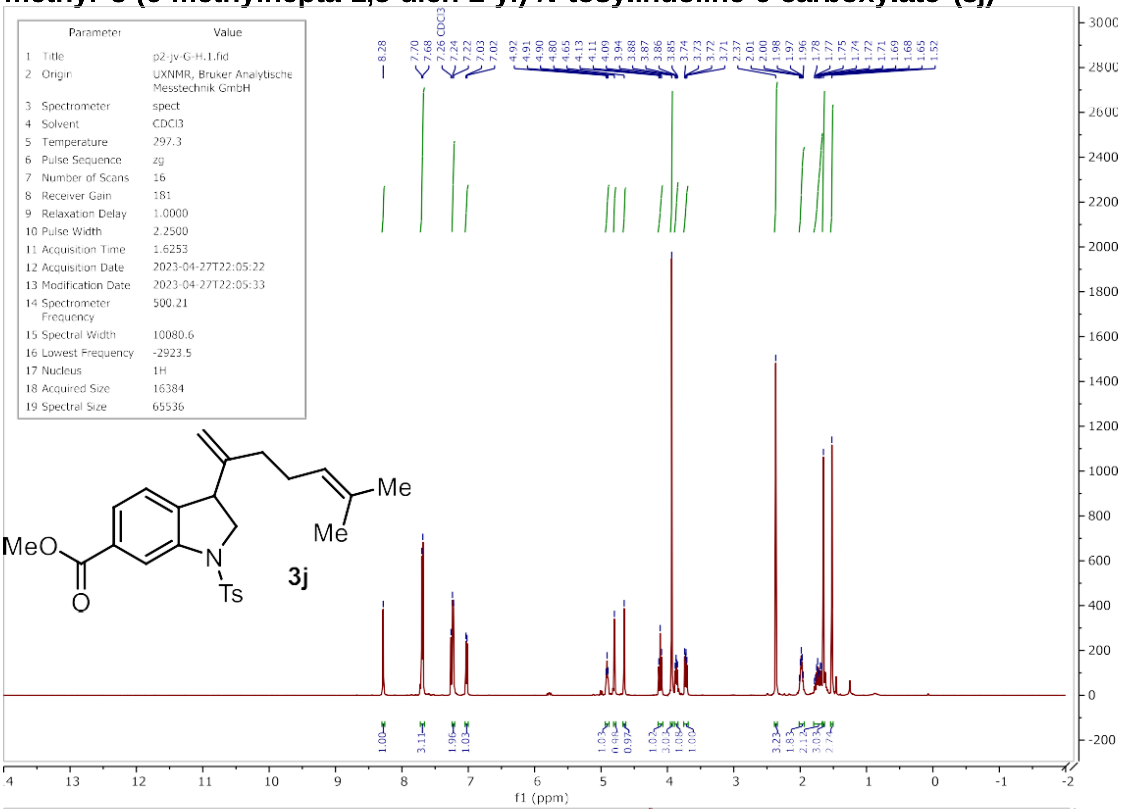

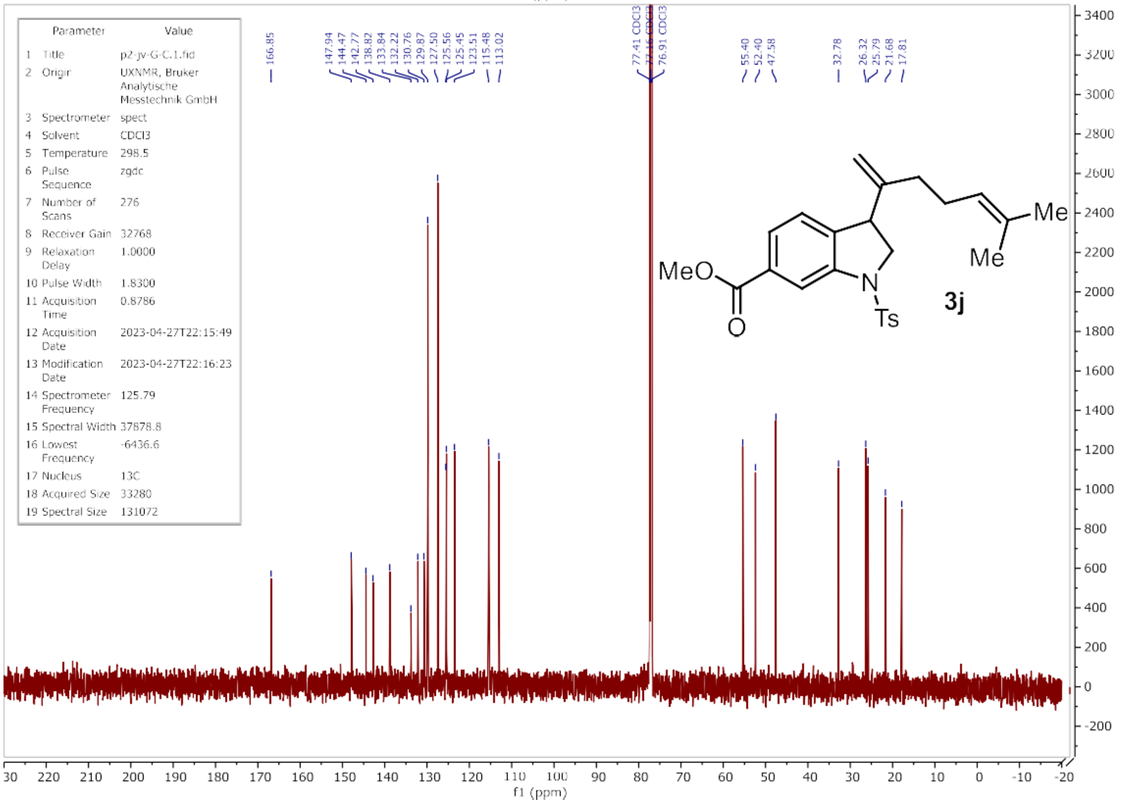

3-(6-methylhepta-1,5-dien-2-yl)-1-(methylsulfonyl)indoline (3a-Ms)

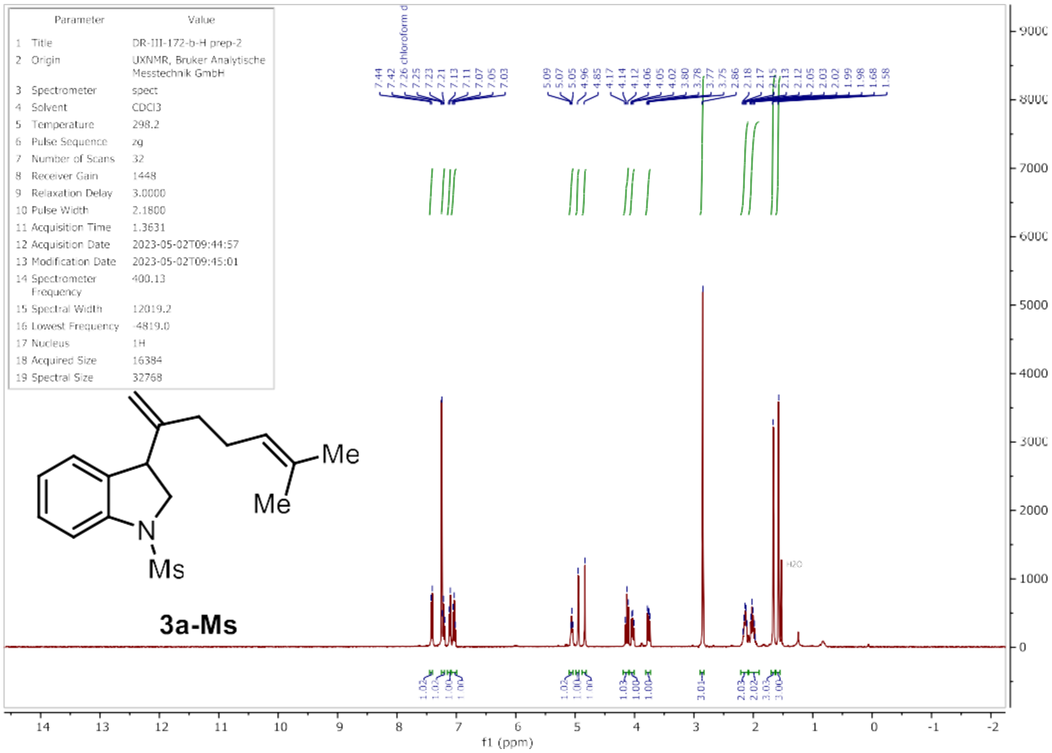

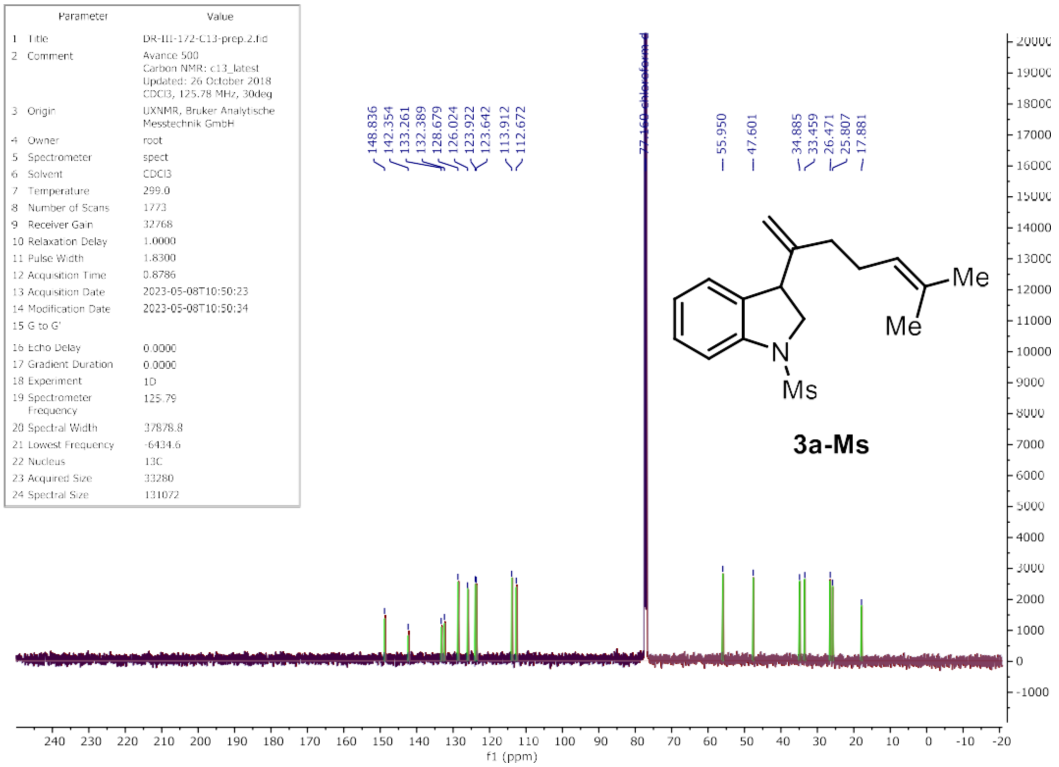

3-(6-methylhepta-1,5-dien-2-yl)-1-(methylsulfonyl)indoline (3k)

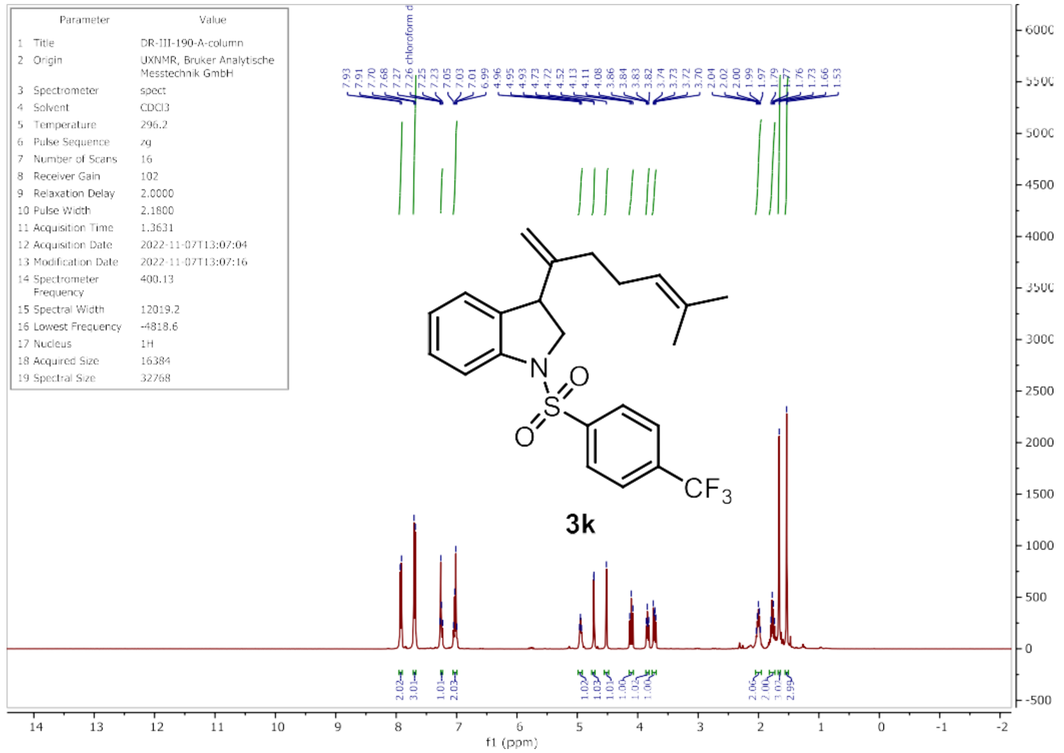

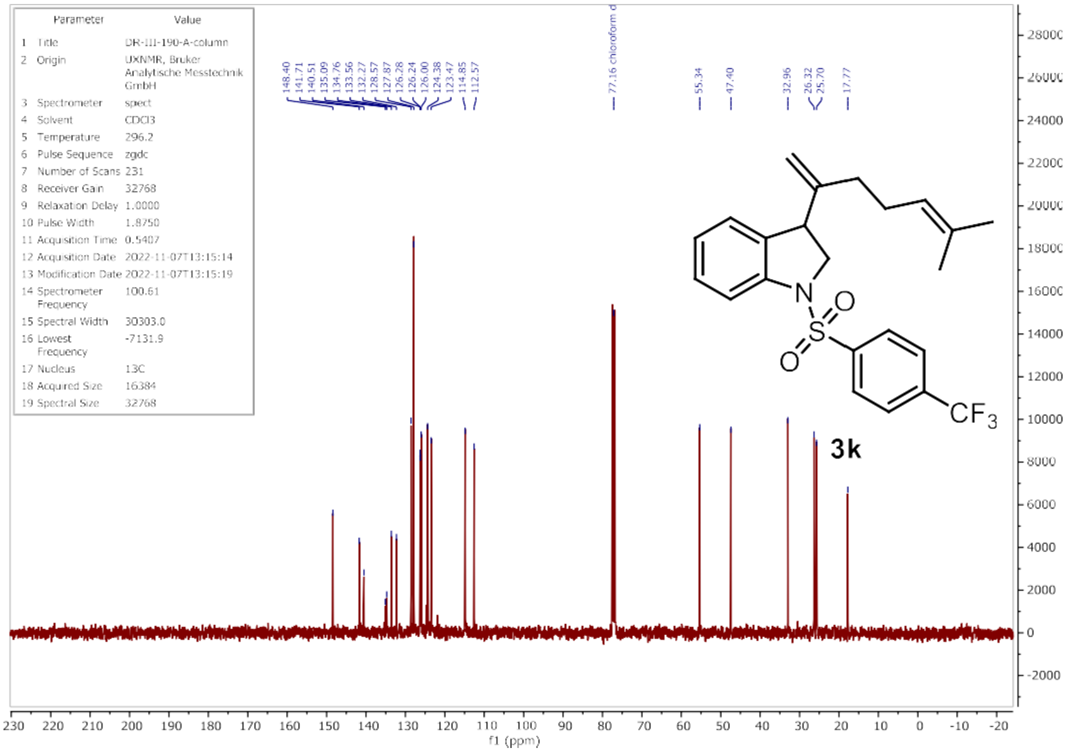

3-(prop-1-en-2-yl)-1-tosylindoline

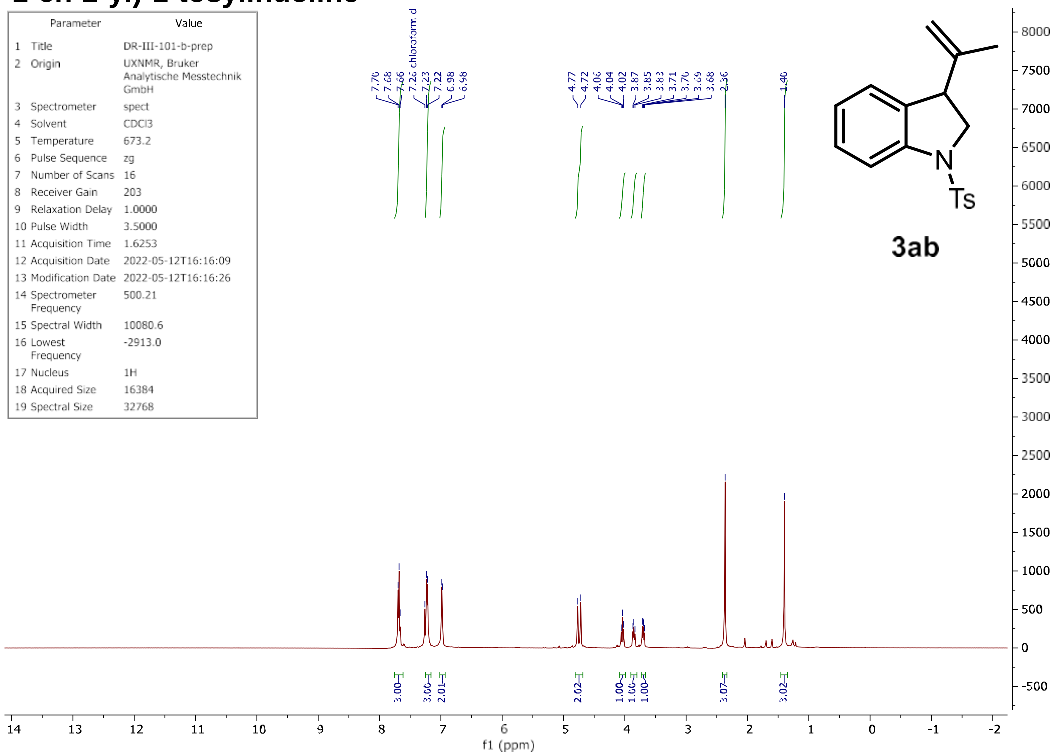

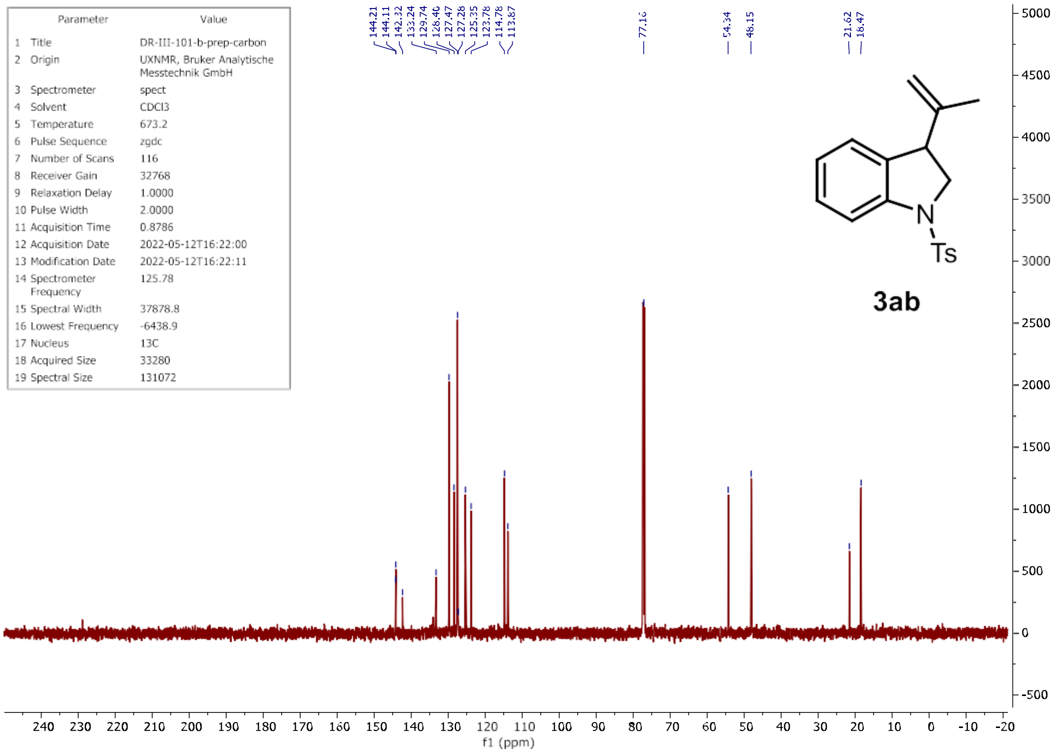

1-(methylsulfonyl)-3-(prop-1-en-2-yl)indoline (3ab-Ms)

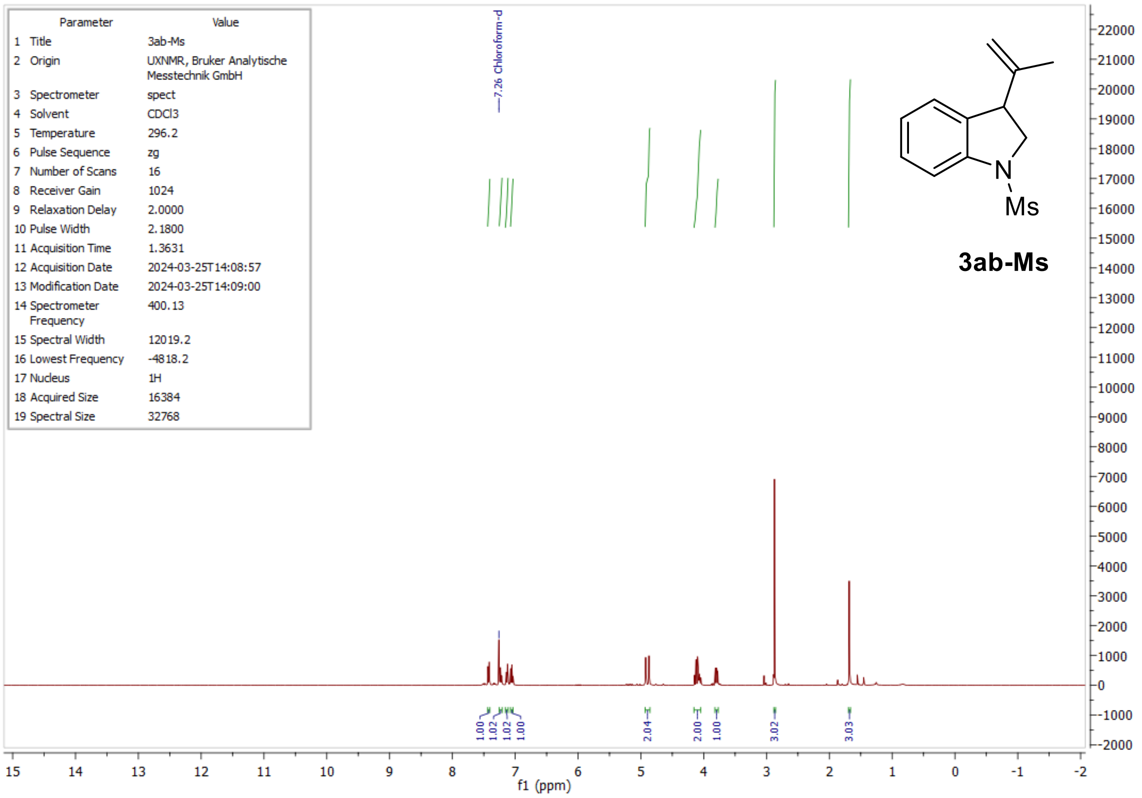

Parameter		Value
1	Title	3ab-Ms
2	Origin	UXNMR, Bruker Analytische Messtechnik GmbH
3	Spectrometer	spect
4	Solvent	CDCl3
5	Temperature	296.2
6	Pulse Sequence	zg
7	Number of Scans	16
8	Receiver Gain	1024
9	Relaxation Delay	2.0000
10	Pulse Width	2.1800
11	Acquisition Time	1.3631
12	Acquisition Date	2024-03-25T14:08:57
13	Modification Date	2024-03-25T14:09:00
14	Spectrometer Frequency	400.13
15	Spectral Width	12019.2
16	Lowest Frequency	-4818.2
17	Nucleus	1H
18	Acquired Size	16384
19	Spectral Size	32768

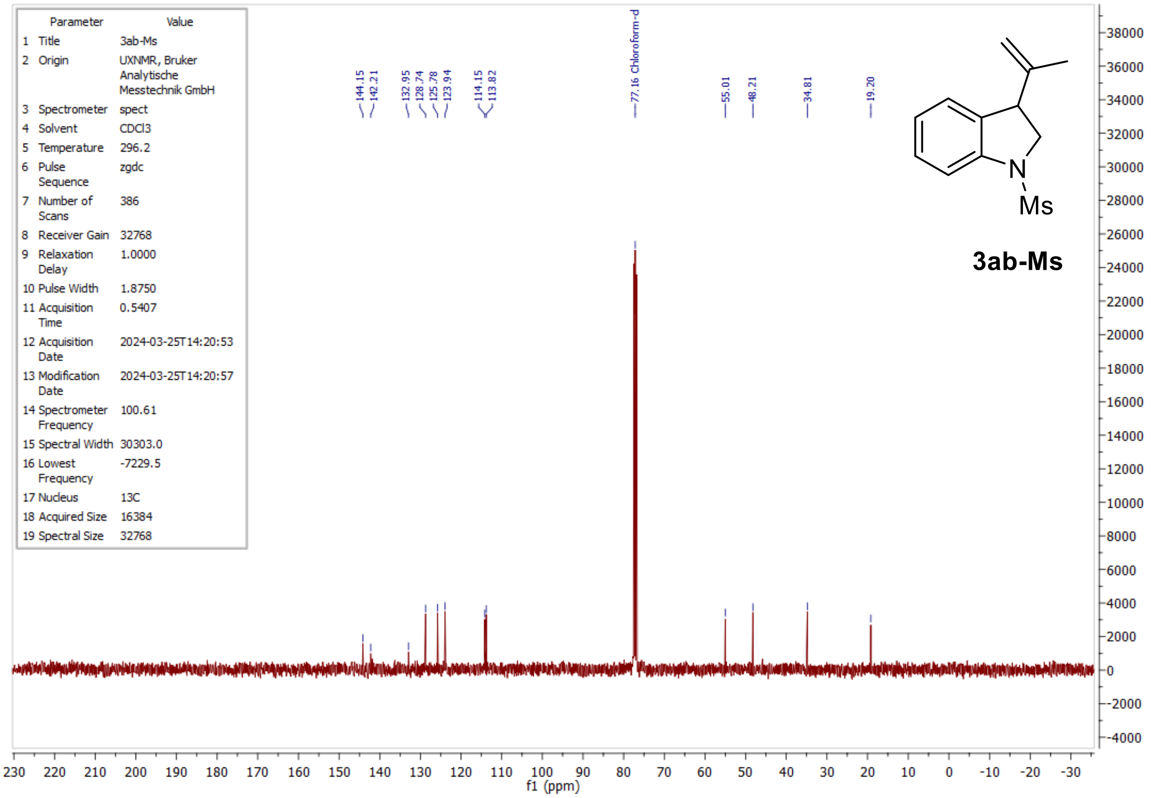

Parameter		Value
1	Title	3ab-Ms
2	Origin	UXNMR, Bruker Analytische Messtechnik GmbH
3	Spectrometer	spect
4	Solvent	CDCl3
5	Temperature	296.2
6	Pulse Sequence	zgdc
7	Number of Scans	386
8	Receiver Gain	32768
9	Relaxation Delay	1.0000
10	Pulse Width	1.8750
11	Acquisition Time	0.5407
12	Acquisition Date	2024-03-25T14:20:53
13	Modification Date	2024-03-25T14:20:57
14	Spectrometer Frequency	100.61
15	Spectral Width	30303.0
16	Lowest Frequency	-7229.5
17	Nucleus	13C
18	Acquired Size	16384
19	Spectral Size	32768

3-(3-phenylprop-1-en-2-yl)-1-tosylindoline (3ac)

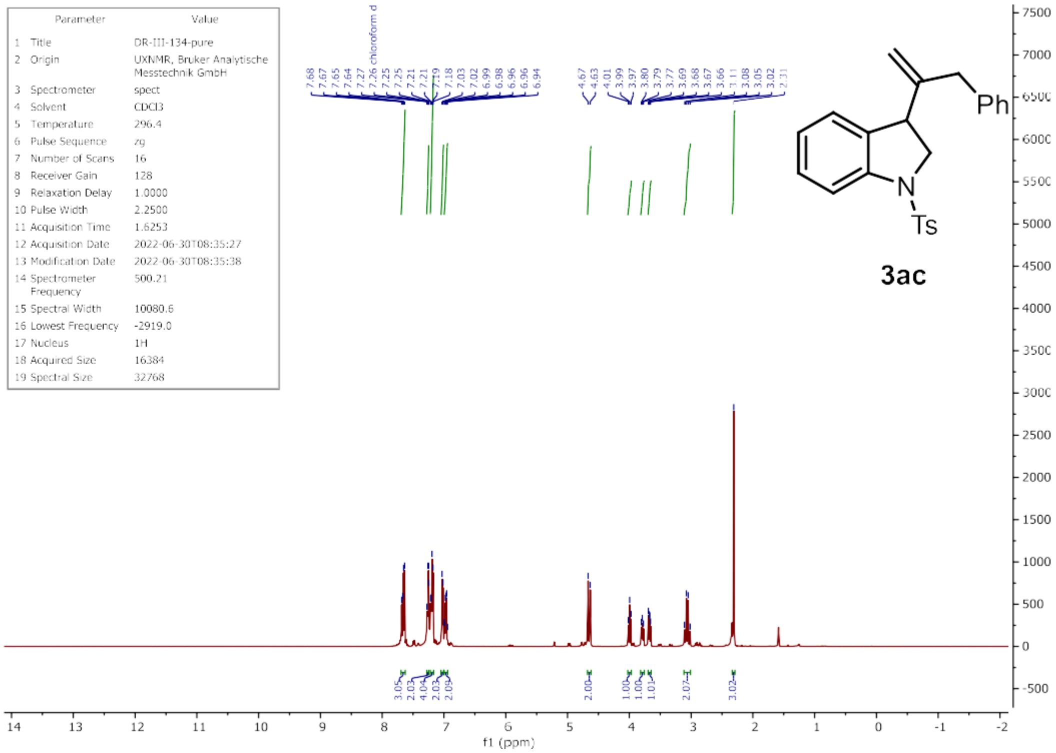

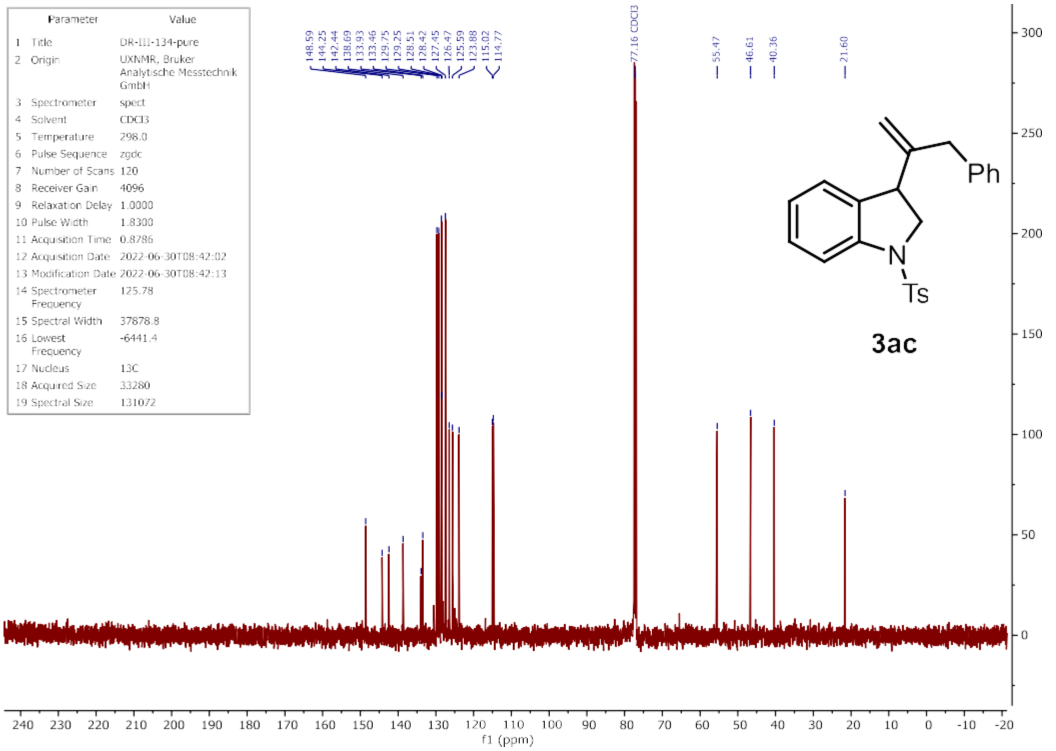

3-(1-phenylvinyl)-1-tosylindoline (3ad)

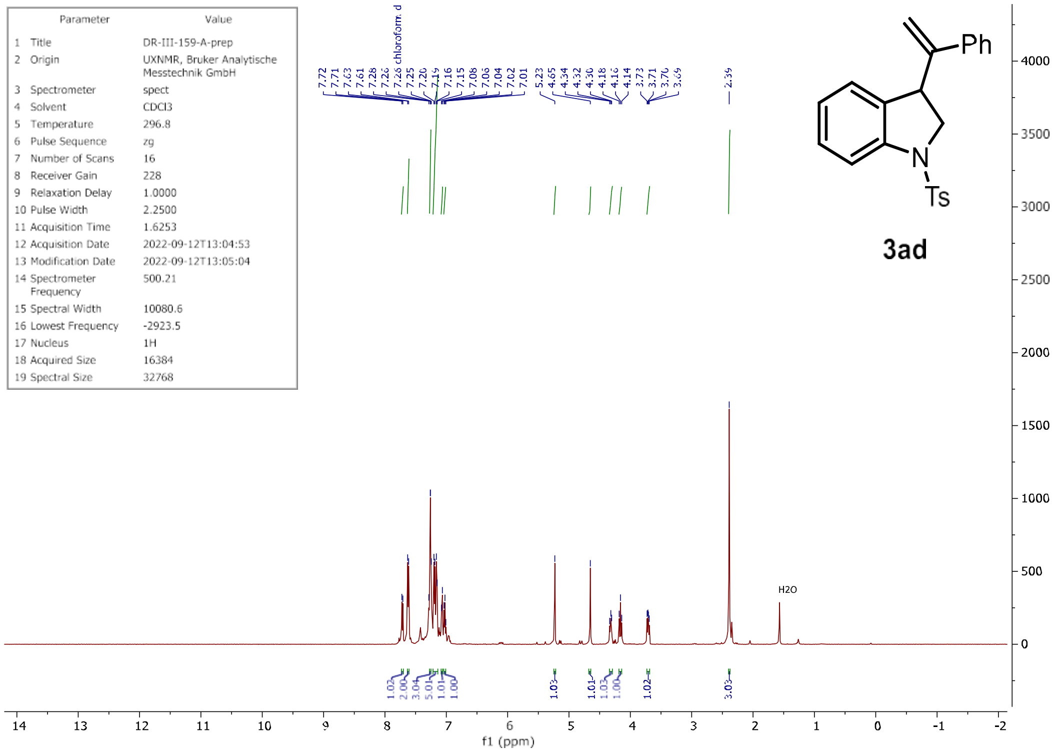

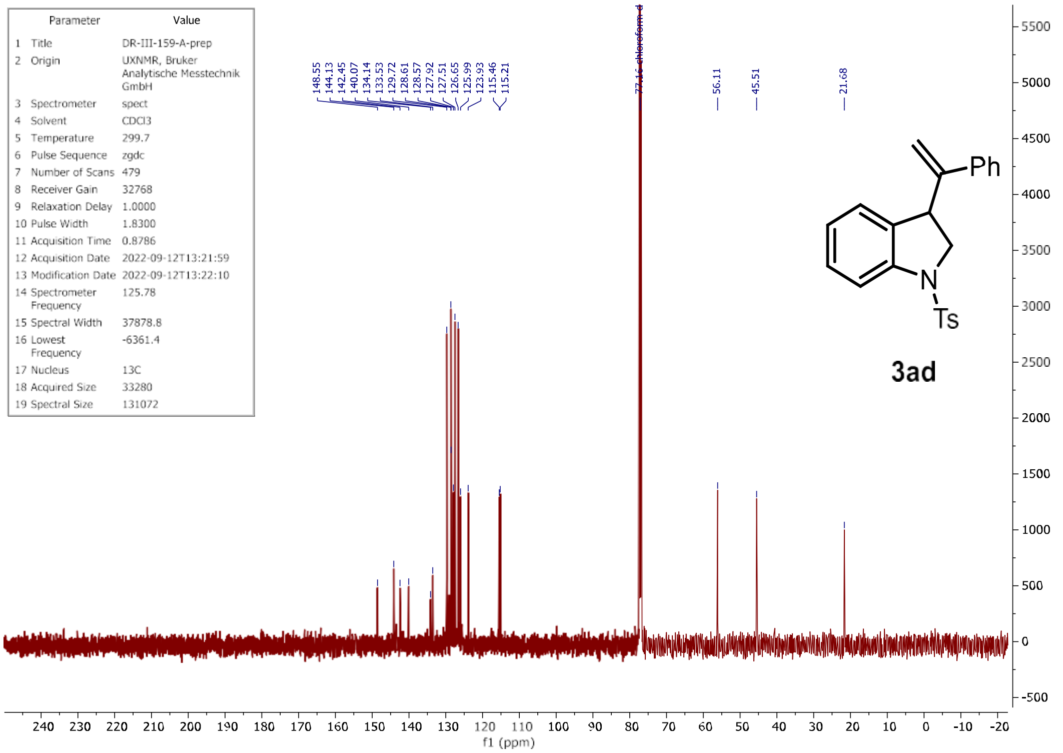

3-(5,5-dimethoxypent-1-en-2-yl)-1-tosylindoline (3ae)

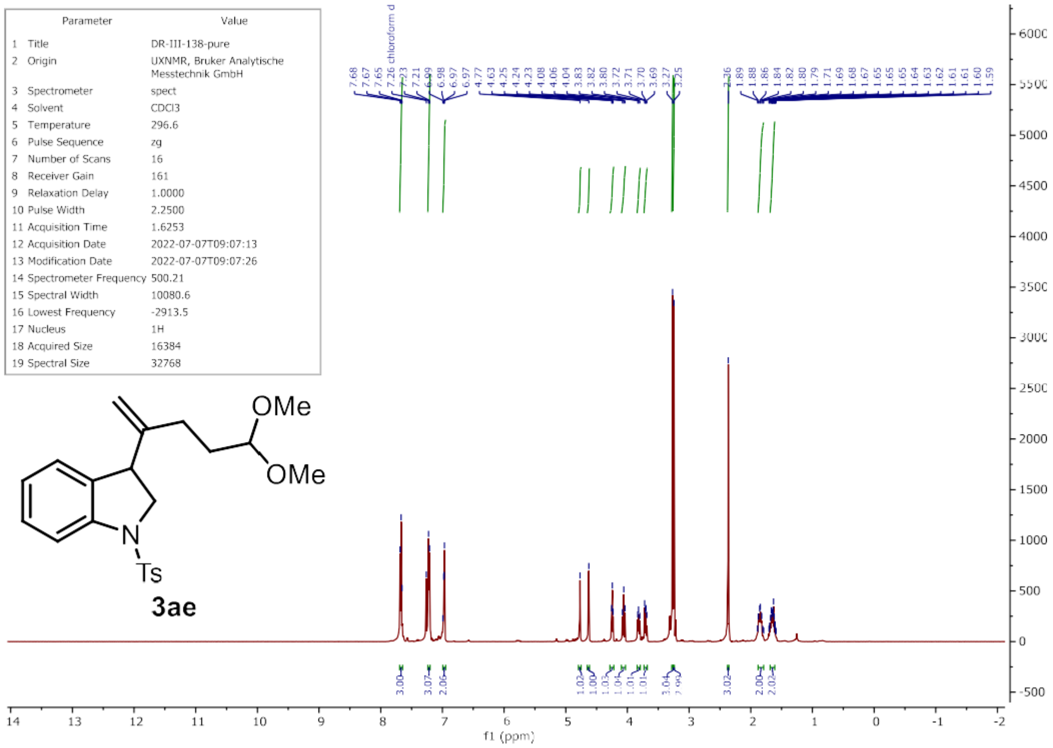

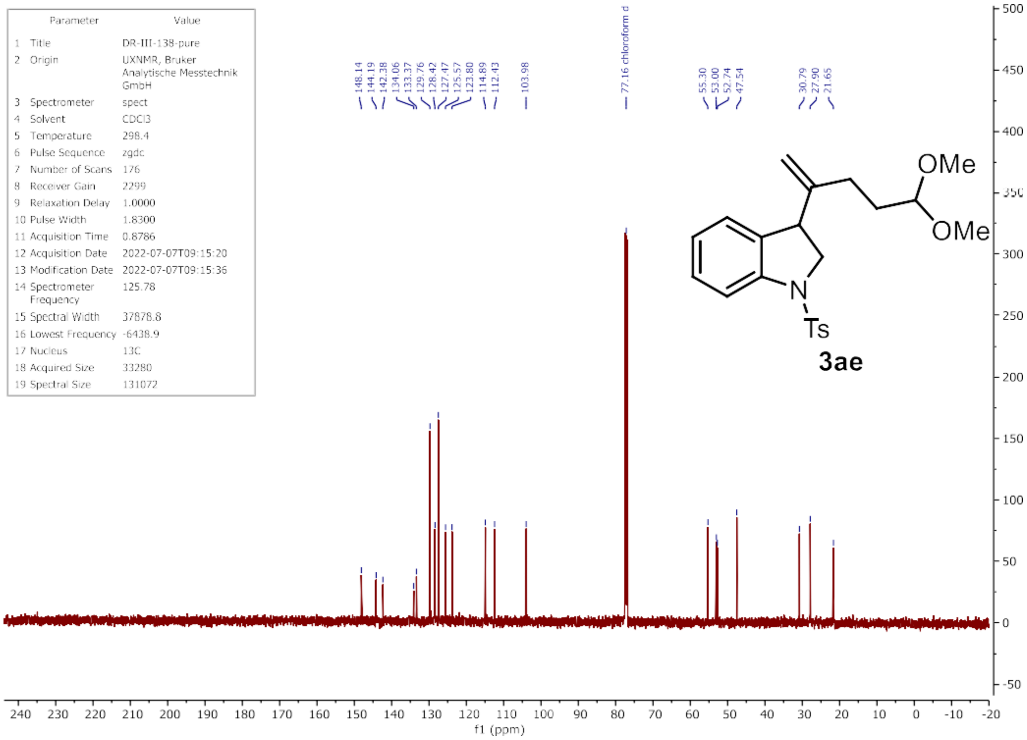

3-(5-((*tert*-butyldimethylsilyl)oxy)pent-1-en-2-yl)-1-tosylindoline (3af)

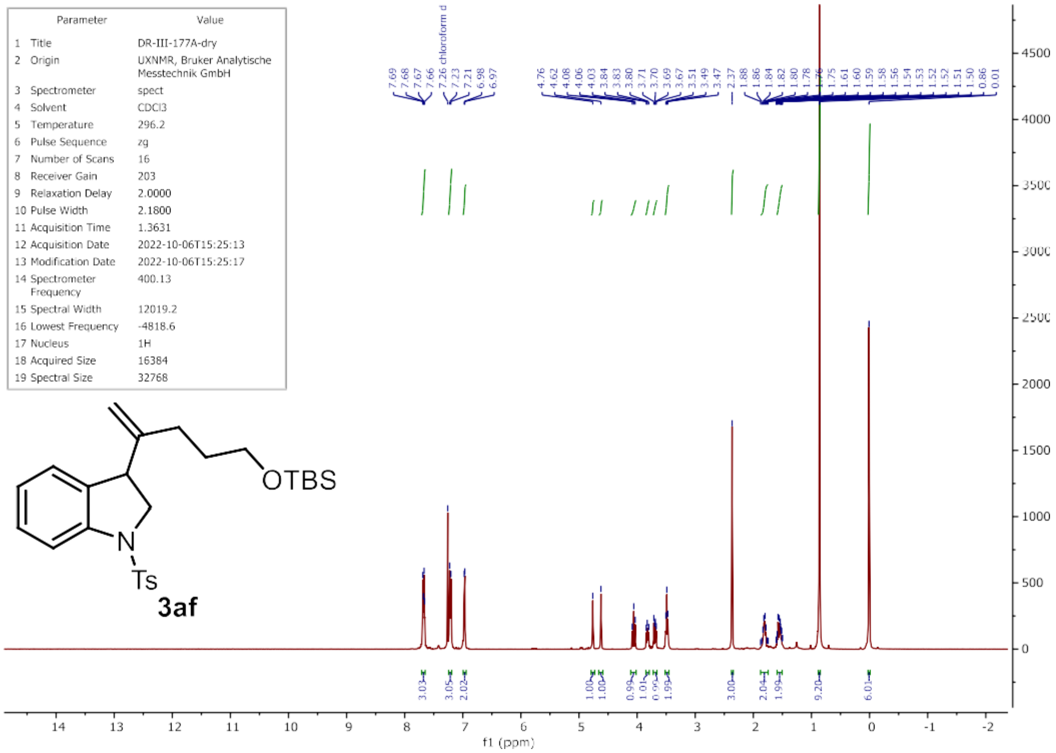

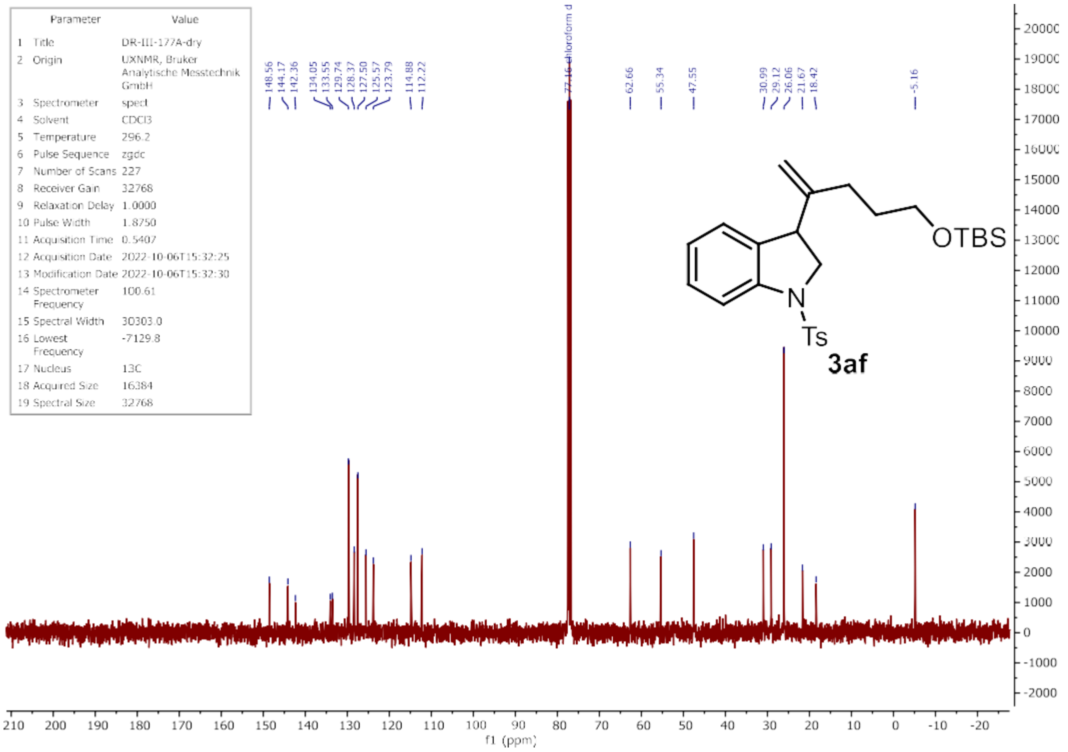

2-(4-(1-tosylindolin-3-yl)pent-4-en-1-yl)isoindoline-1,3-dione (3ag)

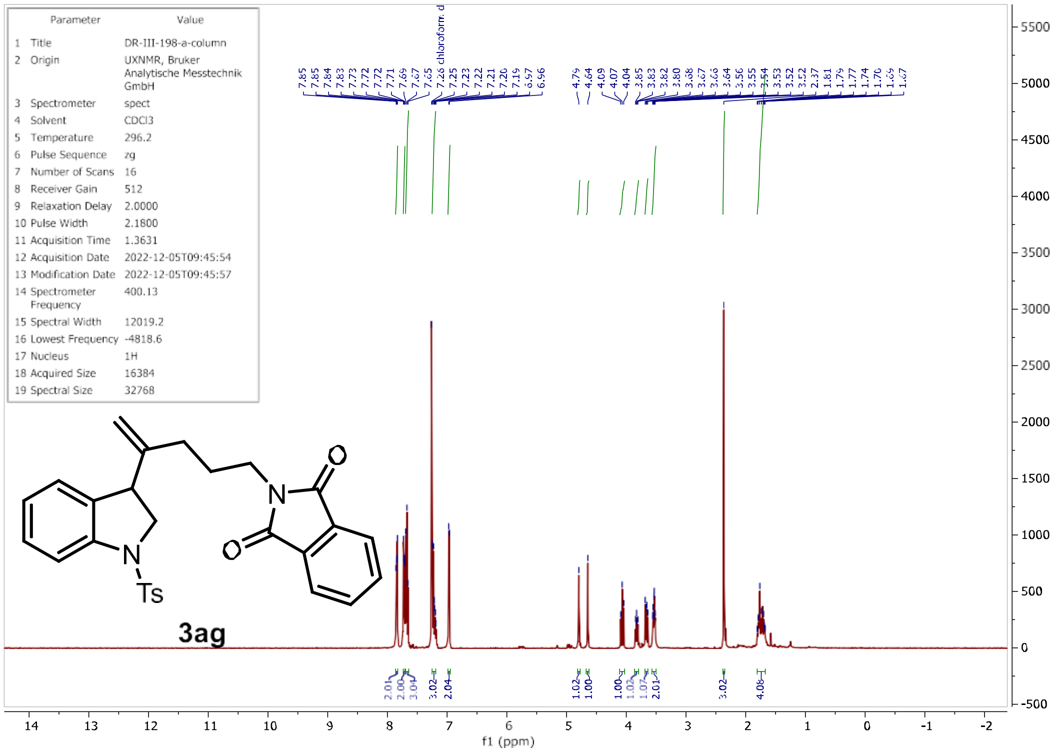

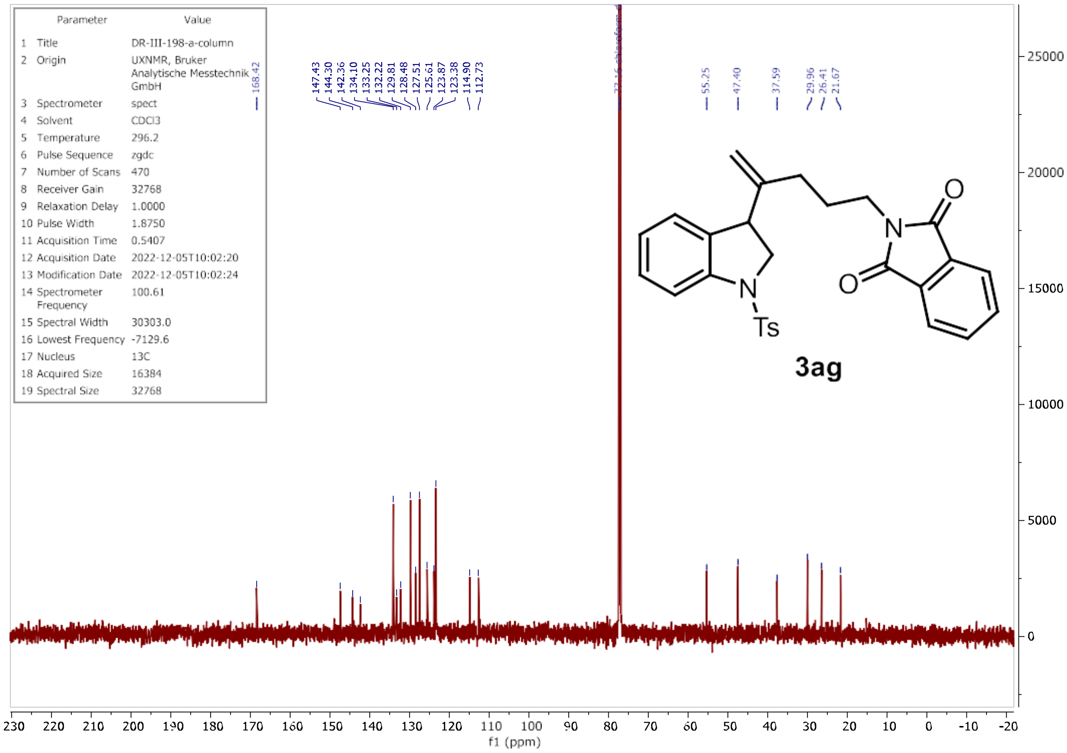

3-(4-(3,3-dimethyloxiran-2-yl)but-1-en-2-yl)-1-tosylindoline (3ah)

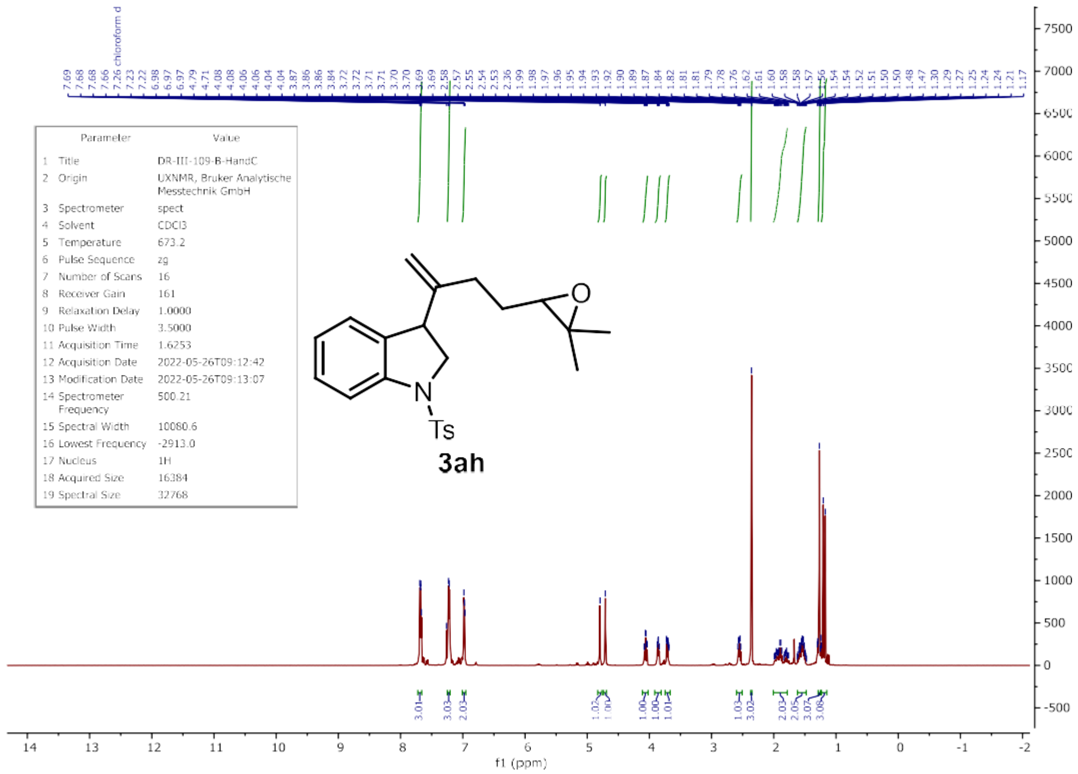

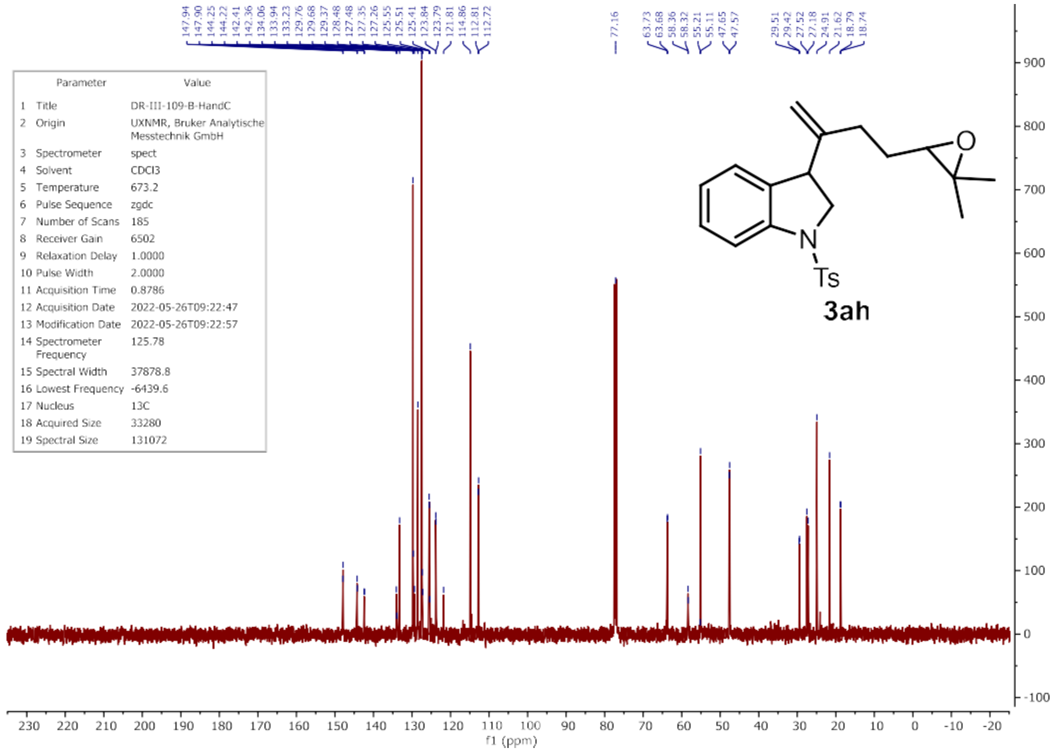

3-(5-(3-(furan-2-yl)propoxy)pent-1-en-2-yl)-1-tosylindoline (3ai)

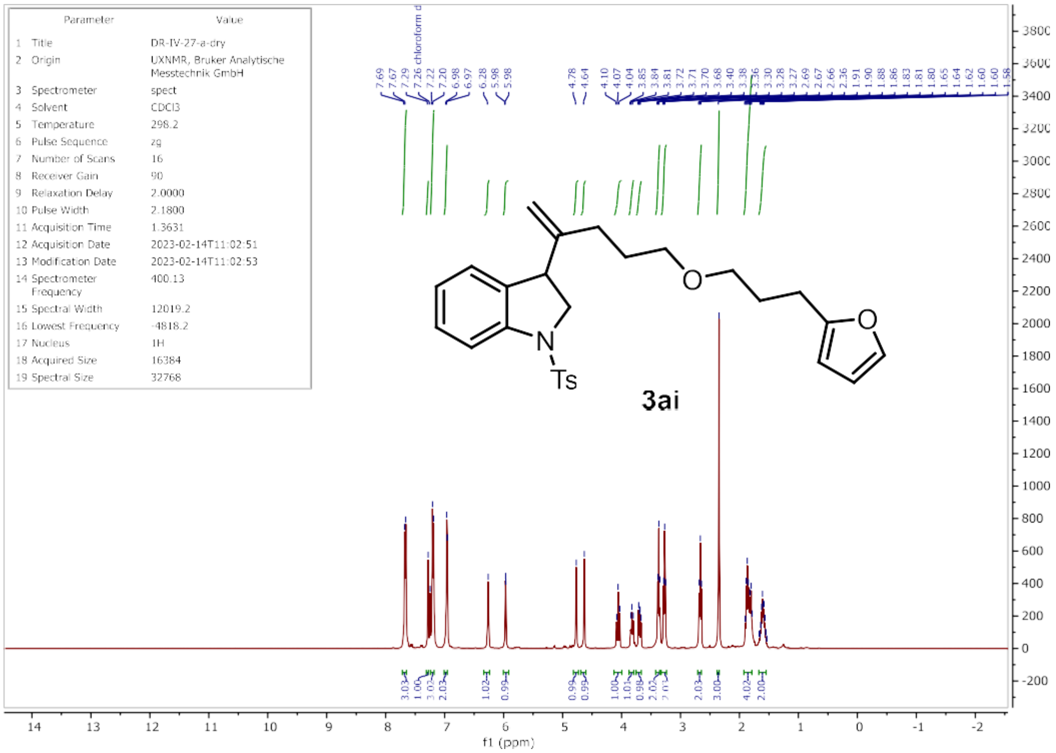

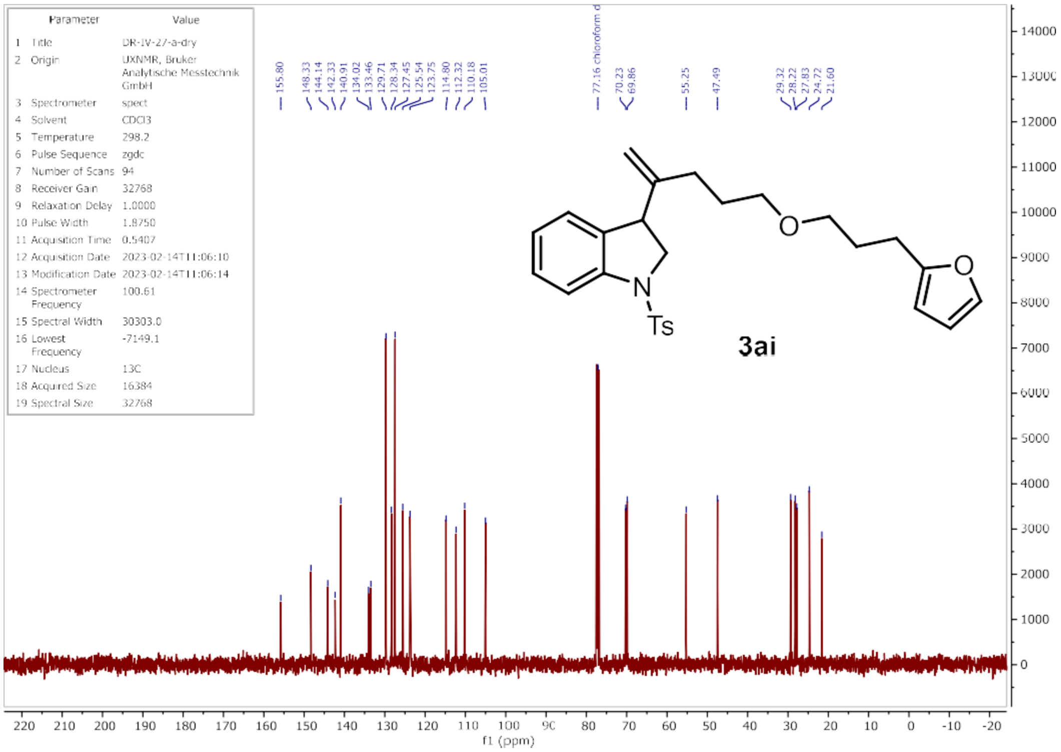

1-methyl-2-(((4-(1-tosylindolin-3-yl)pent-4-en-1-yl)oxy)methyl)-1*H*-indole (3aj)

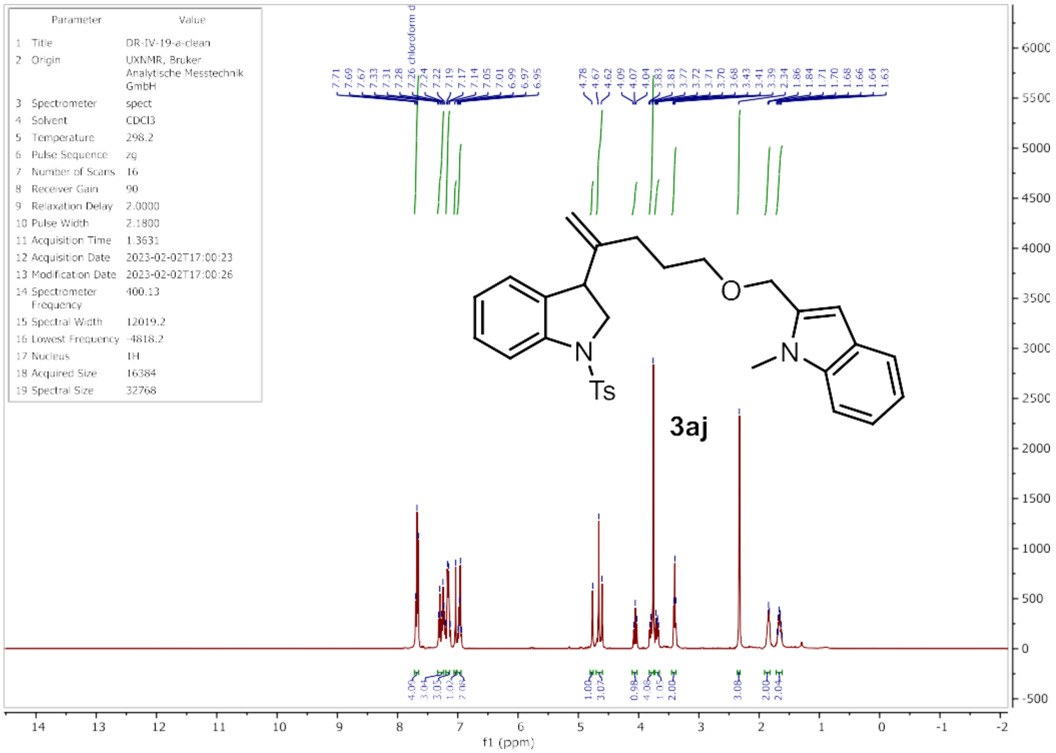

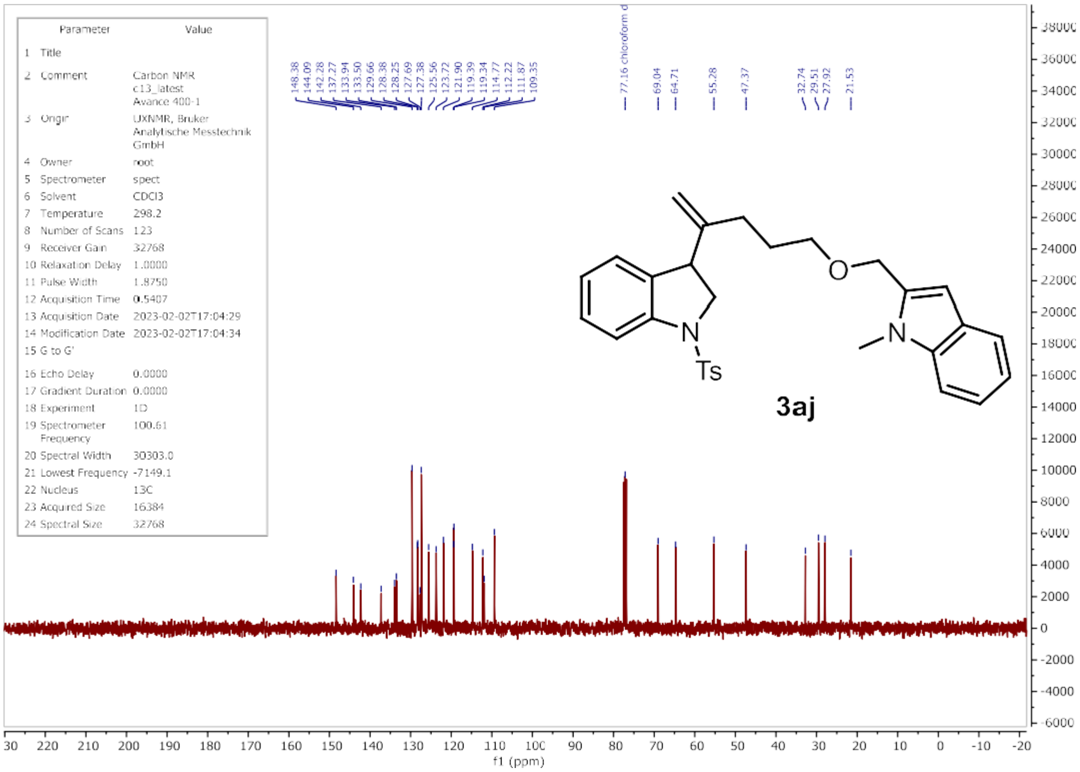

3-(1-((3*r*,5*r*,7*r*)-adamantan-1-yl)vinyl)-1-tosylindoline (3ak)

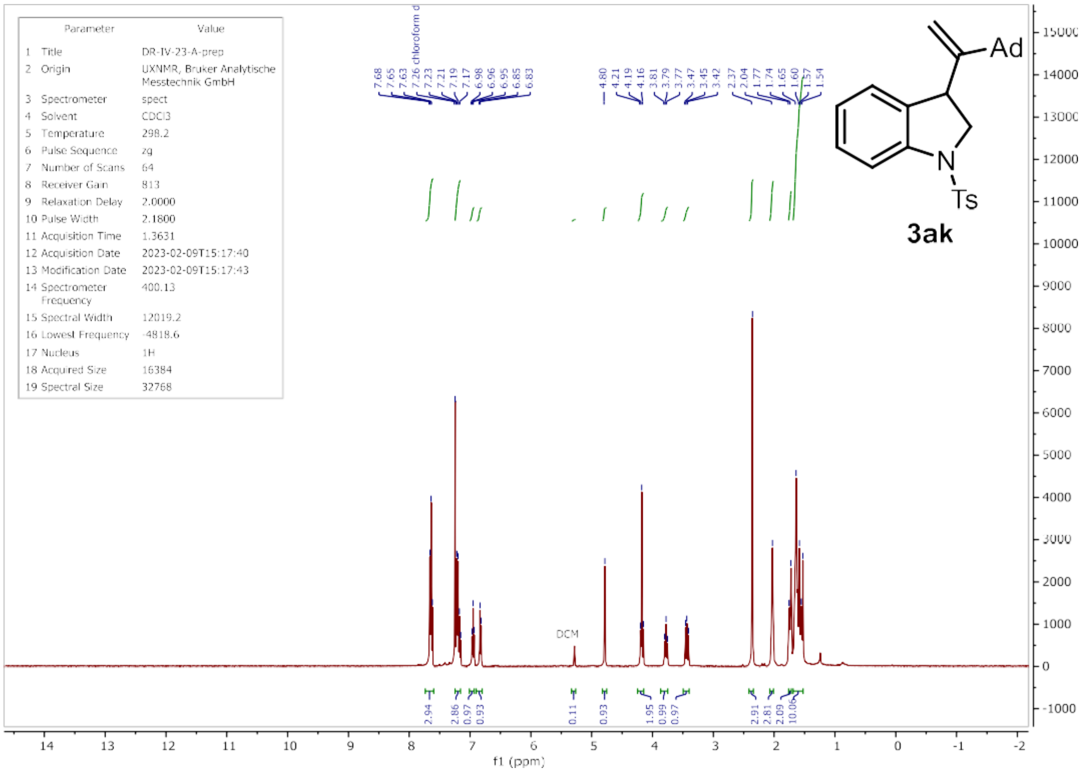

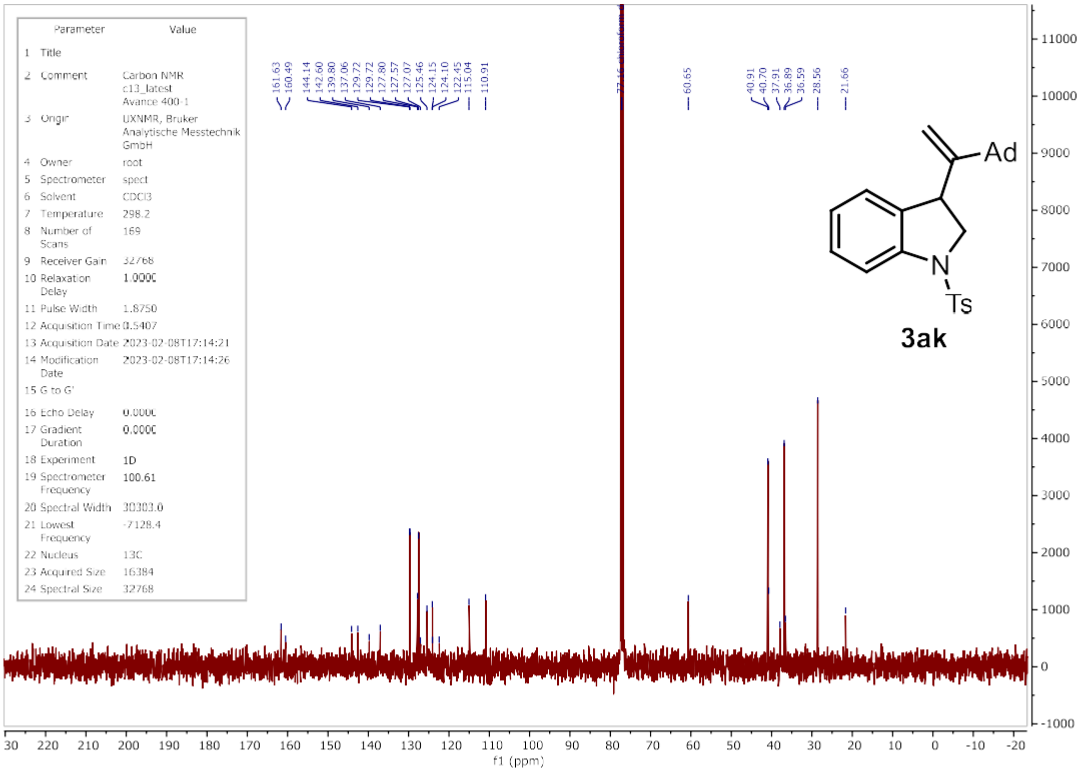

3-((3*r*,5*r*,7*r*)-adamantan-1-yl)-1-tosyl-3-vinylindoline (5ak)

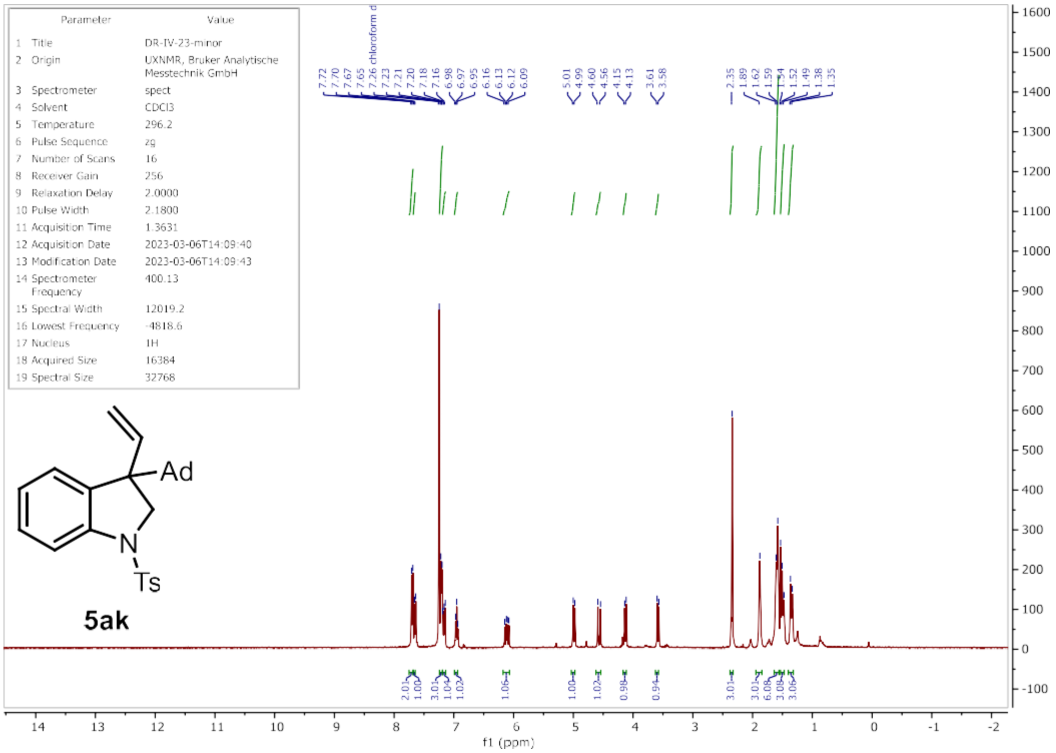

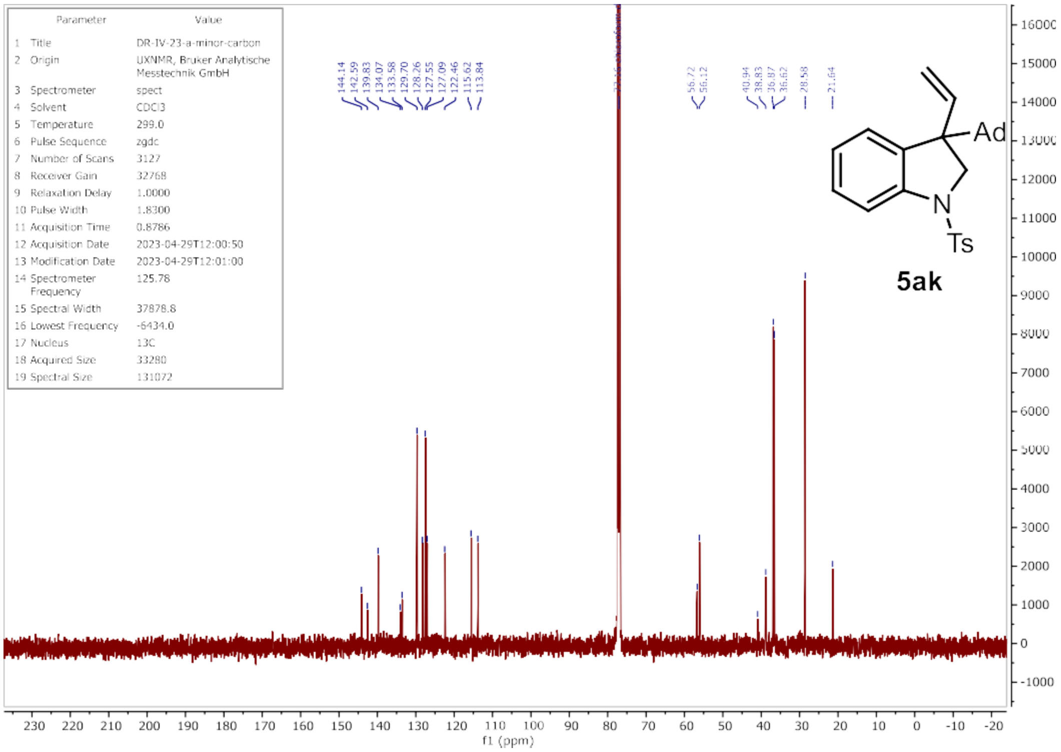

2-phenyl-1-tosyl-3-vinylindoline (6al)

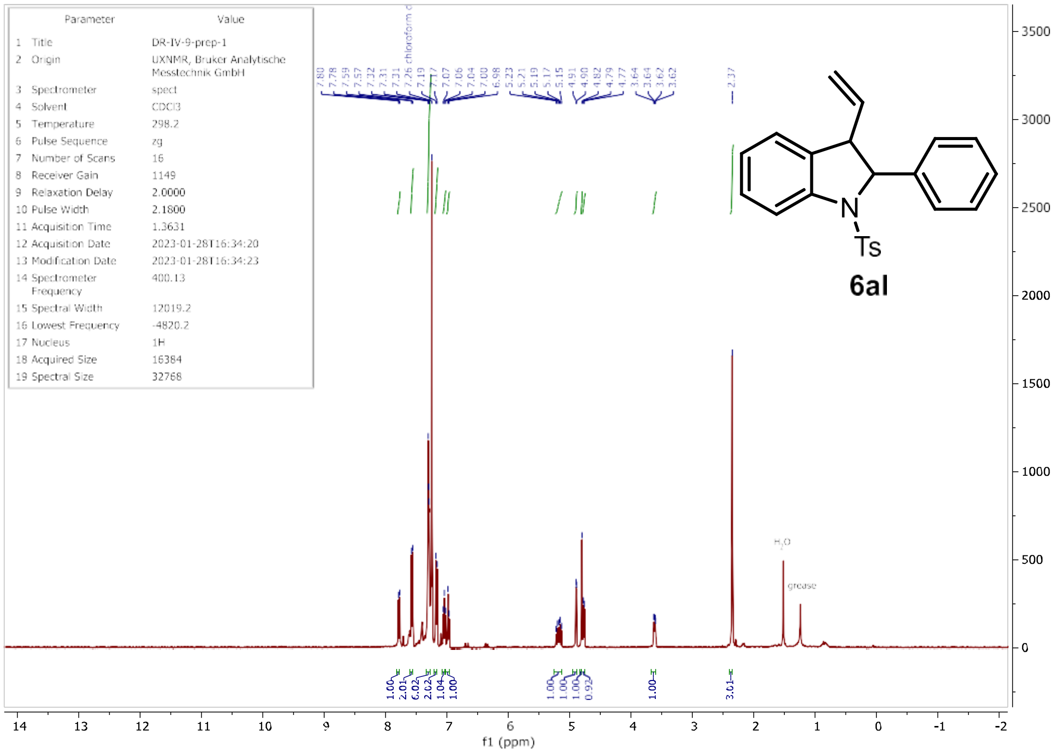

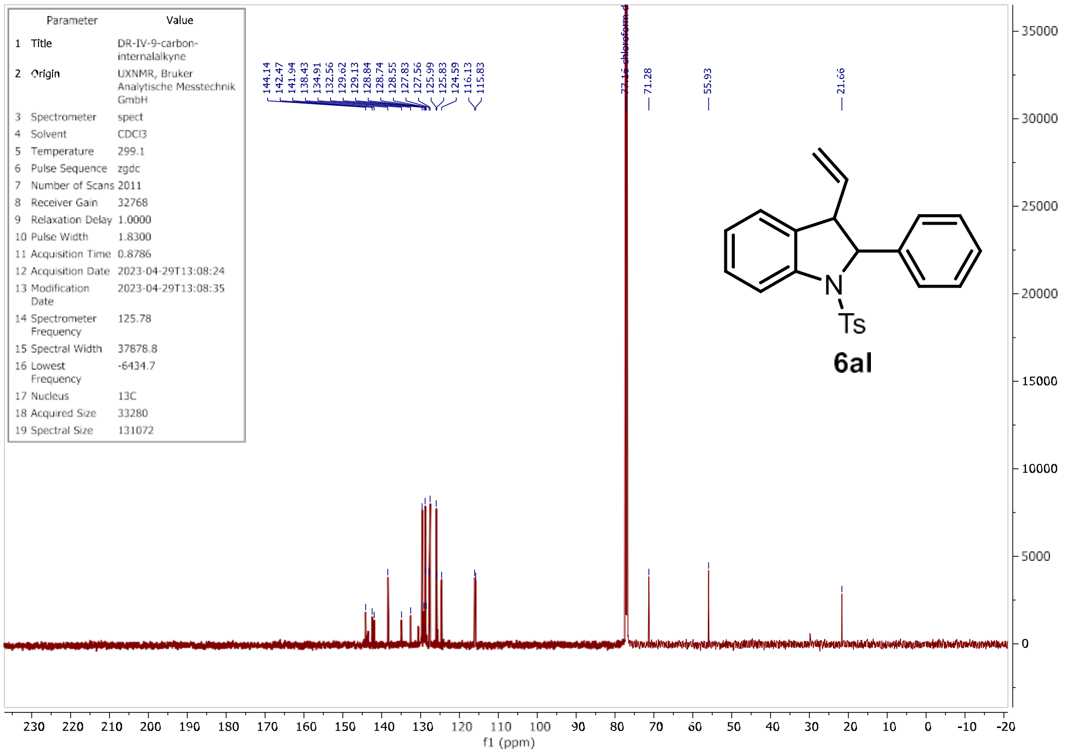

3-(6-methylhepta-1,5-dien-2-yl)indoline (3l)

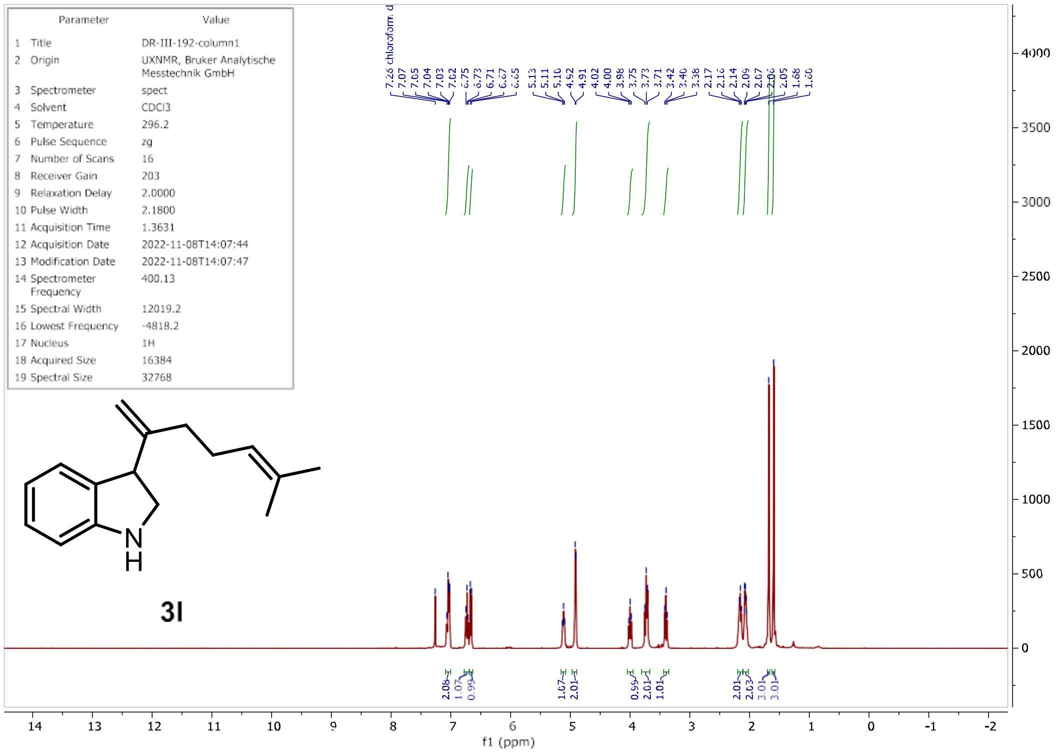

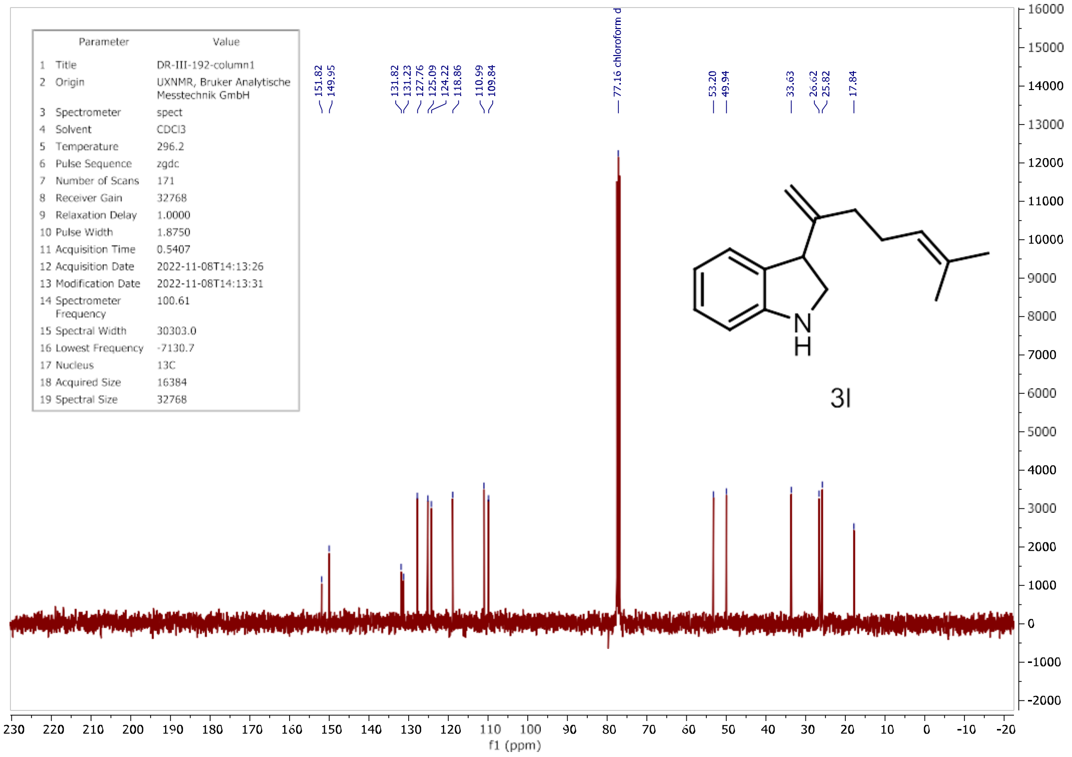

Supplementary References

1. Vaith, J.; Rodina, D.; Spaulding, G. C.; Paradine, S. M., Pd-Catalyzed Heteroannulation Using N-Arylureas as a Sterically Undemanding Ligand Platform. *Journal of the American Chemical Society* **2022,** *144* (15), 6667-6673.

2. Li, H.; Yang, Q.; Xu, L.; Wei, J.; Tang, Y.; Cai, Y., Cu(I)/Chiral Vanadium Complex Cooperatively Catalyzed Asymmetric Sulfonation/Rearrangement of Alkenylfurans. *Organic Letters* **2022,** *24* (44), 8202-8207.

3. Chen, D.; Chen, Y.; Ma, Z.; Zou, L.; Li, J.; Liu, Y., One-Pot Synthesis of Indole-3-acetic Acid Derivatives through the Cascade Tsuji–Trost Reaction and Heck Coupling. *The Journal of Organic Chemistry* **2018,** *83* (12), 6805-6814.

4. Park, S. R.; Findlay, N. J.; Garnier, J.; Zhou, S.; Spicer, M. D.; Murphy, J. A., Electron transfer activity of a cobalt crown carbene complex. *Tetrahedron* **2009,** *65* (52), 10756-10761.

5. Sen, S.; Singh, S.; Sieburth, S. M., A Practical, Two-Step Synthesis of 2-Substituted 1,3-Butadienes. *The Journal of Organic Chemistry* **2009,** *74* (7), 2884-2886.

6. Wu, J. Y.; Moreau, B.; Ritter, T., Iron-Catalyzed 1,4-Hydroboration of 1,3-Dienes. *Journal of the American Chemical Society* **2009,** *131* (36), 12915-12917.

7. Jiang, L.; Cao, P.; Wang, M.; Chen, B.; Wang, B.; Liao, J., Highly Diastereo- and Enantioselective Cu-Catalyzed Borylative Coupling of 1,3-Dienes and Aldimines. *Angewandte Chemie International Edition* **2016,** *55* (44), 13854-13858.

8. Maity, A. K.; Kalb, A. E.; Zeller, M.; Uyeda, C., A Dinickel Catalyzed Cyclopropanation without the Formation of a Metal Carbene Intermediate. *Angewandte Chemie International Edition* **2021,** *60* (4), 1897-1902.

9. Houghtling, K. E.; Canfield, A. M.; Paradine, S. M., Convergent Synthesis of Dihydrobenzofurans via Urea Ligand-Enabled Heteroannulation of 2-Bromophenols with 1,3-Dienes. *Organic Letters* **2022,** *24* (31), 5787-5790.

10. Gensch, T.; dos Passos Gomes, G.; Friederich, P.; Peters, E.; Gaudin, T.; Pollice, R.; Jorner, K.; Nigam, A.; Lindner-D'Addario, M.; Sigman, M. S.; Aspuru-Guzik, A. A Comprehensive Discovery Platform for Organophosphorus Ligands for Catalysis. *J. Am. Chem. Soc.* **2022**, *144*, 1205−1217.

11. Gaussian 16, Revision A.03, Frisch, M. J.; Trucks, G. W.; Schlegel, H. B.; Scuseria, G. E.; Robb, M. A.; Cheeseman, J. R.; Scalmani, G.; Barone, V.; Petersson, G. A.; Nakatsuji, H.; Li, X.; Caricato, M.; Marenich, A. V.; Bloino, J.; Janesko, B. G.; Gomperts, R.; Mennucci, B.; Hratchian, H. P.; Ortiz, J. V.; Izmaylov, A. F.; Sonnenberg, J. L.; Williams-Young, D.; Ding, F.; Lipparini, F.; Egidi, F.; Goings, J.; Peng, B.; Petrone, A.; Hender-son, T.; Ranasinghe, D.; Zakrzewski, V. G.; Gao, J.; Rega, N.; Zheng, G.; Liang, W.; Hada, M.; Ehara, M.; Toyota, K.; Fukuda, R.; Haseg-awa, J.; Ishida, M.; Nakajima, T.; Honda, Y.; Kitao, O.; Nakai, H.; Vreven, T.; Throssell, K.; Montgomery, J. A., Jr.; Peralta, J. E.; Ogli-aro, F.; Bearpark, M. J.; Heyd, J. J.; Brothers, E. N.; Kudin, K. N.; Staroverov, V. N.; Keith, T. A.; Kobayashi, R.; Normand, J.; Raghavachari, K.; Rendell, A. P.; Burant, J. C.; Iyengar, S. S.; To-masi, J.; Cossi, M.; Millam, J. M.; Klene, M.;

Adamo, C.; Cammi, R.; Ochterski, J. W.; Martin, R. L.; Morokuma, K.; Farkas, O.; For-esman, J. B.; Fox, D. J. Gaussian, Inc., Wallingford CT, 2016.

12. Marenich, A. V.; Cramer, C. J.; Truhlar, D. G. Universal solvation model based on solute electron density and a continuum model of the solvent defined by the bulk dielectric constant and atomic surface tensions, *J. Phys. Chem. B* **2009**, *113*, 6378-6396.

13. a) Becke, A. D. *J. Chem. Phys.* **1993**, *98*, 5648−5652. c) Lee, C.; Yang, W.; Parr, R. G. *Phys. Rev. B* **1988**, *37*, 785−789. c) Curtiss, L. A.; Raghavachari, K.; Redfern, P. C.; Rassolov, V.; Pople, J. A. Gaussian-3 (G3) theory for molecules containing first and second-row atoms. *J. Chem. Phys.* **1998**, *109*, 7764 - 7776.

14. a) Becke, A. D. Density-functional exchange-energy approximation with correct asymptotic behavior. *Phys. Rev. A* **1988**, *38*, 3098−3100.

15. Chaia, J.-D.; Head-Gordon, M. Long-range corrected hybrid density functionals with damped atom–atom dispersion corrections. *Phys. Chem. Chem. Phys.* **2008**, *10*, 6615-6620.

16. a) Lefebvre, C.; Khartabil, H.; Boisson, J.-C.; ContrerasGarcía, J.; Piquemal, J.-P.; Hénon, E. The Independent Gradient Model: A New Approach for Probing Strong and weak Interactions in Molecules from Wave Function Calculations. *ChemPhysChem* **2018**, *19*, 724−735. b) Lu, T.; Chen, R. Multiwfn: A multifunctional wavefunction analyzer. *J. Comput. Chem.* **2012**, *33*, 580-592. c) Pettersen, E. F.; Goddard, T. D., Huang, C. C.; Meng, E. C.; Couch, G. S.; Croll T. I.; Morris, J. H.; Ferrin, T. E. UCSF ChimeraX: Structure visualization for researchers, educators, and developers. *Protein Sci.* **2021**, *30*, 70-82.
