# Supplementary material for: Ligand control of regioselectivity in palladium-catalyzed heteroannulation reactions of 1,3-Dienes
Source: Nat Commun. 2024 Jun 26;15:5433. doi: 10.1038/s41467-024-49803-y (PMC11208576; doi:10.1038/s41467-024-49803-y)
Supplement: Supplementary file 3 — Description of Additional Supplementary Files [file 41467_2024_49803_MOESM3_ESM.docx]

**File Name:** Supplementary Code 1

**Description:** Python programming code for the algorithm used to establish the linear regression model
